# Conformational Control of Excited‐State Properties in *Ortho*‐Linked Triarylamine‐Based Donor–Acceptor Conjugates

**DOI:** 10.1002/open.70308

**Published:** 2026-09-30

**Authors:** Monika Flörke, Thomas J. J. Müller

**Affiliations:** ^1^ Faculty of Mathematics and Natural Sciences Institut für Organische Chemie und Makromolekulare Chemie Heinrich‐Heine‐Universität Düsseldorf Düsseldorf Germany

**Keywords:** cyclic voltammetry, donor–acceptor‐systems, fluorescence, *ortho*‐triarylamine emitters, time‐dependent density functional theory calculations

## Abstract

A series of intensely blue‐emissive *ortho*‐linked donor–acceptor conjugates comprising triarylamine donors and cyanopyridine or benzonitrile acceptors was readily synthesized via the bromine–lithium exchange–borylation–Suzuki (BLEBS) sequence in moderate to good yields. The modular synthetic approach enables systematic variation of conformational constraint as a molecular design element for tailoring excited‐state properties. Cyclic voltammetry reveals irreversible dimerization of triarylamine derivatives lacking *para*‐substituents. Photophysical studies in the solid state, solution, and polymethylmethacrylate (PMMA) matrix reveal photoluminescence quantum yields (PLQY) of up to 70%, emission bandwidths as narrow as 2480 cm^−1^ (47 nm), and CIE 1931 coordinates approaching the standard blue point. Phenylene extension increases conformational constraint around the acceptor, inducing hypsochromically shifted emission and providing an additional structural handle for tuning the optical properties. Pronounced positive emission solvatochromism, aggregation‐induced emission enhancement (AIEE), temperature‐dependent spectroscopy, and quantum‐chemical calculations consistently indicate that conformational modulation governs charge‐transfer interactions, excited‐state relaxation, and triplet‐state accessibility. Together, the experimental and theoretical results establish structure–property relationships that provide design principles for the rational engineering of future blue‐emissive organic chromophores.

## Graphical abstract

Highly blue emissive *ortho*‐triarylamine–acceptor (benzonitriles or cyanopyridines) conjugates are rapidly synthesized by bromine–lithium exchange borylation–Suzuki (BLEBS) sequence. Cyclic voltammetry reveals dimerization processes. Photophysical properties display stimuli‐responsive properties revealing them as promising emitters. Quantum chemical calculations render the conjugates potential hybridized localized and charge transfer (HLCT) dyes.

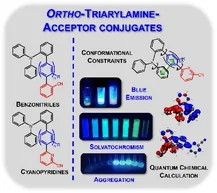

## 1 Introduction

Organic electronics have gained significant prominence in the modern display industry [1] and are a central focus of both scientific and industrial research efforts [2]. In particular, organic light‐emitting diodes (OLEDs) are highly attractive materials due to their low‐temperature processability [3], superior efficiency without backlight‐related losses [4], and intrinsic mechanical flexibility [5]. The chemical versatility of organic functional molecules not only benefits OLED technology but also extends to applications in organic photovoltaics [6] or biosensing technologies [7]. Despite these advantages, the development of highly efficient blue emitters remains a major challenge, primarily due to their limited operational stability arising from degradation pathways that shorten device lifetimes [8, 9]. To address this issue, hybridized local and charge transfer (HLCT) emitters have been explored, as they combine balanced locally excited (LE) and charge‐transfer (CT) characteristics within their excited states [10, 11]. HLCT emitters can mitigate the spin‐statistical limitations of conventional fluorescent emitters by integrating fast radiative decay processes [12] with triplet exciton utilization, thereby reducing efficiency roll of in the device [13]. Blue HLCT emitters are typically based on donor–acceptor (D–A) [14] or donor–π–bridge–acceptor (D–π–A) [15] systems, in which the electronic coupling between donor and acceptor units is finely tuned [16]. The incorporation of a π‐conjugated bridge enables conformational control over the dihedral angle between donor and acceptor moiety, thereby modulating the nature of the excited states and reducing the energy gap between LE and CT states to facilitate mixing states [17]. Triphenylamine (TPA) derivatives are widely employed as donor moieties in emitters [18] owing to their high thermal and photochemical stability [19], excellent hole transporting ability [20], and propeller‐like molecular structure, stabilizing the amorphous morphology of OLED layers [21]. The combination of a moderately strong donor character with pronounced steric hindrance promotes a small energy gap between LE and CT states, favoring the emergence of HLCT behavior [22]. Furthermore, the propeller‐shaped structure of TPA derivatives often leads to aggregation‐induced emission (AIE) properties [23], which arise from restricted intramolecular motions in the aggregated state [24]. On the acceptor side, benzonitrile derivatives are frequently utilized in D–A systems as HLCT emitters [25]. Benzonitriles containing multiple cyano groups tend to exhibit thermally activated delayed fluorescence (TADF) properties [16, 26], whereas mono‐cyano‐substituted derivatives display weaker electron‐withdrawing strength, resulting in reduced CT character [26]. This tunability of acceptor strength allows for modulation between HLCT and TADF mechanisms [16]. In addition, cyanopyridine‐based acceptor units have been investigated as blue emitters due to their enhanced electron‐withdrawing capability compared to benzonitrile analogs [27, 28].

Therefore, we focus on the efficient one‐pot syntheses and systematic investigation of electrochemical and photophysical properties of novel conformationally controlled D–A and D–π–A chromophores exhibiting blue emission. Here, molecular conformation is employed as a design parameter to tune the electronic coupling between donor and acceptor units (Figure 1). In contrast to linear *para*‐substituted D–A systems [29, 30], *ortho*‐substitution induces a pronounced conformational distortion between donor and acceptor units [31]. In particular, the substitution pattern around the D–A bridge can control their relative orientation and dihedral angle, thereby modulating conjugation, electronic coupling, and the resulting excited‐state properties [14, 30, 31]. The resulting spatial proximity may additionally allow for alternative CT pathways, such as through‐space charge transfer (TSCT), alongside conventional through‐bond coupling [32, 33, 34]. Overall, these structural effects demonstrate the potential of conformational control to modulate the electronic properties of D–A chromophores [35].

**FIGURE 1:**
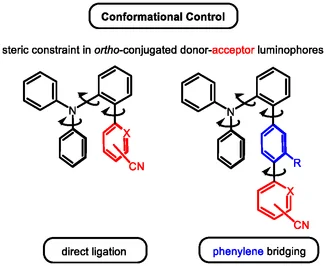
Conformational control in *ortho*‐conjugated donor–acceptor luminophores.

Herein, we report a series of novel cyanopyridine‐ and benzonitrile‐based TPA chromophores, functionalized in the sterically hindered *ortho*‐position at the donor moiety and readily accessible by a one‐pot synthetic sequence. Alongside comprehensive experimental characterization of the properties, the potential HLCT nature of these materials is further elucidated by time‐dependent density functional theory (TD‐DFT) calculations of their electronic structures.

## 2 Results and Discussion

### 2.1 Synthesis

Sequential multicomponent reactions represent an efficient strategy for the synthesis of structurally diverse compound libraries [36]. Recently, this approach has been successfully applied for the preparation of *meta*‐ and *ortho*‐biaryl‐substituted triarylamines [37]. The bromine–lithium exchange–borylation–Suzuki (BLEBS) sequence [38] has previously been employed with high efficiency for the synthesis of *para*‐substituted triarylamine–benzonitrile [29] and ‐pyridine [30] emitters. Based on our prior studies investigating extended π‐conjugation in *para*‐substituted triarylamine–benzonitrile conjugates [29], we became interested in elucidating the effects associated with substitution at the sterically hindered *ortho*‐position. To access such chromophores, the synthetic strategy has been adapted and initially applied to the synthesis of π‐extended donor precursors by a phenylene bridge (Scheme 1). Starting from 2‐bromo‐*N*,*N*‐diphenylaniline (**1**), bromo–lithium exchange followed by borylation affords the boronated species, directly utilized in a one‐pot fashion for Suzuki coupling with the according bromo‐iodo‐benzene **2**. Owing to the steric hindrance at the *ortho*‐position, sodium hydroxide has been employed as base. The resulting π‐extended donors **3** are obtained in good yields of 64%–68%.

**SCHEME 1:**
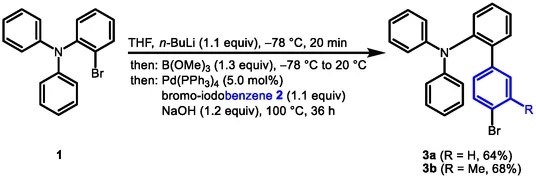
BLEBS synthesis of biaryl‐substituted triarylamine donor moieties **3**.

The successful application of the BLEBS sequence to the triarylamine donors **1** and **3** enables the synthesis of both directly linked and phenylene‐extended D–A conjugates. In this manner, six novel chromophores **6** and **7** incorporating cyanopyridine and benzonitrile acceptor moieties are obtained in moderate to good yields, ranging from 57% to 84% (Scheme 2). Compounds **6c** and **7c** feature additional steric hindrance in proximity to the acceptor unit [29, 30], resulting in highly conformationally constrained conjugates. This structural modification causes only a slight decrease in the overall yields.

**SCHEME 2:**
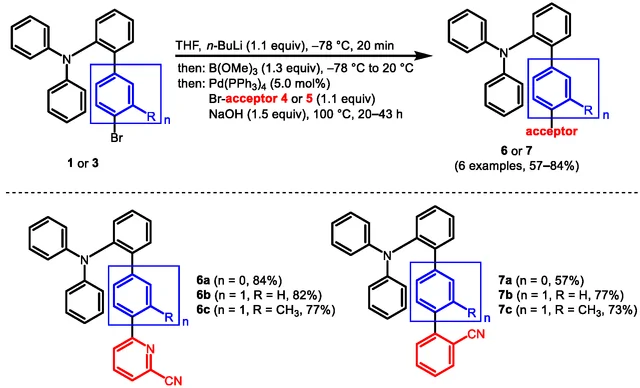
BLEBS synthesis towards the triarylamine–pyridine **6** and triarylamine–benzonitrile conjugates **7**.

All structures of title compounds **6** and **7** can be unambiguously assigned by ^1^H and ^13^C NMR and IR spectroscopy as well as mass spectrometry. In addition, the molecular composition of new compounds has been determined by combustion analysis (see Sections S2 and S3). Furthermore, compound **6b** has been selected for comprehensive structural elucidation by NMR spectroscopy owing to the presence of a characteristic TPA donor moiety and a more complex acceptor unit (see Section S3.1).

### 2.2 Electronic Properties

The electronic ground state of the investigated compounds has been probed by cyclic voltammetry. Compounds **6** and **7** were measured in dichloromethane solutions at room temperature, and all redox potentials were referenced to ferrocene/ferrocenium (Fc/Fc^+^) (*E*_0_^0/+1^ = 0.00 V) [39]. Detailed investigations are provided in the Supporting Information (see Sections S4.2 and S4.3). The cyclic voltammograms exhibit irreversible behavior, as evidenced by the appearance of two redox processes of different magnitudes upon the second measurement cycle (Figure S23). This behavior is consistent with previous reports on the oxidation of unsubstituted TPA, which undergoes irreversible dimerization of the initially formed radical cation to yield the corresponding benzidine [40]. The resulting *N*,*N*,*N′*,*N′*‐tetraaryl benzidine unit is redox‐active in its own right and can undergo two sequential oxidation processes [41]. The electronic properties of tetraaryl benzidine‐based dimers of cyanopyridine‐based conjugates **6a** and **6b** as monomers are separately investigated [42]. The dimerization behavior of *ortho*‐biaryl‐substituted triarylamines has been extensively studied previously, confirming the observed characteristic behavior of this substance class [37].

### 2.3 Photophysical Properties

All six chromophores **6** and **7** exhibit intense blue luminescence in the solid state, in solution, and embedded in a PMMA matrix, which is readily visible to the naked eye (Figure 2). Therefore, the electronic properties of the D–A conjugates were systematically investigated by UV/vis absorption and fluorescence spectroscopy.

**FIGURE 2:**
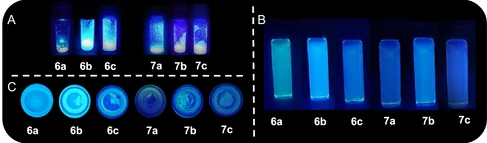
Emission after photoexcitation of compounds **6** and **7** recorded in the solid state (A), solution (toluene, B), and in a PMMA matrix (C) (*λ*_exc_ = 365 nm, *T* = 293 K, *c*(toluene) = 10^−6^ m, *c*(PMMA) = 1 wt%).

### 2.4 Solid‐State Emission

The emission maxima for the cyanopyridine‐based conjugates **6** appear in the range of 434–457 nm (Figure 3A and Table 1). In contrast, the benzonitrile‐based compounds **7** exhibit a more pronounced hypsochromic shift, with emission maxima between 408 and 435 nm, indicating a strong dependence of the emission behavior on the substitution pattern (Figure 3B and Table 1). Notably, the phenylene‐bridged derivatives featuring additional steric hindrance in proximity to the acceptor unit, namely, compounds **6c** and **7c**, display the most pronounced hypsochromic shift within their respective series (Figure 3). Overall, the emission bands are relatively narrow, with full width at half maximum (FWHM) values ranging from 2480 to 2880 cm^–1^ (see Table S4). Absolute photoluminescence quantum yields (PLQY) reach from 24% (**7b**) up to 70% (**6c**) and are generally higher for the cyanopyridine‐based conjugates **6**. Within each compound class, dyes **6c** (0.70) and **7c** (0.31) exhibit the highest PLQY values. In comparison to compound **8** [37] (*λ*_max_ _,em_ = 423 nm, PLQY = 0.16) [37], which features *para*‐substitution at the acceptor moiety (for structure see Table S4), the *ortho*‐substituted analog **7a** exhibits an increased PLQY of 29%. While this difference is consistent with a possible influence of the altered molecular conformation on the excited‐state relaxation pathways, a direct causal relationship between *ortho*‐substitution and the increased PLQY cannot be established from this comparison alone. Indeed, the PLQYs of 18%–29% for dyes **6a**–**c** and 16%–21% for dyes **7a**–**c** do not show a simple monotonic dependence on a single structural parameter, indicating that the observed photophysical properties result from the interplay of molecular conformation and excited‐state electronic structure.

**FIGURE 3:**
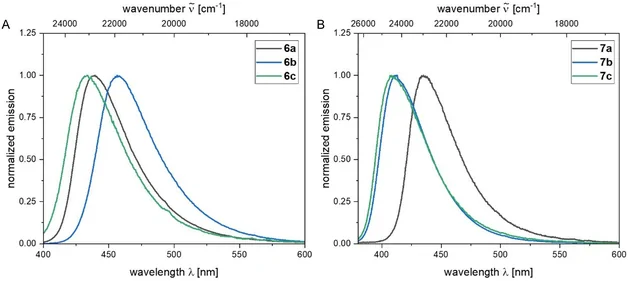
Normalized emission spectra for cyanopyridine‐based dye **6** (A) and benzonitrile‐based donor–acceptor conjugates **7** (B) of the directly linked compound (**6a**, **7a**: black), the π‐extended (**6b**, **7b**: blue) and the π‐extended with additional steric hindrance (**6c**, **7c**: green) recorded in the solid state (*T* = 293 K).

**TABLE 1:** Selected photophysical properties (emission maxima and photoluminescence quantum yields) of compounds **6** and **7** in the solid state.

Compound	*λ*_max,em_, nm (Φ_PL_)^a^	Compound	*λ*_max,em_, nm (Φ_PL_)^a^
**6a**	440 (0.47)	**7a**	435 (0.29)
**6b**	457 (0.31)	**7b**	413 (0.24)
**6c**	434 (0.70)	**7c**	408 (0.31)

a Emission in the solid state and absolute photoluminescence quantum yield Φ_PL_ determined with an integrating sphere (powder sample, *T* = 293 K).

Furthermore, the fluorescence lifetime *τ* (3.8*–*12.5 ns) (see Section S6), as well as experimentally obtained radiative (*k*_r_^exp^) and nonradiative (*k*_nr_^exp^) rate constants [43], are determined (Table S4). For all investigated conjugates, comparatively high radiative rate constants in the order of 10^7^ *–*10^8^ s^–1^ are observed. The cyanopyridine‐based conjugates **6** exhibit longer fluorescence lifetimes than the corresponding benzonitrile‐based compounds **7**. In conjunction with their higher PLQYs, this indicates a diminished nonradiative depopulation of the S_1_ state. Within each compound class, compounds **6c** and **7c** display the highest radiative rate constants *k*_r_^exp^. Notably, for compound **6c**, the radiative rate constant exceeds the nonradiative one, highlighting the particularly efficient emissive decay.

Overall, the pronounced hypsochromically shifted emission maxima, narrow emission bandwidths, and high radiative rate constants observed in the solid state render compounds **6** and **7** promising candidates for application as OLED emitters and warrant further investigation. The effects of the structural modifications on the solid‐state photophysical properties are not fully monotonic across the investigated series. The additional methyl group in derivatives **6c** and **7c** is expected to primarily influence the torsional geometry at the aryl–aryl junction, with the observed emission shift being more pronounced for the pair **6b**/**6c**. The corresponding changes in the emission spectra for dyes **7b**/**7c** are comparatively subtle. This difference suggests that the effect of steric substitution on solid‐state properties is not solely governed by conformational changes but may also depend on the nature of the acceptor unit or the molecular environment.

### 2.5 Emission in Solution

All chromophores exhibit fluorescence in toluene and ethyl acetate solutions upon excitation with UV light (for details, see Section S5.2), with emission colors ranging from blue to green (Figures 2 and 6). The observed variation in emission color already suggests a pronounced influence of solvent polarity on photophysical properties.

The absorption spectra in toluene display at least two distinct absorption maxima in the UV region, which markedly differ in their relative intensities (see Section S5.2). The absorption maxima at higher energy (291–297 nm) appear as intense bands with molar absorption coefficients *ε* ranging from 17,100 to 43,100 L mol^–1^ cm^–1^ (Table 2)_._ In contrast, the absorption maxima at lower energy are typically observed as shoulders (345–353 nm), exhibiting significantly reduced molar absorption coefficients *ε* (3200–4100 L mol^–1^ cm^–1^), indicative of a weaker electronic transition. For compound **6b,** as an exception, a maximum at lower energy is observed (Figure 4A). For cyanopyridine‐based conjugates **6,** this maximum or shoulder is generally more pronounced in the spectra than for the benzonitrile‐based conjugates **7** (Figure 4). Furthermore, the phenylene‐bridged chromophores bearing additional steric hindrance (**6c** and **7c**) exhibit a less distinct shoulder due to increased overlap with the absorption band at higher energy and in general the highest molar absorption coefficients in their respective series (Table 2). The emission spectra recorded in toluene exhibit a pronounced dependence on the substitution pattern. The emission maxima for the cyanopyridine‐based compounds **6** (442–447 nm) are bathochromically shifted relative to the corresponding benzonitrile‐based analogs **7** (424–441 nm), consistent with the trend observed in the solid state (Tables 1 and 2). As previously noted, compounds **6c** (442 nm) and **7c** (424 nm) display the most pronounced hypsochromic shifts within their respective series. While the additional methyl group has only a minor effect on the emission spectrum of **6b** (448 nm) compared to **6c** (442 nm), more pronounced changes are observed for the corresponding derivatives **7**. Notably, this trend is reversed in the solid state and is further discussed below. Compound **6a** exhibits an additional shoulder at 469 nm, indicative of overlapping electronic transitions, which directly influences the bandwidth. Accordingly, a relatively large FWHM value of 4740 cm^–1^ is observed. All compounds show substantial FWHM values (3240–4740 cm^−1^) and Stokes shifts ranging from 5200 to 7200 cm^–1^ (Table S5). The PLQYs in toluene are moderate (0.16–0.29) and, in contrast to the solid‐state measurements, do not exhibit a clear trend. Degassing of the solution has no significant effect on the PLQY of compound **6b**, whereas a slight increase is observed for compound **7b**, which could indicate triplet involvement in the emission [44]. Exemplified for compound **7b**, the energy gap Δ*E*_ST_ between the excited singlet and triplet state was estimated using the onsets of the fluorescence and phosphorescence spectra at 77 K [45], revealing a large gap of 0.48 eV (Figure S50). At low temperature for the fluorescence, two distinct maxima at 416 and 495 nm are observed presumably due to different conformers frozen in solution. For the longest wavelength emission band, a reduced Δ*E*_ST_ value of 0.20 eV can be estimated (Figure S51). To investigate possible contribution of different conformers in frozen solution, a rotational energy profile is calculated (for details see Section S5.4), identifying two minima of comparable energy. Further optimizations utilizing toluene as a solvent confirm that two stable rotamers differ in free energy by 3.8 kJ/mol. Boltzmann distribution [46] was predicted and an interconversion barrier calculated to be 7.9 kcal/mol, indicating at room temperature fast interconversion [47]. At 77 K, conformational freezing is likely with possible coexistence of both rotamers. Fluorescence lifetimes in toluene (4.5–10.5 ns) are notably shorter than those measured in the solid state (Tables S4 and S5). Within each compound class, dyes **6c** (6.9 ns) and **7c** (5.1 ns) exhibit the shortest lifetimes. Compound **6a** displays two distinct fluorescence lifetimes indicative of the involvement of different conformers. The radiative rate constants in toluene remain high (in the order of 10^7^ s^–1^), however, the nonradiative rate constants are even larger (in the order of 10^8^ s^–1^), accounting for the overall moderate PLQYs in toluene solutions. Degassing of the samples leads to prolonged fluorescence lifetimes, as exemplified by compounds **6b** and **7b**, accompanied by a decrease of *k*_r_^exp^ and *k*_nr_^exp^. In both cases, particularly for compound **7b**, the nonradiative decay is markedly suppressed, which is indicative of reduced quenching by molecular oxygen [44] (Table S5).

**TABLE 2:** Selected photophysical properties (absorption and emission maxima, photoluminescence quantum yields and Stokes shifts) of compounds **6** and **7** in solution (toluene and ethyl acetate).

Compound	*λ*_max,abs_, nm (*ε*/L mol^*–*1 ^ cm^−^^1^)^a^	*λ*_max,em_, nm (Φ_PL_)^b^	Δ$\bar{\nu }$, cm^−^^1^ c	*λ*_max,abs_, nm (*ε*/L mol^*–*1 ^ cm^*–*1^)^c^	*λ*_max,em_, nm (Φ_PL_)^b^		Δ$\bar{\nu }$, cm^−^^1^ c
In toluene				In ethyl acetate			
**6a**	291 (22,500), 351sh (3800)	457 (0.18) 469sh	7200	268sh (24,900), 287 (32,200), 350sh (5800)	452sh 485 (0.09)		8000
**6b**	295 (39,200), 353 (3900)	448 (0.29) (0.28)^d^	6000	291 (46,400), 349sh (4100)	474 (0.16)		7600
**6c**	293 (43,100), 347sh (4800)	442 (0.24)	6200	274sh (26,000), 289 (28,300), 345sh (2900)	463 (0.25)		7400
**7a**	294 (17,100), 349sh (3200)	434 (0.16)	5600	263sh (7700), 292 (18,500), 348sh (3100)	445 (0.15)		6300
**7b**	284sh (25,400), 296 (29,200), 345sh (3700)	441 (0.21) (0.28)^d^	6300	269 (41,300), 294 (43,900), 345sh (5500)	458 (0.16)		7200
**7c**	297 (29,900), 348sh (4100)	424 (0.19)	5200	261 (17,600), 295 (21,200), 343sh (2800)	435 (0.15)		6200

a Recorded in toluene or ethyl acetate, *T* = 293 K, *c*_abs_ = 10^*–*5^ m.

b Recorded in toluene or ethyl acetate, *T* = 293 K, *c*_em_ = 10^*–*7^ m, absolute quantum yields determined using an integrating sphere, *λ*_exc_ = *λ*_max__,__abs_.

c $\text{Δ}\tilde{\nu }=\left(\frac{1}{\lambda_{\max,\mathrm{abs}}}-\frac{1}{\lambda_{\max,\mathrm{em}}}\right)\cdot 10^{7}$ (*λ*_max_ in nm) [cm^−1^].

d Sample degassed with nitrogen for 1 h prior to measurement.

**FIGURE 4:**
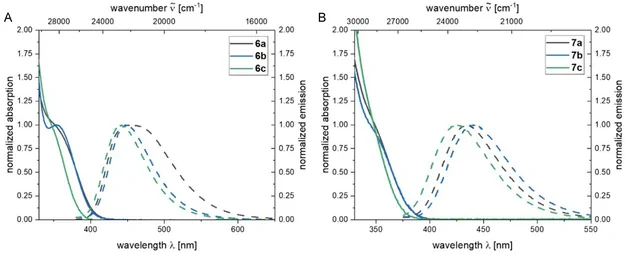
Normalized UV/vis absorption (bold lines) and emission (dashed lines) spectra for cyanopyridine‐based **6** (A) and benzonitrile‐based donor–acceptor conjugates **7** (B) of the directly linked compound (**6a**, **7a**: black), the π‐extended (**6b**, **7b**: blue) and the π‐extended with additional steric hindrance (**6c**, **7c**: green) recorded in toluene (*T* = 293 K, *c*_abs_ = 10^–5^ m, *c*_em_ = 10^–6^ m, *λ*_exc_ = *λ*_max,abs_).

Notably, the sterically demanding derivatives **6c** and **7c** exhibit the most hypsochromically shifted emission within their respective series in solution and particularly in the solid state (Tables 1 and 2). This observation suggests that steric constraint can influence the D–A conformation and, consequently, the electronic coupling as well as emission properties. However, the emission trends are not identical in solution and in the solid state. The wavelength of emission maxima do not follow a simple monotonic relationship with the degree of steric constraint. For example, conjugates **6a**–**c** display a progressive hypsochromic shift in toluene (457, 448, and 442 nm), whereas the solid‐state emission of dye **6b** is bathochromically shifted relative to that of dye **6a** (440, 457, and 434 nm). Similarly, dyes **7a**–**c** exhibit a non‐monotonic trend in toluene (434, 441, and 424 nm), while a pronounced hypsochromic shift is observed in the solid state (435, 413, and 408 nm). These differences indicate that the photophysical response cannot be attributed solely to steric substitution or increased π‐conjugation but rather results from the interplay of molecular conformation, excited‐state electronic structure, and the surrounding environment. In solution, conformational freedom and solvation can influence the accessible geometries and relaxation pathways, whereas in the solid state, molecular packing and intermolecular interactions additionally constrain the molecular conformation. These conformational changes are reflected in the calculated dihedral angles, providing a structural perspective on the observed differences in photophysical properties. The relationship between the dihedral angles, their changes upon excitation, and the corresponding emission properties is discussed in detail in the following sections. These results indicate that the structural modifications affect the molecular conformation, although the resulting changes in the photophysical properties cannot be attributed to conformational changes alone.

Similar to observations in toluene, conjugates **6** and **7** exhibit in ethyl acetate an absorption maximum at higher energies (287–295 nm), characterized by increased intensity and molar absorption coefficients *ε* in the range of 18,500–46,400 L mol^–1^ cm^–1^ (see Section S5.2 and Table 2)_._ Additionally, a shoulder (343–350 nm) at lower energy is present, displaying significantly reduced molar absorption coefficients *ε* (2800–5800 L mol^–1^ cm^–1^) (Figure 5 and Table 2). Furthermore, in ethyl acetate, for most derivatives, a third absorption band (261–274 nm) at high energy is observed, with molar absorption coefficients *ε* ranging of 7700–41,300 L mol^–1^ cm^–1^ (Table 2). Owing to the extended spectral window, this feature is observed and appears predominantly as a shoulder, except for compounds **7b** and **7c**, where it is clearly resolved as a distinct maximum (see Section S5.2). In the more polar solvent ethyl acetate, all absorption bands are slightly hypsochromically shifted, and the shoulder at lower energy is less pronounced compared to toluene.

**FIGURE 5:**
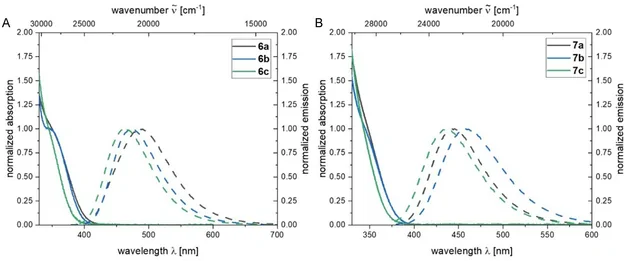
Normalized UV/vis absorption (bold lines) and emission (dashed lines) spectra for cyanopyridine‐based **6** (A) and benzonitrile‐based donor–acceptor conjugates **7** (B) of the directly linked compound (**6a**, **7a**: black), the π‐extended (**6b**, **7b**: blue) and the π‐extended with additional steric hindrance (**6c**, **7c**: green) recorded in ethyl acetate (*T* = 293 K, *c*_abs_ = 10^–5^ m, *c*_em_ = 10^–6^ m, *λ*_exc_ = *λ*_max,abs_).

The emission maxima in ethyl acetate are bathochromically shifted for all conjugates **6** (452–485 nm) and **7** (435–458 nm), indicating positive emission solvatochromism [48] and, consequently, a polar character of the first excited state [49] (Table 2). As previously noted, compounds **6c** (463 nm) and **7c** (435 nm) exhibit the most pronounced hypsochromic shifts within their respective series. Compound **6a** shows a less distinct emission shoulder at 452 nm alongside a maximum at 485 nm, resulting in a reduced bandwidth of 4250 cm^–1^, suggesting that one electronic transition becomes less favored with increasing solvent polarity. For the remaining compounds, the FWHM values (3340–4250 cm^–1^) and all Stokes shifts (6200–8000 cm^–1^) increase with increasing solvent polarity (Table S5). The PLQYs decrease in different amounts for most compounds in ethyl acetate (0.09–0.25), while the fluorescence lifetimes (6.0–8.7 ns) are prolonged. Compound **6a** exhibits a single lifetime component in ethyl acetate, consistent with the diminished shoulder observed in its emission spectrum. Due to the often reduced PLQYs and extended lifetimes, both radiative and nonradiative rate constants slightly decrease or remain within the same order of magnitude as in the less‐polar solvent toluene (Table S5). This behavior is typical for the stabilization of an intramolecular charge transfer (ICT) type of excited state in the polar solvent [50]. The PLQY thereby decreases by additional nonradiative deactivation channels, leading to conical intersections [51].

As demonstrated previously, in particular, the emission behavior of the investigated conjugates is strongly dependent on the solvent polarity, albeit to varying extents (Table 2). Consequently, compound **6b** was selected for a detailed investigation of solvent polarity effects. As observed by the naked eye, the emission color spans from deep blue in cyclohexane to green in acetonitrile, whereas the emission in ethanol appears comparatively weak (Figure 6).

**FIGURE 6:**
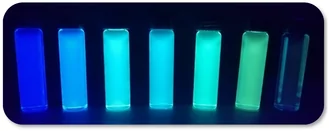
Emission solvatochromism of compound **6b** recorded in different solvents (from left to right: cyclohexane, toluene, ethyl acetate, THF, dichloromethane, acetonitrile, and ethanol *λ*_exc_ = 365 nm, *T* = 293 K, *c* = 10^−6^ m).

For quantification of the solvatochromism behavior, the absorption and emission spectra of compound **6b** were recorded in six different solvents of variable polarity (Figure 7).

**FIGURE 7:**
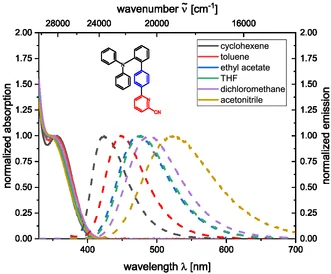
UV/vis absorption and emission spectra of the cyanopyridine‐based conjugate **6b** in different solvents: cyclohexane (black), toluene (red), ethyl acetate (blue), THF (green), dichloromethane (purple), and acetonitrile (gold). Recorded in different solvents (absorption spectra: bold lines, *T* = 293 K, *c*_abs_ = 10^−5^ m and emission spectra: dashed lines, *T* = 293 K, *c*_em_ = 10^−6^ m).

The absorption maximum at lower energy is, in general, hypsochromically shifted (345–357 nm, Table 3), albeit to a comparatively small extent with increasing solvent polarity, indicating low sensitivity toward solvent effects. The distinct maximum at lower energy in nonpolar solvents, such as cyclohexane and toluene, progressively overlaps with the band at higher energy, resulting in the appearance of a shoulder (Figure 7). In contrast, the emission behavior exhibits a pronounced dependence on solvent polarity, as evidenced by a bathochromic shift of the emission maxima from 423 to 525 nm with increasing polarity from cyclohexane to acetonitrile, reflecting a significantly higher dipolar character of the excited state relative to the ground state [52] (Table 3). A plot of the emission maxima against Reichardt's [53] *E*_*T*_(30) solvent polarity scale reveals a reasonable linear correlation (*r* ^2^ = 0.97) (see Section S5.5 and Figure S66), supporting positive emission solvatochromism [54]. Lippert–Mataga analysis is employed to quantify the dipolar character of the excited state by evaluating the solvent‐dependent change in dipole moment [55, 56, 57, 58, 59, 60]. Assuming a spherical dipole model, the Onsager radius for the investigated solvents is determined by TD‐DFT calculations based on the optimized ground‐state structure in the gas phase (see Section S7.7). From the linear correlation (*r* ^2^ = 0.94) between the Stokes shifts Δ$\tilde{\nu }$ and orientation polarizability Δ*f*, a change of dipole moment Δ*μ* of 17.9 Debye can be estimated (see Section S5.5 and Figure S67). For comparison, Δ*μ* can be additionally estimated utilizing the molecule radius *a* derived from bond lengths. Due to the twisted molecular conformation, the terphenyl unit is selected as a representative structural motif for this purpose (see Figure S68). This procedure results in an estimated molecular radius of 5.67 Å and a corresponding dipole moment change of 16.5 D (*r* ^2^ = 0.97), in the same order as the value obtained from the calculated Onsager radius (Figure S67). The linear dependency indicates a substantial dipolar character of the excited state, in agreement with the experimentally observed solvatochromism and TD‐DFT calculations (vide infra). The PLQY remains comparatively constant with increasing solvent polarity, reaching a maximum of 29% in toluene and up to 23% in dichloromethane. In high‐polar solvent acetonitrile, however, the PLQY drops markedly to 12% (Table 3). Additionally, fluorescence lifetimes are generally prolonged by the increased solvent polarity (4.6–12.7 ns), while both *k*_r_^exp^ and *k*_nr_^exp^ decrease, which can be rationalized by stabilization of an ICT state, as previously discussed (Table S8) [45]. In acetonitrile, the pronounced decrease in PLQY accompanied by enhanced nonradiative decay is consistent with increased conformational relaxation and a more twisted ICT (TICT) character [61], leading to reduced *k*_*r*_^exp^ rates and increased nonradiative relaxation pathways [62]. Furthermore, the observed increase in Stokes shifts and FWHM values is consistent with stabilization of the ICT state in more polar environments (Table S8) [63, 64].

**TABLE 3:** Selected photophysical properties (absorption and emission maxima, photoluminescence quantum yields and Stokes shifts) of conjugate **6b** recorded in different solvents.

Solvent	*λ*_max,abs_, nm (*ε*/L mol^*–*1^ cm^*–*1^)^a^	*λ*_max,em_, nm (Φ_PL_)^b^	Δ$\tilde{\nu }$, cm^−^^1^ c	Solvent	*λ*_max,abs_, nm (*ε*/L mol^*–*1^ cm^*–*1^)^a^	*λ*_max,em_, nm (Φ_PL_)^b^	Δ$\tilde{\nu }$, cm^−^^1^ c
Cyclohexane	236sh (23,800), 294 (57,400), 357 (4900)	423 (0.20)	4400	THF	281sh (40,300), 292 (62,700), 345sh (6200)	478 (0.20)	8100
Toluene	295 (39,200), 353 (3900)	448 (0.29)	6000	dichloromethane	293 (58,900), 349sh (4900)	490 (0.23)	8200
Ethyl acetate	291 (46,400), 349sh (4100)	476 (0.16)	7600	Acetonitrile	233sh (28,500), 289 (65,300), 346sh (5900)	525 (0.12)	9900

a Recorded in different solvents, *T* = 293 K, *c*_abs_ = 10^*–*5^ m.

b Recorded in different solvents, *T* = 293 K, *c*_em_ = 10^*–*7^ m, absolute quantum yields determined using an integrating sphere, *λ*_exc _= *λ*_max,_ _abs_.

c $\text{Δ}\tilde{\nu }=\left(\frac{1}{\lambda_{\max,\mathrm{abs}}}-\frac{1}{\lambda_{\max,\mathrm{em}}}\right)\cdot 10^{7}$ ($\lambda_{\max}$ in nm) [cm^−1^].

### 2.6 Emission Upon Induced Aggregation

As previously observed, compound **6b** exhibits comparatively weak emission in ethanol (Figure 6). Owing to the propeller‐like structure of the TPA donor unit, pronounced luminescence in the aggregated state can be anticipated [24]. Consequently, compound **6b** was investigated with respect to AIE in ethanol/water mixtures with water fractions of up to 95%. A distinct blue emission becomes visible to the naked eye at a water content of approximately 60%, and blue emission lights up, attributable to aggregation at increased water content (Figure 8A). Analysis of the emission spectra as a function of water fraction allows quantification of the AIE behavior (Figure 8). In pure ethanol solutions with up to 60% water content, the emission intensity remains low. At a water fraction of 60%, an emission band emerges with a maximum at 448 nm, which is hypsochromically shifted relative to that in pure ethanol (497 nm, Figure 8B). The PLQY increases markedly from 1% to 37% (Figure 8C). At water fractions above 80%, the emission band undergoes a bathochromic shift to a maximum at 466 nm, accompanied by a decrease in emission intensity. This behavior may reflect changes in the aggregation state, potentially associated with the formation of less emissive or amorphous aggregates [65]. However, the precise nature and morphology of the aggregates were not further characterized in the present study.

**FIGURE 8:**
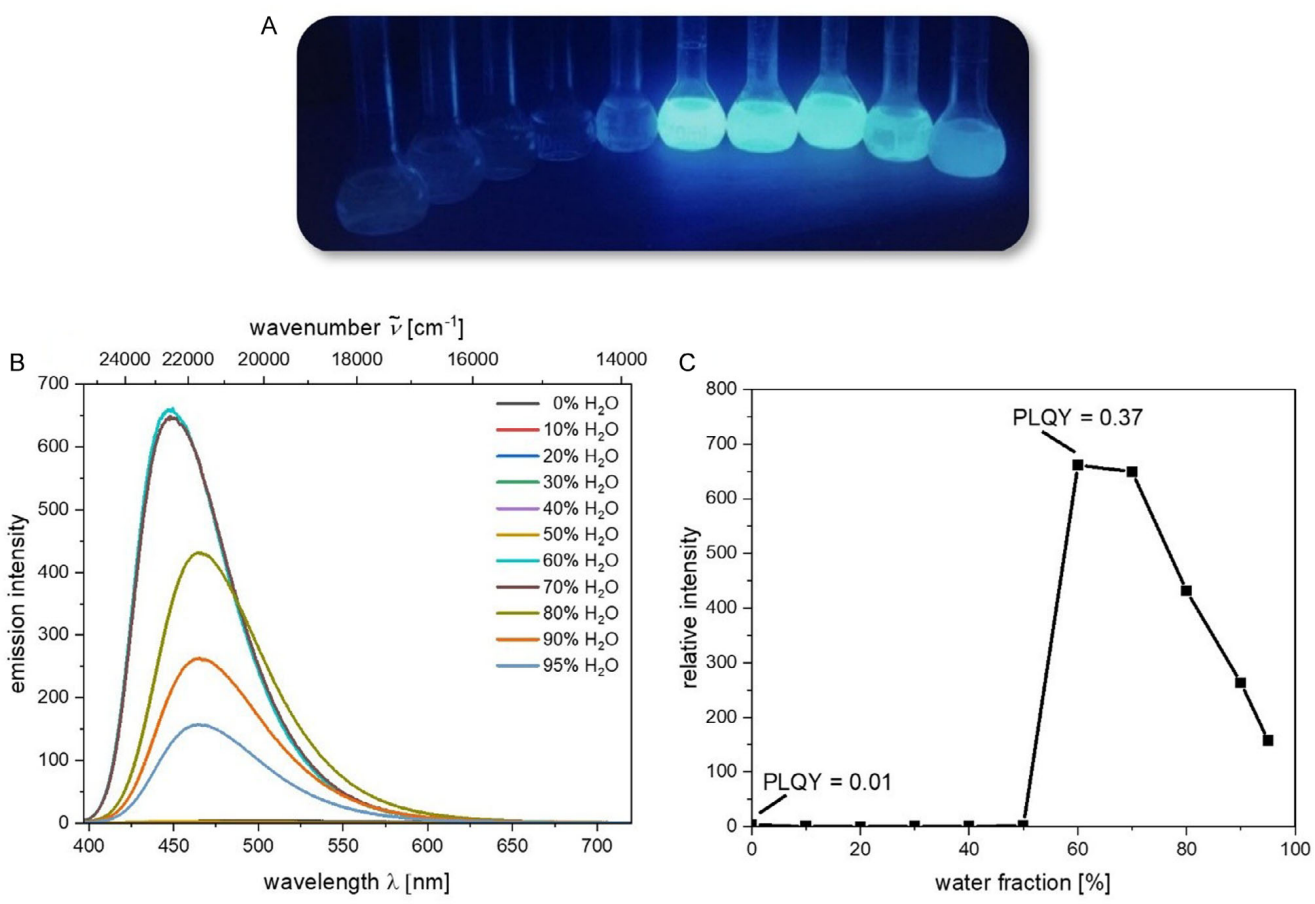
Qualitative AIE study of compound **6b** recorded in different ethanol/water mixtures (water fraction from left to right: 10%, 20%, 30%, 40%, 50%, 60%, 70%, 80%, 90%, 95%, *λ*_exc_ = 365 nm, *T* = 293 K, *c* = 10^−4^ m) (A), emission spectra for compound **6b** in different ethanol/water mixtures (*T* = 293 K, *c*_em_ = 10^−6^ m, *λ*_exc_ = *λ*_max,em_) (B), and the change in emission intensity as a function of the water fraction (C).

To further assess the generality of the observed AIE behavior and its dependence on molecular structure, additional derivatives **6a** and **7b** were investigated (see Section S5.6). Compound **6b** is selected as a representative example due to its pronounced AIE response and because it contains a phenylene bridge characteristic of the investigated molecular design. Compound **6a**, lacking the phenylene bridge, also exhibited AIE behavior, although the maximum emission intensity is observed at a higher water content of 80%, compared to 60% for dye **6b** (Figure S69). This indicates that the phenylene bridge is not essential for AIE but influences the aggregation conditions. Furthermore, compound **7b** bearing a benzonitrile acceptor is investigated to evaluate the influence of the acceptor type. In this case, the emission behavior differs from that of compound **6b**. Up to a water content of 40%, compound **7b** exhibits detectable emission intensity with the maximum shifting bathochromically from 480 to 510 nm, presumably due to positive emission solvatochromism (Figure S70). Above 50% water content, aggregation was accompanied by a pronounced increase in emission intensity and a hypsochromic shift of the emission maximum to 410 nm. The emission intensity reached a maximum at 60% water fraction, followed by a decrease at 70%. At 80% water content, a renewed increase in emission intensity accompanied by a bathochromic shift to 444 nm was observed, which may indicate a change in the aggregation mode at high water contents. These additional studies demonstrate that AIE or AIEE behavior is not restricted to compound **6b** while also highlighting the influence of the bridge and acceptor unit on the aggregation and emission behavior. The AIE studies were performed using ethanol/water mixtures; other solvent combinations were not systematically examined in the present study.

### 2.7 Emission in PMMA Matrix

For a detailed evaluation of their suitability as OLED emitters, all conjugates were embedded in a PMMA matrix, where they exhibited blue emission (Figure 2). The emission properties are strongly influenced by the rigid matrix environment (Figure 9). For the cyanopyridine‐based conjugates **6**, the emission maxima are observed in the range from 424 to 444 nm, whereas the benzonitrile‐based conjugates **7** show maxima between 408 and 428 nm (Table 4). The previously observed general trend for the π‐extended conjugates bearing additional steric hindrance adjacent to the acceptor unit, compound **6c** (424 nm) and **7c** (408 nm), display the most pronounced hypsochromic shift of the emission maxima. Compared to their solid‐state emission behavior, the spectra in the ridged matrix exhibit broader bandwidths (3300–3860 cm^−1^), while the PLQYs are similar or decreased for most conjugates (0.19–0.45) (Table S6). In contrast, compound **7c** shows an increased PLQY in the matrix, which can be attributed to the suppression of nonradiative decay pathways [66]. To further elucidate this behavior, fluorescence lifetimes are investigated. In the PMMA matrix, the decay profiles could be fitted with a biexponential function, indicating the presence of an additional decay pathway that may account for the reduced PLQY observed for most conjugates. Two distinct lifetime components are identified: a shorter component (3.5–7.5 ns) and a longer component (7.6–14.9 ns), presumably due to the involvement of different conformers (Table S6). To assess their applicability as OLED emitters, the CIE coordinates of the conjugates in the PMMA matrix are determined. The obtained values (0.154–0.166, 0.053–0.109), particularly for compounds **6c**, **7a,** and **7b**, are in good agreement with the standard blue point (0.15, 0.06) for electroluminescene (EL) recommended by the European Broadcast Union (EBU) [67], thereby identified as promising candidates (Table 4).

**FIGURE 9:**
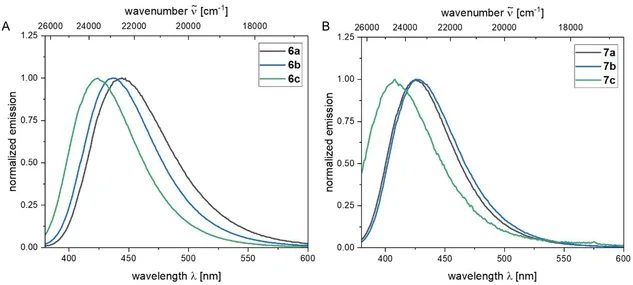
Normalized emission spectra for cyanopyridine‐based **6** (A) and benzonitrile‐based donor–acceptor conjugates **7** (B) of the directly linked compound (**6a**, **7a**: black), the π‐extended (**6b**, **7b**: blue) and the π‐extended with additional steric hindrance (**6c**, **7c**: green) recorded in a PMMA matrix (*T* = 293 K, *c*_comp_. = 1 wt%).

**TABLE 4:** Selected photophysical properties (emission maxima, photoluminescence quantum yields and CIE 1931 coordinates) of compounds **6** and **7** in a PMMA matrix (*T* = 293 K, *c*_comp._ = 1 wt%).

Compound	*λ*_max,em_, nm (Φ_PL_)^a^	CIE 1931 (*x*, *y*)	Compound	*λ*_max,em_, nm (Φ_PL_)^a^	CIE 1931 (*x*, *y*)
**6a**	444 (0.26)	0.155, 0.109	**7a**	426 (0.19)	0.156, 0.056
**6b**	438 (0.33)	0.154, 0.078	**7b**	428 (0.27)	0.155, 0.057
**6c**	424 (0.29)	0.156, 0.053	**7c**	408 (0.45)	0.166, 0.064

a Emission maxima and absolute photoluminescence quantum yield Φ_PL_ determined with an integrating sphere (matrix sample, *T* = 293 K).

For deeper insight into the emission processes, the temperature dependence of the photoluminescence behavior of compound **7b** was investigated (Figure 10). In toluene, the compound already exhibits sensitivity to degassing, indicating the involvement of oxygen‐dependent deactivation pathways [44]. Upon cooling from 300 to 80 K, the emission maximum undergoes a slight hypsochromic shift from 428 to 423 nm. Concomitantly, the emission intensity initially increases at 260 and 220 K, followed by a gradual decrease upon further cooling to 80 K (see Section S5.3 and Table S7). This non‐monotonic behavior can be attributed to the temperature‐dependent interplay of competing relaxation pathways. Initial cooling enhances emission due to restricted molecular motion and suppression of nonradiative decay channels [68]. At lower temperature (<220 K), however, conformational dynamics become effectively frozen, leading to the stabilization of weakly emissive or nonemissive excited states such as ICT or triplet states, which results in a reduction of overall emission intensity [69]. Notably, beginning at approximately 220 K, an additional bathochromically shifted shoulder emerges at around 500 nm and increases in intensity with decreasing temperature (Figure 10). This feature indicates the growing contribution of a second emissive state, which may originate either from the population of lower energy excited states like triplet states [70] or from the emission of a second conformer, similar to the behavior observed in frozen toluene solution (Figure S50). Time‐gated measurements further confirm the involvement of long‐lived emissive species. At 80 K, two emissive components with lifetimes exceeding 1 μs are detected. One component overlaps with the steady‐state fluorescence in the photoluminescence spectra, presumably assigned as delayed fluorescence originating from one conformer, while the second component could be attributed to phosphorescence (see Section S5.3 and Figure S61). The increasing contribution of these long‐lived processes accounts for the apparent decrease in emission intensity (Figure 10). A more detailed analysis of the gated emission reveals two distinct decay pathways, one in the range of 1–1000 μs and an additional long‐lived component in the range of 1000–10,000 μs (see Section S5.3 and Figure S62), indicating the coexistence of multiple emissive pathways, most likely arising from overlapping delayed fluorescence and phosphorescence contributions associated with different conformers. Evaluation of the Δ*E* *_ST_* value in the matrix yields a gap of 0.44 eV for the higher‐energy emission band, comparable to the value determined in solution (0.49 eV) (Figure S63). Consequently, it is reasonable to assume similar behavior for the less pronounced bathochromically shifted shoulder in the matrix, with a similarly reduced Δ*E*_*ST*_ value, possibly resulting in delayed fluorescence for that conformer. Rotamer analysis in toluene predicts two stable rotamers presumably frozen in solution at 77 K (see Section S5.4) with likely similar behavior in a frozen PMMA matrix. The fluorescence lifetime remains largely unaffected by decreasing temperature (see Section S6.3 and Figure S82), exhibiting two distinct lifetimes of 5.9 and 12.4 ns (*χ* ^2^ = 1.17). However, at 80 K, phosphorescence is observable with a maximum at 512 nm (see Figure S61), exhibiting a long lifetime of 1.3 s (*χ* ^2^ = 1.19) (see Section S6.3 and Figure S83), which supports the assignment of triplet state involvement at low temperatures.

**FIGURE 10:**
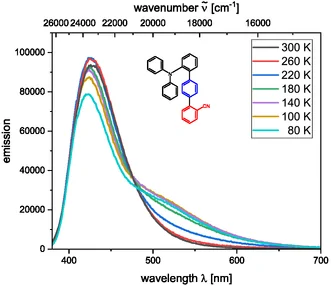
Relative emission spectra depending on the measurement temperature (300 K (black), 260 K (red), 220 K (blue), 180 K (green), 140 K (purple), 100 K (gold) and 80 K (turquoise)) of compound **7b** recorded in a PMMA matrix (*T* = 300–80 K, *c*_comp_. = 1 wt%).

### 2.8 Calculated Electronic Structure in Solution

For deeper insights into the electronic structures of the D–A conjugates and rationalizing the experimentally determined photophysical data in toluene, TD‐DFT calculations were performed using Gaussian 16 [71]. After a first screening of typically used long‐range corrected hybrid density functionals for D–A conjugates: cam‐B3LYP [72], ωB97X‐D [73, 74], and LC‐ωHPBE [75, 76], the best fit with the experimental data is obtained using cam‐B3LYP [72] as functional and Pople's 6‐311+G* [77] basis set (see Section S7.9 and Table S11). All minimum energy conformations were confirmed by analytical frequency analysis (NImag = 0). Consistent with the spectroscopic properties, the calculations were carried out using the polarizable continuum model (PCM) with toluene as the dielectric medium. For all conjugates, the calculated absorption bands are hypsochromically shifted compared to the experimental data (deviations of 0.24–0.50 eV for dye **6** and 0.48–0.56 eV for **7**) (Table 5), but the absorption shape and intensity are in good agreement (see Section S7.9 and Figure S84A). According to TD‐DFT calculations using the cam‐B3LYP functional, the absorption bands at higher energy consist of several transitions, merging into one broad band (see Table S12). Usually, highest occupied molecular orbital (HOMO) to lowest unoccupied molecular orbital (LUMO)+X or HOMO−1 to LUMO+X transitions are involved, indicating a combination of mainly locally excited (LE) state of the TPA donor moiety, HLCT character associated with the acceptor unit and bridging segment, as well as CT transitions between the donor and acceptor moieties, as revealed by frontier molecular orbital analysis (see Section S7.11). This mixed excited‐state nature results in moderate oscillator strengths of 0.1701–0.6804, depending on the respective excited‐state character (Table S12). The longest wavelength absorption is mainly attributed to HOMO to LUMO+1 or HOMO to transitions with low oscillator strengths of 0.0290–0.2074, which is in good agreement with the observed low molar absorption coefficients, typical for CT character [79] (Table 5). The electronic transitions are predominantly characterized by a dominant natural transition orbital (NTO) pair with a contribution exceeding 91%, with the sole exception of compound **6b**, which displays an additional pair with a negligible contribution (Table S15).To evaluate the electron–hole separation of the first excited state (longest wavelength absorption), the Δ*r* index (average hole–electron distance) [80] and the *S* *_r_* index (overlap degree of hole and electron) [81] are used, whereas large Δ*r* and low *S* *_r_* values indicate pronounced charge transfer (CT) character, although no strict thresholds exist [80, 81]. For both compound classes **6** and **7,** photoexcitation induces a pronounced CT from the triarylamine moiety to the acceptor unit or the phenylene‐bridged acceptor unit, as indicated by charge density difference (CDD) plots with high Δ*r* values up to 3.09 Å (Figure S85 and Table 5). The introduction of a phenylene bridge and the additional steric hindrance close to the acceptor moiety leads to decreased CT character due to reduced Δ*r* and increased *S* *_r_* values (Table 5). The CDD plots and in‐depth analysis of the frontier molecular orbitals and the percentage orbital contribution indicate an HLCT character since the electron density is overlapping mainly on the phenyl ring proximal to the acceptor unit or the phenylene bridge (see Figure S85 and Section S7.11). The orbital contribution depends on the ligation behavior. The directly linked conjugates **6a** and **7a** display a narrow orbital contribution, while the phenylene bridging causes a broader orbital contribution of up to four orbitals (Section S7.11). For compound **6a,** the calculations reveal two distinct transitions from the ground state to S_1_ and S_2_ states overlapping in the experimentally observed longest wavelength absorption band, possessing predominantly HLCT and CT character (Figure S87). The energy difference of the orbitals involved reveal for compound **6b** the lowest value which is in good agreement with the most bathochromically shifted absorption band (Figure S96A). Due to substantial deviations of up to 0.56 eV observed for the calculated longest wavelength absorption maxima, an additional study employing hybrid density functionals with varying fractions of Hartree–Fock (HF) exchange, specifically, the Boese‐Martin for kinetics (BMK) functional [82] (42% HF exchange) and M06‐2X [83] (54% HF exchange) was performed (Table S6). Among the tested functionals, BMK provides the best overall agreement with the experimental data (see Figure S84), reducing the deviations to 0.06–0.15 eV for conjugates **6** and 0.09–0.19 eV for **7** (Table 5). Compared to cam‐B3LYP, the BMK functional predicts a more pronounced CT character for the first excited state, as reflected by larger Δ*r* values of up to 3.75 Å and reduced *S* *_r_* values to 0.24 (Table 5). As illustrated by the CDD plots exemplified for conjugate **6**, an increased accumulation of electron density on the acceptor unit is predicted (Figure S85). The improved agreement obtained with BMK can be rationalized by the different treatment of HF exchange. BMK employes a constant HF‐exchange contribution of 42%, while cam‐B3LYP utilizes a range‐dependent scheme in which the HF‐exchange fraction increases with interelectronic distance up to 65%. The larger long‐range HF contribution can lead to increased HOMO–LUMO gaps and therefore presumably overestimate the excitation energies compared to the experiment. Since the BMK‐ and cam‐B3LYP‐optimized geometries exhibit only minor differences in bond lengths and dihedral angles, the improved agreement of BMK is attributed primarily to differences in the electronic‐structure description rather than to substantial changes in molecular conformation (Table S14).

**TABLE 5:** TD‐DFT calculations on the longest wavelength absorption maxima (Gaussian 16, cam‐B3LYP and BMK/6‐311+G*, PCM toluene) of compound **6** and **7** in comparison with the experimental data.

Compound	*λ*_max,abs,exp_, nm (eV) (*ε*/L mol^*–*1^ cm^*–*1^)^a^	*λ*_max,abs,calcd_, nm (eV), (oscillator strength) most dominant contribution	Δ*r*, Å^b^	*S* *_r_* b	
cam‐B3LYP					
** 6a**	351sh (3.53) (3800)	315 (0.0840) HOMO → LUMO (72%) 329 (3.77) (0.0290) HOMO → LUMO+1 (69%)	3.09	0.30	
** 6b**	353 (3.51) (3900)	311 (3.99) (0.2074) HOMO → LUMO+1 (55%)	2.66	0.51	
** 6c**	347sh (3.57) (4800)	305 (4.07) (0.1868) HOMO → LUMO+1 (56%)	2.36	0.46	
** 7a**	349sh (3.55) (3200)	304 (4.08) (0.0295) HOMO → LUMO (84%)	3.00	0.35	
** 7b**	345sh (3.59) (3700)	305 (4.07) (0.1853) HOMO → LUMO (44%) HOMO → LUMO+1 (29%)	2.61	0.43	
** 7c**	348sh (3.56) (4100)	301 (4.12) (0.1715) HOMO → LUMO+1 (54%)	2.34	0.45	
BMK					
** 6a**	351sh (3.53) (3800)	346 (0.0783) HOMO → LUMO+1 (67%) 357 (3.47) (0.0041) HOMO → LUMO (69%)	3.48	0.24	
** 6b**	353 (3.51) (3900)	343 (3.61) (0.1152) HOMO → LUMO (43%) HOMO → LUMO+1 (44%)	3.75	0.38	
** 6c**	347sh (3.57) (4800)	333 (3.72) (0.1191) HOMO → LUMO+1 (74%)	3.21	0.42	
** 7a**	349sh (3.55) (3200)	335 (3.70) (0.0148) HOMO → LUMO (94%)	3.34	0.29	
** 7b**	345sh (3.59) (3700)	337 (3.68) (0.1143) HOMO → LUMO (72%)	3.56	0.38	
** 7c**	348sh (3.56) (4100)	331 (3.75) (0.1185) HOMO → LUMO (55%) HOMO → LUMO+1 (32%)	3.01	0.42	

a Recorded in toluene, *T* = 293 K, *c*_abs_ = 10^*–*5^ m.

b Determined using Multiwfn [78].

Although cam‐B3LYP functional was employed consistently for the systematic description of absorption and emission properties, BMK was additionally employed for the absorption transitions due to improved agreement with the experimental absorption maxima (Table S13). Accordingly, detailed NTO analysis of the lowest‐energy absorption transitions and additional hole–electron analysis of sterically hindered conjugates **6c** and **7c** were conducted based on BMK results to further elucidate the electronic nature of the lowest excited states (see Section S7.10). Based on calculations employing the BMK functional, the lowest‐energy absorption transitions of conjugates **6** and **7** can each be described by a single dominant NTO pair, accounting for more than 96% of the corresponding transition density (Figure 11A,B). In both conjugate classes, the hole is predominantly localized on the triarylamine donor moiety, whereas the particle is mainly distributed over the biphenyl or terphenyl unit, extending toward and including the acceptor moiety. This spatial distribution indicates a substantial overlap between the corresponding electron densities. In depth hole–electron distribution and interfragment CT (IFCT) analysis were performed for the sterically hindered compounds **6c** and **7c,** further supporting the mixed character (Table S16 and Section S7.10). The hole is strongly localized for both compounds on fragment F1 (93.5%) including the TPA donor, whereas the excited electron is distributed over fragments F1–F3, with substantial populations on F2 (phenylene bridge) and F3 (acceptor moiety) (Figure 11C,D). Pronounced hole–electron overlap can be observed on F1 (60.9% and 63.9%) due to the phenyl ring connecting the bridge (Figure 11 and Table S16). Consistently, IFCT analysis reveals significant CT contributions of 50.5% and 46.8% for dyes **6c** and **7c**, respectively, alongside LE contributions of 37.6% and 41.3%, respectively. The dominant transfer pathways for dyes **6c** and **7c** are F1 → F2 (0.306/0.300 e) and F1 → F3 (0.181/0.151 e). Collectively, these results demonstrate that excited states of both compounds exhibit pronounced HLCT character, with dye **6c** showing a slightly stronger CT component than **7c**.

**FIGURE 11:**
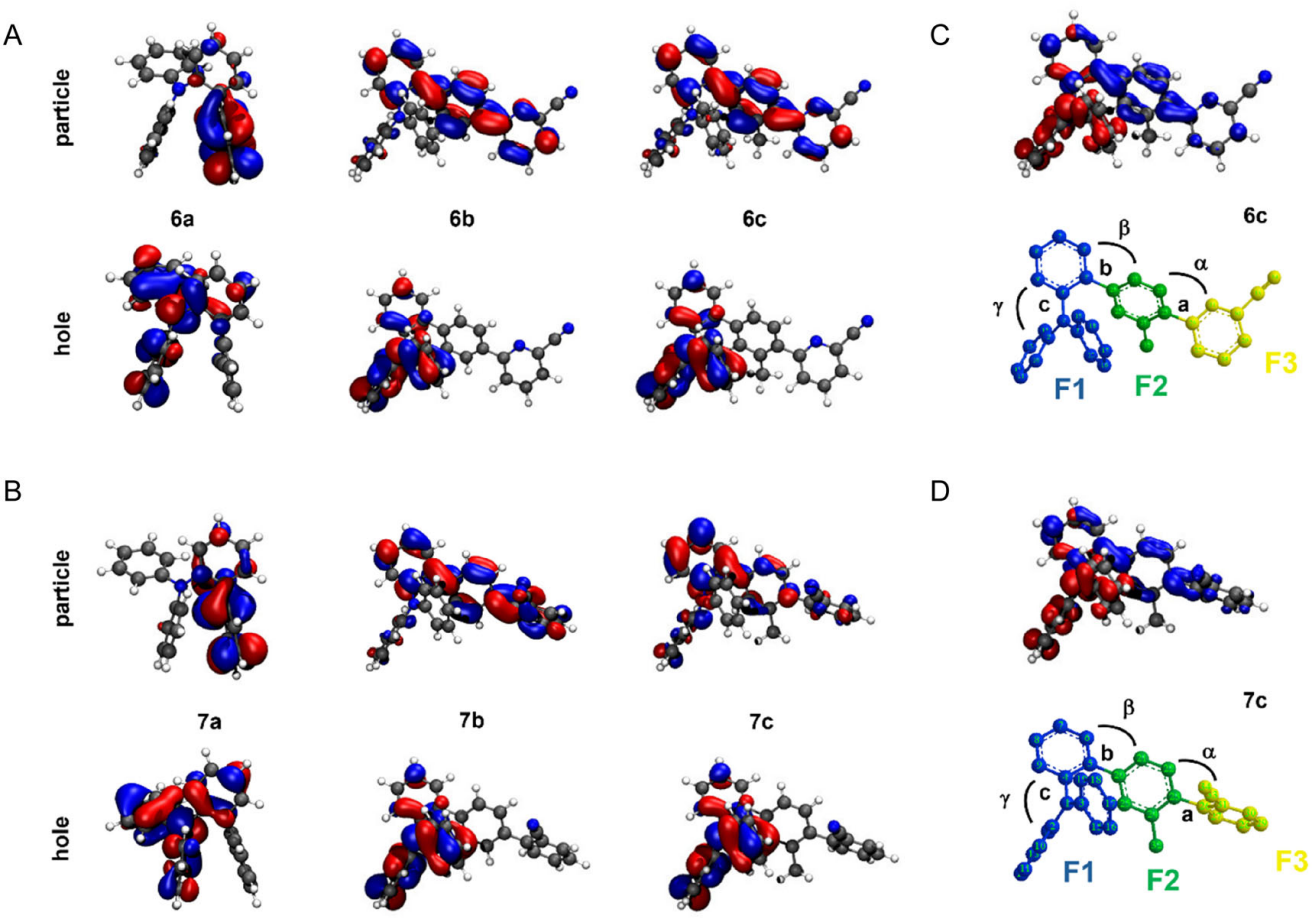
Natural transition orbitals (NTOs) with hole and particle contribution of the lowest‐energy excited state transition (S_0 _→ S_1_) of cyanopyridine‐based **6** (A) and benzonitrile‐based donor–acceptor conjugates **7** (B) (Gaussian 16, BMK/6‐311+G*, PCM toluene, isosurface value at 0.03). The leading single NTO pair accounts for 97.9% (**6a**), 98.0% (**6b**), 97.7% (**6c**), 97.2 (**7a**), 96.9% (**7b**), and 97.6% (**7c**) of the transition density. For representative compounds **6c** (C) and **7c** (D), the corresponding hole and electron densities (blue indicates electron accumulation and red electron depletion, isosurface value at 0.002) as determined using Multiwfn [78] are shown together with the results of the fragment analysis, including selected bond lengths (a, b, and c) and dihedral angles (α, β, and γ).

For the calculated emission, cam‐B3LYP‐based TD‐DFT calculations reveal emission bands that are characterized by HOMO to LUMO transitions with comparatively low to moderate oscillator strength of 0.0330–0.2214, being in good agreement with the experimental emission maxima for dyes **6a** and **7a** with deviations of 0.03 eV (Table S12). For the other compounds, deviations of up to 0.24 eV are observed significantly reduced compared to the longest wavelength absorption with deviations of up to 0.56 eV (Table S12). For conjugates **6, 7a,** and **7c,** the CT character is reduced for the emission (Δ*r* = 1.94–2.75 Å, *S* *_r_* = 0.36–0.57) compared to the longest wavelength absorption (Δ*r* = 2.34–3.09 Å, *S* *_r_* = 0.30–0.51), except for compound **7b,** where the CT character is increased for the emission to a Δ*r* value of 5.76 Å. The calculated emission of dye **7b** displays the highest deviation to the experiment of 0.24 eV, presumably not correctly describing the CT characteristic. Exemplified for compound **6b,** the emission is also calculated using the BMK functional underestimating the excited state energy and leading to deviations of 0.20 eV similar to the cam‐B3LYP functional overestimating the excited state energy (deviation of 0.17 eV) (Table S11). Since the deviations to the experiment are not reduced similar to the observed absorption behavior, the emission is not fully characterized using the BMK functional. The frontier molecular orbitals (Figures S94 and S95) and exemplified for compound **6b** the corresponding NTOs of the emission (Figure 12) also indicate HLCT character since the electron density overlaps on the TPA phenyl ring close to the phenylene bridge. Due to the reduced CT character of the emission compared to the absorption, further HLCT character is likely. The calculated transitions of compound **6b** are summarized in a Jablonski diagram (Figure 12). Upon photoexcitation, the molecular structure is planarized based on reduced dihedra angles α and β, resulting in the observed Stokes shifts. Compared to compound **6c** (see Section S7.9 and Figure S86), for dye **6b,** the excited‐state energies are decreased especially for the S_1_ state, resulting in the observed bathochromic shift of the emission maxima (Figure 12 and Table S12). For most compounds, the transition from the absorption to the emission geometry is accompanied by a decrease in bond lengths and a reduction of the dihedral angle α (Table S14).

**FIGURE 12:**
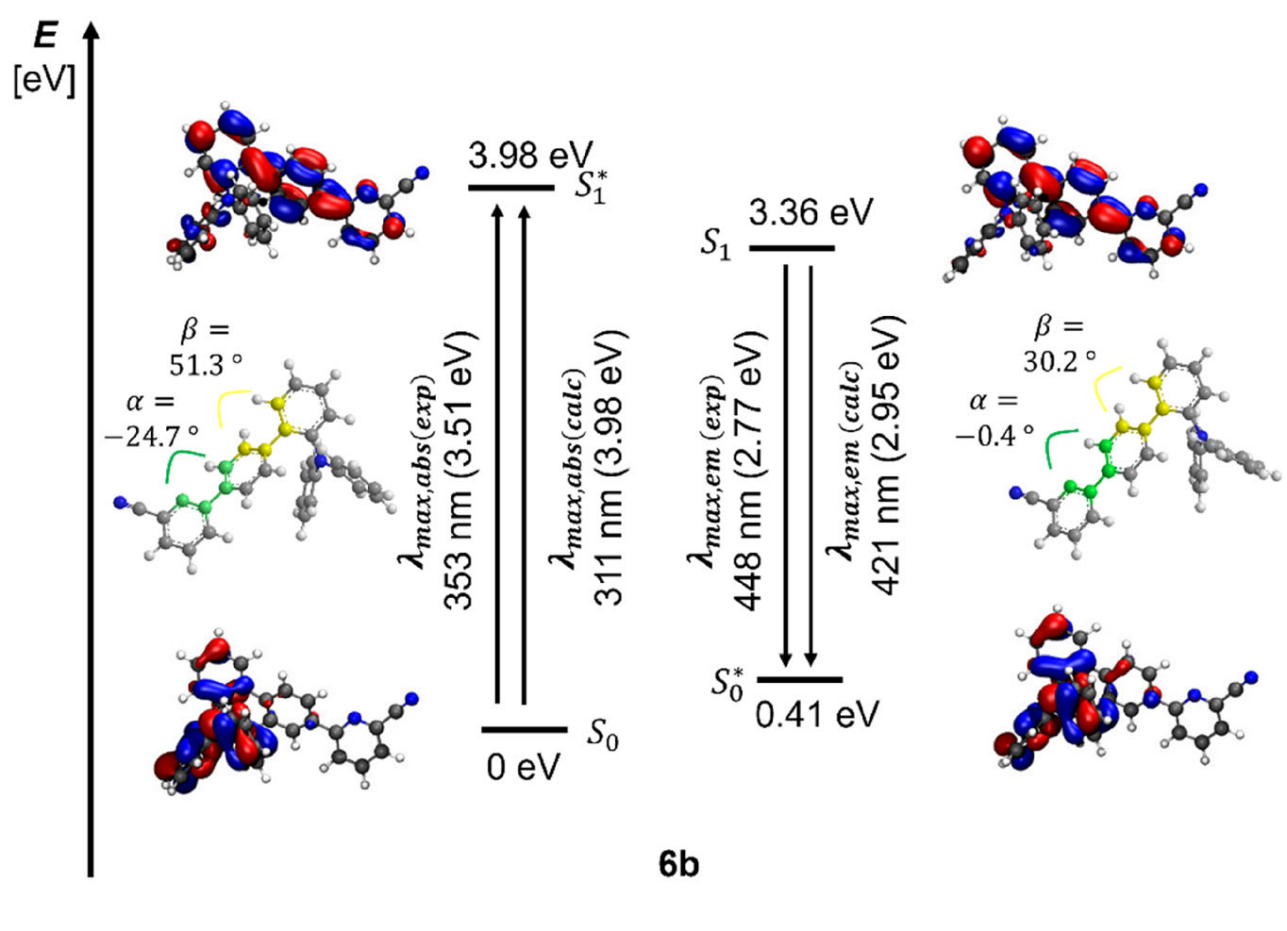
TD‐DFT‐calculated Jablonski diagram of compound **6b** including NTOs of the longest wavelength absorption and emission transition determined by Multiwfn [78]. Selected dihedra angles near the acceptor α (green) and donor β (yellow) moiety are shown. (Gaussian 16, cam‐B3LYP/6‐311+G*, PCM toluene, isosurface value at 0.03.)

To elucidate the structural origin of the observed differences in the emission properties, the relevant structural parameters of conjugates **6a**–**c** and **7a**–**c** were determined from the TD‐DFT‐optimized geometries using the cam‐B3LYP functional with toluene as solvent (Table S14 and Figure 11C,D). The acceptor–bridge (a), bridge–donor (b), and phenyl–diphenylamine (c) bond lengths exhibit only minor variations across all conjugates, ranging from 1.433–1.471 Å, 1.429–1.432 Å, and 1.403–1.417 Å, respectively. This indicates that the structural modifications predominantly affect the torsional geometry rather than the underlying covalent bond lengths. For conjugates **6a**–**c**, the acceptor–bridge dihedral angle α changes substantially from 20.0° for dye **6a** to −0.44° and −25.1° for dyes **6b** and **6c**, respectively. In parallel, the phenyl–diphenylamine dihedral angle γ decreases from 56.8° in dye **6a** to approximately 45° in the bridged derivatives. These conformational changes are accompanied by a progressive hypsochromic shift of the emission maximum in toluene from 457 nm for dye **6a** to 448 and 442 nm for dyes **6b** and **6c**, respectively, while the calculated oscillator strength increases markedly from 0.0438 (**6a**) to 0.2214 (**6b**) and 0.2179 (**6c**). The substantial increase in oscillator strength suggests that the conformational reorganization induced by the structural modifications enhances the electronic coupling and/or transition‐dipole character of the emissive excited state. A similar but more complex conformational dependence is observed for conjugates **7a**–**c**. Here, α undergoes a particularly pronounced change, from −137.3° for dye **7a** to 36.7° and 50.4° for dyes **7b** and **7c**, whereas γ increases from 38.0° to approximately 46°. The corresponding emission maxima are 434, 441, and 424 nm, respectively, while the oscillator strengths increase from 0.0330 (**7a**) to 0.1881 (**7b**) and 0.1529 (**7c**). Notably, the non‐monotonic evolution of the emission maxima despite the substantial changes in α demonstrates that the emission wavelength cannot be directly correlated with a single dihedral angle. Instead, the photophysical response appears to arise from the interplay between molecular conformation and the electronic structure of the excited state. Overall, the calculated geometries reveal a clear relationship between structural modification and conformational reorganization, particularly in the acceptor–bridge dihedral angle α. These conformational changes strongly affect the oscillator strength, indicating altered electronic coupling within the chromophores. In contrast, the emission maxima show no simple monotonic dependence on the individual dihedral angles, suggesting that the emission properties arise from the interplay of molecular conformation and excited‐state electronic structure. The relatively small effect of the additional methyl group in dyes **6b**/**6c**, compared with the more pronounced changes in dyes **7a**–**c**, further indicates that steric substitution primarily modulates molecular conformation rather than directly controlling the emission wavelength. Overall, molecular conformation represents an important design parameter for tuning the electronic and photophysical properties of these chromophores.

In summary, TD‐DFT calculations utilizing cam‐B3LYP functional were employed consistently to calculate the absorption and emission properties, enabling a direct comparison across the investigated compounds. For the absorption spectra, complementary TD‐DFT calculations utilizing BMK functional yielded in improved agreement with the experimental data, reducing the maximum deviation from 0.56 to 0.19 eV compared to cam‐B3LYP. In contrast, BMK did not provide a corresponding improvement for the calculated emission of representative compound **6b**, therefore, cam‐B3LYP was retained for the systematic emission calculations, for which the maximum deviation was 0.24 eV. As illustrated by NTO analysis, CDD plots, and CT‐related parameters, both the longest‐wavelength absorption and emission transitions exhibit HLCT character, arising from electron‐density overlap upon photoexcitation on the TPA phenyl ring adjacent to the acceptor or the phenylene‐bridged acceptor unit. TD‐DFT calculations demonstrate that steric substitution primarily modulates the torsional geometry of the chromophores, thereby altering their electronic coupling and oscillator strengths, while the emission wavelength reflects the combined effects of molecular conformation and excited‐state electronic structure rather than a single dihedral angle.

## 3 Conclusion

The BLEBS sequence provides a concise, modular one‐pot synthetic approach to novel *ortho*‐linked D–A conjugates with triarylamine donors and cyanopyridine or benzonitrile acceptors, enabling systematic variation of molecular topology and conformational constraint. Comprehensive electrochemical, photophysical, and theoretical investigations reveal efficient blue emission with high solid‐state PLQYs, narrow emission bandwidths, pronounced solvatochromism, AIE enhancement, and experimental evidence for long‐lived triplet‐state involvement consistent with an HLCT contribution to the emissive excited state. Across the investigated series, increasing conformational constraint through *ortho*‐linkage and phenylene extension systematically modulates D–A interactions as well as dihedral angles, excited‐state relaxation pathways, and emission characteristics. Beyond introducing a versatile synthetic platform, this study demonstrates that conformational control constitutes a powerful molecular design strategy for tailoring excited‐state properties in D–A chromophores. The resulting structure–property relationships provide insights for the rational development of future blue‐emissive organic materials.

## Funding

This study was supported by Deutsche Forschungsgemeinschaft (396890929 (GRK 2482) and Mu 1088/9‐1) and Fonds der Chemischen Industrie.

## Conflicts of Interest

The authors declare no conflicts of interest.

## Supporting information

Supplementary Material

## Data Availability

The data that support the findings of this study are available in the Supporting Information of this article.
