# Supplementary material for: Conformational Control of Excited‐State Properties in Ortho‐Linked Triarylamine‐Based Donor–Acceptor Conjugates
Source: ChemistryOpen. 2026 Sep 30;15(10):e70308. doi: 10.1002/open.70308 (PMC13627975; doi:10.1002/open.70308)
Supplement: Supplementary file 1 [file OPEN-15-e70308-s001.pdf]

Supporting Information

Table of Contents

1 General considerations ... 3
2 Synthesis ... 5
2.1 Starting materials ... 5
2.1.1 General procedure (**GP1**) for the synthesis of π-extended donors **3** ... 6
2.2 Product synthesis ... 9
2.2.1 General procedure (**GP2**) for the synthesis of cyanopyridine-based conjugates **6** and benzonitrile-based conjugates **7** ... 9
3 ^{1}H and ^{13}C NMR spectra ... 15
3.1 Structural assignment of compound **6b** by NMR spectroscopy ... 24
4 Cyclic voltammetry ... 28
4.1 Dimerization process of compound **3a** ... 28
4.2 Dimerization process of compounds **6a**–**c** and **7a**–**c** ... 28
4.3 Cyclic voltammograms of conjugates **6** and **7** ... 30
5 UV/Vis absorption and emission spectra of **6** and **7** ... 33
5.1 Emission spectra in the solid state ... 33
5.2 UV/Vis absorption and emission spectra in solution ... 37
5.3 Emission spectra in a PMMA matrix ... 48
5.4 Rotamer analysis ... 53
5.5 Solvatochromism ... 55
5.6 Emission spectra upon induced aggregation ... 59
6 Photoluminescence lifetime measurements ... 61
6.1 Decay spectra in the solid state ... 61
6.2 Decay spectra in solution ... 62
6.3 Decay spectra in a PMMA matrix ... 65
7 Quantum chemical calculations ... 68
7.1 Quantum chemical calculation data of 6-(2-(diphenylamino)phenyl)picolinonitrile (**6a**) ... 68
7.2 Quantum chemical calculation data of 6-(2′-(diphenylamino)-[1,1′-biphenyl]-4-yl)picolinonitrile (**6b**) ... 79
7.3 Quantum chemical calculation data of 6-(2′-(diphenylamino)-3-methyl-[1,1′-biphenyl]-4-yl)picolinonitrile (**6c**) ... 110
7.4 Quantum chemical calculation data of 2′-(diphenylamino)-[1,1′-biphenyl]-2-carbonitrile (**7a**) ... 123

7.5 Quantum chemical calculation data of 2″-(diphenylamino)-[1,1′:4′,1″-terphenyl]-2-carbonitrile (**7b**) 134
7.6 Quantum chemical calculation data of 2″-(diphenylamino)-2′ -methyl-[1,1′:4′,1″-terphenyl]-2-carbonitrile (**7c**) 148
7.7 Quantum chemical calculation data of 6-(2′-(diphenylamino)-[1,1′-biphenyl]-4-yl)picolinonitrile (**6b**) in the gas phase 162
7.8 Quantum chemical calculation data of rotamer analysis of compound **7b** 164
7.8.1 Rotation acceptor unit (BMK/6-31+G*) 164
7.8.2 Rotation bridged acceptor unit (BMK/6-31+G*) 165
7.9 Calculated absorption spectra and transitions in comparison to the experimental data 170
7.10 NTO analysis and excited-state character as well as charge-transfer analysis exemplified for compound **6c** and **7c** 178
7.11 Frontier molecular orbital contributions to transitions to excited states and orbital energy differences in toluene 181
8 References 187

1 General considerations

All reactions were conducted in oven-dried Schlenk glassware using septa and syringes under inert nitrogen atmosphere. 2-Bromo-*N*,*N*-diphenylaniline is a known compound and was synthesized according to literature procedures.[1] Reaction temperatures were controlled using preheated silicone oil or cooling baths (dry ice/acetone for −78 °C). All reagents and catalyst were purchased reagent-grade from BLD Pharm, Acros Organics, Alfa Aesar and VWR Chemicals and used without purification, if not indicated otherwise. THF was dried using a solvent purification system (MB-SPS 800 solvent drying system by M. Braun). Toluene was dried over activated 4 Å molecular sieves. Reaction progress was monitored qualitatively by TLC Silica gel 60 F234 aluminium sheets obtained from MACHEREY-NAGEL GmbH & Co. KG. The spots were visualized under UV light at 254 and 365 nm. Purification of the compounds was performed on silica gel 60 M (0.04−0.063 mm) from MACHEREY-NAGEL GmbH & Co. KG by flash technique under pressure of 2 bar. The crude mixtures were absorbed on Celite 545 form Carl Roth GmbH & Co. KG prior to column chromatographic purification. As eluent *n*-hexane and mixtures of *n*-hexane/ethyl acetate or *n*-hexane/dichloromethane were freshly distilled on the rotary evaporator. During recrystallization, heating was stopped once the crude product was completely dissolved. By leaving the flask in the silicone oil bath, slow cooling of the solution was ensured. The isolated products were dried for at least 24 h under vacuum (10^{-2} to 10^{-3} mbar).

Characterization was primarily carried out by the department CeMSA@HHU of the Heinrich Heine University Düsseldorf. ^{1}H, ^{13}C and 135-DEPT ^{13}C NMR as well as 2D NMR spectra were recorded on Bruker AVIII-300 and AVIII-600 spectrometers. Deuterated solvents (DMSO-d_6 and acetone-d_6) were used and resonances of the solvents served as internal standard (DMSO-d_6: ^{1}H δ 2.50, ^{13}C δ 39.52; water* in DMSO-d_6 ^{1}H δ 3.33, acetone-d_6: ^{1}H δ 2.05, ^{13}C δ 29.84; 206.26, water* in acetone-d_6 ^{1}H δ 2.84). Signal multiplicities were abbreviated as follows: s: singlet; d: doublet; t: triplet; q: quatet; dd: doublet of doublet; ddd: doublet of doublet of doublets; dt: doublet of triplet; m: multiplet. The type of carbon nucleus was determined based on 135-DEPT ^{13}C NMR spectra and are denoted as CH_3 (primary), CH_2 (secondary), CH (tertiary), and quaternary carbon nuclei with C_{quat}. Structural elucidation was supported by 2D NMR (HSQC, HMBC, NOESY) spectra. Electron ionization (EI) mass spectra were recorded on a Finnigan MAT TSQ 7000 instrument. Reported are all EI mass peaks with an intensity greater than 10% of the base peak and the mole peak. Infrared (IR) spectra were obtained using the device Shimadzu IR Affinity-1, equipped with an attenuated total reflection (ATR) unit. Signal intensities are abbreviated as follows: s (strong, 0–75%), m (medium, 75–90%), w (weak, 90–100%). Melting points (uncorrected) were measured using a Büchi Melting Point B-540 device. Combustion analyses were performed on a Perkin Elmer Series II Analyser 2400 in the microanalytical laboratory of the Institute for Pharmaceutical and Medicinal Chemistry at Heinrich Heine University.

* Due to the water content in DMSO-d_6 and acetone-d_6, a signal for HDO is observed in the ^{1}H NMR spectra and labeled as “water”.

UV/Vis absorption spectra were recorded in cyclohexane, toluene, ethyl acetate, THF, dichloromethane, acetonitrile and ethanol in high performance liquid chromatography (HPLC) grade at 298 K on a Perkin–Elmer UV/Vis/NIR Lambda 19 spectrometer. Molar extinction coefficients ε were determined from

absorption measurements at five different concentrations. Emission spectra and fluorescence lifetimes were measured under the same solvent conditions at 293 K using a PerkinElmer LS55 or an Edinburgh FS5 spectrometer. A xenon vapor lamp was employed for steady-state emission measurements, while fluorescence lifetimes were recorded using a pulsed diode laser (EPL-375, 372.2 nm) in time-correlated single-photon counting (TCSPC) mode in count ranges between 10^3 and 10^6 counts as the stop condition in each case. To determine decay times in the order of seconds the multichannel scaling (MCS) mode was used in combination with a pulsed xenon vapor lamp (5 W microsecond flash lamp for the FS5). The instrumental response function (IRF) was recorded under the same measurement conditions, but with detection of the excitation wavelength. A dilute Ludox® water solution was used as the scattering solution for this purpose. Decay analysis was performed using the FS5 software, with fits selected based on residuals between 1.0 to 1.3. All oxygen-free samples were prepared by injecting argon for 60 minutes. For film preparation, 1 wt% of the compound (0.2 mg) and polymethyl methacrylate (PMMA) (20 mg) were dissolved in dichloromethane (2 mL), cast into molds, and then cured at 298 K overnight. Low-temperature emission measurements were conducted using the FS5 temperature module with liquid nitrogen cooling. Absolute quantum yields were determined using an integrating sphere with excitation at the corresponding longest wavelength absorption or excitation maximum at the FS5 spectrometer with software by Edinburgh instruments.

Cyclic voltammetry (CV) experiments were performed using a Princeton Applied Reasearch VersaSTAT3 potentiostat/galvanostat in a small volume cell (4.0 mL) with a three-electrode setup under argon in dry and degassed dichloromethane at 293 K. A platinum disk (1 mm diameter) served as the working electrode, a platinum wire (0.5 mm diameter) as the counter electrode and a silver/silver chloride electrode filled with saturated sodium chloride solution c = 3.0 M as the reference electrode. Tetrabutylammonium hexafluorophosphate at a concentration of c = 0.1 M was used as electrolyte. Potentials were calibrated using decamethylferrocene (DMFc) $[FeCp^*_2]/[FeCp^*_2]^+$ as an internal standard and referenced against ferrocene $[FeCp_2]/[FeCp_2]^+$ ($E_0^{0/+1}$ = 0 mV)[2] which was used as in internal starting point. This procedure provided a difference in oxidation potentials of -546 mV from DMFc to Fc. Reported potentials are the arithmetic average of the potential by the different scan rates of 100, 250, 500 and 1000 mVs^{-1} extrapolated to 0 mVs^{-1}.

Quantum chemical calculations were carried out utilizing the high-performance computing (HPC) cluster HILBERT of the Center for Information and Media Technology (ZIM) and provided Gaussian 16[3] software package at the Heinrich Heine University Düsseldorf. Time-dependent density functional theory (TD-DFT) was employed and optimized geometries confirmed as minima (NImag = 0) by frequency analysis. Solvent effects were included using the polarizable continuum model (PMC) with toluene as the solvent. The applied functionals and basis sets are specified in the corresponding results sections.

2 Synthesis

2.1 Starting materials

The donor moiety, 2-bromo-*N*,*N*-diphenylaniline (**1**) was synthesized via palladium-catalyzed Buchwald-Hartwig amination of diphenylamine (**DPh**) in accordance with a reported literature procedure[1] (Scheme S1). Notably, extending the reaction time to 23 h, compared to the reported 12 h, resulted in an increased isolated yield of 96%.

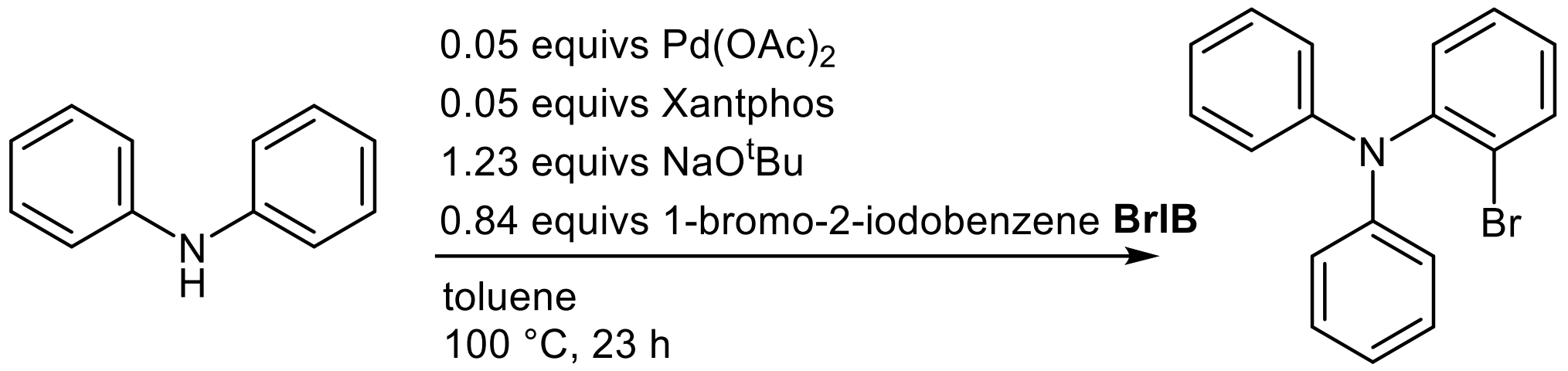

DPh **1**, 96%

Scheme S1. Buchwald-Hartwig coupling towards the donor moiety **1**.

Synthesis conditions and characterization

2-Bromo-*N*,*N*-diphenylaniline (1)[1]

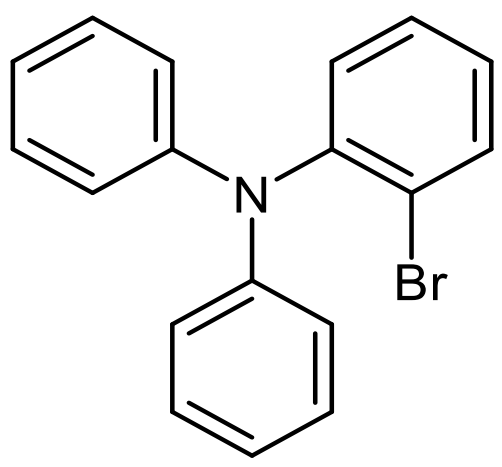

1

$C_{18}H_{14}BrN_2$

[324.22]

Under nitrogen in a dried Schlenk tube, 0.74 g diphenylamine **DPh** (1.00 equivs, 4.30 mmol), 0.04 g palladium(II) acetate (0.05 equivs, 0.22 mmol), 0.51 g sodium *tert*-butoxide (1.23 equivs, 5.30 mmol), and 0.11 g (9,9-Dimethyl-9*H*-xanthene-4,5-diyl)bis(diphenylphosphane) (Xantphos) (0.05 equivs, 0.22 mmol) were suspended in 5.50 mL toluene. Then 0.46 mL 1-bromo-2-iodobenzene **BrIB** (0.84 equivs, 3.60 mmol) was added and stirred at 100 °C for 23 h. After cooling to room temperature, destilled water (5 mL) was added to the reaction mixture and the organic phase was separated. The aqueous phase was extracted with dichloromethane (3 x 5 mL). The combined organic phase was dried with anhydrous magnesium sulphate. The precipitate was filtered off, and the solvent was removed under reduced pressure. The reaction mixture was diluted with dichloromethane and adsorbed onto Celite©. The crude product was purified by column chromatography (*n*-hexane/dichloromethane 97:3). The purified compound was obtained as a colourless solid with a yield of 1.12 g (3.45 mmol, 96%). Mp. 55 °C (Lit.: 63 °C)[4]. R_f = 0.43 (*n*-hexane/dichloromethane 97:3). ^{1}H NMR (300 MHz, DMSO-d$_6$) δ = 7.75 (dd, ^3J = 8.0 Hz, ^4J = 1.5 Hz, 1H), 7.46 (ddd, ^3J = 8.0 Hz, ^3J = 7.3 Hz, ^4J = 1.5 Hz, 1H), 7.33–7.21 (m, 6H), 7.02–6.91 (m, 2H), 6.91–6.82 (m, 4H). ^{13}C NMR (75 MHz, DMSO-d$_6$) δ = 146.3 (C_{quat}), 144.6 (C_{quat}), 134.3 (CH), 131.9 (CH), 129.6 (CH), 129.3 (CH), 128.3 (CH), 123.3 (C_{quat}), 122.0 (CH), 121.2

(CH). EI MS (70 eV, *m/z* (%)): 326 ($[M]^+$, 7), 325 ($[^{81}Br-M]^+$, 42), 324 ($[M]^+$, 6), 323 ($[^{79}Br-M]^+$, 41), 245 (28), 244 ($[C_{18}H_{14}N]^+$, 100), 243 ($[C_{18}H_{13}N]^{2+}$, 40), 242 (21), 241 (19), 167 ($[C_{12}H_9N]^+$, 24), 166 ($[C_{12}H_8N]^{2+}$, 46), 122 (20), 120 (20), 77 ($[C_6H_5]^+$, 5).

2.1.1 General procedure (GP1) for the synthesis of π-extended donors 3

Under nitrogen in a dried Schlenk tube with magnetic stir bar compound **1** (162 mg, 0.50 mmol, 1.00 equiv) was dissolved in dry THF (2.5 mL) and cooled to −78 °C (dry ice/acetone bath). Then, *n*-butyllithium solution in hexanes (0.39 mL, 0.55 mmol, 1.10 equiv, 1.39 M) was added dropwise and the solution was stirred at – 78 °C for 20 min. Afterwards, trimethyl borate (0.08 mL, 0.65 mmol, 1.30 equiv) was added and the solution was warmed to room temperature. Then, $Pd(PPh_3)_4$ (28.9 mmol, 5 mol%), bromo-iodobenzene **2** (0.55 mmol, 1.10 equiv), and sodium hydroxide (24 mg, 0.60 mmol, 1.20 equiv) were added to the reaction mixture. The solution was stirred at 100 °C (oil bath) for 36 h. After cooling to room temperature, the reaction mixture was diluted with ethyl acetate and purified by flash chromatography on silica gel (*n*-hexane/ethyl acetate 150:1). After recrystallisation from *n*-hexane and drying under high vacuum compounds **3** were obtained as solids (Table S1).

Table S1. Experimental details for the synthesis of π-extended donor moieties **3**.

entry	bromo-iodo-benzene 2	π-extended donor 3
**1**	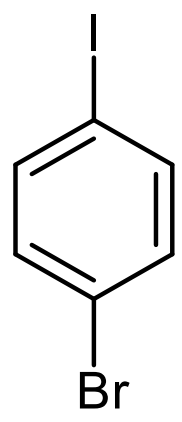 1-bromo-4-iodo-benzene, **2a** (156 mg, 0.55 mmol)	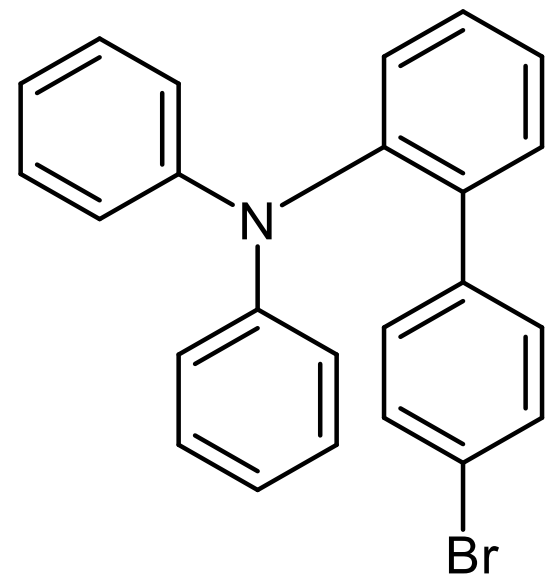 **3a** (127 mg, 0.32 mmol, 64%)
**2**	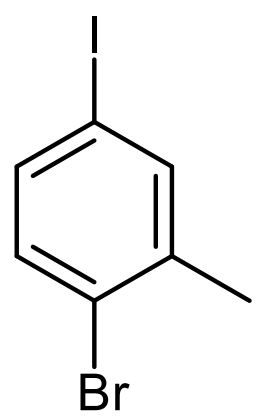 1-bromo-4-iodo-2-methyl-benzene, **2b** (163 mg, 0.55 mmol)	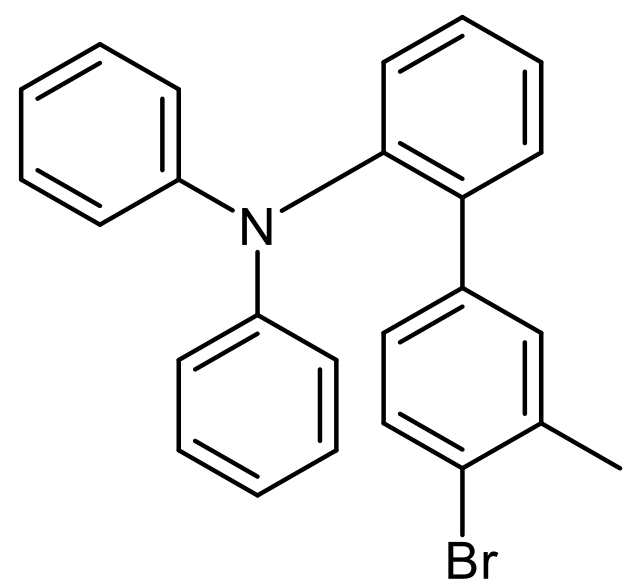 **3b** (140 mg, 0.34 mmol, 68%)

Synthesis conditions and characterization

4'-Bromo-*N*,*N*-diphenyl-[1,1'-biphenyl]-2-amine (3a)

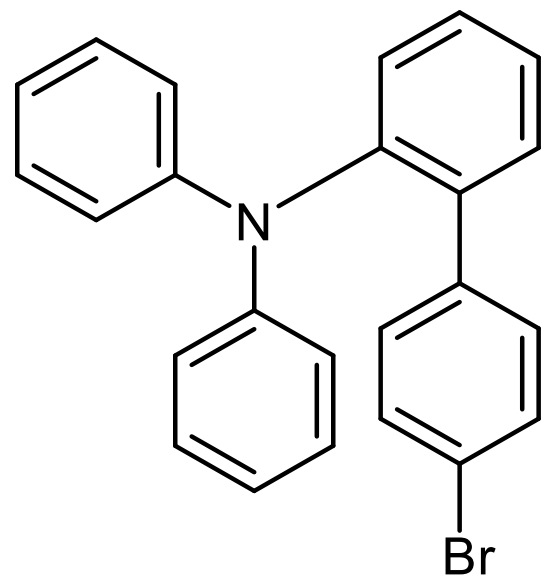

3a

$C_{24}H_{18}BrN$

[400.32]

The synthesis was performed by GP1. After purification by flash column chromatography on silica gel (*n*-hexane/ethyl acetate 150:1) and recrystallization from *n*-hexane, compound **3a** (127 mg, 0.32 mmol, 64%) was isolated as a colorless solid, Mp 162 °C. R_f = 0.50 (*n*-hexane/ethyl acetate 100:1). ^{1}H NMR (300 MHz, DMSO-d$_6$): δ = 7.47–7.39 (m, 1H), 7.36–7.28 (m, 4H), 7.24 (dt, ^3J = 7.8 Hz, ^4J = 1.0 Hz, 1H), 7.11 (ddd, ^3J = 9.0 Hz, ^3J = 7.0 Hz, ^4J = 1.9 Hz, 6H), 6.83 (td, ^3J = 7.3 Hz, ^4J = 1.2 Hz, 2H), 6.80–6.74 (m, 4H). ^{13}C NMR (75 MHz, DMSO-d$_6$): δ = 192.5 (C_{quat}), 146.7 (C_{quat}), 144.0 (C_{quat}), 138.2 (C_{quat}), 131.4 (CH), 130.6 (CH), 130.1 (CH), 129.4 (CH), 129.2 (CH), 128.7 (CH), 126.1 (CH), 121.4 (CH), 120.3 (C_{quat}). EI MS (70 eV, *m*/*z* (%)): 402 ([M]$^+$, 22), 401 ([^{81}Br-M]$^+$, 99), 400 (26), 399 ([^{79}Br-M]$^+$, 100), 323 (11), 321 (14), 320 ([$C_{24}H_{18}N$]$^+$, 18), 319 ([$C_{24}H_{18}N$]$^{2+}$, 50), 318 (14), 244 ([$C_{18}H_{14}N$]$^+$, 21), 243 ([$C_{18}H_{14}N$]$^{2+}$, 21), 242 (23), 241 (27), 217 (11), 215 (10), 167 ([$C_{12}H_9N$]$^+$, 15), 166 ([$C_{12}H_9N$]$^{2+}$, 13), 160 (19), 152 (20), 151 (13), 139 (16), 121 (15), 77 ([C_6H_5]$^+$, 6). IR $\tilde{\nu}$ [cm^{-1}] = 3057 (w), 3024 (w), 1585 (m), 1572 (w), 1487 (m), 1474 (m), 1439 (m), 1391 (w), 1310 (m), 1279 (m), 1269 (m), 1234 (w), 1177 (w), 1153 (w), 1111 (w), 1098 (w), 1072 (m), 1043 (w), 1005 (m), 947 (w), 920 (w), 864 (w), 829 (m), 748 (s), 694 (s), 671 (w), 621 (m). Anal. calcd. for $C_{24}H_{18}BrN$ (400.32): C 72.01, H 4.53, N 3.50; Found C 72.08, H 4.50, N 3.44.

4′-Brom-3′-methyl-*N,N*- diphenyl-(1,1′-biphenyl)-2-amine (3b)

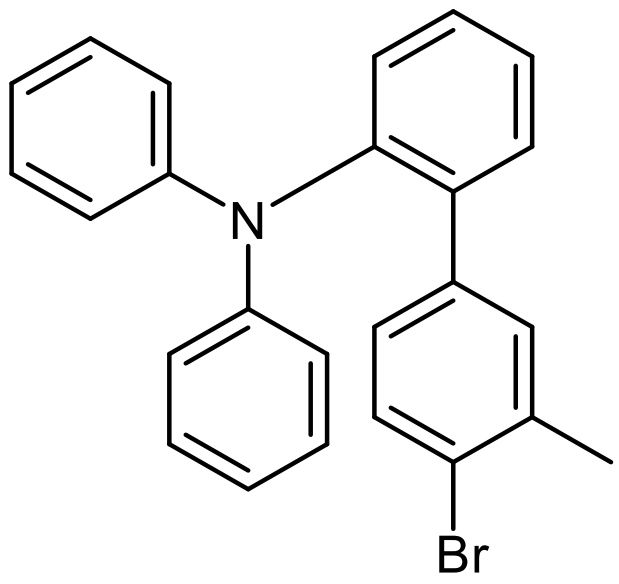

3b

$C_{25}H_{20}BrN$

[414.35]

The synthesis was performed by GP1. After purification by flash column chromatography on silica gel (*n*-hexane/ethyl acetate 150:1) and recrystallization from *n*-hexane, compound **3b** (140 mg, 0.34 mmol, 68%) was isolated as a colorless solid, Mp 90 °C. R_f = 0.41 (*n*-hexane/ethyl acetate 100:1). ^{1}H NMR (300 MHz, acetone-d$_6$): δ = 7.43 (ddd, ^3J = 7.8 Hz, ^3J = 6.5 Hz, ^4J = 2.5 Hz, 1H), 7.39–7.30 (m, 3H), 7.25 (dd, ^3J = 8.0 Hz, ^4J = 1.3 Hz, 1H), 7.15–7.07 (m, 5H), 6.99 (dd, ^3J = 8.3 Hz, ^4J = 2.3 Hz, 1H), 6.88–6.78 (m, 6H), 2.19 (s, 3H). ^{13}C NMR (151 MHz, acetone-d$_6$): δ = 148.6 (C_{quat}), 145.8 (C_{quat}), 140.2 (C_{quat}), 140.0 (C_{quat}), 137.7 (C_{quat}), 132.5 (CH), 132.4 (CH), 132.1 (CH), 130.4 (CH), 130.1 (CH), 129.7 (CH), 128.6 (CH), 126.9 (CH), 123.7 (C_{quat}), 122.9 (CH), 122.5 (CH), 22.8 (CH_3). EI MS (*m*/*z* (%)): 416 (24), 415 ([^{81}Br-M]$^+$, 99), 414 ([M]$^+$, 28), 413 ([^{79}Br-M]$^+$, 100), 334 ([$C_{25}H_{20}N$]$^+$, 13), 333 ([$C_{25}H_{20}N$]$^{2+}$, 39), 321 (39), 320 ([$C_{24}H_{18}N$]$^+$, 10), 319 ([$C_{24}H_{18}N$]$^{2+}$, 13), 318 (11), 256 (10), 254 (11), 245 (42), 244 ([$C_{18}H_{14}N$]$^+$, 39), 243 ([$C_{18}H_{14}N$]$^{2+}$, 33), 242 (19), 241 (24), 167 ([$C_{12}H_9N$]$^+$, 34), 166 ([$C_{12}H_9N$]$^{2+}$, 40), 152 (15), 151 (10), 77 ([C_6H_5]$^+$, 11). IR $\tilde{\nu}$ [cm^{-1}] = 3063 (w), 3021 (w), 2984 (w), 2963 (w), 2924 (w), 2361 (w), 1896 (w), 1585 (m), 1487 (m), 1468 (m), 1441 (m), 1381 (w), 1310 (m), 1294 (m), 1281 (m), 1271 (m), 1240 (w), 1175 (w), 1155 (w), 1109 (w), 1076 (w), 1051 (w), 1026 (m), 997 (w), 978 (w), 951 (w), 922 (w), 876 (w), 864 (w), 824 (m), 812 (w), 764 (m), 750 (s), 727 (w), 692 (s), 621 (m). Anal. calcd. for $C_{25}H_{20}BrN$ (414.35): C 72.47, H 4.87, N 3.38; Found C 72.61, H 4.85, N 3.29.

2.2 Product synthesis

2.2.1 General procedure (GP2) for the synthesis of cyanopyridine-based conjugates 6 and benzonitrile-based conjugates 7

Under nitrogen in a dried Schlenk tube **1** or **3** (1.00 equiv) was dissolved in dry THF (0.2 mmol/mL) and cooled to −78 °C (dry ice/acetone bath). Then, *n*-butyllithium solution in hexanes (1.10 equiv, 1.39 M) was added dropwise and the solution was stirred for 20 min at – 78 °C. Afterwards, trimethyl borate (1.30 equiv) was added and the solution was warmed to room temperature. Then, $Pd(PPh_3)_4$ (5 mol%), acceptor (1.10 equiv) and sodium hydroxide (1.50 equiv) was added to the reaction mixture. The solution was stirred at 100 °C (oil bath) for 20–43 h. After cooling to room temperature the reaction mixture was diluted with ethyl acetate and purified by flash chromatography on silica gel (*n*-hexane/ethyl acetate). After recrystallisation from ethanol and then drying under high vacuum compounds **6** and **7** were optained as solids (Table S2).

Table S2. Experimental details for the synthesis of the donor-acceptor conjugates **6** and **7**.

entry	donor 1 or 3	acceptor 4 or 5	reaction time	compound 6 or 7
1[a]	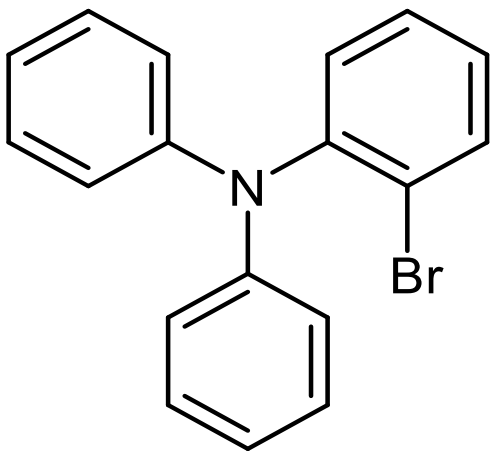 **1** (162 mg, 0.50 mmol)	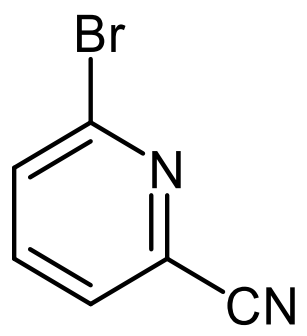 **4** (101 mg, 0.55 mmol)	28 h	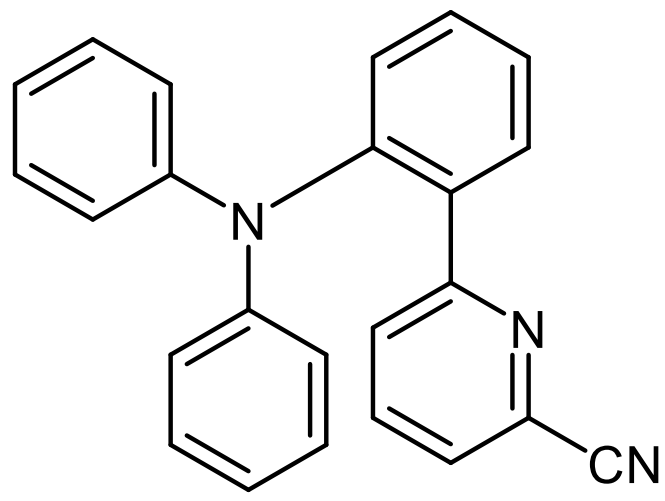 **6a** (150 mg, 0.42 mmol, 84%)
2[a]	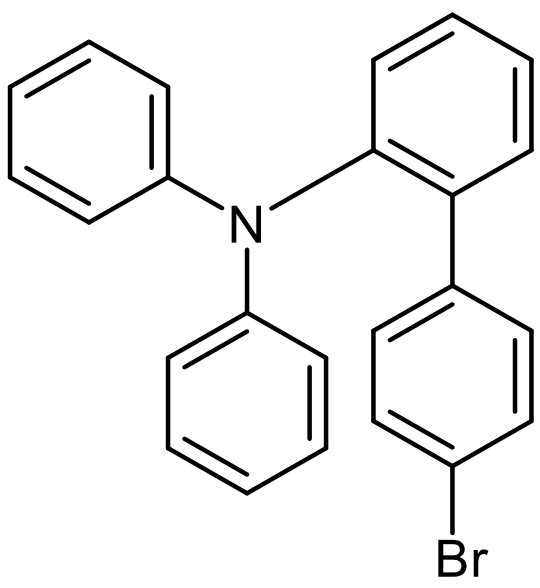 **3a** (200 mg, 0.50 mmol)	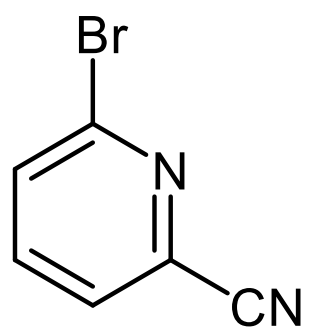 **4** (101 mg, 0.55 mmol)	21 h	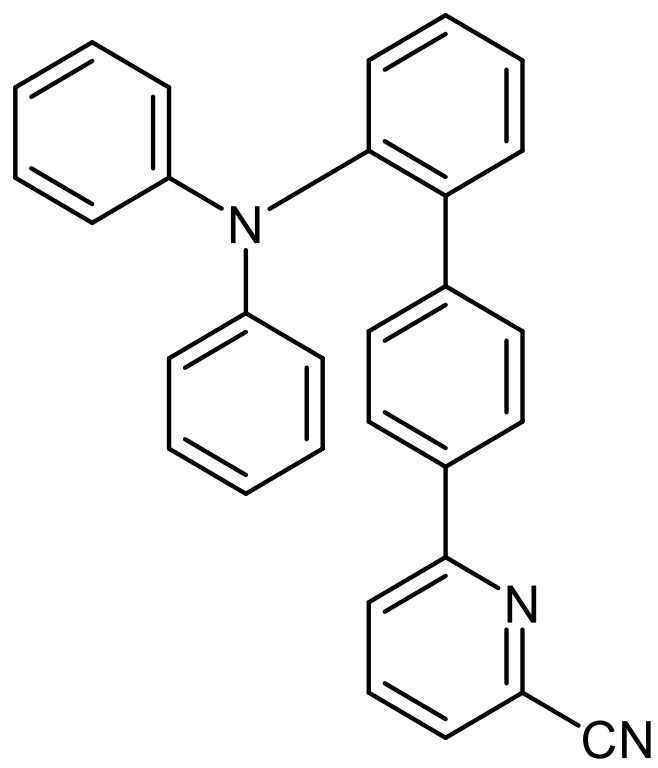 **6b** (174 mg, 0.41 mmol, 82%)

**3**[a]	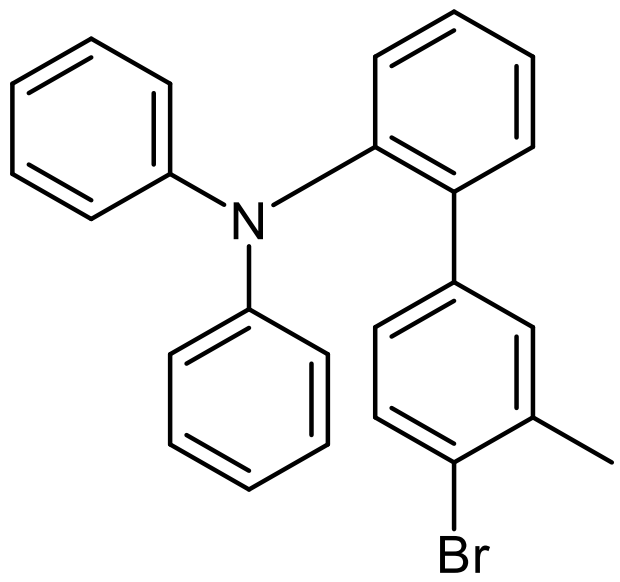<br>**3b**<br>(207 mg, 0.50 mmol)	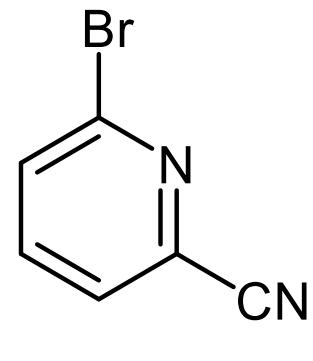<br>**4**<br>(101 mg,<br>0.55 mmol)	20 h	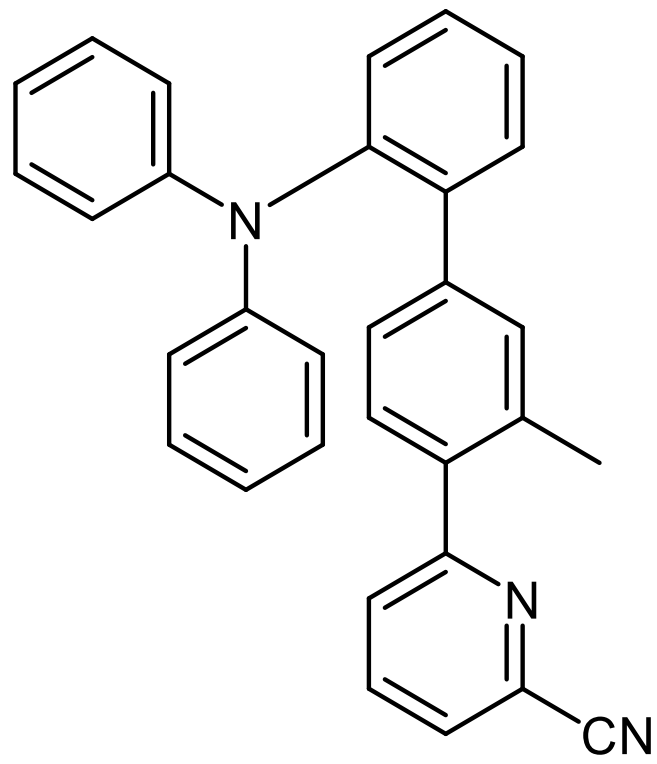<br>**6c**<br>(170 mg, 0.34 mmol, 77%)
**4**[b]	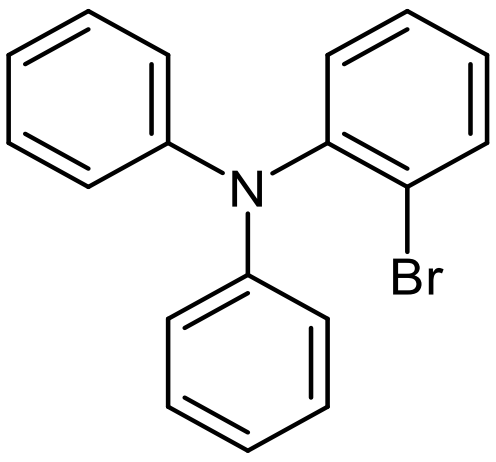<br>**1**<br>(113 mg, 0.35 mmol)	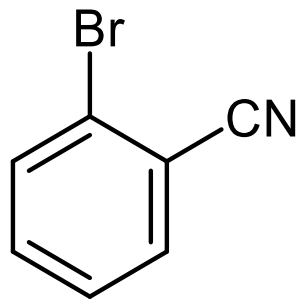<br>**5**<br>(71 mg, 0.39 mmol)	43 h	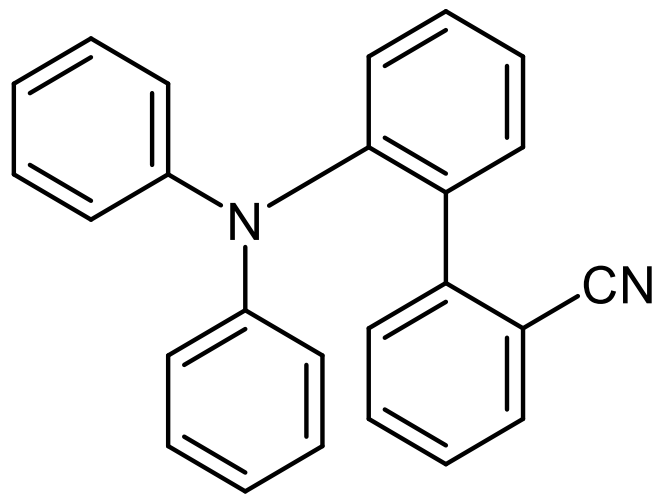<br>**7a**<br>(69.6 mg, 0.20 mmol, 57%)
**5**[a]	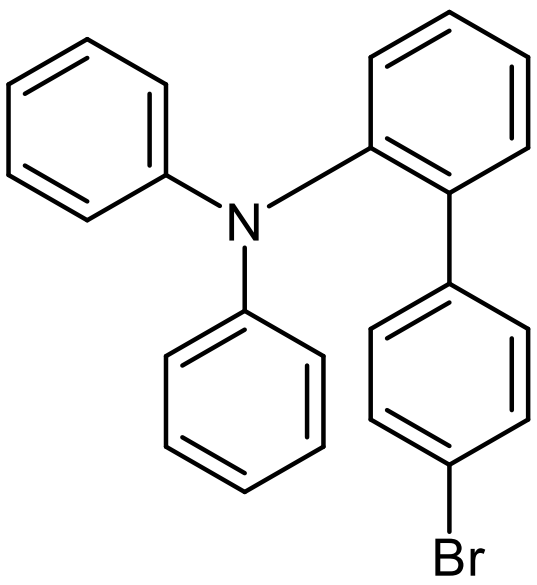<br>**3a**<br>(188 mg, 0.47 mmol)	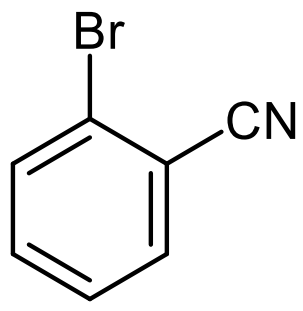<br>**5**<br>(93 mg, 0.51 mmol)	28 h	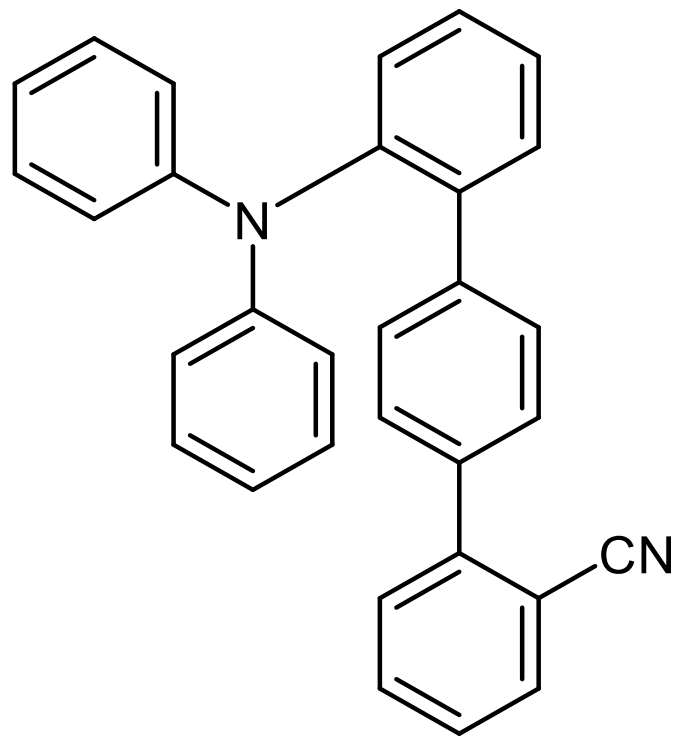<br>**7b**<br>(150 mg, 0.36 mmol, 77%)
**6**[c]	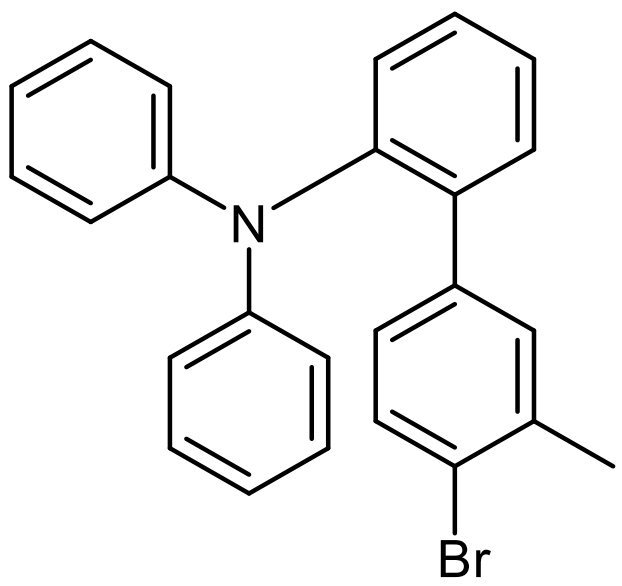<br>**3b**<br>(174 mg, 0.42 mmol)	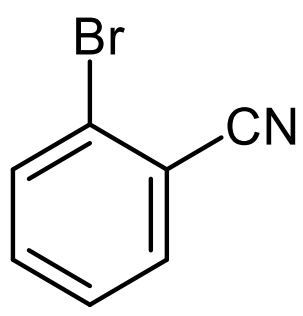<br>**5**<br>(84 mg, 0.46 mmol)	24 h	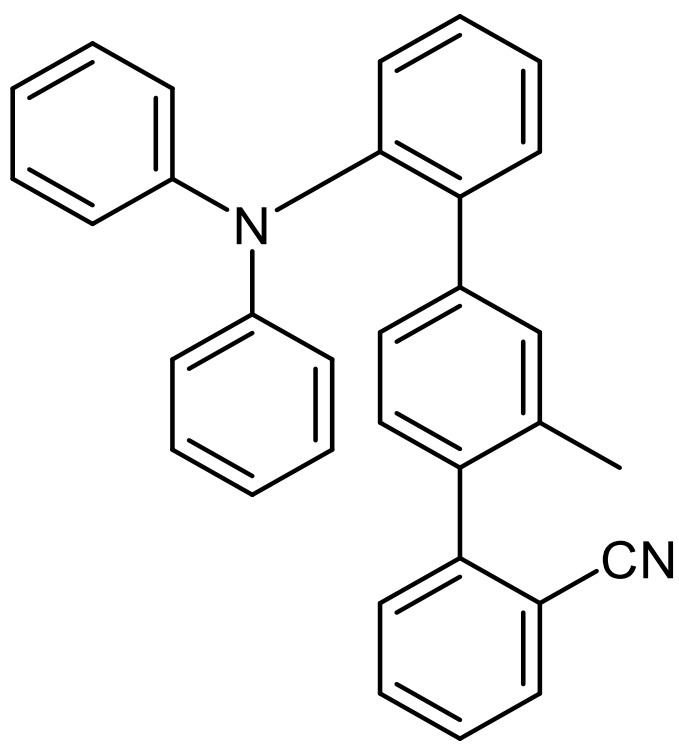<br>**7c**<br>(130 mg, 0.31 mmol, 73%)

[a] For the reaction was used: THF (2.5 mL), *n*-BuLi (0.39 mL), $B(OMe)_3$ (0.08 mL), $Pd(PPh_3)_4$ (29 mg), NaOH (30 mg). [b] For the reaction was used: THF (1.75 mL), *n*-BuLi (0.28 mL), $B(OMe)_3$ (0.06 mL), $Pd(PPh_3)_4$ (20 mg),

NaOH (21 mg). [c] For the reaction was used: THF (2.1 mL), *n*-BuLi (0.33 mL), $B(OMe)_3$ (0.07 mL), $Pd(PPh_3)_4$ (25 mg), NaOH (25 mg).

Synthesis conditions and characterization

6-(2-(Diphenylamino)phenyl)picolinonitrile (6a)

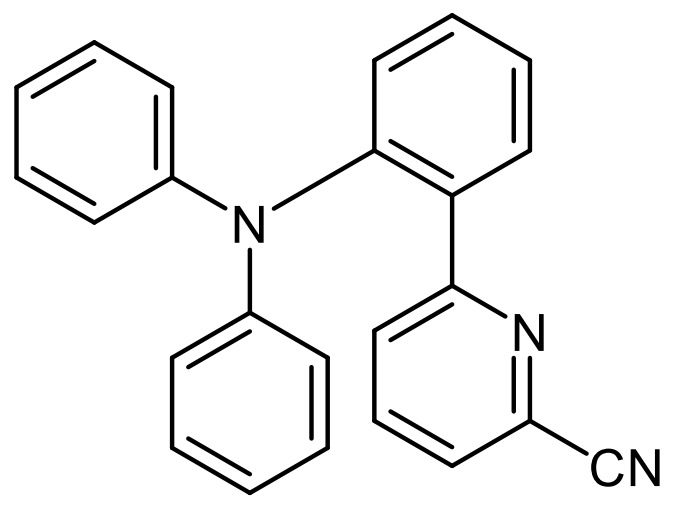

6a

$C_{24}H_{17}N_3$

[347.42]

The synthesis was performed by GP2. After purification by flash column chromatography on silica gel (*n*-hexane/ethyl acetate 10:1) compound **6a** (150 mg, 0.42 mmol, 84%) was isolated as a colorless solid, Mp 128 °C. R_f = 0.26 (*n*-hexane/ethyl acetate 10:1). ^{1}H NMR (300 MHz, acetone-d$_6$): δ = 7.87–7.79 (m, 2H), 7.67–7.58 (m, 2H), 7.52 (td, ^3J = 7.6 Hz, ^4J = 1.7 Hz, 1H), 7.38 (td, ^3J = 7.5 Hz, ^4J = 1.3 Hz, 1H), 7.24 (dd, ^3J = 8.0 Hz, ^4J = 1.3 Hz, 1H), 7.18–7.08 (m, 4H), 6.93–6.82 (m, 6H). ^{13}C NMR (75 MHz, acetone-d$_6$): δ = 159.9 (C_{quat}), 148.3 (C_{quat}), 146.3 (C_{quat}), 138.3 (CH), 137.1 (C_{quat}), 133.8 (C_{quat}), 132.6 (CH), 131.7 (CH), 129.9 (CH), 129.8 (CH), 128.4 (CH), 127.4 (CH), 126.5 (CH), 123.3 (CH), 123.0 (CH), 118.1 (C_{quat}). EI MS (70 eV, *m*/*z* (%)): 348 ([M]$^+$, 22), 347 ([M]$^+$, 100), 346 (68), 244 ($[C_{18}H_{14}N]^+$, 10), 243 ($[C_{18}H_{14}N]^{2+}$, 12), 167 ($[C_{12}H_9N]^+$, 12), 166 ($[C_{12}H_9N]^{2+}$, 11). IR $\tilde{\nu}$ [cm^{-1}] = 3645 (w), 3084 (w), 3063 (w), 3051 (w), 3034 (w), 2990 (w), 2889 (w), 2855 (w), 2236 (w), 1585 (m), 1574 (m), 1558 (w), 1487 (m), 1450 (m), 1435 (m), 1398 (w), 1333 (w), 1310 (w), 1288 (m), 1265 (m), 1238 (w), 1204 (w), 1153 (w), 1115 (w), 1076 (w), 1034 (w), 989 (w), 814 (m), 764 (m), 750 (s), 737 (m), 692 (s), 654 (w), 623 (m). Anal. calcd. for $C_{24}H_{17}N_3$ (347.42): C 82.97, H 4.93, N 12.10; Found C 82.75, H 4.82, N 11.94.

6-(2′-(Diphenylamino)-[1,1′-biphenyl]-4-yl)picolinonitrile (6b)

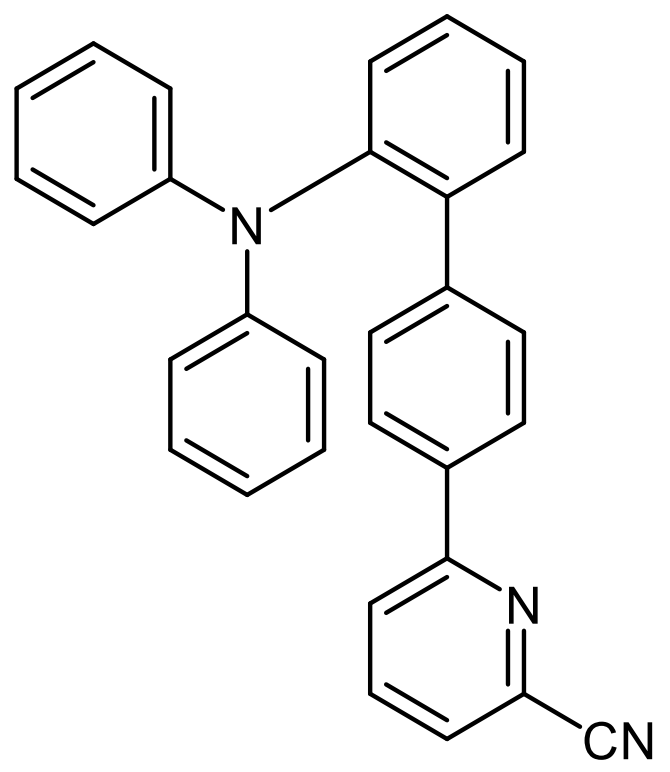

6b

$C_{30}H_{21}N_3$

[423.52]

The synthesis was performed by GP2. After purification by flash column chromatography on silica gel (*n*-hexane/ethyl acetate 10:1) compound **6b** (174 mg, 0.41 mmol, 82%) was isolated as a colorless solid, Mp 157 °C. R_f = 0.19 (*n*-hexane/ethyl acetate 10:1). ^{1}H NMR (300 MHz, acetone-d$_6$): δ = 8.18 (dd, ^3J = 8.3 Hz, ^4J = 1.1 Hz, 1H), 8.09 (t, ^3J = 7.9 Hz, 1H), 7.95 (d, ^3J = 8.4 Hz, 2H), 7.84 (dd, ^3J = 7.5 Hz, ^4J = 1.1 Hz, 1H), 7.51–7.35 (m, 5H), 7.30 (dd, ^3J = 7.7 Hz, ^4J = 1.9 Hz, 1H), 7.14–7.06 (m, 4H), 6.89–6.79 (m, 6H). ^{13}C NMR (75 MHz, acetone-d$_6$): δ = 159.0 (C$_{quat}$), 148.5 (C$_{quat}$), 145.8 (C$_{quat}$), 142.6 (C$_{quat}$), 140.4 (C$_{quat}$), 139.5 (CH), 136.5 (C$_{quat}$), 134.3 (C$_{quat}$), 132.6 (CH), 130.5 (CH), 130.2 (CH), 130.0 (CH), 129.7 (CH), 127.8 (CH), 127.3 (CH), 127.0 (CH), 124.5 (CH), 122.9 (CH), 122.5 (CH), 118.3 (C$_{quat}$). EI MS (70 eV, *m*/*z* (%)) 424 (30), 423 ([M]$^+$, 100), 422 (12), 212 (13), 103 ([$C_6H_3N_2$]$^+$, 10). IR $\tilde{\nu}$ [cm^{-1}] = 3970 (w), 3065 (w), 1585 (m), 1564 (w), 1551 (w), 1483 (m), 1447 (m), 1420 (w), 1327 (w), 1312 (w), 1290 (w), 1273 (m), 1238 (w), 1206 (w), 1167 (w), 989 (w), 810 (m), 754 (s), 735 (w), 694 (m), 673 (w), 650 (w), 638 (w), 623 (w). Anal. calcd. for $C_{30}H_{21}N_3$ (423.52) C 85.08, H 5.00, N 9.92; Found C 84.89, H 5.02, N 9.67.

6-(2′-(Diphenylamino)-3-methyl-[1,1′-biphenyl]-4-yl)picolinonitrile (6c)

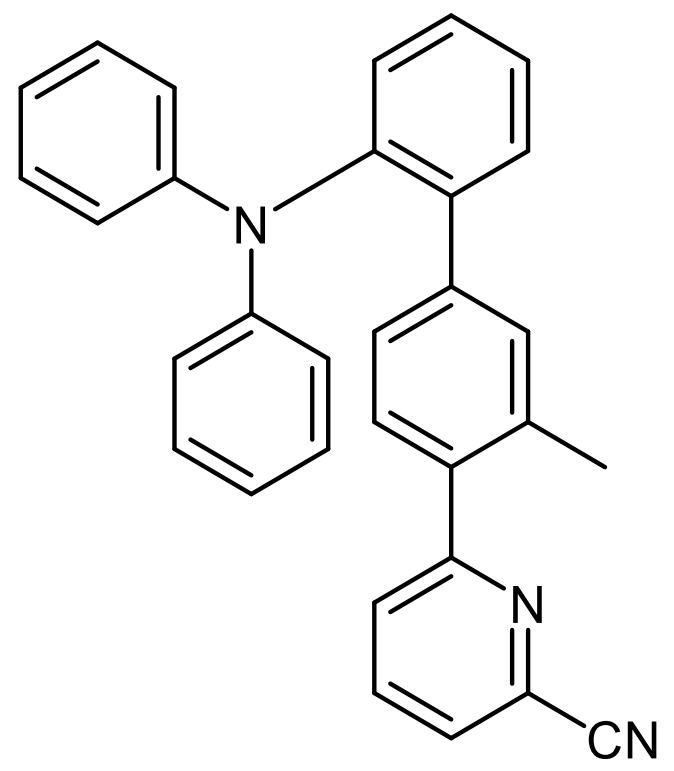

6c

$C_{31}H_{23}N_3$

[437.55]

The synthesis was performed by GP2. After purification by flash column chromatography on silica gel (*n*-hexane/ethyl acetate 10:1) compound **6c** (170 mg, 0.34 mmol, 77%) was isolated as a colourless solid, Mp 168 °C. R_f = 0.20 (*n*-hexane/ethyl acetate 10:1). ^{1}H NMR (300 MHz, aceton-d$_6$): δ = 8.10 (t, ^3J = 7.9 Hz, 1H), 7.88 (dd, ^3J = 7.7 Hz, ^4J = 1.0 Hz, 1H), 7.76 (dd, ^3J = 8.0 Hz, ^4J = 1.0 Hz, 1H), 7.45 (ddd, ^3J = 7.6 Hz, ^3J = 6.9 Hz, ^4J = 1.4 Hz, 2H), 7.40–7.34 (m, 1H), 7.30 (dd, ^3J = 8.1 Hz, ^4J = 1.5 Hz, 1H), 7.26 (d, ^3J = 7.9 Hz, 1H), 7.19 (dd, ^3J = 8.0 Hz, ^4J = 1.8 Hz, 1H), 7.16 – 7.08 (m, 5H), 6.88–6.81 (m, 6H), 2.22 (s, 3H). ^{13}C NMR (75 MHz, aceton-d$_6$): δ = 162.1 (C$_{quat}$), 148.6 (C$_{quat}$), 145.9 (C$_{quat}$), 141.5 (C$_{quat}$), 140.7 (C$_{quat}$), 138.9 (CH), 137.8 (C$_{quat}$), 136.2 (C$_{quat}$), 133.8 (C$_{quat}$), 132.5 (CH), 132.2 (CH), 130.4 (CH), 130.1 (CH), 130.0 (CH), 129.6 (CH), 128.6 (CH), 127.1 (CH), 126.9 (CH), 122.9 (CH), 122.4 (CH), 118.3 (C$_{quat}$), 20.5 (CH$_3$). EI MS (70 eV, *m*/*z* (%)) 438 (31), 437 ([M]$^+$, 100), 436 (14), 218 (16), 167 ([$C_{12}H_9N$]$^+$, 9), 166 ([$C_{12}H_9N$]$^{2+}$, 11). IR $\tilde{\nu}$ [cm^{-1}] = 3022 (w), 3007 (w), 2920 (w), 2369 (w), 2232 (w), 1607 (w), 1585 (m), 1558 (w), 1483 (m), 1449 (m), 1437 (m), 1420 (w), 1387 (w), 1375 (w), 1327 (w), 1312 (w), 1294 (m), 1269 (m), 1254 (w), 1204 (w), 1155 (w), 1130 (w), 1115 (w), 1080 (w), 1053 (w), 1028 (w), 980 (w), 920 (w), 889 (w), 868 (w), 833 (w), 808 (m), 754 (s), 727 (w), 696 (s), 673 (m), 662

(w), 625 (m), 611 (m). Anal. calcd. for $C_{31}H_{23}N_3$ (437.55): C 85.10, H 5.30, N 9.60; Found C 84.94, H 5.40, N 9.44.

2′-(Diphenylamino)-[1,1′-biphenyl]-2-carbonitrile (7a)

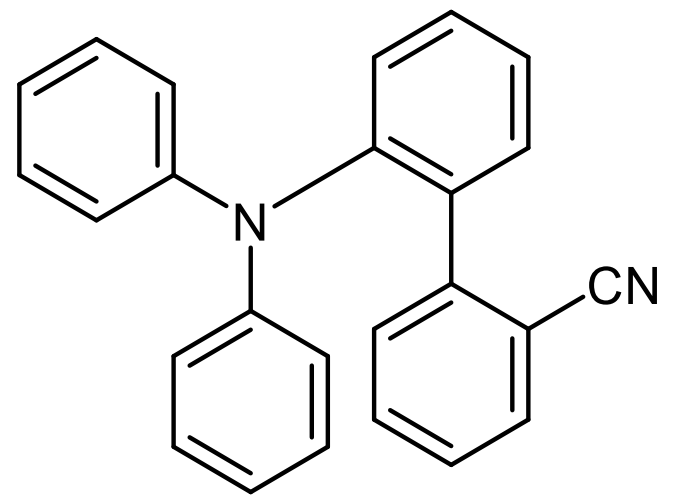

7a

$C_{25}H_{18}N_2$

[346.43]

The synthesis was performed by GP2. After purification by flash column chromatography on silica gel (*n*-hexane/ethyl acetate 10:1) compound **7a** (69.6 mg, 0.20 mmol, 57%) was isolated as a colorless solid, Mp 90 °C. R_f = 0.51 (*n*-hexane/ethyl acetate 10:1). ^{1}H NMR (300 MHz, acetone-d$_6$): δ = 7.53–7.42 (m, 3H), 7.41–7.25 (m, 5H), 7.15–7.06 (m, 4H), 6.92–6.79 (m, 6H). ^{13}C NMR (75 MHz, acetone-d$_6$): δ = 148.5 (C_{quat}), 147.0 (C_{quat}), 144.4 (C_{quat}), 136.8 (C_{quat}), 133.5 (CH), 132.9 (CH), 132.8 (CH), 131.4 (CH), 131.0 (CH), 129.8 (CH), 128.9 (CH), 128.3 (CH), 126.0 (CH), 123.6 (CH), 123.0 (CH), 118.8 (C_{quat}), 113.4 (C_{quat}). EI MS (70 eV, *m*/*z* (%)): 347 ([M]$^+$, 27), 346 ([M]$^+$, 100), 345 (27), 172 (10), 167 ([$C_{12}H_9N$]$^+$, 9), 166 ([$C_{12}H_9N$]$^{2+}$, 10). IR $\tilde{\nu}$ [cm^{-1}] = 3057 (w), 3034 (w), 2920 (w), 2853 (w), 2286 (w), 2226 (w), 1950 (w), 1927 (w), 1587 (m), 1489 (m), 1470 (m), 1431 (m), 1325 (m), 1314 (m), 1294 (m), 1269 (m), 1242 (w), 1175 (m), 1152 (w), 1109 (w), 1078 (w), 1047 (w), 1026 (w), 991 (w), 949 (w), 920 (w), 897 (w), 880 (w), 837 (w), 754 (s), 739 (m), 727 (w), 696 (s), 664 (w), 625 (m). Anal. calcd. for $C_{25}H_{18}N_2$ (346.43): C 86.88, H 5.24, N 8.09; Found: C 86.61, H 5.26, N 7.86.

2″-(Diphenylamino)-[1,1′:4′,1″-terphenyl]-2-carbonitrile (7b)

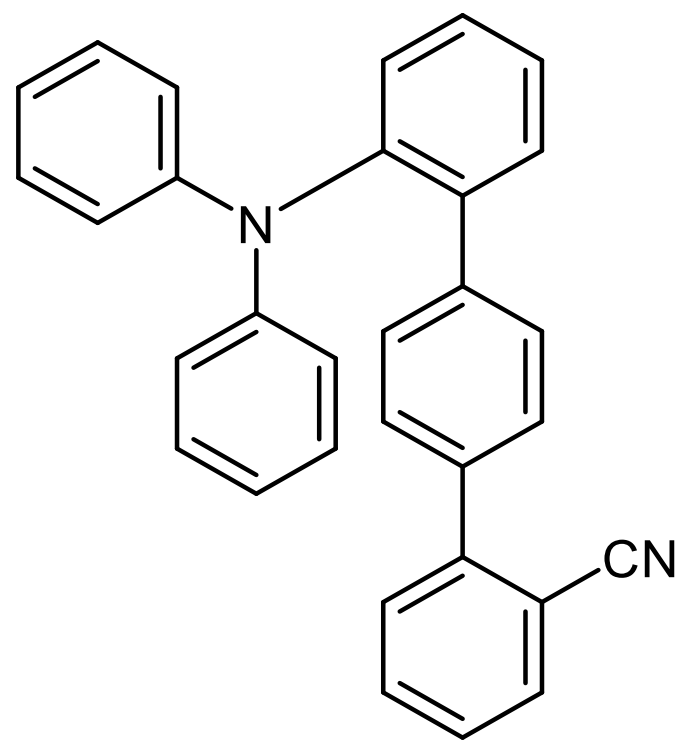

7b

$C_{31}H_{22}N_2$

[422.53]

The synthesis was performed by GP2. After purification by flash column chromatography on silica gel (*n*-hexane/ethyl acetate 10:1) compound **7b** (150 mg, 0.36 mmol, 77%) was isolated as a colorless solid, Mp 164 °C. R_f = 0.34 (*n*-hexane/ethyl acetate 10:1). ^{1}H NMR (300 MHz, acetone-d$_6$): δ = 7.86 (dd, ^3J =

7.8 Hz, ^{4}J = 1.2 Hz, 1H), 7.75 (td, ^{3}J = 7.7 Hz, ^{4}J = 1.4 Hz, 1H), 7.59–7.51 (m, 2H), 7.51–7.43 (m, 2H), 7.41–7.29 (m, 6H), 7.15–7.07 (m, 4H), 6.88–6.79 (m, 6H). ^{13}C NMR (75 MHz, acetone-d$_6$): δ = 148.5 (C$_{quat}$), 146.1 (C$_{quat}$), 145.8 (C$_{quat}$), 141.2 (C$_{quat}$), 140.7 (C$_{quat}$), 137.7 (C$_{quat}$), 134.6 (CH), 134.0 (CH), 132.5 (CH), 131.0 (CH), 130.2 (CH), 130.1 (CH), 129.7 (CH), 129.2 (CH), 128.8 (CH), 126.9 (CH), 123.0 (CH), 122.4 (CH), 119.0 (C$_{quat}$), 111.9 (C$_{quat}$). EI MS (70 eV, *m*/*z* (%)) 423 ([M]$^+$, 28), 422 ([M]$^+$, 100), 421 (25), 211 (10), 167 ([$C_{12}H_9N$]$^+$, 8). IR $\tilde{\nu}$ [cm^{-1}] = 3063 (w), 3032 (w), 2357 (w), 2326 (w), 2220 (w), 1585 (m), 1578 (w), 1485 (m), 1476 (m), 1439 (w), 1396 (w), 1317 (m), 1296 (m), 1277 (m), 1242 (w), 1188 (w), 1171 (w), 1155 (w), 1115 (w), 1101 (w), 1076 (w), 1028 (w), 1009 (w), 995 (w), 957 (w), 841 (m), 741 (m), 698 (m), 675 (w), 640 (w), 621 (m). Anal. calcd. for $C_{31}H_{22}N_2$ (422.53): C 88.12, H 5.25, N 6.63; Found C 87.95, H 5.06, N 6.55.

2″-(Diphenylamino)-2′ -methyl-[1,1′:4′,1″-terphenyl]-2-carbonitrile (7c)

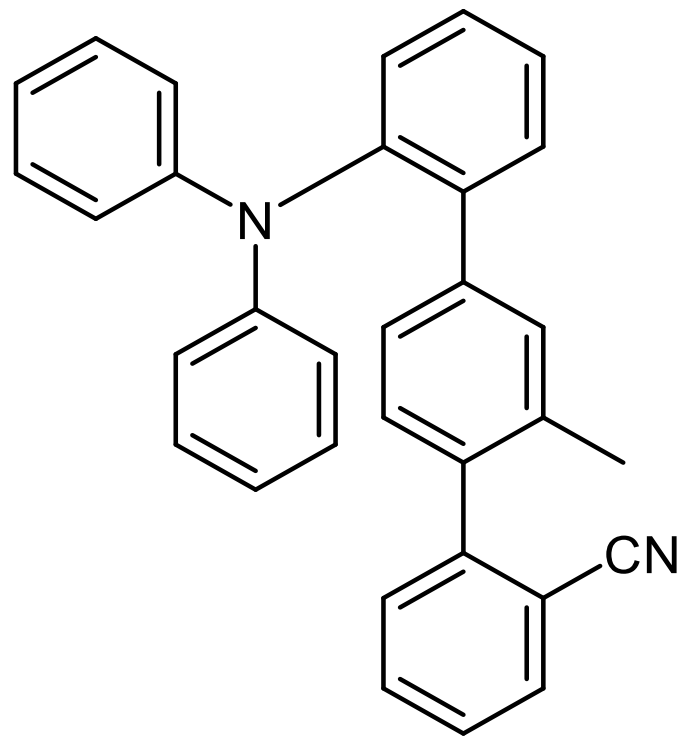

7c

$C_{32}H_{24}N_2$

[436.56]

The synthesis was performed by GP2. After purification by flash column chromatography on silica gel (*n*-hexane/ethyl acetate 10:1) compound **7c** (130 mg, 0.31 mmol, 73%) was isolated as a colorless solid, Mp 121 °C. R_f = 0.38 (*n*-hexane/ethyl acetate 10:1). ^{1}H NMR (300 MHz, acetone-d$_6$): δ = 7.86 (dd, ^{3}J = 8.1 Hz, ^{4}J = 1.1 Hz, 1H), 7.75 (td, ^{3}J = 7.7 Hz, ^{4}J = 1.4 Hz, 1H), 7.57 (td, ^{3}J = 7.7 Hz, ^{4}J = 1.3 Hz, 1H), 7.49–7.40 (m, 2H), 7.39–7.32 (m, 3H), 7.18–7.08 (m, 5H), 7.06–7.03 (m, 1H), 7.02 (d, ^{3}J = 7.8 Hz, 1H), 6.88–6.80 (m, 6H), 1.99 (s, 3H). ^{13}C NMR (75 MHz, acetone-d$_6$): δ = 148.6 (C$_{quat}$), 146.1 (C$_{quat}$), 141.2 (C$_{quat}$), 141.1 (C$_{quat}$), 137.7 (C$_{quat}$), 135.8 (C$_{quat}$), 133.8 (CH), 133.6 (CH), 132.4 (CH), 131.6 (CH), 131.5 (CH), 130.2 (CH), 129.9 (CH),129.8 (CH), 129.7 (CH), 128.8 (CH) 126.8 (CH), 123.0 (CH), 122.3 (CH), 118.6 (C$_{quat}$), 113.5 (C$_{quat}$), 19.9 (CH_3). EI MS (70 eV, *m*/*z* (%)) 437 (33), 436 ([M]$^+$, 100), 435 (19). IR $\tilde{\nu}$ [cm^{-1}] = 3084 (w), 3021 (w), 3003 (w), 2924 (w), 2864 (w), 2226 (w), 1585 (m), 1558 (w), 1489 (m), 1476 (m), 1441 (m), 1396 (w), 1323 (m), 1310 (m), 1277 (m), 1236 (w), 1111 (w), 1076 (w), 1032 (w), 957 (w), 880 (m), 868 (m), 824 (w), 746 (s), 692 (s), 677 (w), 667 (w), 629 (w), 611 (m). Anal. calcd. for $C_{32}H_{24}N_2$ (436.56): C 88.04, H 5.54, N 6.42; Found C 88.02, H 5.51, N 6.47.

3 ^{1}H and ^{13}C NMR spectra

2-Bromo-*N*,*N*-diphenylaniline (1)

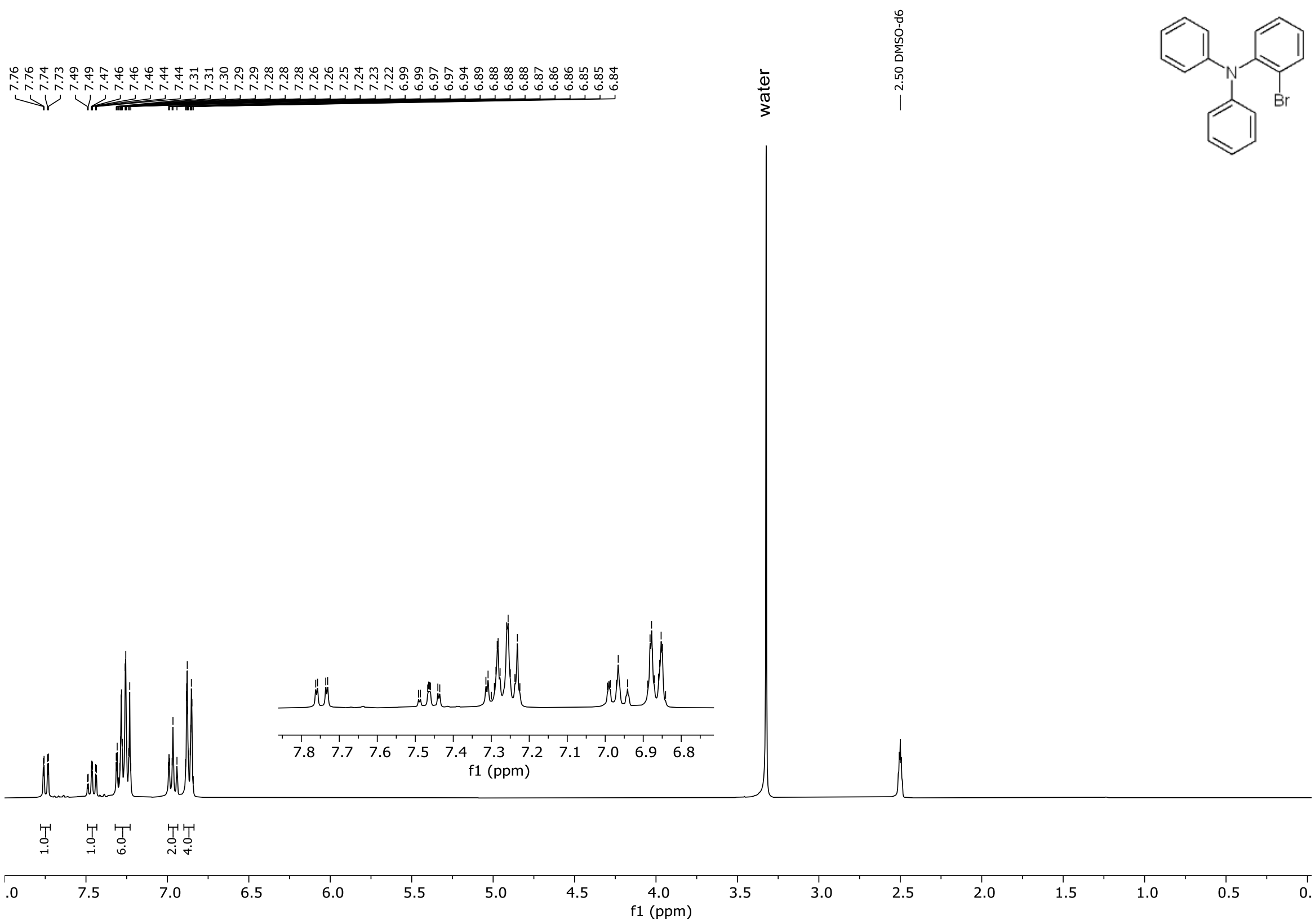

Figure S1. ^{1}H NMR spectrum of compound **1** (300 MHz, DMSO-d$_6$, 298 K).

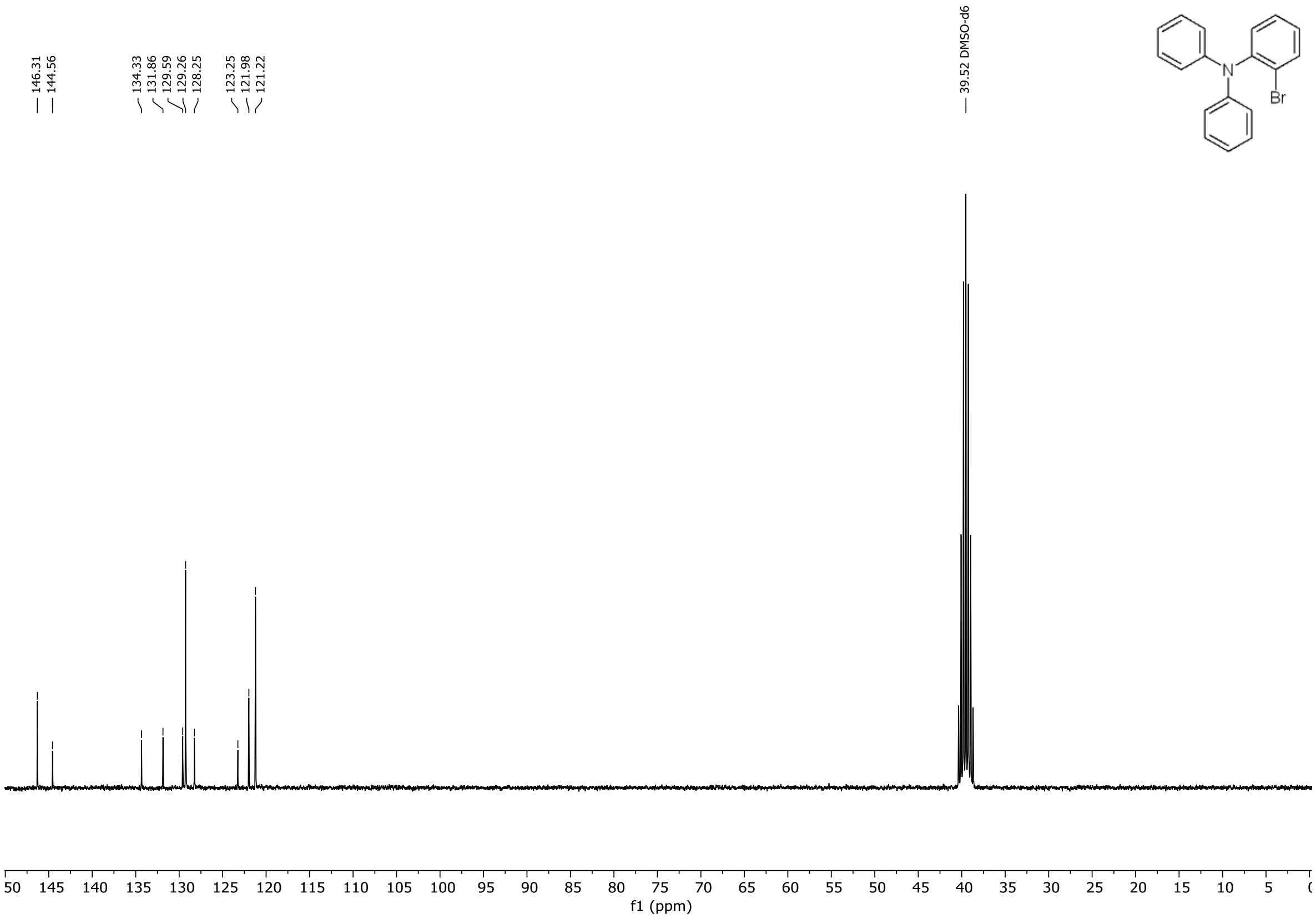

Figure S2. ^{13}C NMR spectrum of compound **1** (75 MHz, DMSO-d$_6$, 298 K).

4'-Bromo-*N*,*N*-diphenyl-[1,1'-biphenyl]-2-amine (3a)

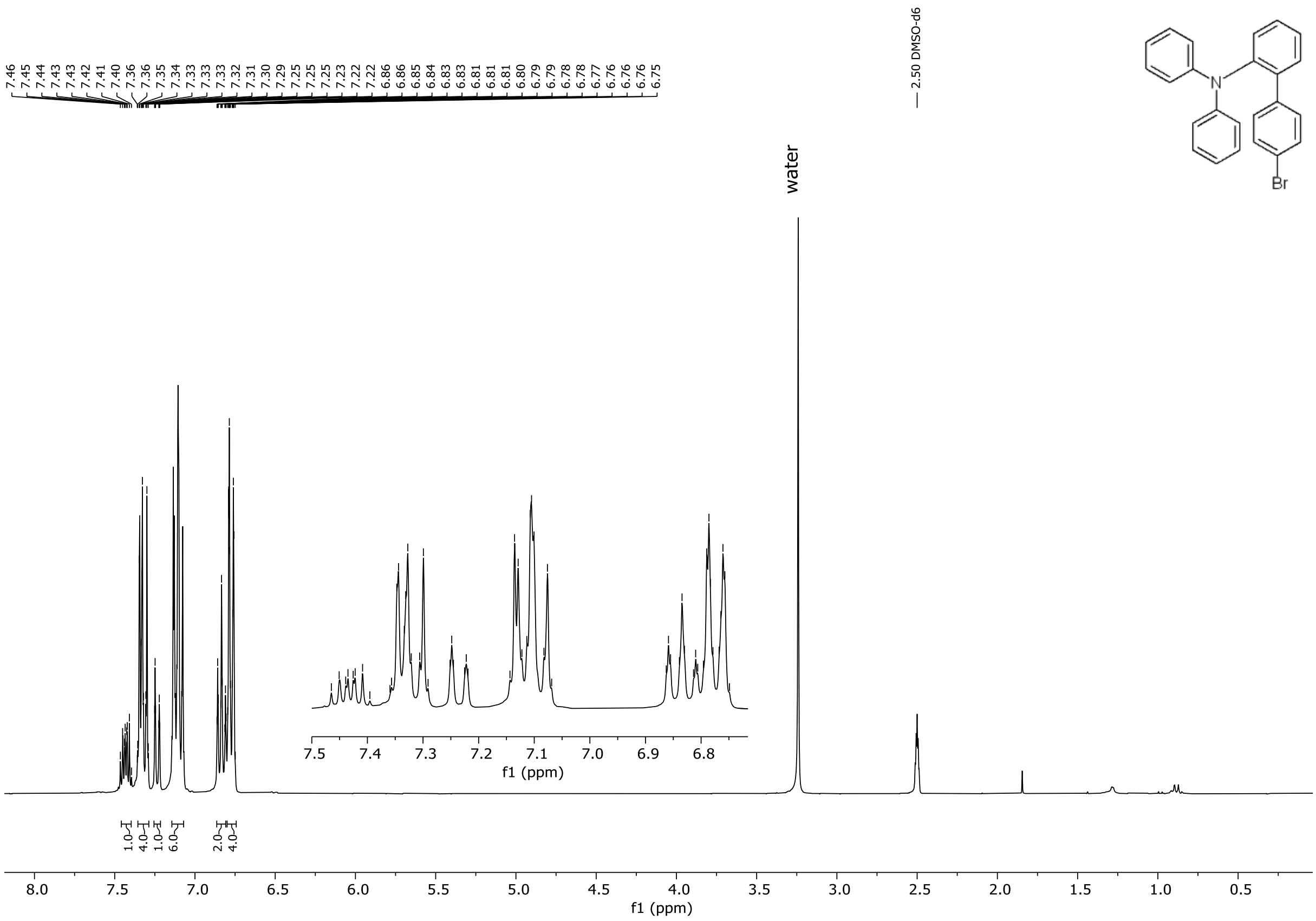

Figure S3. ^{1}H NMR spectrum of compound **3a** (300 MHz, DMSO-d_6, 298 K).

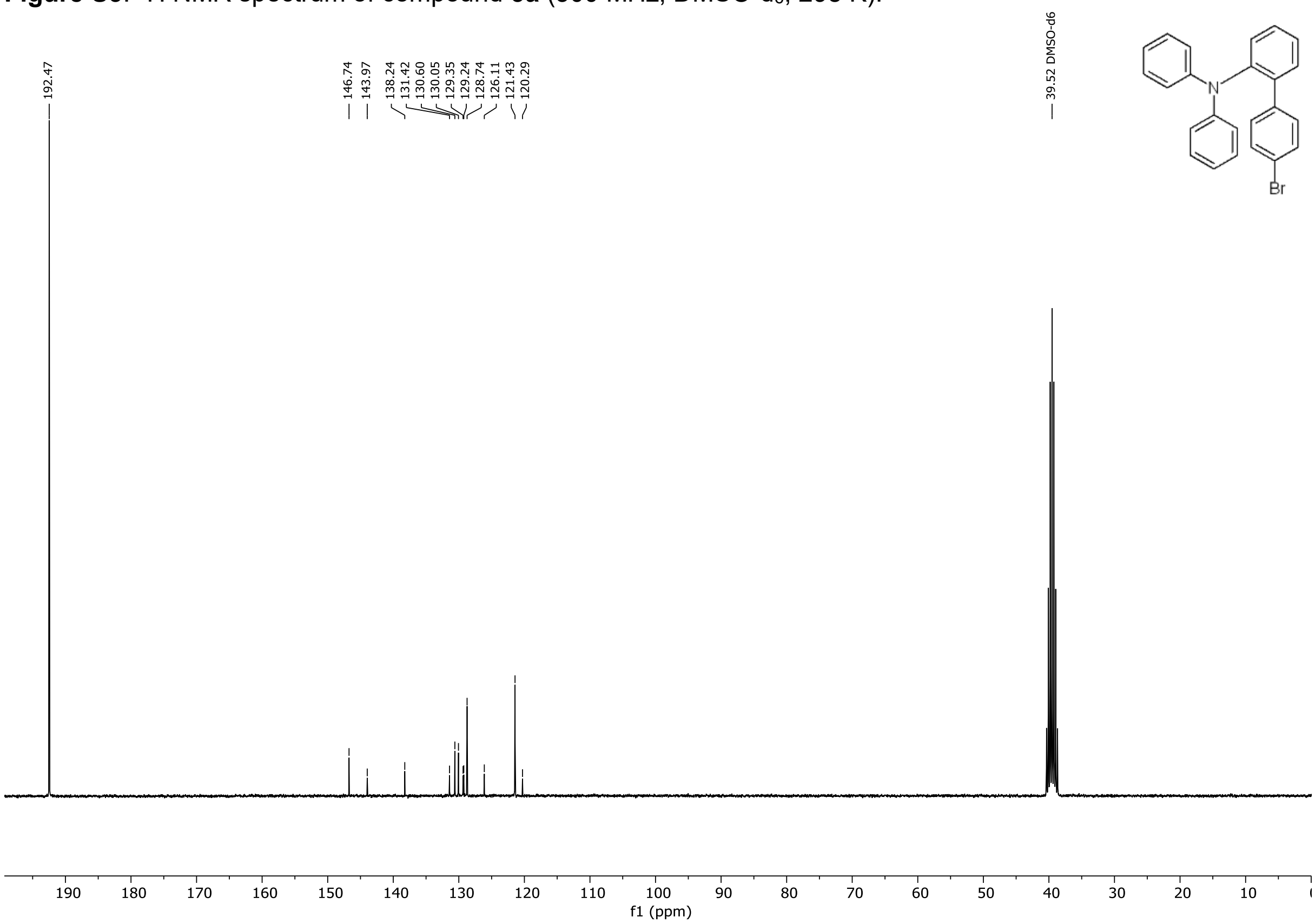

Figure S4. ^{13}C NMR spectrum of compound **3a** (75 MHz, DMSO-d_6, 298 K).

4′-Brom-3′-methyl-*N,N*- diphenyl-(1,1′-biphenyl)-2-amine (3b)

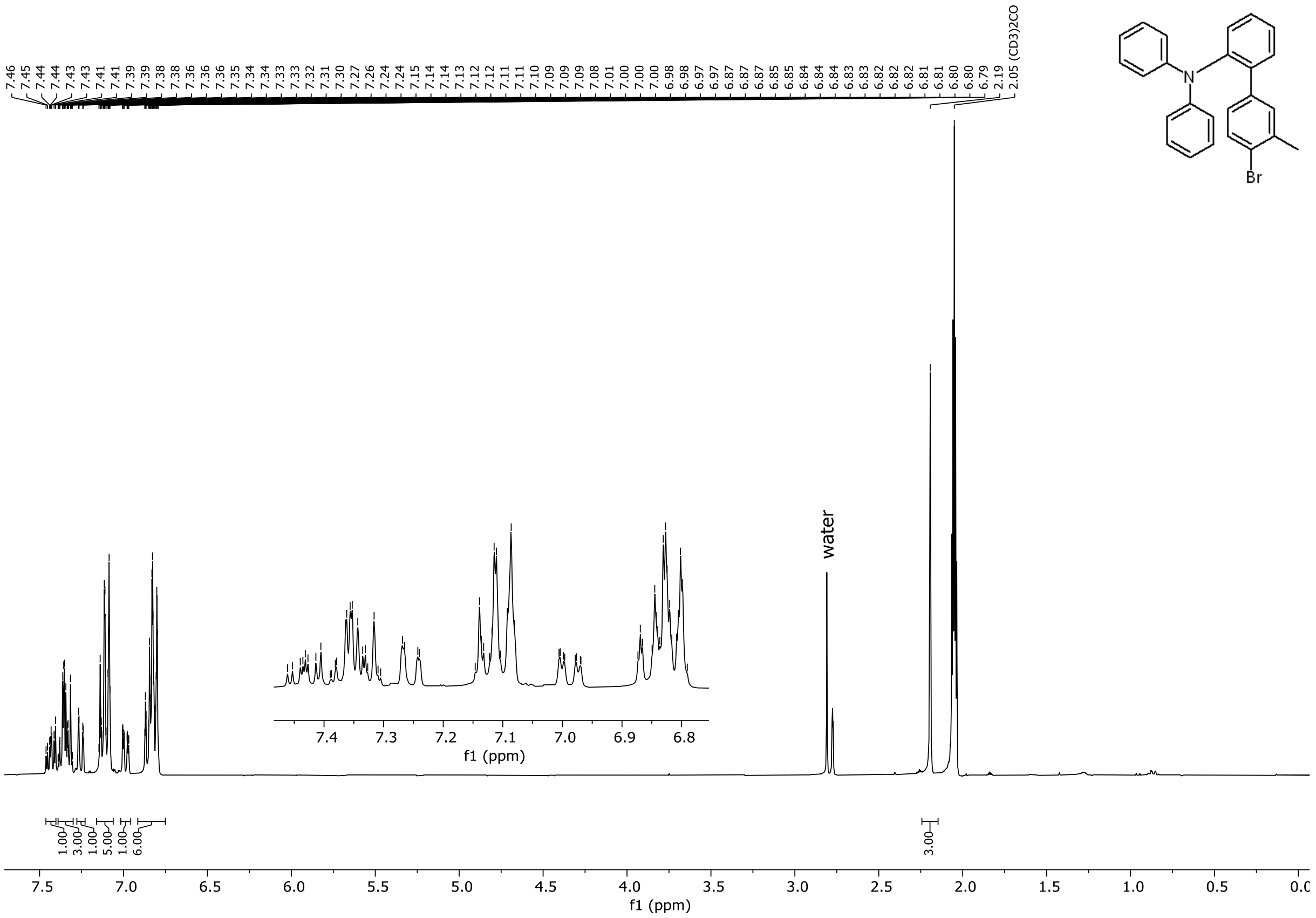

Figure S5. ^{1}H NMR spectrum of compound **3b** (300 MHz, acetone-d_6, 298 K).

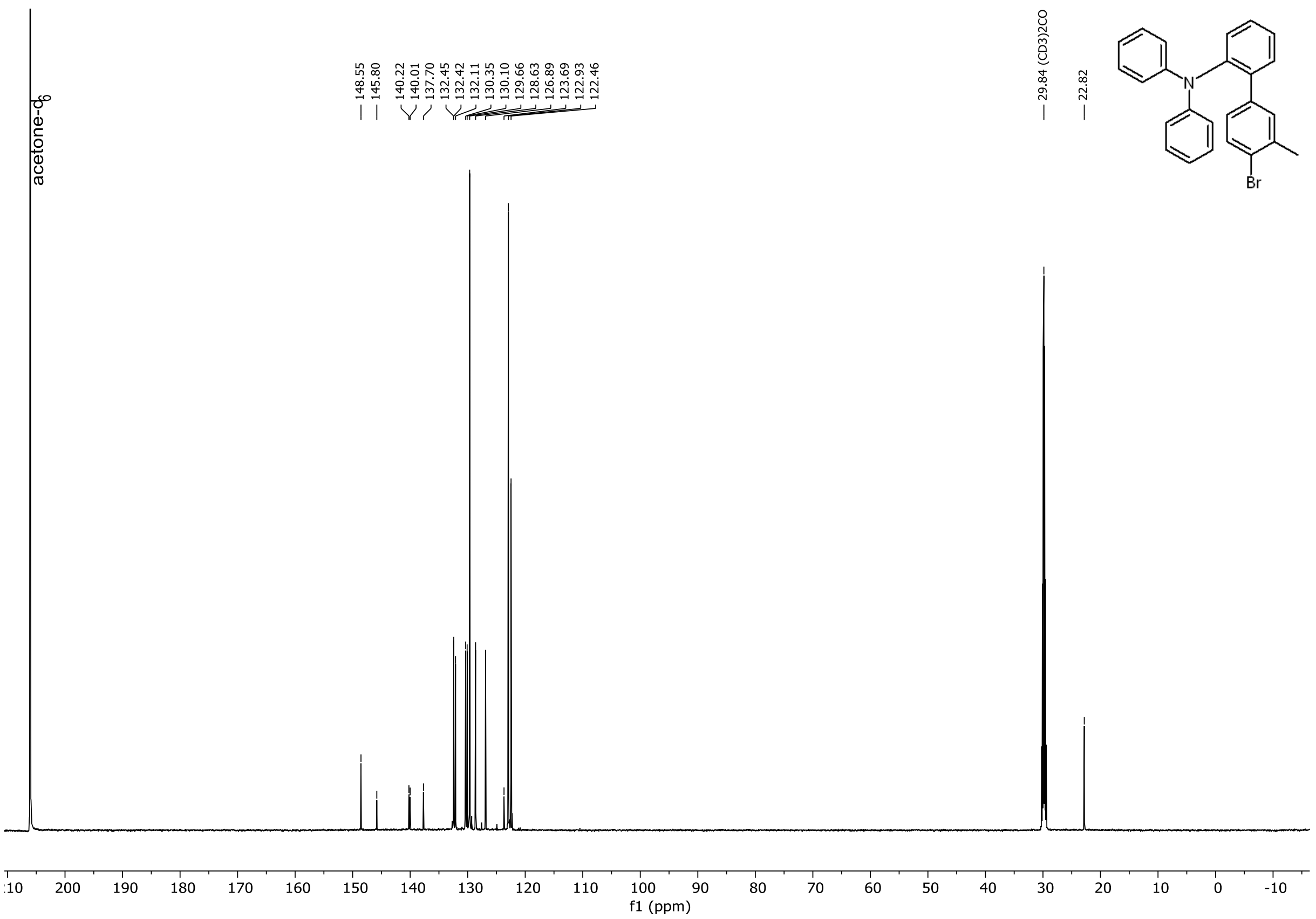

Figure S6. ^{13}C NMR spectrum of compound **3b** (151 MHz, acetone-d_6, 305 K).

6-(2-(Diphenylamino)phenyl)picolinonitrile (6a)

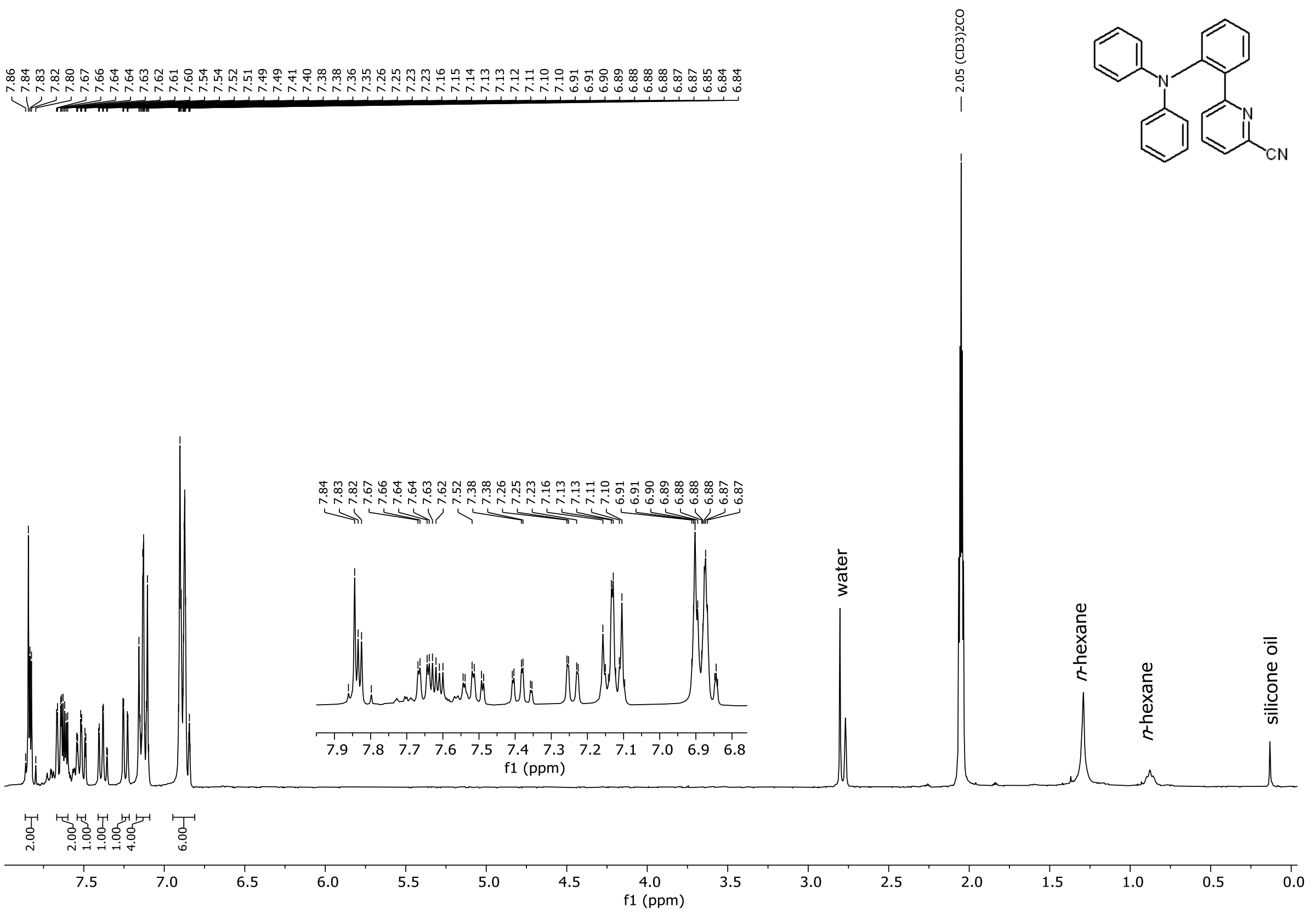

Figure S7. ^{1}H NMR spectrum of compound **6a** (300 MHz, acetone-d_6, 299 K).

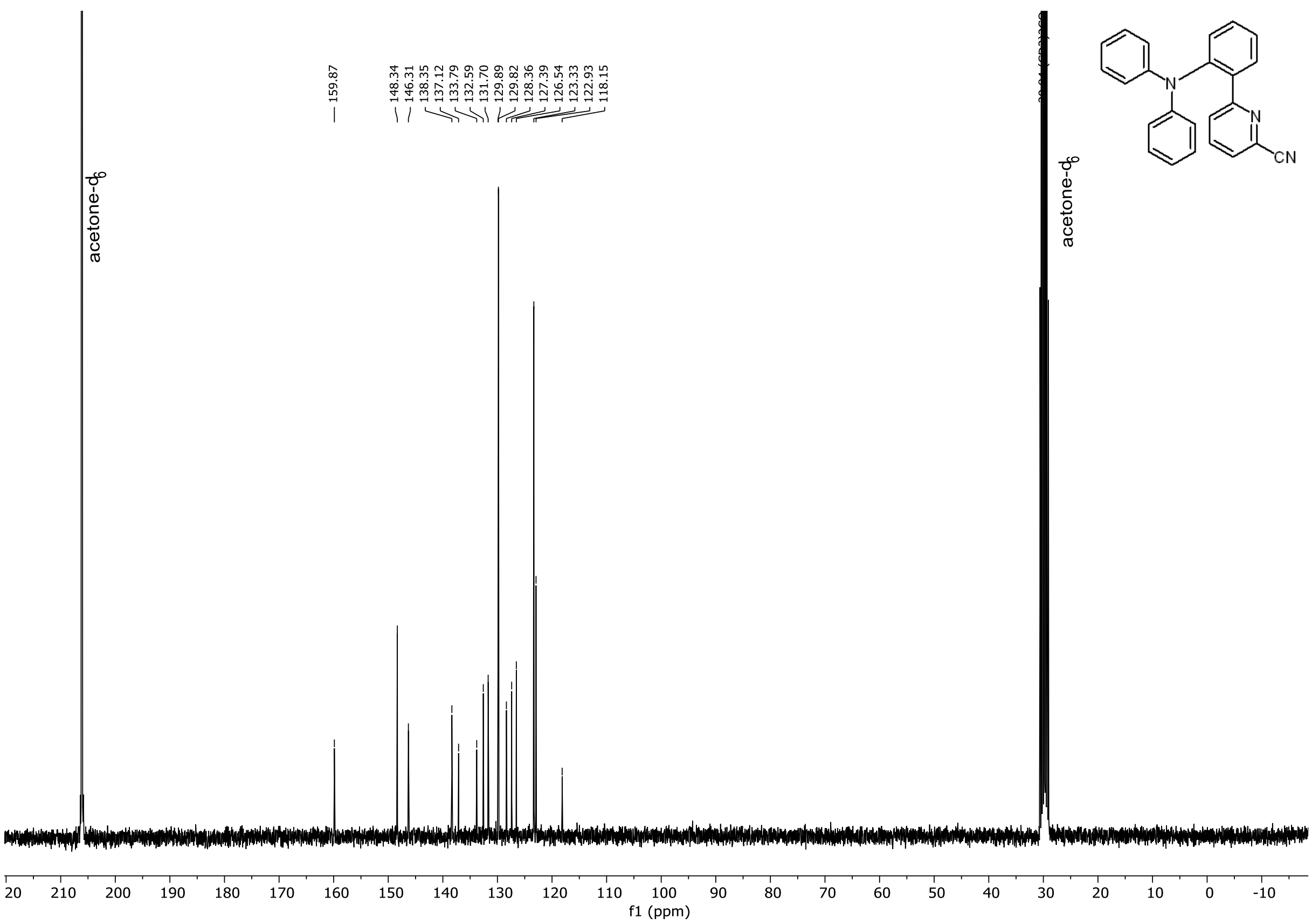

Figure S8. ^{13}C NMR spectrum of compound **6a** (75 MHz, acetone-d_6, 299 K).

6-(2′-(Diphenylamino)-[1,1′-biphenyl]-4-yl)picolinonitrile (6b)

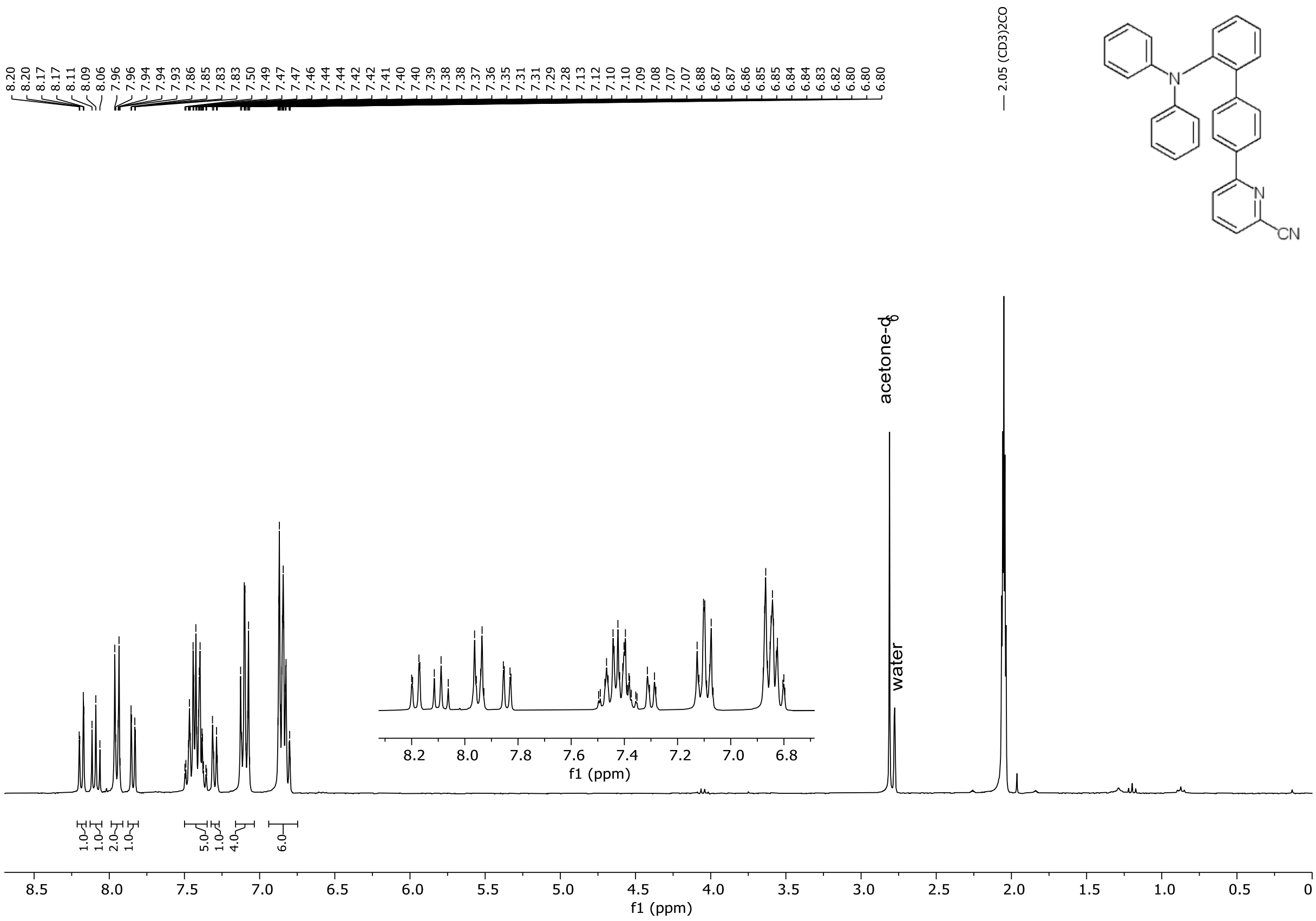

Figure S9. ^{1}H NMR spectrum of compound **6b** (300 MHz, acetone-d6, 299 K).

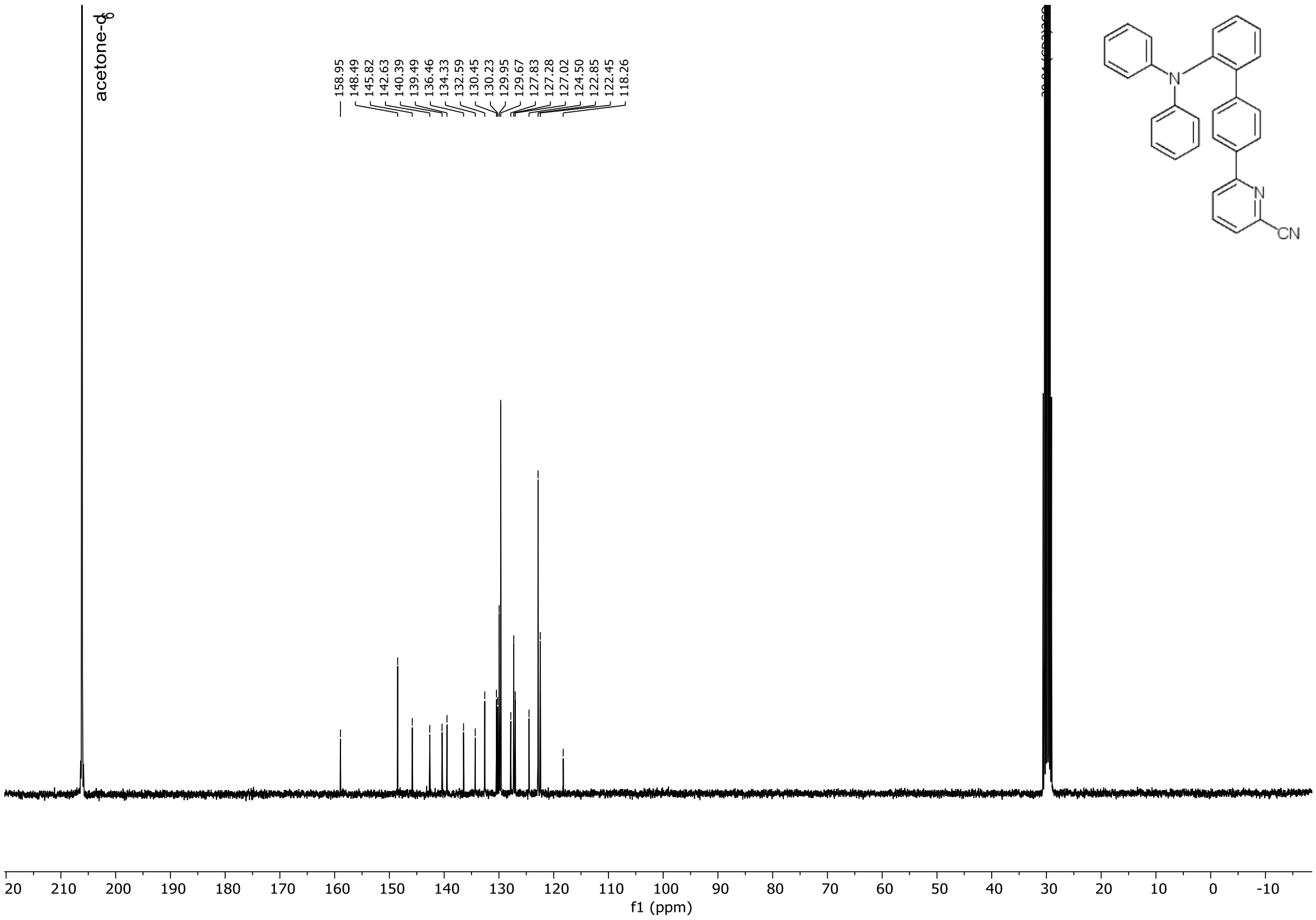

Figure S10. ^{13}C NMR spectrum of compound **6b** (75 MHz, acetone-d6, 299 K).

6-(2′-(Diphenylamino)-3-methyl-[1,1′-biphenyl]-4-yl)picolinonitrile (6c)

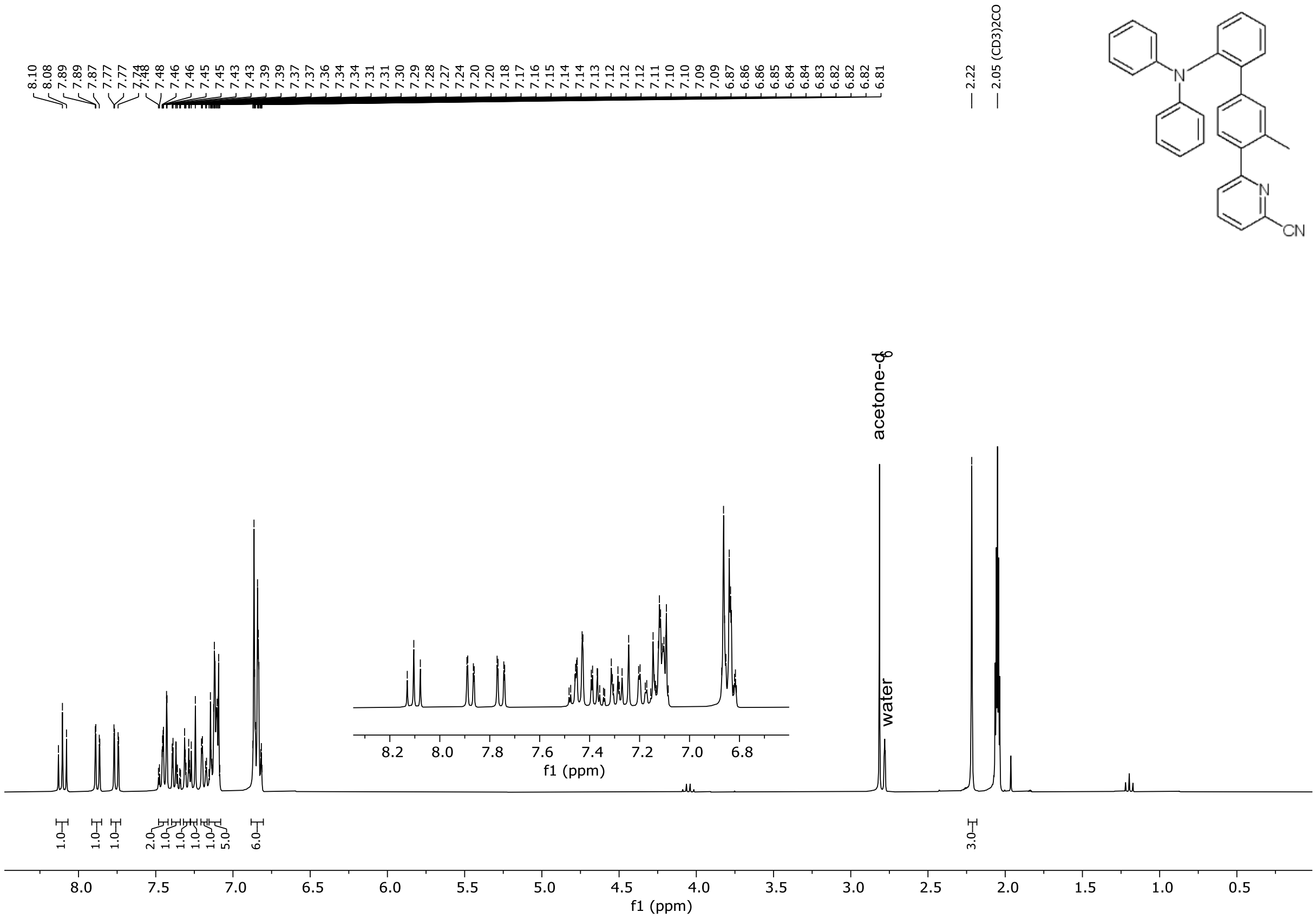

Figure S11. ^{1}H NMR spectrum of compound **6c** (300 MHz, acetone-d6, 298 K).

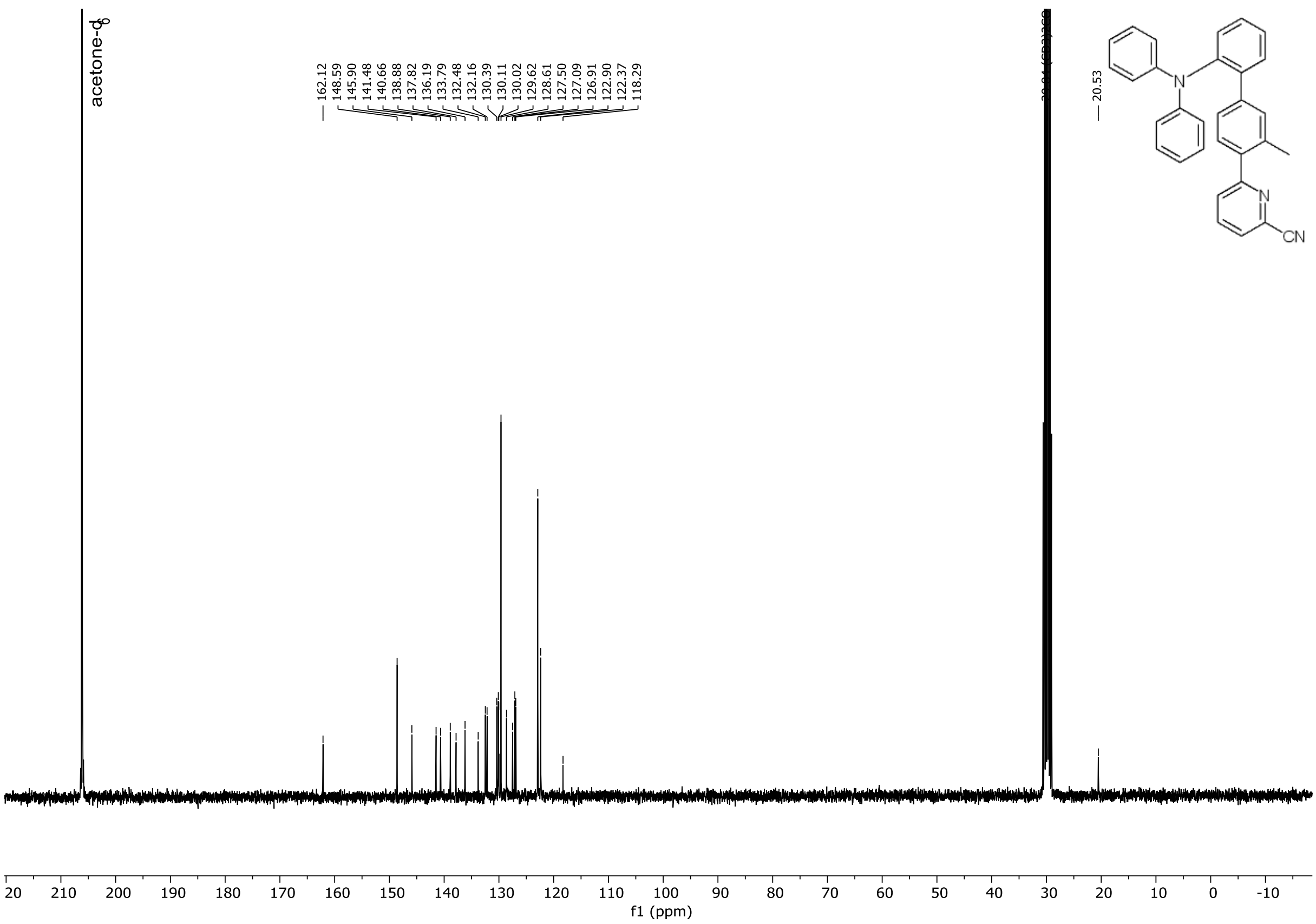

Figure S12. ^{13}C NMR spectrum of compound **6c** (75 MHz, acetone-d6, 298 K).

2′-(Diphenylamino)-[1,1′-biphenyl]-2-carbonitrile (7a)

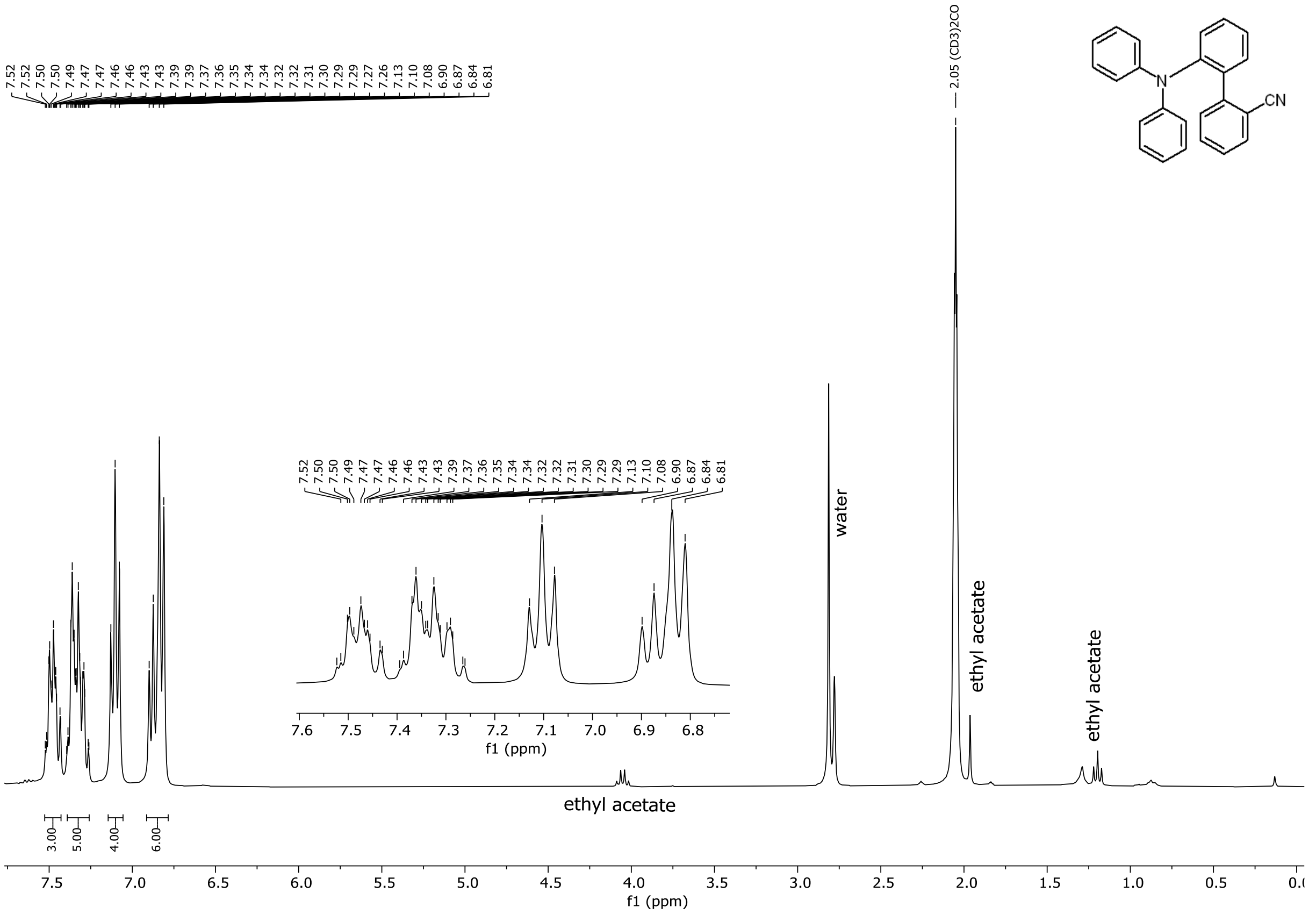

Figure S13. ^{1}H NMR spectrum of compound **7a** (300 MHz, acetone-d$_6$, 298 K).

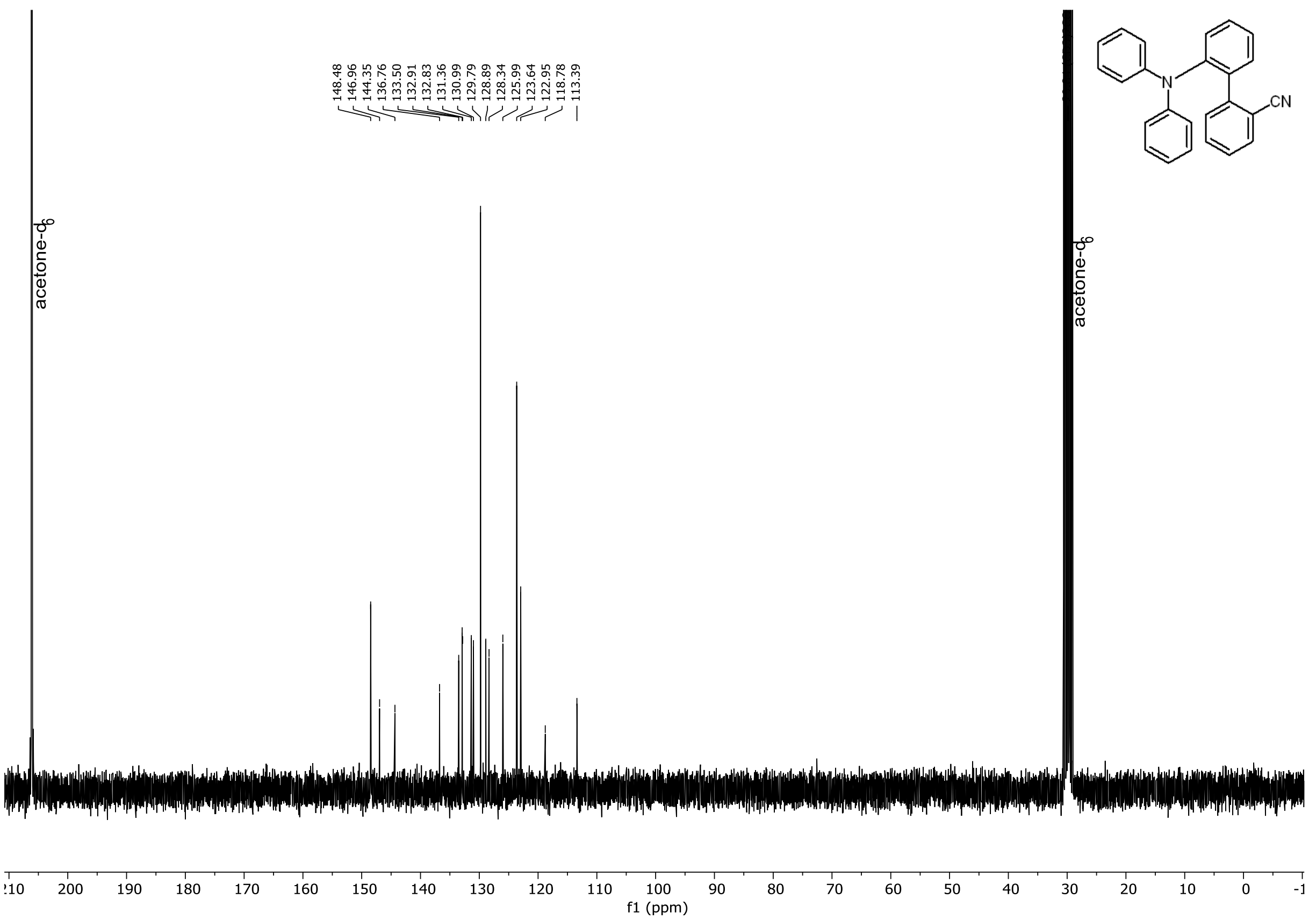

Figure S14. ^{13}C NMR spectrum of compound **7a** (75 MHz, acetone-d$_6$, 298 K).

2″-(Diphenylamino)-[1,1′:4′,1″-terphenyl]-2-carbonitrile (7b)

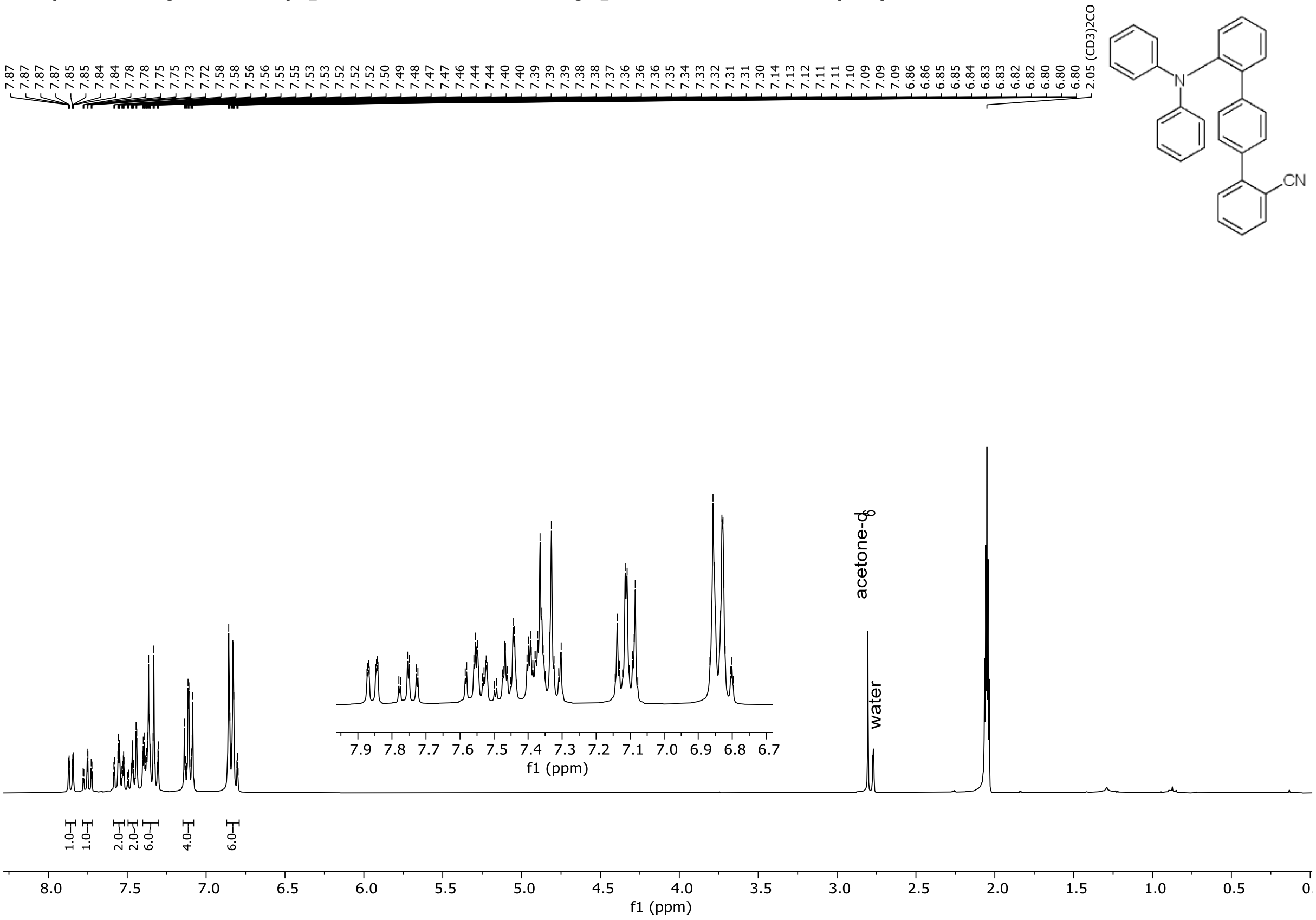

Figure S15. ^{1}H NMR spectrum of compound **7b** (300 MHz, acetone-d6, 299 K).

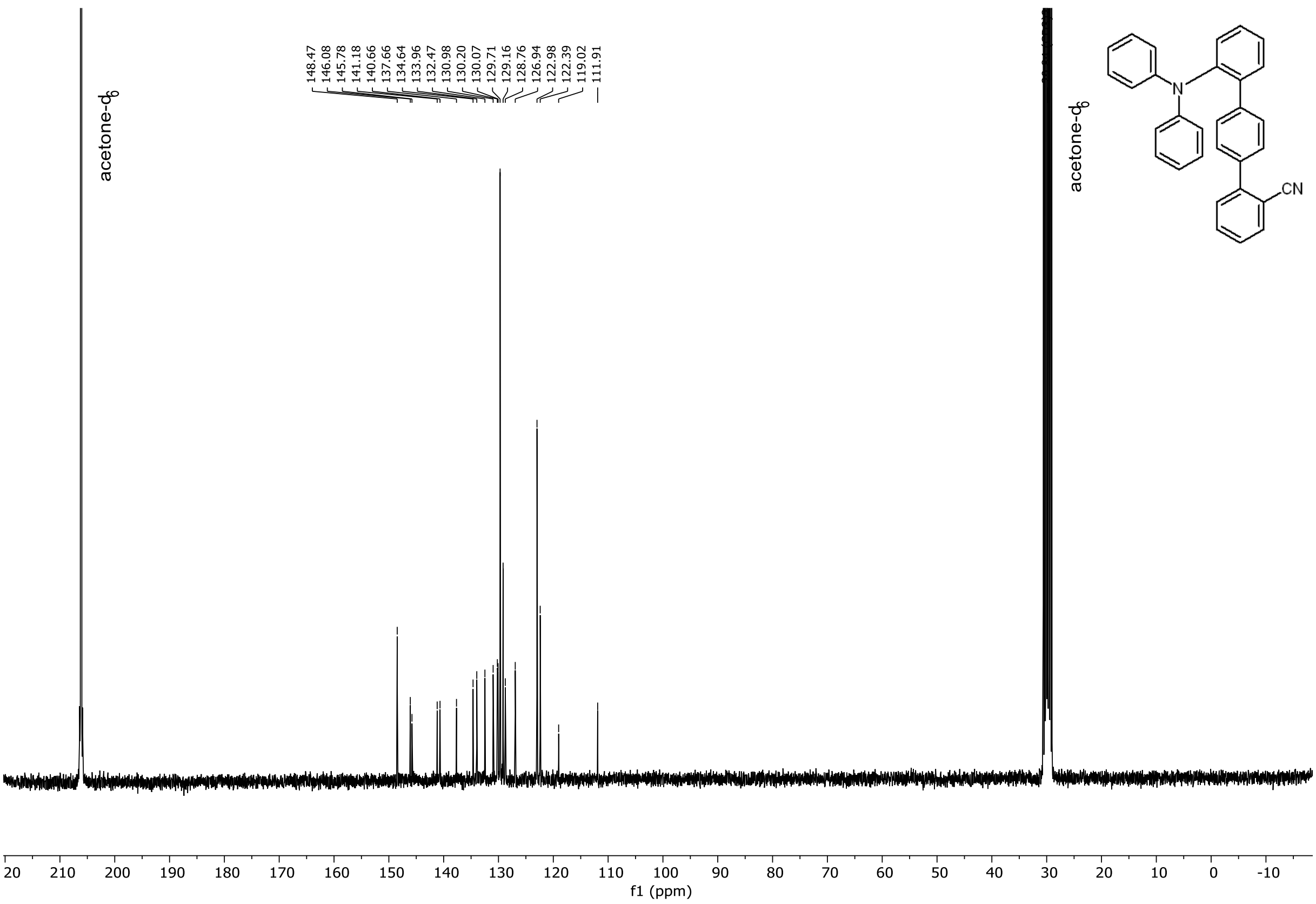

Figure S16. ^{13}C NMR spectrum of compound **7b** (75 MHz, acetone-d6, 299 K).

2″-(Diphenylamino)-2′ -methyl-[1,1′:4′,1″-terphenyl]-2-carbonitrile (7c)

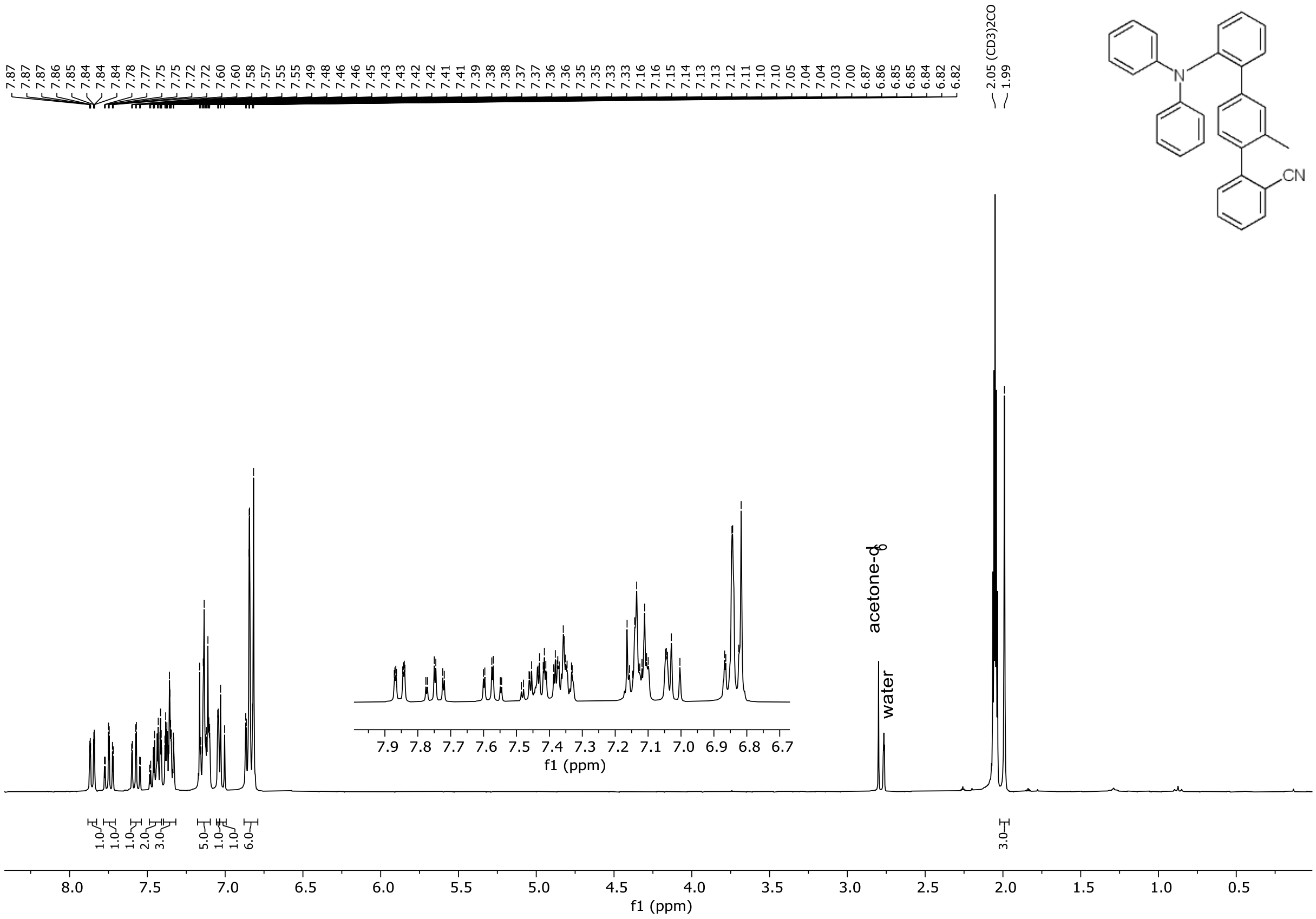

Figure S17. ^{1}H NMR spectrum of compound **7c** (300 MHz, acetone-d$_6$, 300 K).

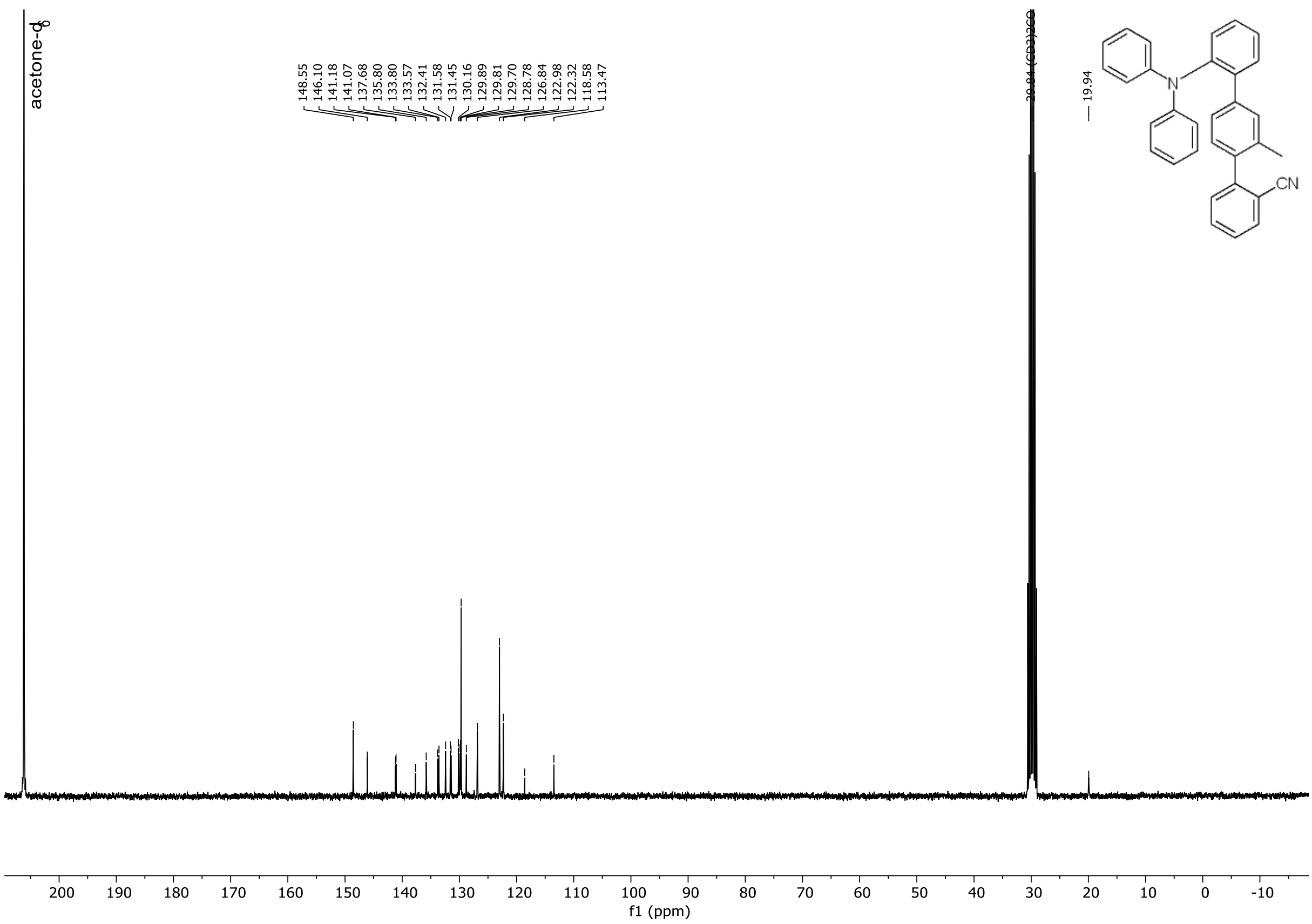

Figure S18. ^{13}C NMR spectrum of compound **7c** (75 MHz, acetone-d$_6$, 299 K).

3.1 Structural assignment of compound 6b by NMR spectroscopy

For the structural elucidation of compound **9b**, taken as a representative example for the donor-acceptor conjugates (Figure S19), ^{1}H, ^{13}C and 135-DEPT NMR spectra and mass spectra were recorded. In addition, 2D NMR spectroscopy, IR spectroscopy and elemental analysis were employed. Further signal assignments were supported by the application of the increment system.

The ^{1}H NMR spectrum of compound **9b** exhibits the expected number of signals. A relevant excerpt of the aromatic region is shown in Figure S19.

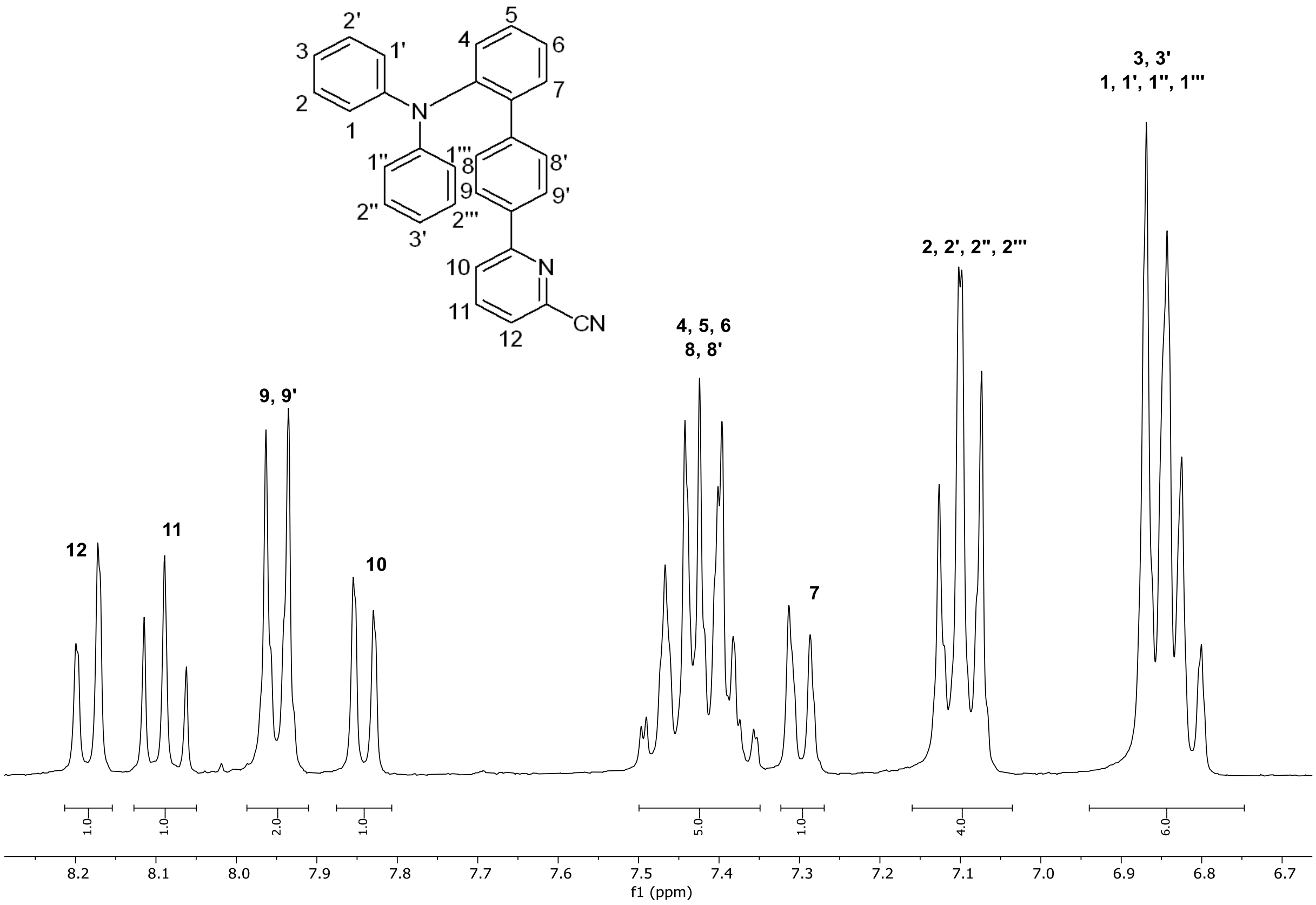

Figure S19. Excerpt of the ^{1}H NMR spectrum of compound **6b** in the chemical shift range of δ = 8.2–6.7 (recorded in acetone-d$_6$ on a 300 MHz NMR spectrometer at 298 K).

As anticipated, only aromatic proton signals are observed. Due to their proximity to the nitrogen atom and the electron-withdrawing cyano group within the acceptor moiety, the protons at positions 10, 11, and 12 are significantly downfield-shifted. The signal at δ 8.18 is assigned to the proton at position 12 and appears as a doublet of doublet with an integral of one and coupling constants of ^{3}J = 8.3 Hz and ^{4}J = 1.1 Hz. This splitting pattern arises from vicinal coupling with the proton at position 11 and long-range coupling to position 10. The signal at δ 8.09 appears as a triplet with a coupling constant of ^{3}J = 7.9 Hz and is attributed to the proton at position 11, reflecting vicinal coupling to the protons at positions 12 and 10. The observed coupling constant represents the average of the individual vicinal couplings (^{3}J = 8.3 Hz to position 12 and ^{3}J = 7.5 Hz to position 10). Furthermore, spatial proximity between the protons at positions 10, 11, and 12 is evident. At δ 7.95, a doublet with an integral of two and a coupling constant of ^{3}J = 8.4 Hz is observed and assigned to the protons at positions 9 and 9'. These protons are downfield-shifted due to their spatial proximity to the nitrogen atom at the acceptor unit. The doublet

splitting results from vicinal coupling to the protons at positions 8 and 8', whose signals overlap with others to form a multiplet. The signal at δ 7.84 appears as a doublet of doublet with an integral of one and is assigned to the proton at position 10. The splitting arises from vicinal coupling to position 11 (^3J = 7.5 Hz) and long-range coupling to position 12 (^4J = 1.1 Hz). The multiplet in the region δ 7.51–7.35, integrating for five protons, results from overlapping signals of the protons at positions 4, 5, 6, 8, and 8'. Both coupling interactions and spatial proximity to the protons at positions 9 and 9' are evident, which can be rationalized by coupling through positions 8 and 8'. At δ 7.30, a doublet of doublet with an integral of one and coupling constants of ^3J = 7.7 Hz and ^4J = 1.9 Hz is observed and assigned to the proton at position 7. This assignment is supported by its spatial proximity to the multiplet at δ 7.51–7.35 and the absence of proximity to positions 9 and 9'. Additionally, this proton is further removed from the donor nitrogen atom than the proton at position 4, resulting in a relative upfield shift. Based on the increment system, the multiplet at δ 7.14–7.06 (integral of four) is assigned to the chemical equivalent protons at positions 2, 2', 2'', and 2''', while the multiplet at δ 6.89–6.79 (integral of six) corresponds to the protons at positions 1, 1', 1'', 1''', 3, and 3'.

The ^{13}C NMR spectrum exhibits the expected number of signals (Figures S20 and S21). Figure S20 shows the ^{13}C NMR spectrum in the range of δ 160–134. The signal at δ 159.0 corresponds to a quaternary carbon nucleus, which is assigned to position 18. Due to its direct proximity to the nitrogen atom in the acceptor moiety, a pronounced downfield shift is observed. The signal at δ 148.5 also corresponds to a quaternary carbon atom and is assigned to the chemically equivalent carbon nuclei at positions 13 and 13'. Likewise, the signal at δ 145.8 represents another quaternary carbon atom, which is attributed to position 14. The direct proximity of the carbon atoms at positions 13, 13', and 14 to the donor nitrogen atom results in a downfield shift of these signals. The signals at δ 142.6 and δ 140.4 correspond to quaternary carbon atoms that are observed exclusively in π-extended derivatives. These signals are therefore assigned to the carbon atoms at positions 17 (δ 142.6) and 16 (δ 140.4). The carbon atom at position 17 is located closer to the acceptor nitrogen atom and thus exhibits a more pronounced downfield shift compared to position 16. The signal at δ 139.5 is identified as a methine carbon nucleus (CH) in the DEPT-135 spectrum and is assigned to position 11. The signal at δ 136.5 corresponds to a quaternary carbon atom at position 15, where the proximity to the donor nitrogen atom results in a downfield shift. The signal at δ 134.3 is attributed to a quaternary carbon atom at position 19. The electron-withdrawing cyano substituent in the α-position increases the local electron density, leading to a relative upfield shift of this signal.

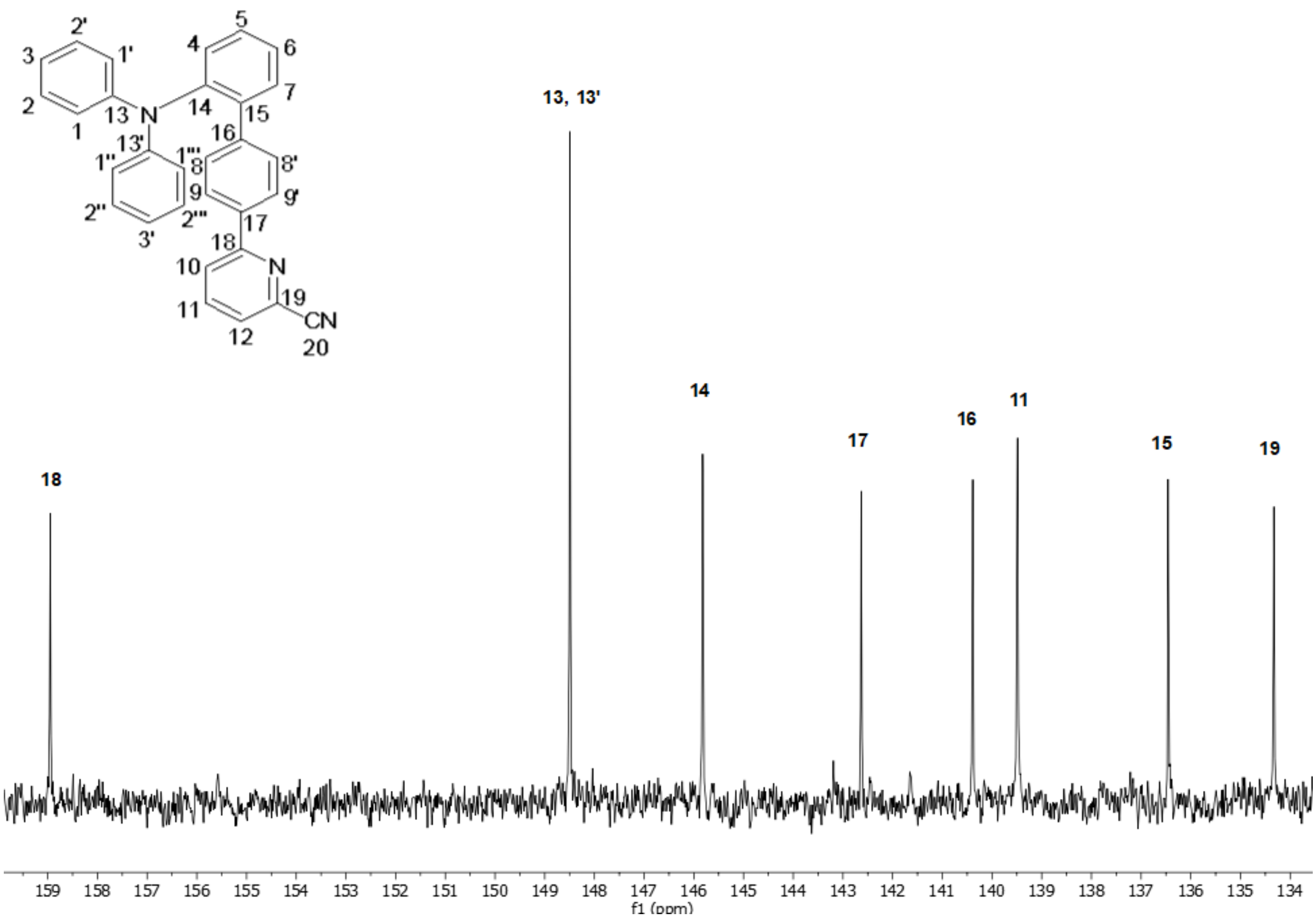

Figure S20. Excerpt of the ^{13}C NMR spectrum of compound **6b** in the chemical shift range of δ 160–134. Recorded in acetone-d$_6$ on a 75 MHz NMR spectrometer at 298 K.

Figure S21 displays the ^{13}C NMR spectrum in the range of δ 133–117. The signals at δ 132.6, 130.5, 130.2, and 130.0 ppm are assigned, based on the increment system, to the carbon atoms at positions 4, 5, 6, 8, and 8'. With increasing distance from the donor nitrogen atom, the signals shift progressively upfield. Accordingly, the signal at δ 132.6 ppm is assigned to position 4, δ 130.5 ppm to position 5, and δ 130.2 ppm to position 6. The chemically equivalent carbon atoms at positions 8 and 8' correspond to the signal at δ 130.0 ppm. Based on the increment system, the signal at δ 129.7 ppm is assigned to the chemically equivalent carbon atoms at positions 2, 2', 2'', and 2'''. The signal at δ 127.8 ppm is attributed to the carbon atom at position 7, supported by its coupling to the corresponding proton and its relatively large distance from the donor nitrogen atom results in an upfield shift. The signal at δ 127.3 ppm is assigned to the chemically equivalent carbon atoms at positions 9 and 9'. The carbon atoms at positions 10 and 12 are located in 3- and 1-positions, respectively, relative to the electron-withdrawing cyano substituent. Due to the resulting upfield shifts, these are assigned to the signals at δ 127.0 ppm (position 10) and δ 124.5 ppm (position 12). The signal at δ 122.9 ppm is attributed to the chemically equivalent carbon atoms at positions 1, 1', 1'', and 1''', while the signal at δ 122.5 ppm corresponds to the chemically equivalent carbon atoms at positions 3 and 3'. Finally, based on the increment system, the signal at δ 118.3 ppm is assigned to the quaternary carbon atom at position 20, which is part of the cyano substituent.

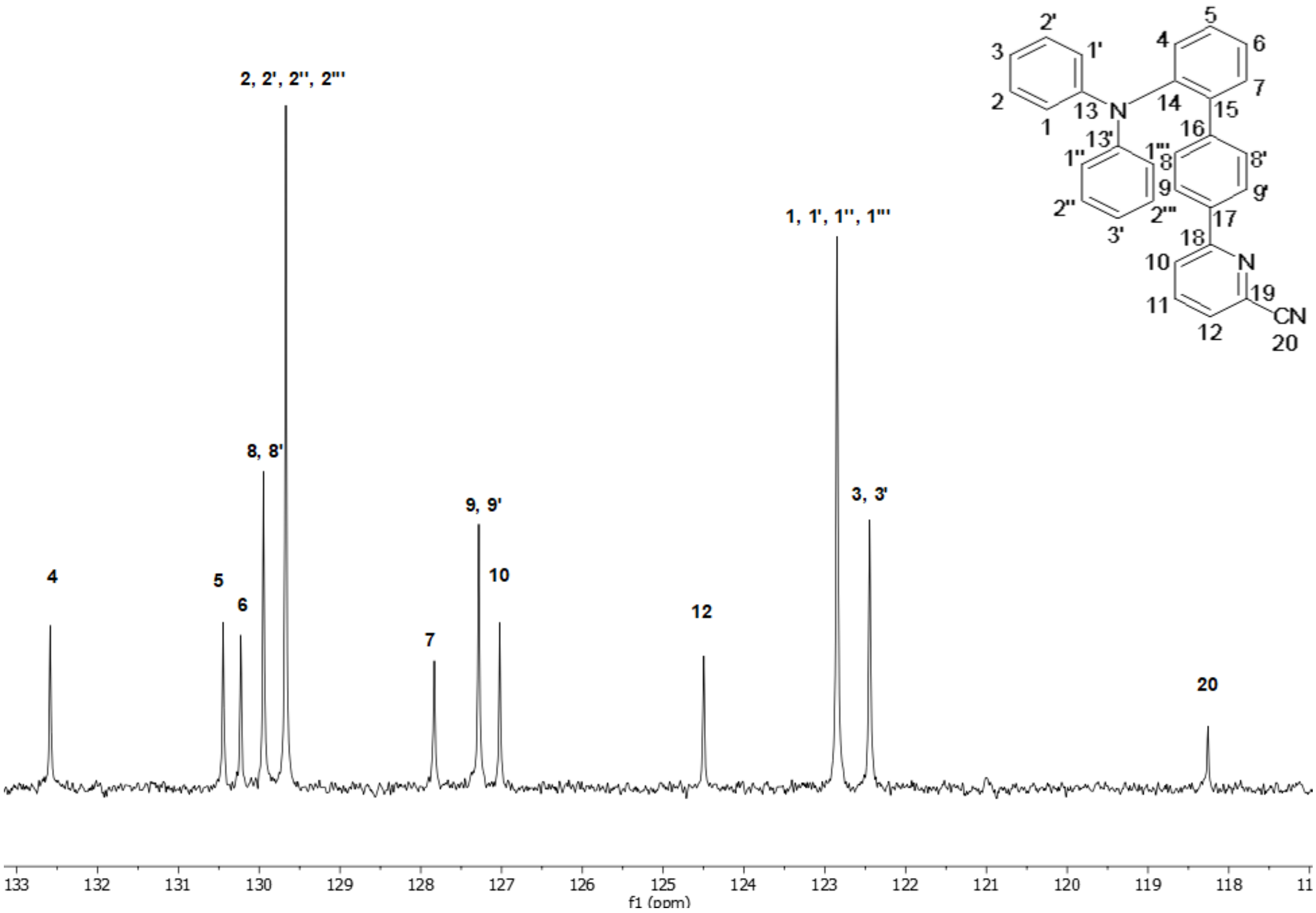

Figure S21. Excerpt of the ^{13}C NMR spectrum of compound **6b** in the chemical shift range of δ 133–117. Recorded in acetone-d_6 on a 75 MHz NMR spectrometer at 298 K.

4 Cyclic voltammetry

4.1 Dimerization process of compound 3a

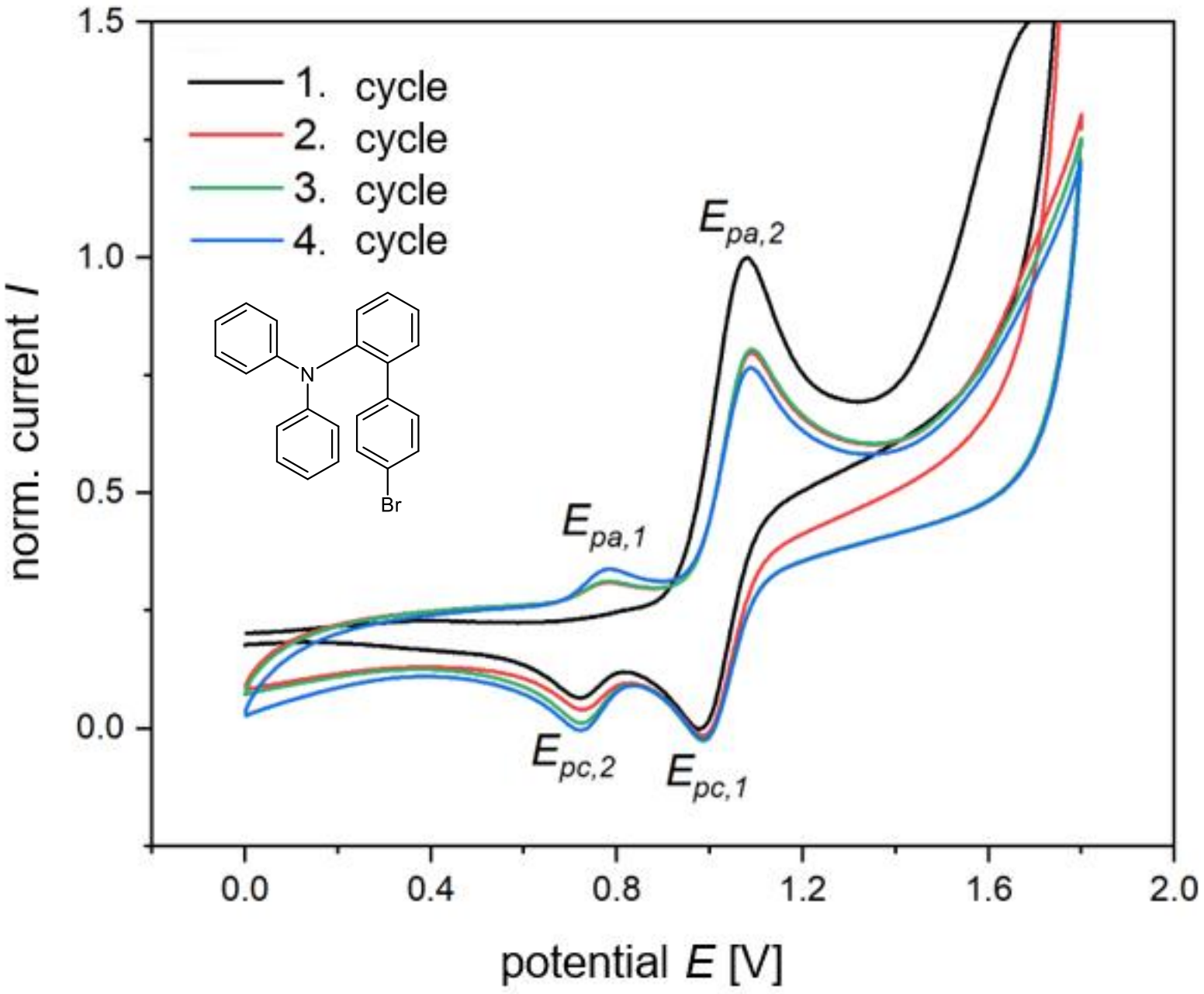

Figure S22. Cyclic voltammograms of compound **3a** for the first four measurement cycles (first black, second red, third green, fourth blue) recorded in dichloromethane (T = 293 K, ν = 100 mV/s, electrolyte: [^{n}Bu$_4$N][PF$_6$], Pt working electrode, Pt counter electrode, Ag/AgCl reference electrode, redox standard: decamethylferrocene $E_0^{0/+1}$ = -0.55 V (vs. ferrocene $E_0^{0/+1}$ = 0.00 V[2])).

4.2 Dimerization process of compounds 6a–c and 7a–c

The cyclic voltammograms of compounds **6** and **7** exhibit irreversible behavior, as evidenced by the appearance of two redox processes of different magnitudes upon the second measurement cycle (Figure S23).

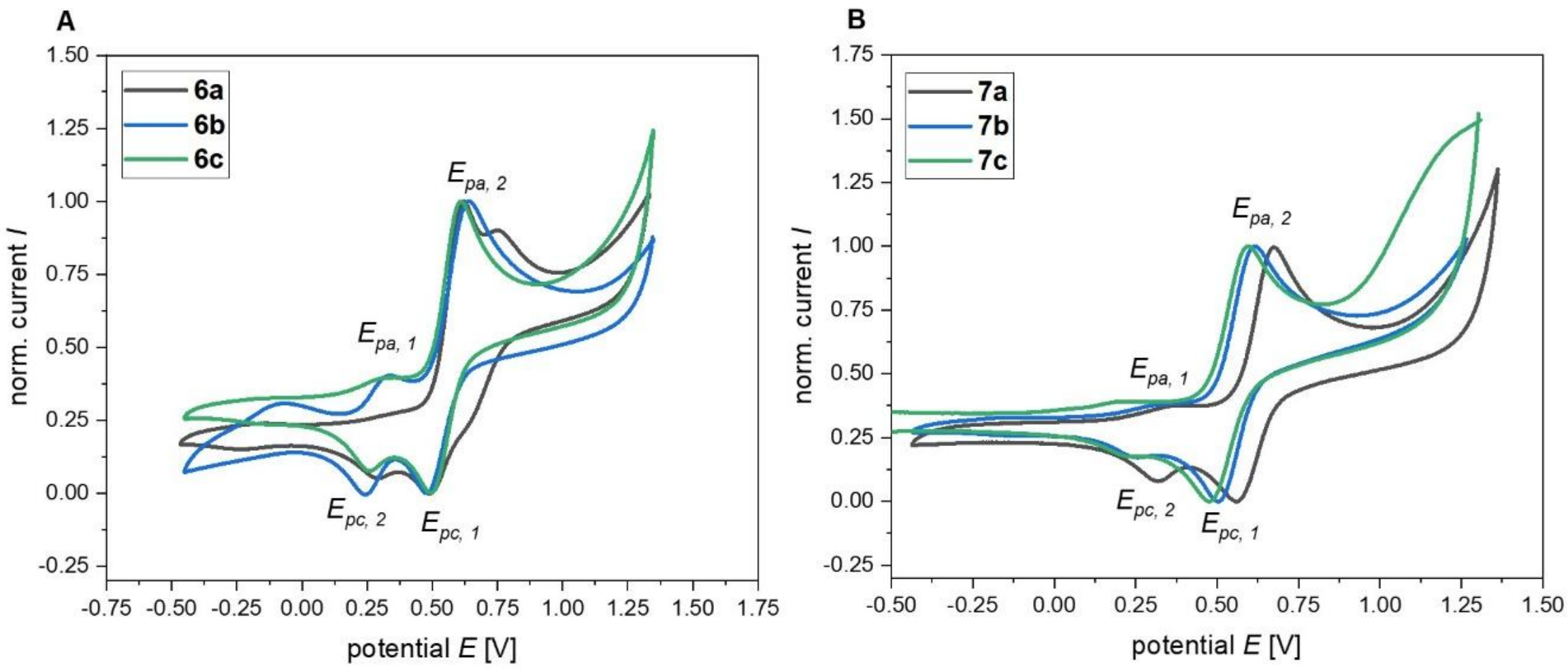

Figure S23. Cyclic voltammograms (second measurement cycle) of of cyanopyridine- **6** (**A**) and benzonitrile-based donor–acceptor conjugates **7** (**B**) of the directly linked compound (**6a**, **7a**: black), the π-extended (**6b**, **7b**: blue) and the π-extended with additional steric hindrance (**6c**, **7c**: green) recorded in CH_2Cl_2 (T = 293 K, ν = 100 mV/s, electrolyte: [^{n}Bu$_4$N][PF$_6$], Pt working electrode, Pt counter electrode, Ag/AgCl reference electrode, redox standard: decamethylferrocene $E_0^{0/+1}$ = –0.55 V (vs. ferrocene $E_0^{0/+1}$ = 0.00 V[2])).

During the first anodic half-cycle, oxidation occurs at $E_{pa,2}$ (Figure S23), leading to the formation of an unstable radical cation. As for the oxidative dimerization of triphenylamine (TPA) (Scheme S1), followed by subsequent dimerization on the electrode surface, deprotonation occurs to restore aromaticity. The resulting *N*,*N*,*N'*,*N'*-tetraaryl benzidine unit can undergo two sequential oxidation processes.[5] These originate from the radical cation of the dimer at $E_{pa,1}$, first observed in the second measurement cycle, and proceed to the dication of the dimer. The latter overlaps with the oxidation of the monomer at $E_{pa,2}$, resulting in a single intense signal (Figure S23). The electronic properties of tetraaryl benzidine-based dimers of cyanopyridine-based conjugates **6a** and **6b** as monomers were separately investigated.[6] In the cathodic half-cycle two reduction processes are observed, the first at $E_{pc,1}$ corresponds to the formation of the radical cation of the dimer, while the second at $E_{pc,2}$ yields to the neutral dimer (Figure S23).

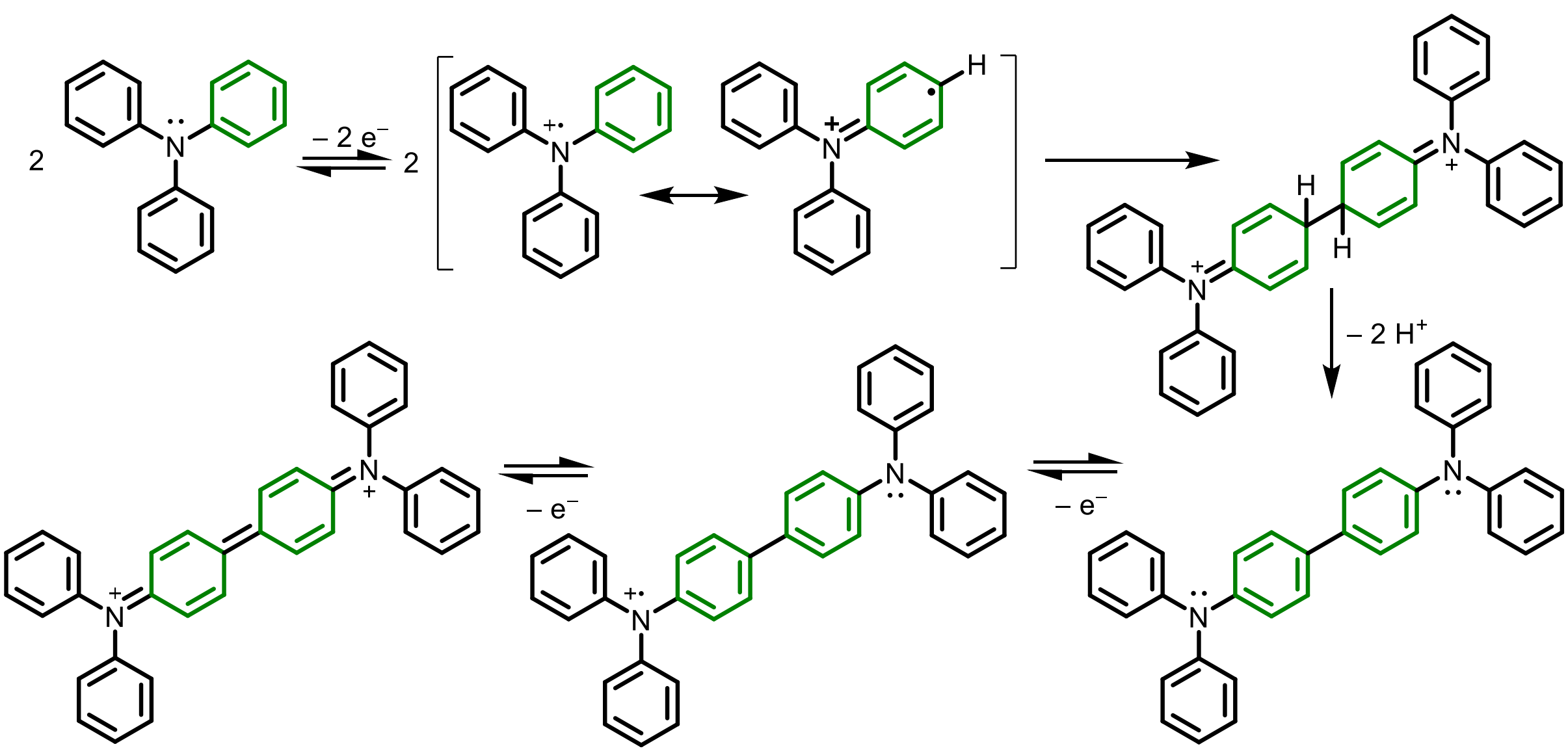

Scheme S1. Electrochemical dimerization of TPA.

With an increased number of sweep cycles, the intensity of $E_{pa,2}$ (0.59–0.67 V) decreases due to the progressive consumption of the monomer during dimer formation (Figure S23). In contrast, the intensity of $E_{pa,1}$ (0.22–0.40 V), $E_{pc,1}$ (0.47–0.56 V) and $E_{pc,2}$ (0.21–0.31 V) increase, reflecting the accumulation of the dimer (Table S3, Figure S22). For the formation of the radical cation, the half-wave potentials $E_0^{0/+1}$ for the triarylamine monomers **6** (0.55–0.57 V) and **7** (0.53–0.62 V) are determined (Table S3). The redox process appears to be localized on the triphenylamine donor moieties, as variations in the acceptor units do not significantly influence the redox potentials. Compound **6a** exhibits an additional third redox process, which could be attributed to a second oxidation of the triphenylamine unit, leading to the formation of a dication (Figure S23A). Notably, the π-extended benzonitrile-based donor–acceptor conjugates (**7b** and **7c**) display lower first redox potentials, consistent with increased delocalization of the radical cation over the larger π-system, whereas the cyanopyridine-based conjugates **6** show comparable values. The dimerization behavior of *ortho*-biaryl-substituted triarylamines has been extensively studied previously.[7] In this context, compound **7a** can be compared to the structural similar compound **8**[7] (Table S3), confirming the characteristic behavior of this substance class.

Table S3. Selected electrochemical properties (anodic peak potentials $E_{pa,1}$ and $E_{pa,2}$ as well as cathodic peak potentials $E_{pc,1}$ and $E_{pc,2}$ and the first redox potential $E_0^{0/+1}$ of the monomers **6** and **7** and compare compound **8**[7]).

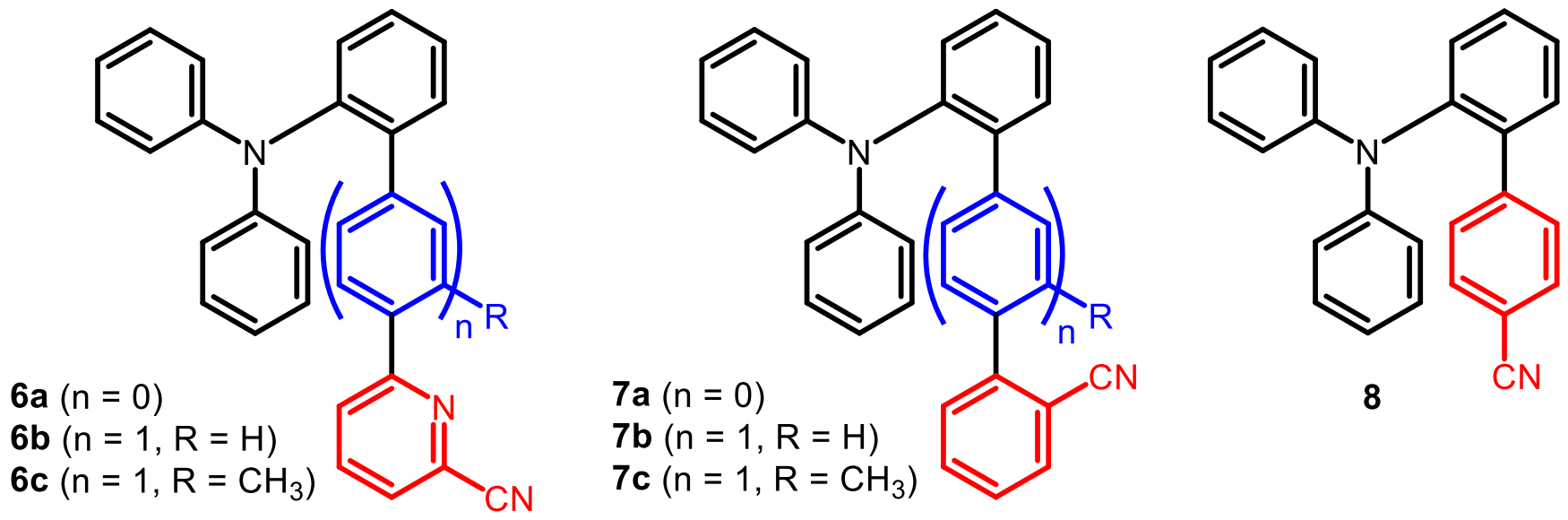

compound	$E_{pa,1}$ [a] / V	$E_{pa,2}$ [a] / V	$E_{pc,1}$ [a] / V	$E_{pc,2}$ [a] / V	$E_0^{0/+1}$ [b] / V
**6a**	0.35	0.61	0.49	0.29	0.55
**6b**	0.40	0.64	0.48	0.24	0.57
**6c**	0.40	0.61	0.49	0.25	0.55
**7a**	0.39	0.67	0.56	0.31	0.62
**7b**	0.32	0.62	0.50	0.24	0.56
**7c**	0.22	0.59	0.47	0.21	0.53
**8**	0.39	0.71	0.58	0.32	0.63

[a] Recorded in CH_2Cl_2, T = 293 K, v = 100 mV/s, electrolyte: [^{n}Bu$_4$N][PF$_6$], Pt working electrode, Pt counter electrode, Ag/AgCl reference electrode, redox standard: decamethylferrocene $E_0^{0/+1}$ = –0.55 V (vs. ferrocene $E_0^{0/+1}$ = 0.00 V[2]). [b] Recorded in CH_2Cl_2, T = 293 K, extrapolation of data with v = 100, 250, 500, 1000 to 0.00 mV/s, electrolyte: [^{n}Bu$_4$N][PF$_6$], Pt working electrode, Pt counter electrode, Ag/AgCl reference electrode, $E_0 = \frac{(E_{pa}-E_{pc})}{2}$ with redox standard: decamethylferrocene $E_0^{0/+1}$ = –0.55 V (vs. ferrocene $E_0^{0/+1}$ = 0.00 V[2]).

4.3 Cyclic voltammograms of conjugates 6 and 7

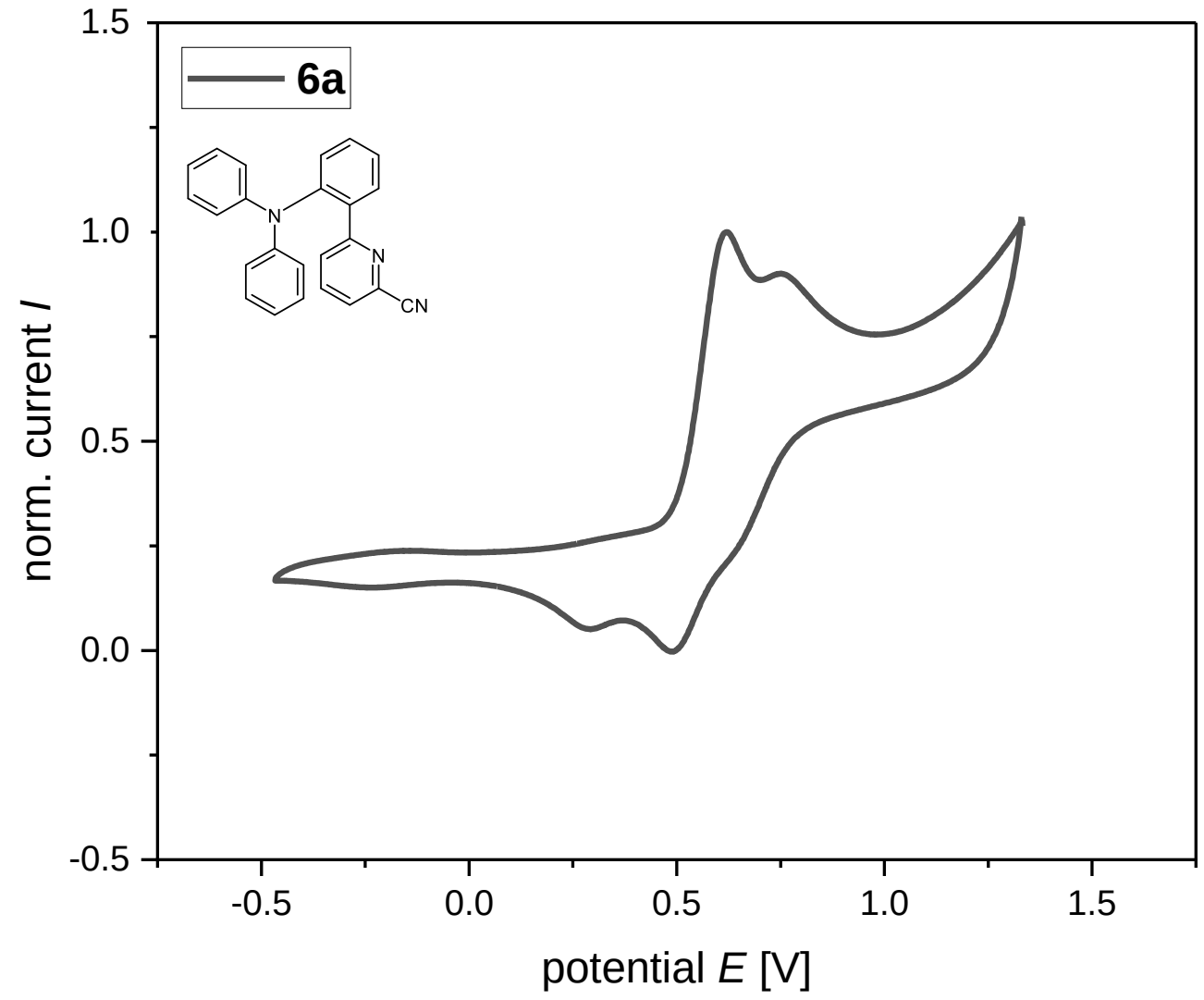

Figure S24. Cyclic voltammogram of compound **6a** (second measurement cycle) recorded in dichloromethane (T = 293 K, v = 100 mV/s, electrolyte: [^{n}Bu$_4$N][PF$_6$], Pt working electrode, Pt counter electrode, Ag/AgCl reference electrode, redox standard: decamethylferrocene $E_0^{0/+1}$ = –0.55 V (vs. ferrocene $E_0^{0/+1}$ = 0.00 V[2])).

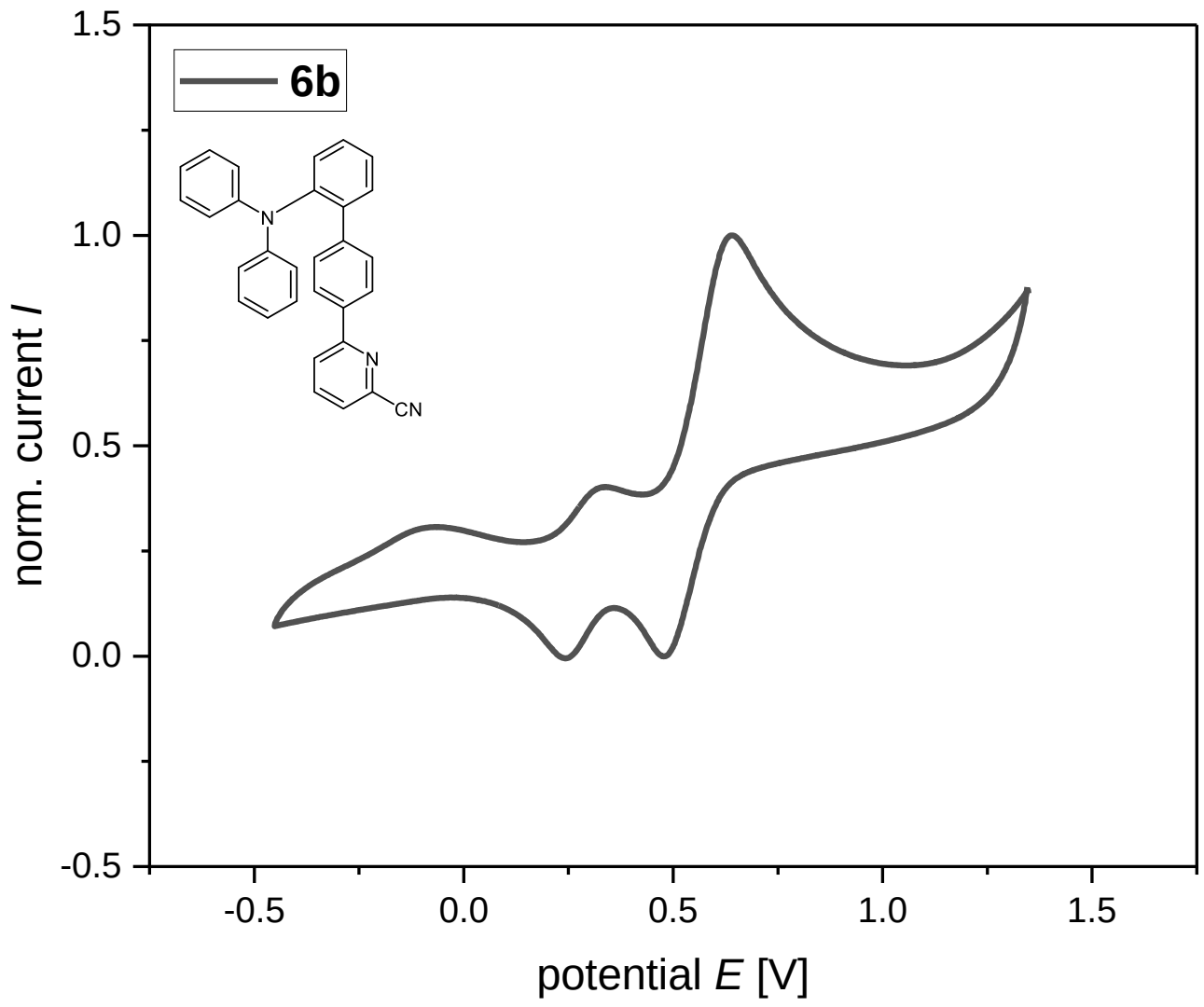

Figure S25. Cyclic voltammogram of compound **6b** (second measurement cycle) recorded in dichloromethane (*T* = 293 K, ν = 100 mV/s, electrolyte: [^{n}Bu$_4$N][PF$_6$], Pt working electrode, Pt counter electrode, Ag/AgCl reference electrode, redox standard: decamethylferrocene $E_0^{0/+1}$ = −0.55 V (vs. ferrocene $E_0^{0/+1}$ = 0.00 V[2])).

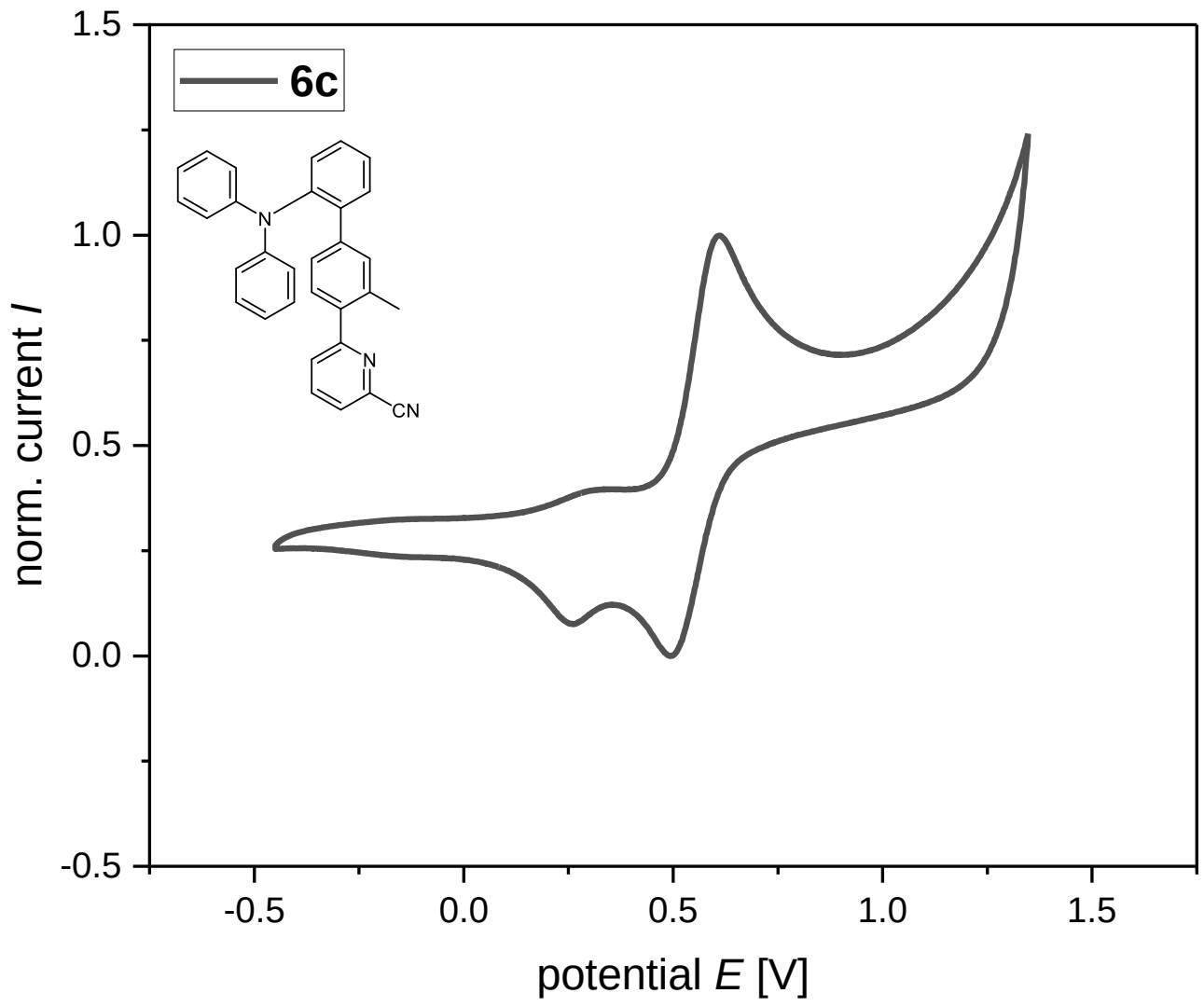

Figure S26. Cyclic voltammogram of compound **6c** (second measurement cycle) recorded in dichloromethane (*T* = 293 K, ν = 100 mV/s, electrolyte: [^{n}Bu$_4$N][PF$_6$], Pt working electrode, Pt counter electrode, Ag/AgCl reference electrode, redox standard: decamethylferrocene $E_0^{0/+1}$ = −0.55 V (vs. ferrocene $E_0^{0/+1}$ = 0.00 V[2])).

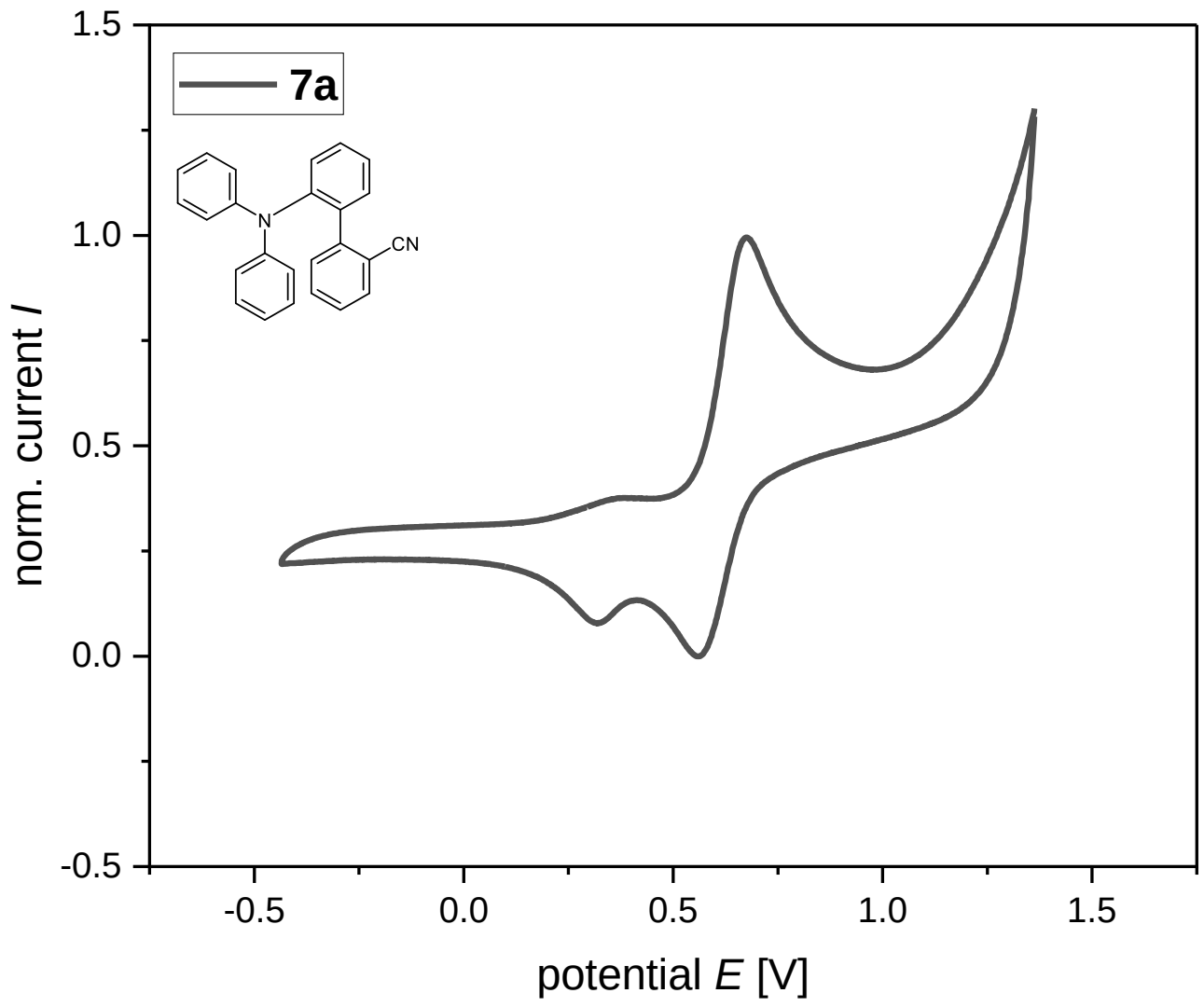

Figure S27. Cyclic voltammogram of compound **7a** (second measurement cycle) recorded in dichloromethane (*T* = 293 K, ν = 100 mV/s, electrolyte: [^{n}Bu$_4$N][PF$_6$], Pt working electrode, Pt counter electrode, Ag/AgCl reference electrode, redox standard: decamethylferrocene $E_0^{0/+1}$ = −0.55 V (vs. ferrocene $E_0^{0/+1}$ = 0.00 V[2])).

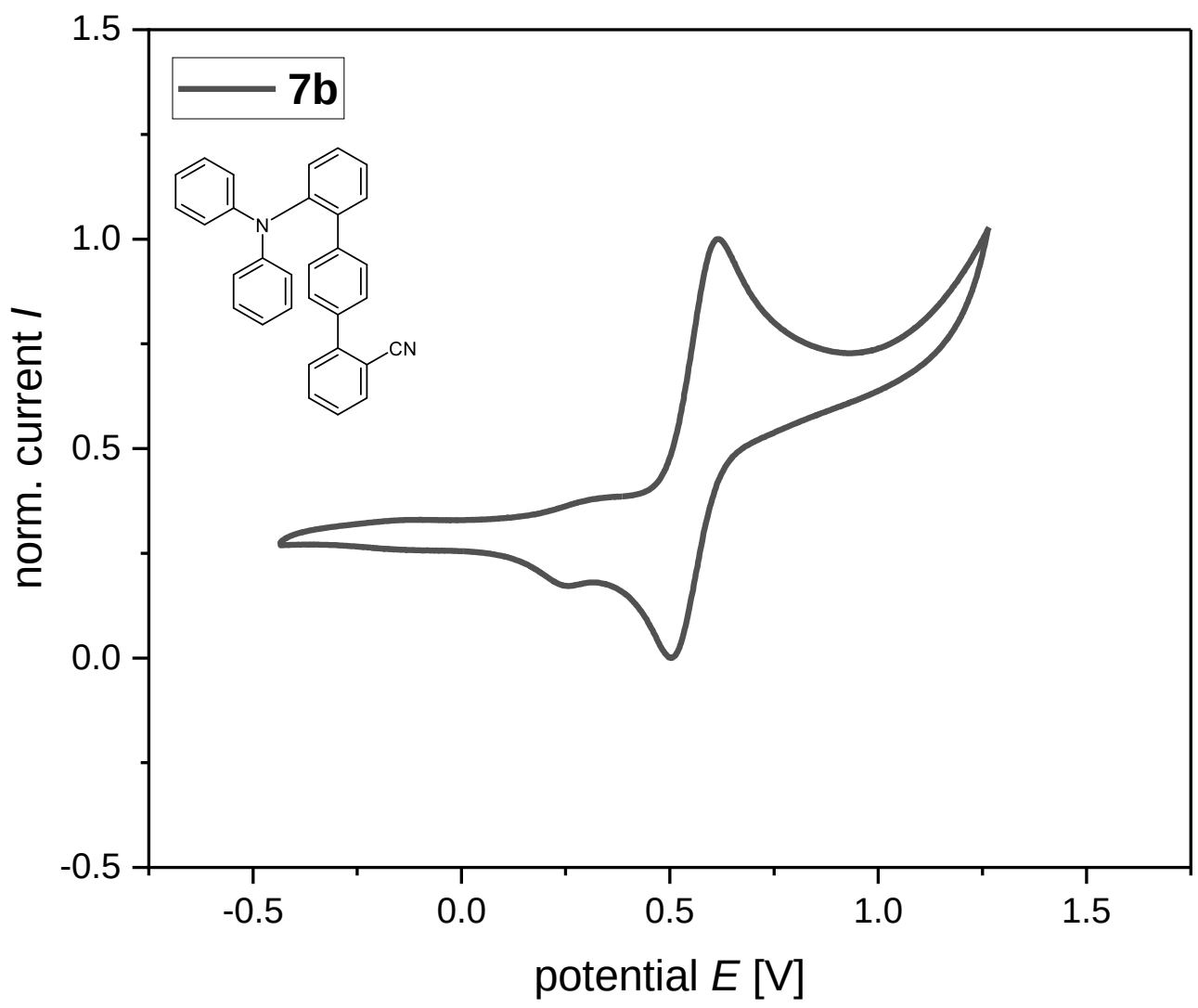

Figure S28. Cyclic voltammogram of compound **7b** (second measurement cycle) recorded in dichloromethane (*T* = 293 K, ν = 100 mV/s, electrolyte: [^{n}Bu$_4$N][PF$_6$], Pt working electrode, Pt counter electrode, Ag/AgCl reference electrode, redox standard: decamethylferrocene $E_0^{0/+1}$ = −0.55 V (vs. ferrocene $E_0^{0/+1}$ = 0.00 V[2])).

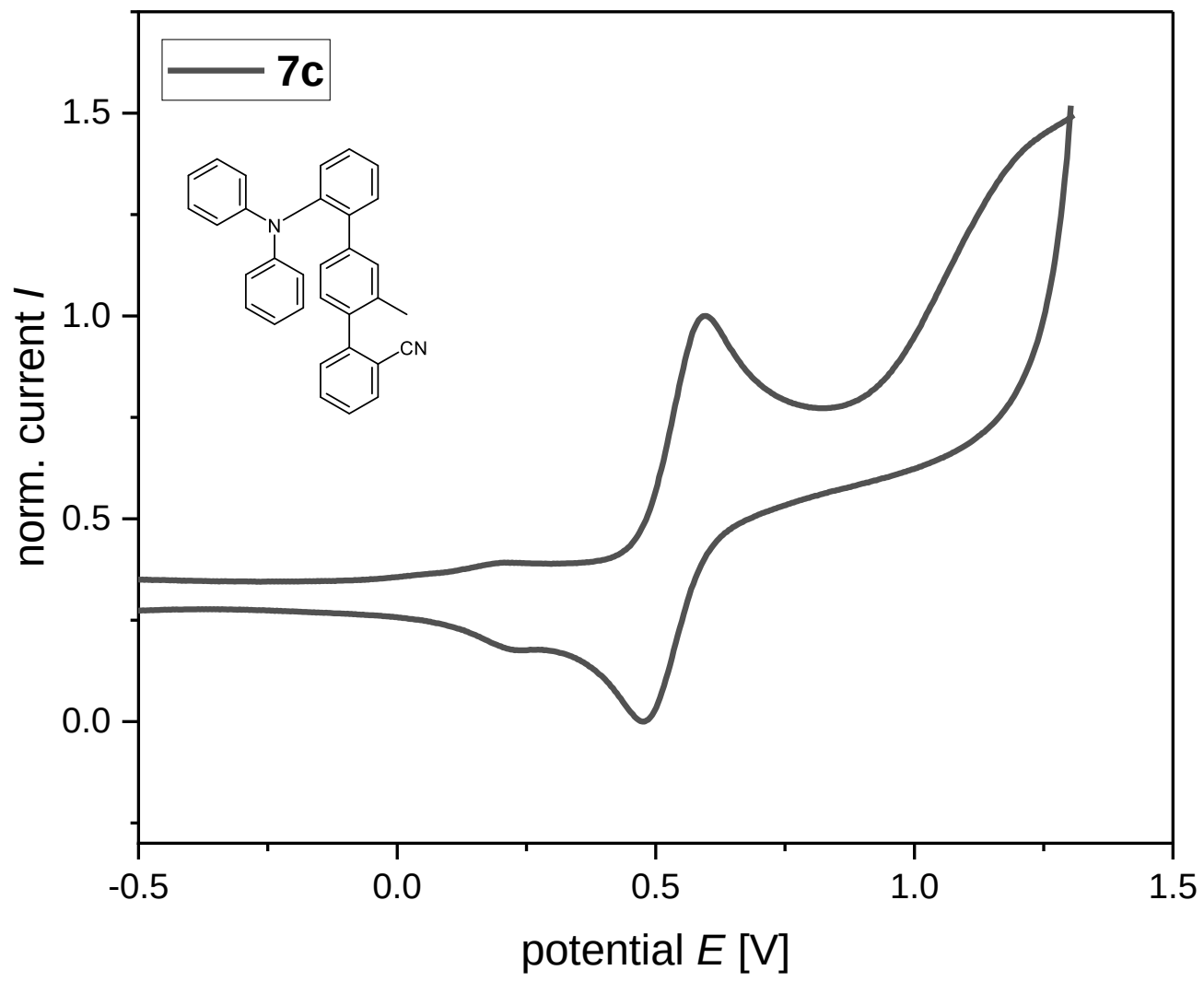

Figure S29. Cyclic voltammogram of compound **7c** (second measurement cycle) recorded in dichloromethane (T = 293 K, ν = 100 mV/s, electrolyte: [^{n}Bu$_4$N][PF$_6$], Pt working electrode, Pt counter electrode, Ag/AgCl reference electrode, redox standard: decamethylferrocene $E_0^{0/+1}$ = −0.55 V (vs. ferrocene $E_0^{0/+1}$ = 0.00 V[2])).

5 UV/Vis absorption and emission spectra of 6 and 7

5.1 Emission spectra in the solid state

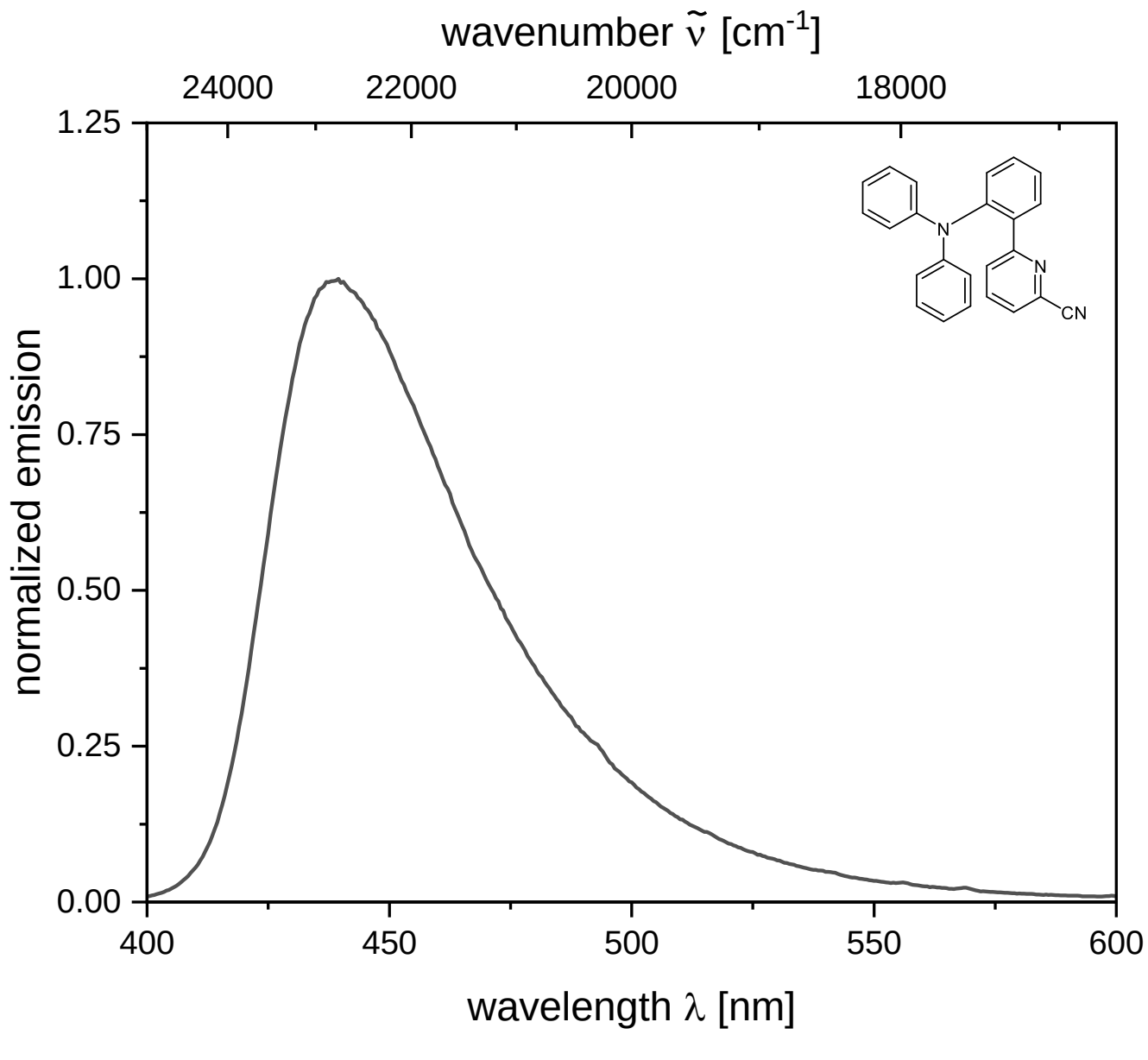

Figure S30. Normalized emission spectrum of **6a** recorded in the solid state, T = 298 K, λ_{exc} = 351 nm.

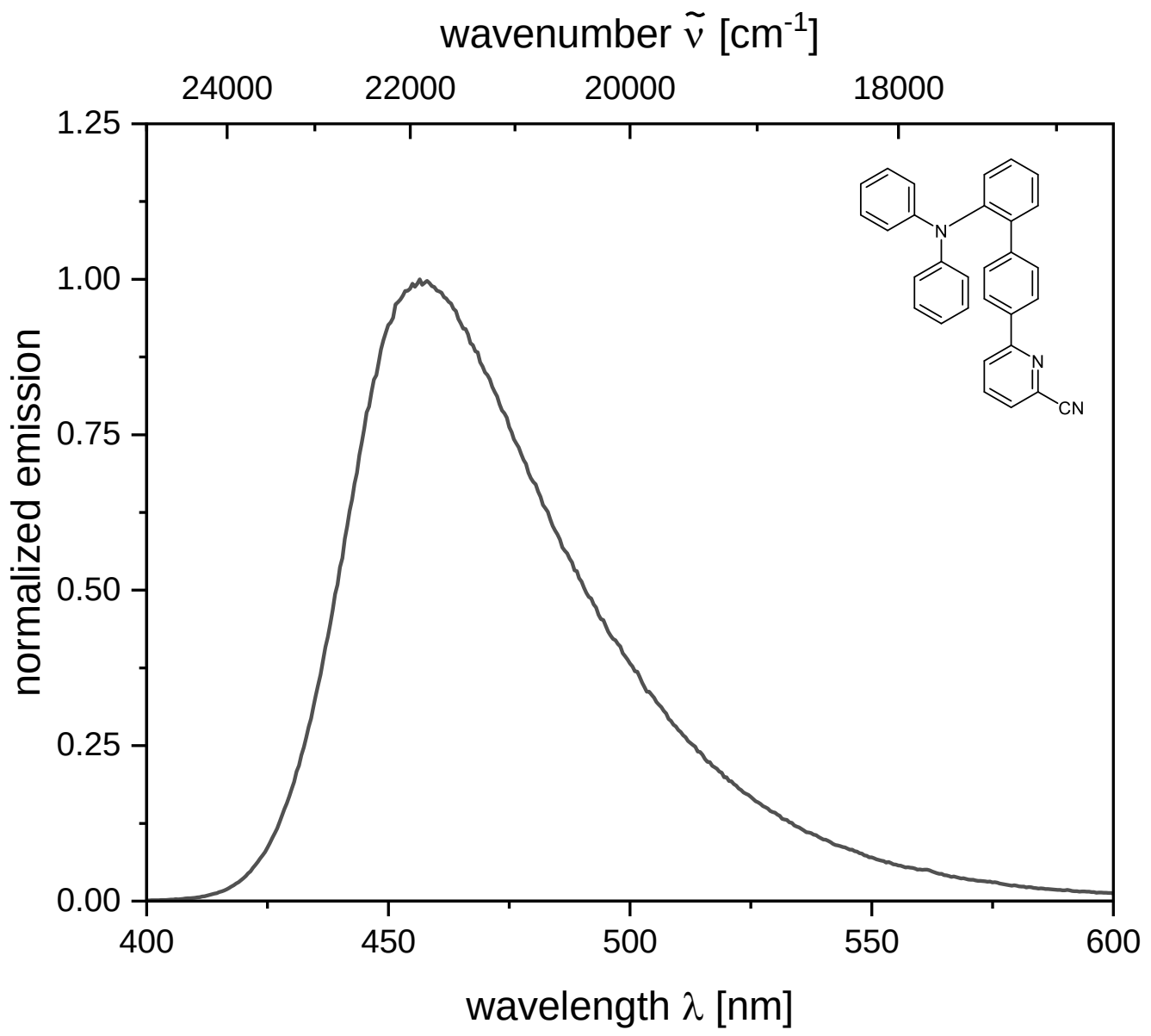

Figure S31. Normalized emission spectrum of **6b** recorded in the solid state, *T* = 298 K, λ_{exc} = 353 nm.

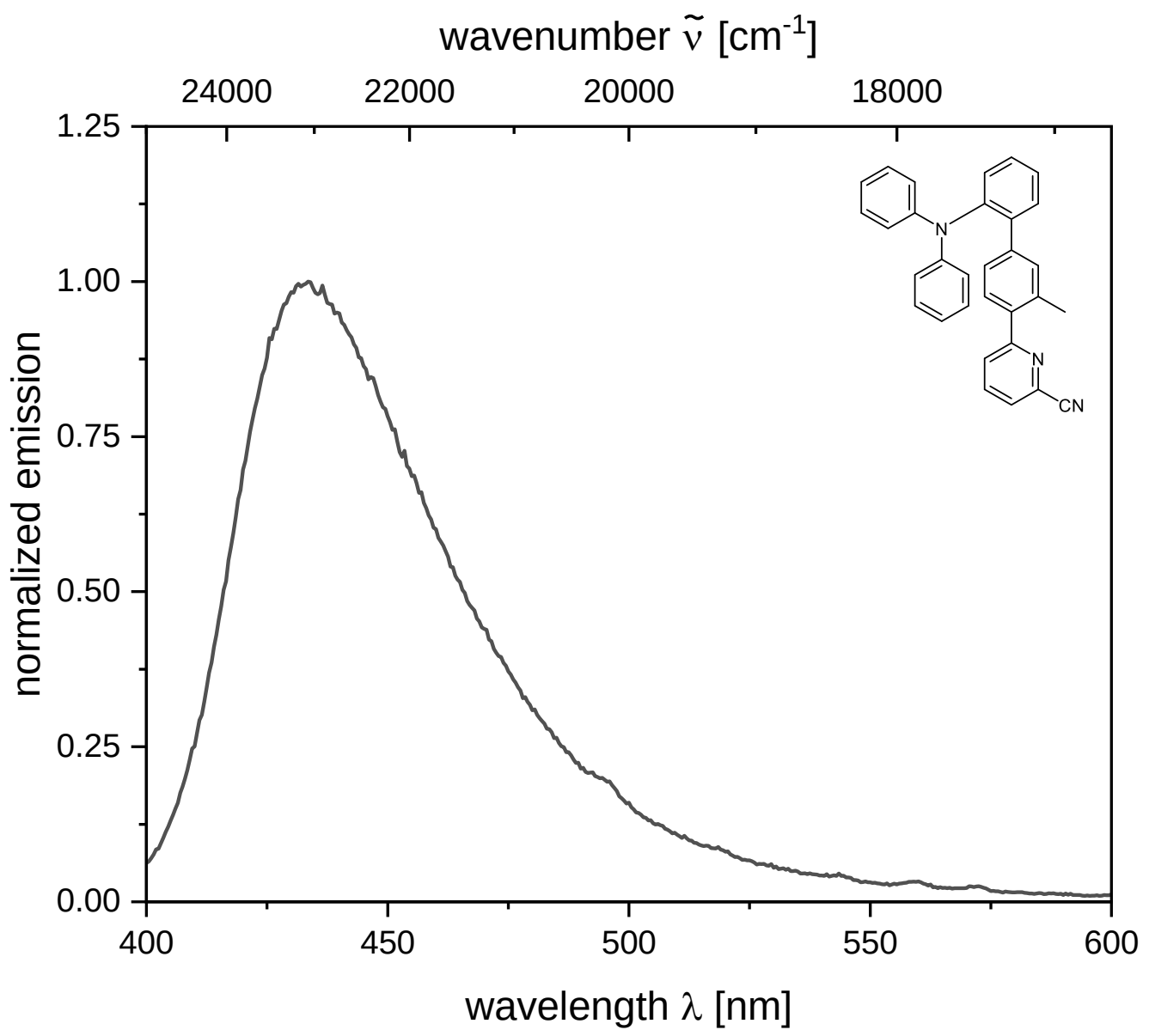

Figure S32. Normalized emission spectrum of **6c** recorded in the solid state, *T* = 298 K, λ_{exc} = 347 nm.

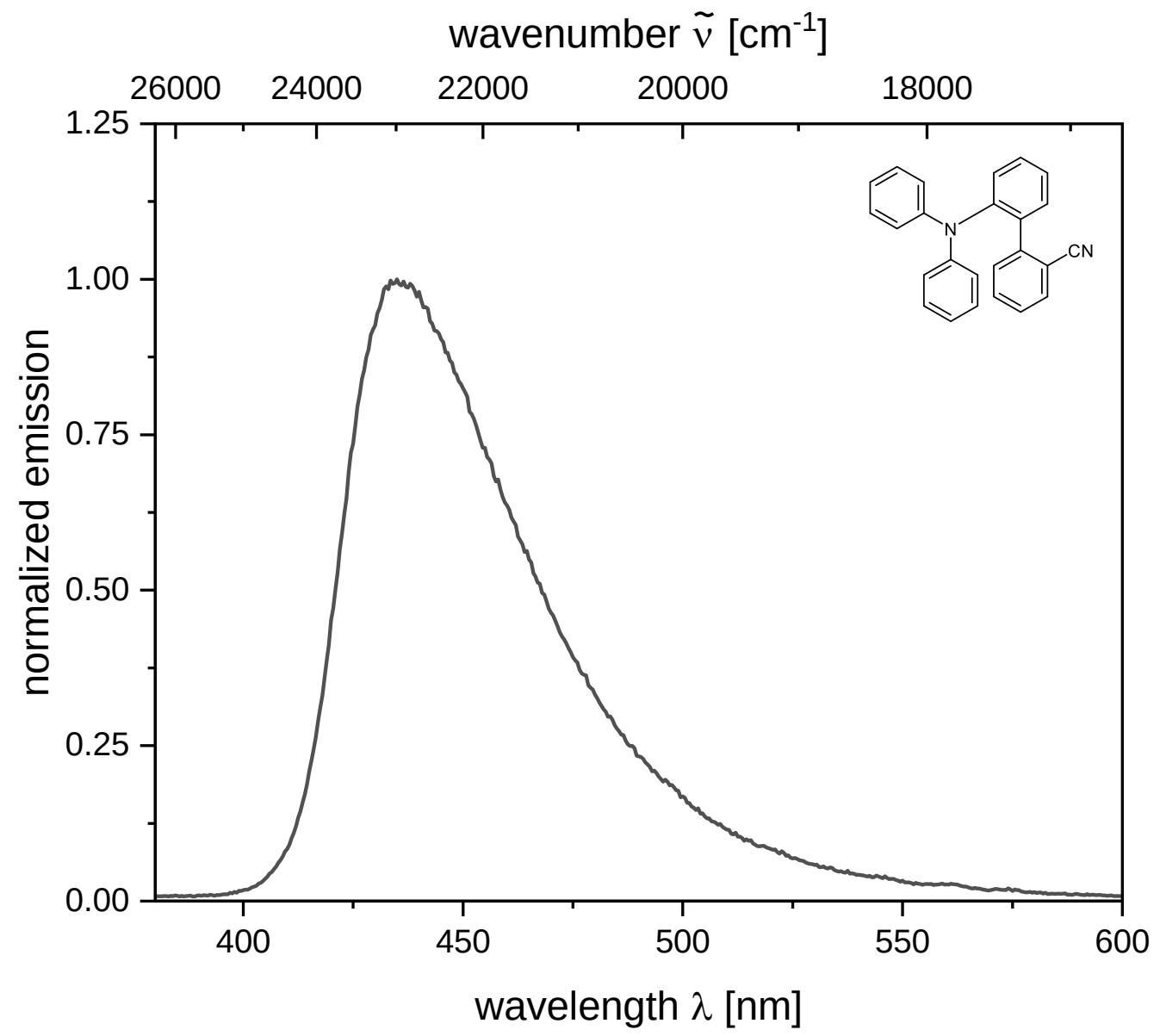

Figure S33. Normalized emission spectrum of **7a** recorded in the solid state, *T* = 298 K, λ_{exc} = 349 nm.

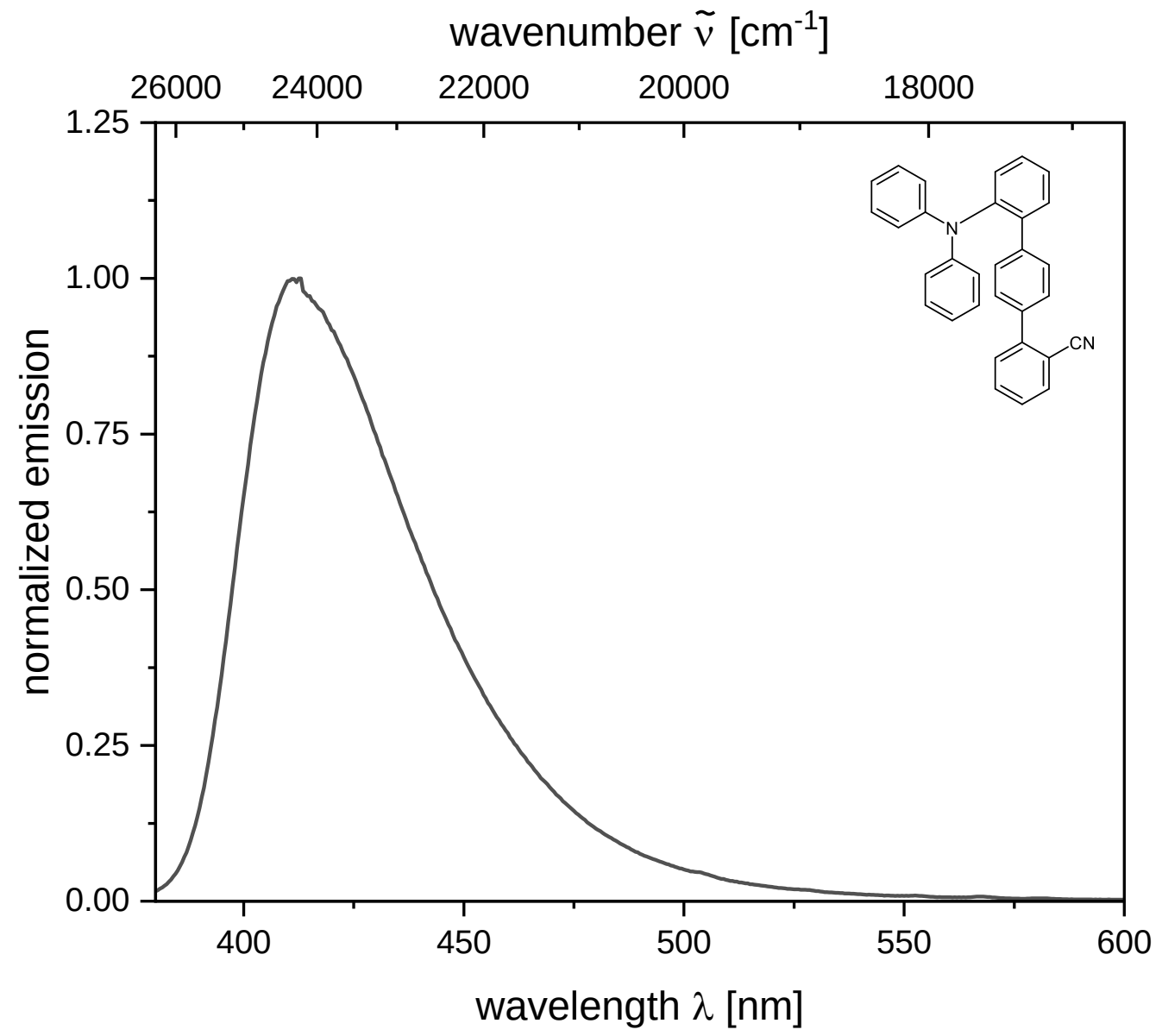

Figure S34. Normalized emission spectrum of **7b** recorded in the solid state, *T* = 298 K, λ_{exc} = 345 nm.

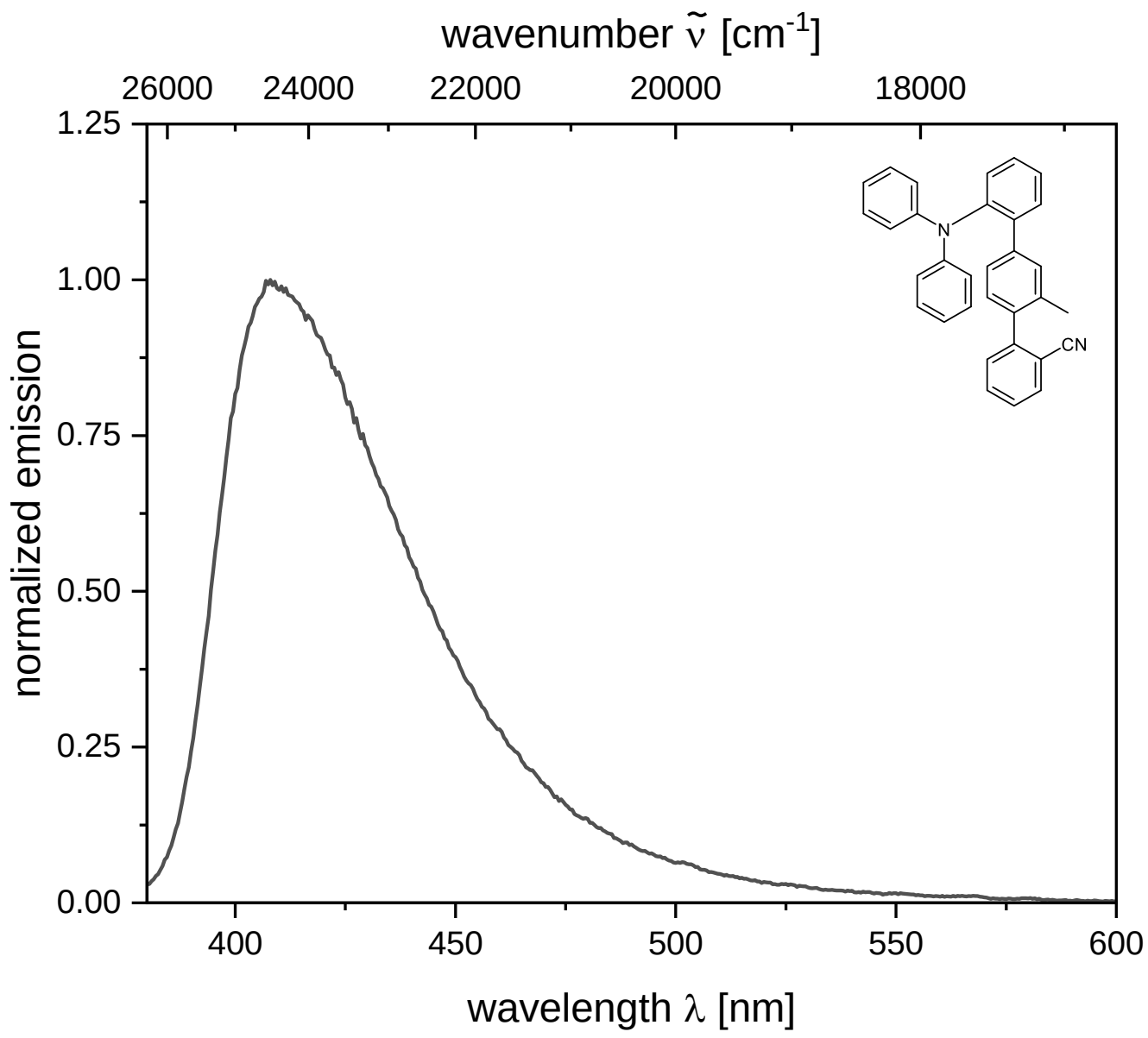

Figure S35. Normalized emission spectrum of **7c** recorded in the solid state, T = 298 K, λ_{exc} = 348 nm.

Table S4. Selected photophysical properties (emission maxima, photoluminescence quantum yields, FWHM, fluorescence lifetimes, radiative and non-radiative rate constants) of compound **6** and **7** in the solid state.

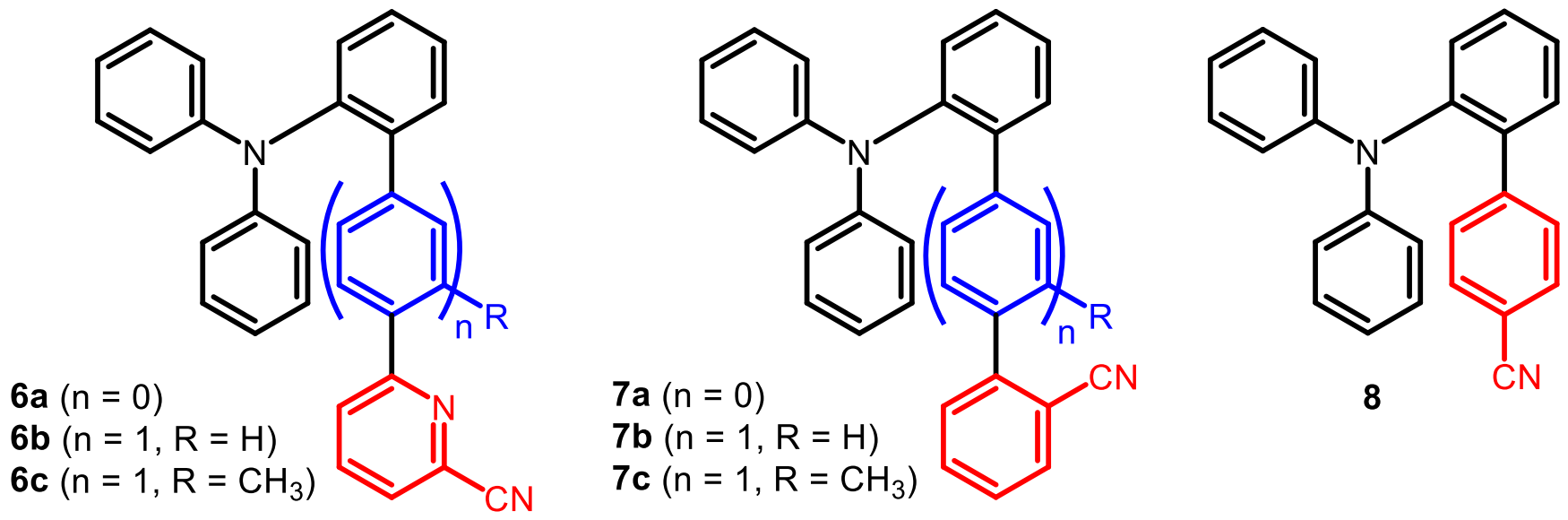

compound	$\lambda_{max,em}$ / nm (Φ_{PL})[a]	FWHM / cm^{-1} (nm)[b]	τ / ns (χ^2)[c]	k_r^{exp} / s^{-1} [d]	k_{nr}^{exp} / s^{-1} [e]
**6a**	440 (0.47)	2480 (48)	9.6 (1.09)	$4.9 \cdot 10^7$	$5.5 \cdot 10^7$
**6b**	457 (0.31)	2490 (52)	12.5 (1.19)	$2.5 \cdot 10^7$	$5.5 \cdot 10^7$
**6c**	434 (0.70)	2600 (49)	7.0 (1.09)	$1.0 \cdot 10^8$	$4.3 \cdot 10^7$
**7a**	435 (0.29)	2480 (47)	7.1 (1.02)	$4.1 \cdot 10^7$	$1.0 \cdot 10^8$
**7b**	413 (0.24)	2700 (46)	3.8 (1.12)	$6.3 \cdot 10^7$	$2.0 \cdot 10^8$
**7c**	408 (0.31)	2880 (48)	3.9 (1.08)	$7.9 \cdot 10^7$	$1.8 \cdot 10^8$

[a] Emission in the solid state and absolute photoluminescence quantum yield Φ_{PL} determined with an integrating sphere (powder sample, T = 293 K). [b] FWHM [cm^{-1}] determined using $10^7 \cdot$ (FWHM [nm]/(λ_{em} [nm])^2). [c] Residuals χ^2. [d] $k_r^{exp} = \frac{\Phi_{PL}}{\tau}$. [e] $k_{nr}^{exp} = \frac{1-\Phi_{PL}}{\tau}$.

5.2 UV/Vis absorption and emission spectra in solution

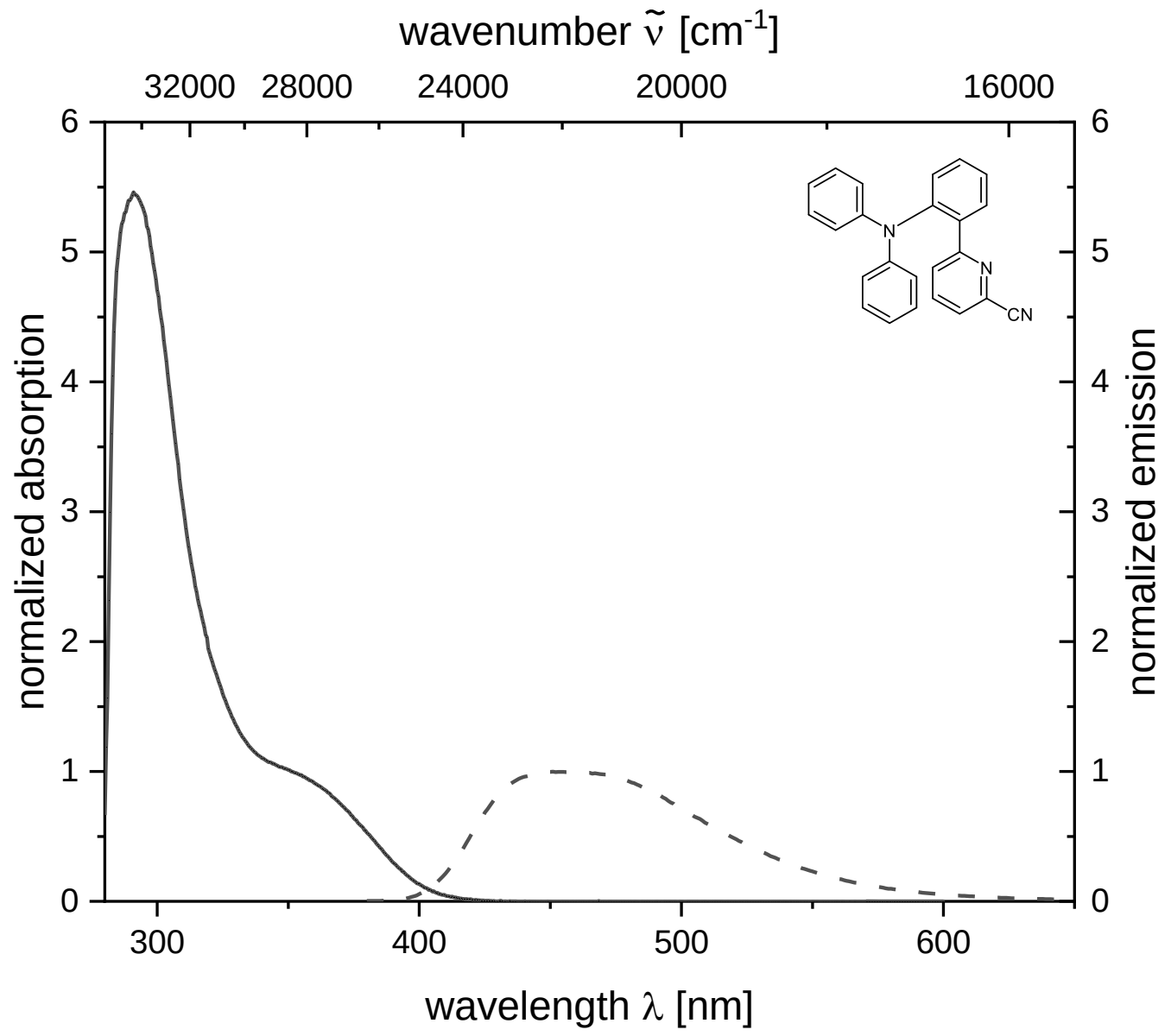

Figure S36. Normalized absorption (solid line) and emission spectrum (dashed line) of **6a** recorded in toluene, T = 298 K, c_{abs}(**6a**) = 10^{-5} M, c_{em}(**M46**) = 10^{-6} M, $\lambda_{exc} = \lambda_{max}$.

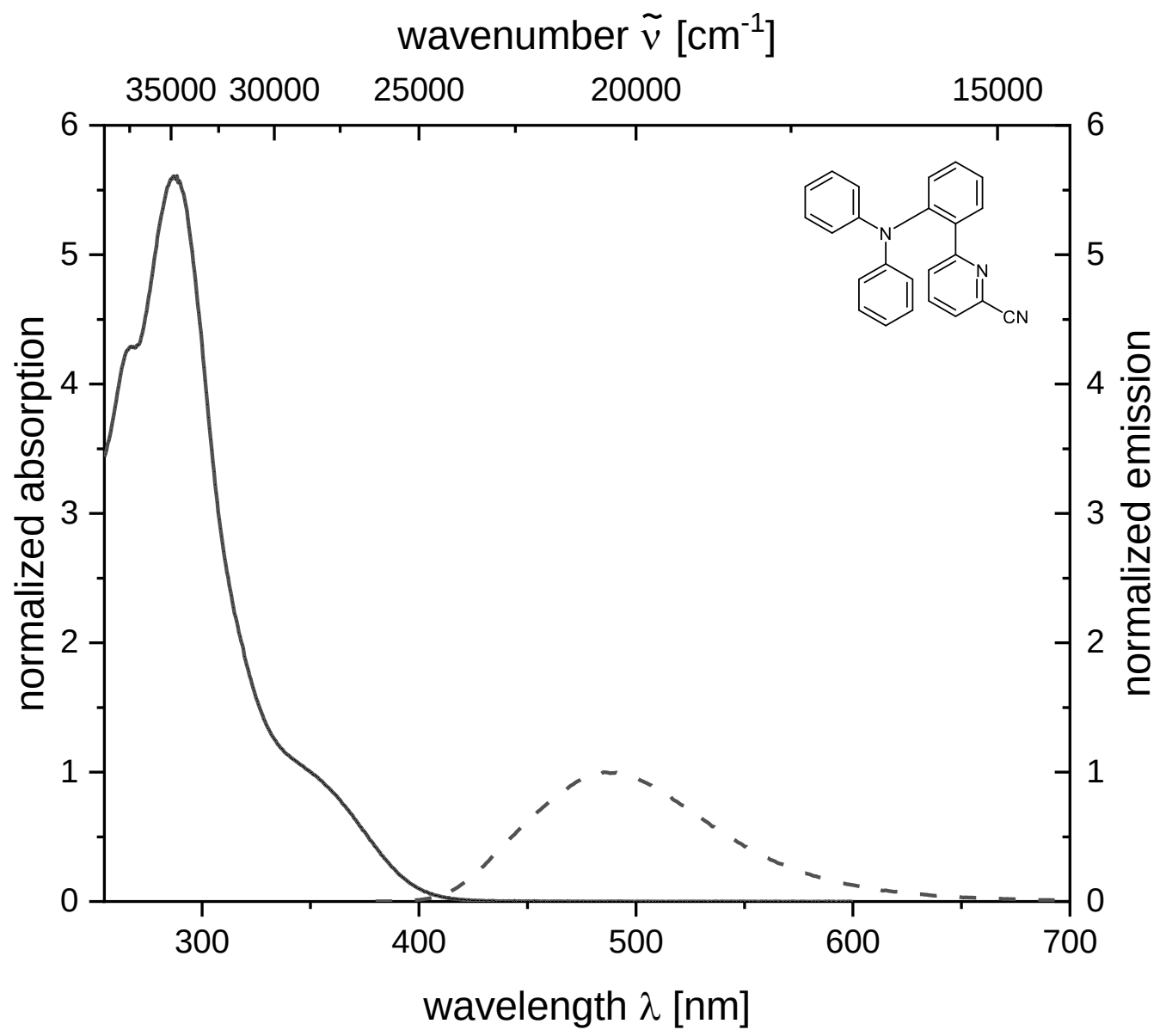

Figure S37. Normalized absorption (solid line) and emission spectrum (dashed line) of **6a** recorded in ethyl acetate, T = 298 K, c_{abs}(**6a**) = 10^{-5} M, c_{em}(**6a**) = 10^{-6} M, $\lambda_{exc} = \lambda_{max}$.

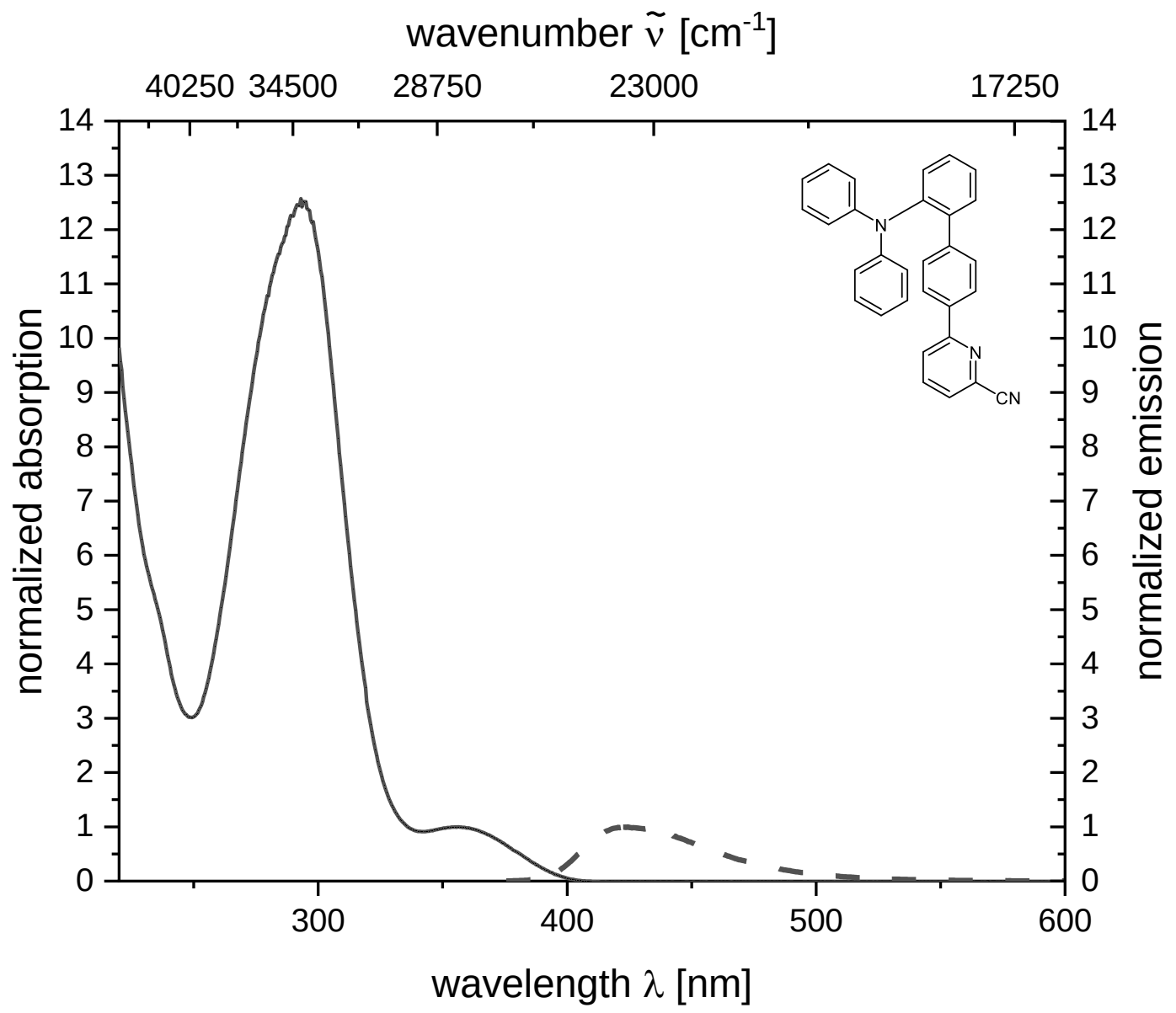

Figure S38. Normalized absorption (solid line) and emission spectrum (dashed line) of **6b** recorded in cyclohexane, T = 298 K, c_{abs}(**6b**) = 10^{-5} M, c_{em}(**6b**) = 10^{-6} M, $\lambda_{exc} = \lambda_{max}$.

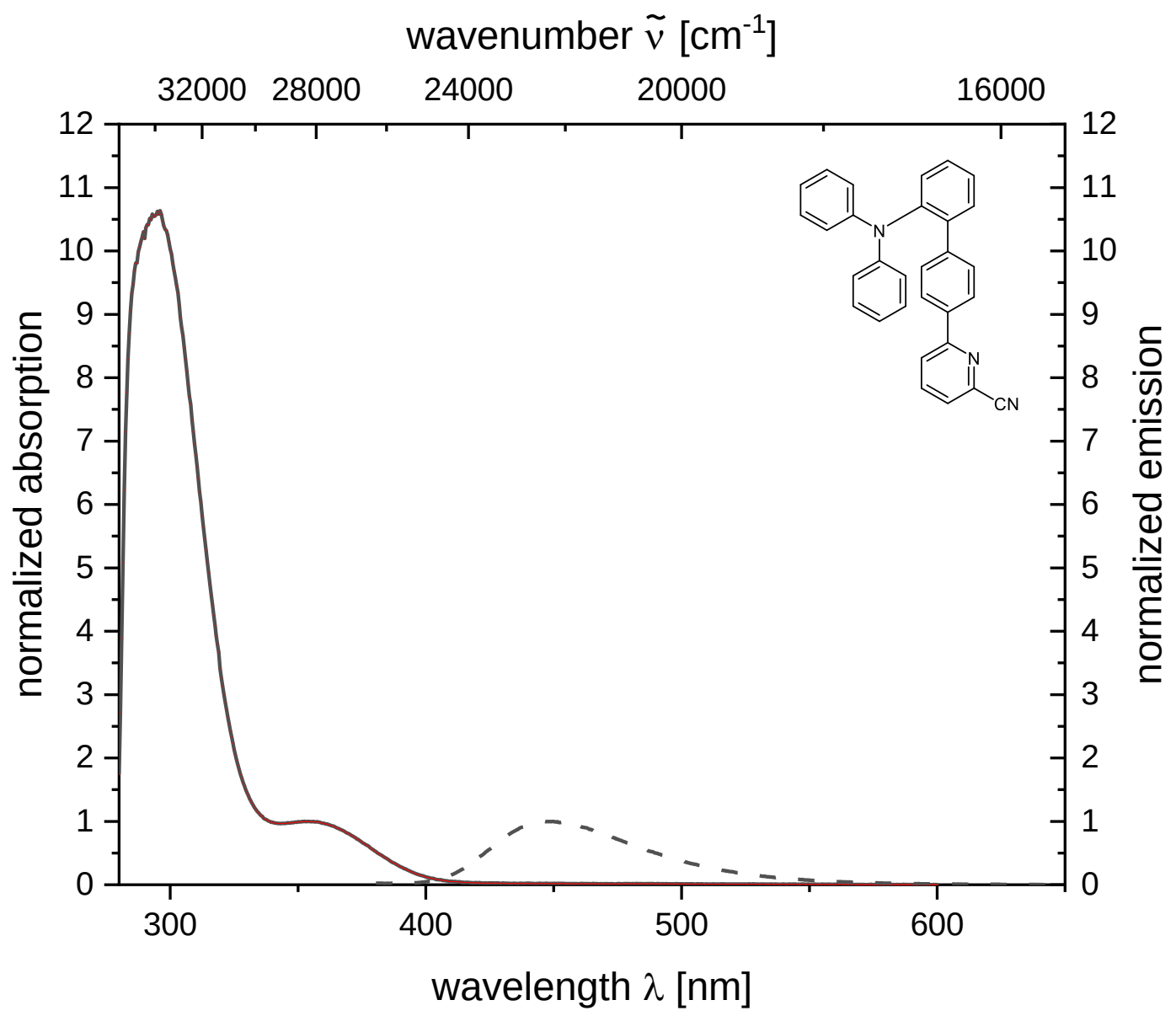

Figure S39. Normalized absorption (solid line) and emission spectrum (dashed line) of **6b** recorded in toluene, T = 298 K, c_{abs}(**6b**) = 10^{-5} M, c_{em}(**6b**) = 10^{-6} M, $\lambda_{exc} = \lambda_{max}$.

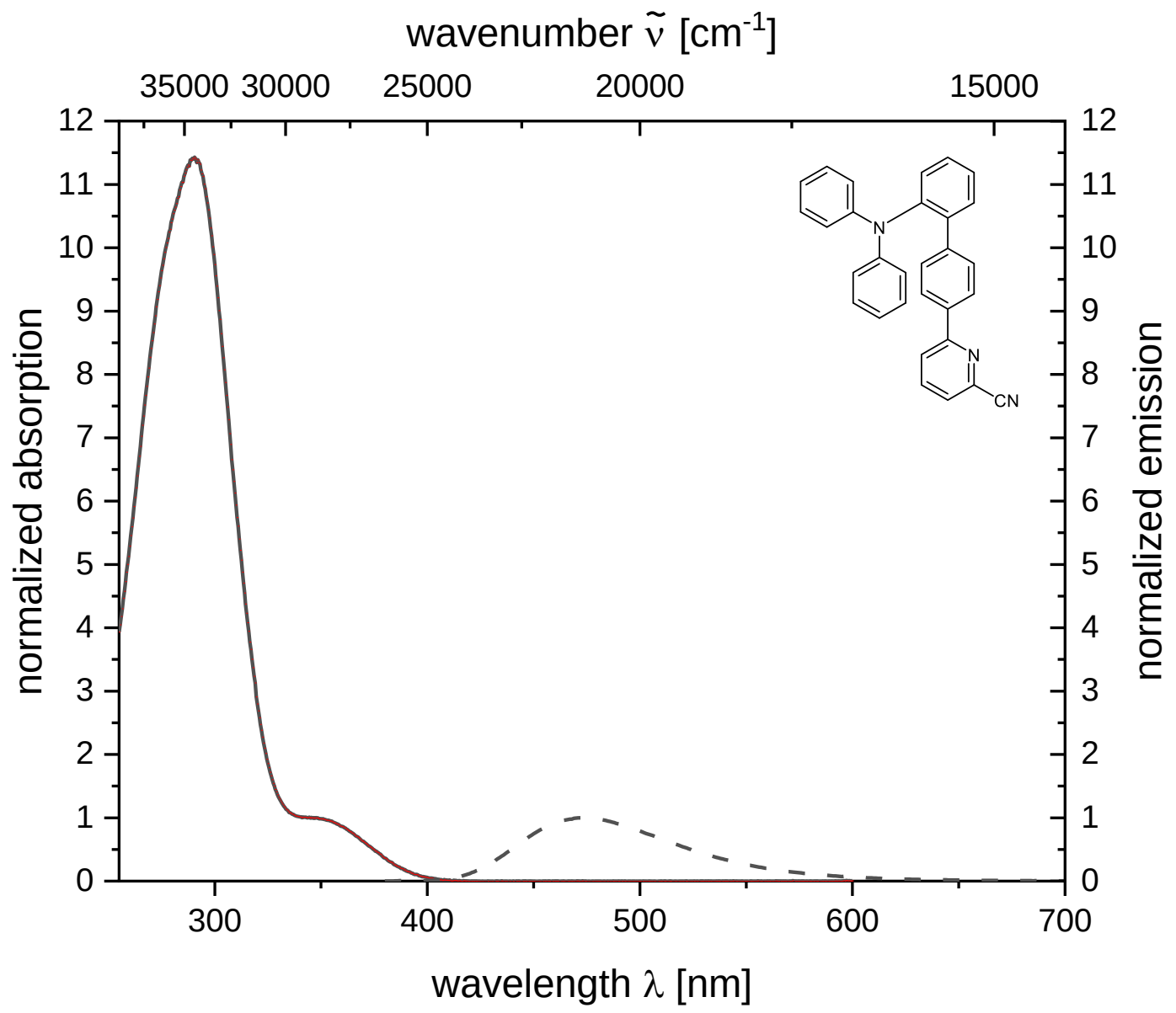

Figure S40. Normalized absorption (solid line) and emission spectrum (dashed line) of **6b** recorded in ethyl acetate, T = 298 K, c_{abs}(**6b**) = 10^{-5} M, c_{em}(**6b**) = 10^{-6} M, $\lambda_{exc} = \lambda_{max}$.

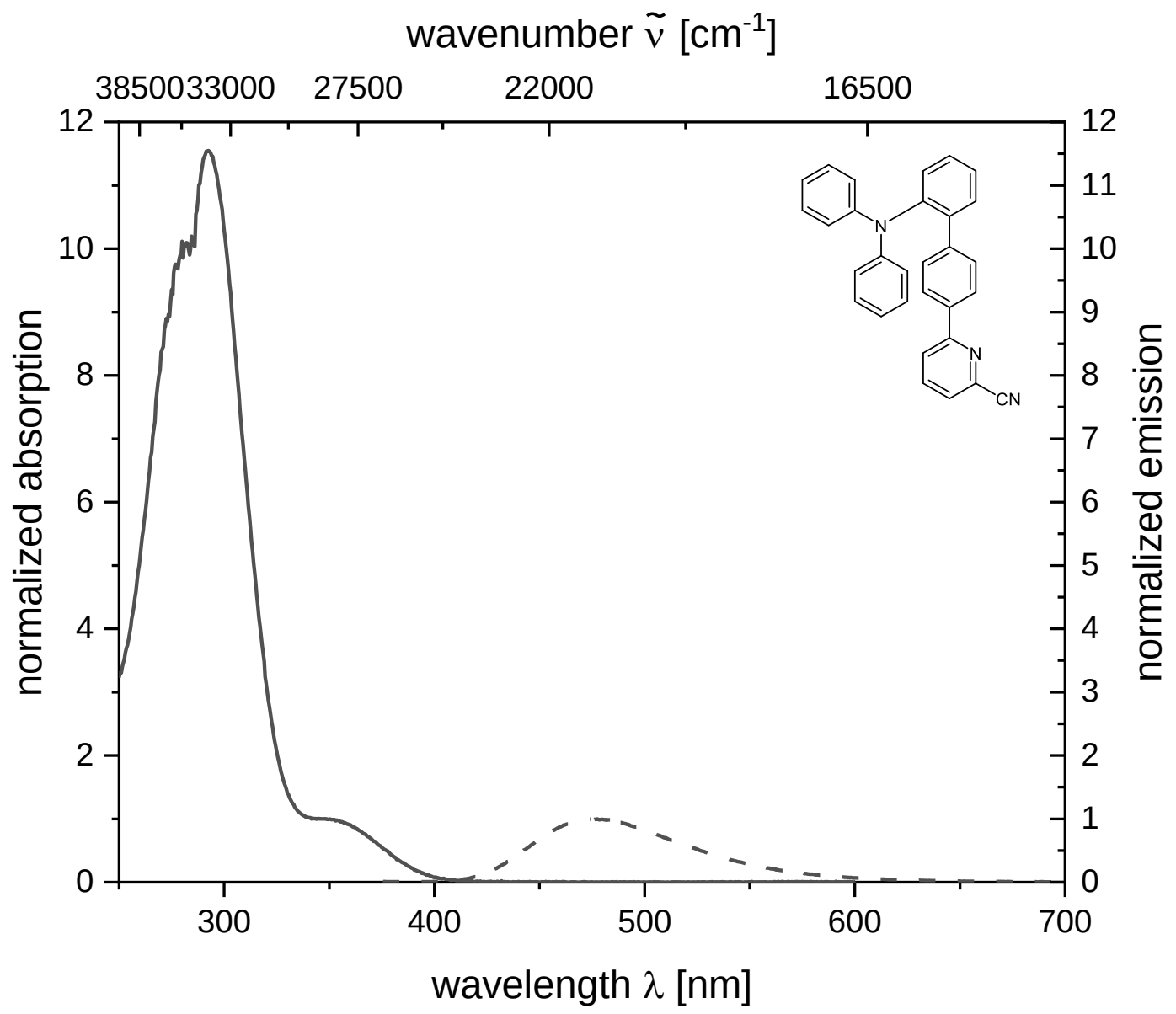

Figure S41. Normalized absorption (solid line) and emission spectrum (dashed line) of **6b** recorded in THF, T = 298 K, c_{abs}(**6b**) = 10^{-5} M, c_{em}(**6b**) = 10^{-6} M, $\lambda_{exc} = \lambda_{max}$.

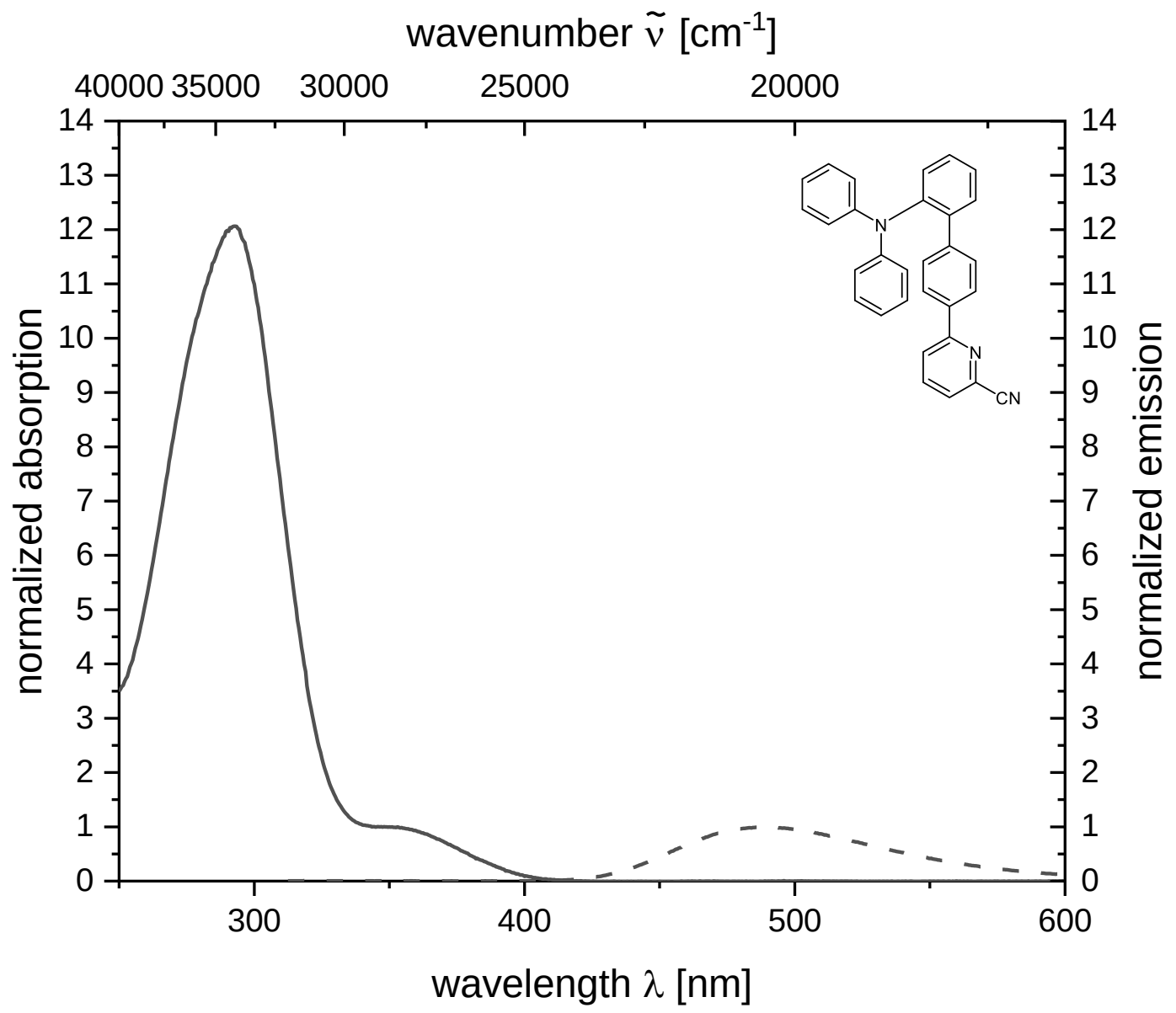

Figure S42. Normalized absorption (solid line) and emission spectrum (dashed line) of **6b** recorded in dichloromethane, T = 298 K, c_{abs}(**6b**) = 10^{-5} M, c_{em}(**6b**) = 10^{-6} M, $\lambda_{exc} = \lambda_{max}$.

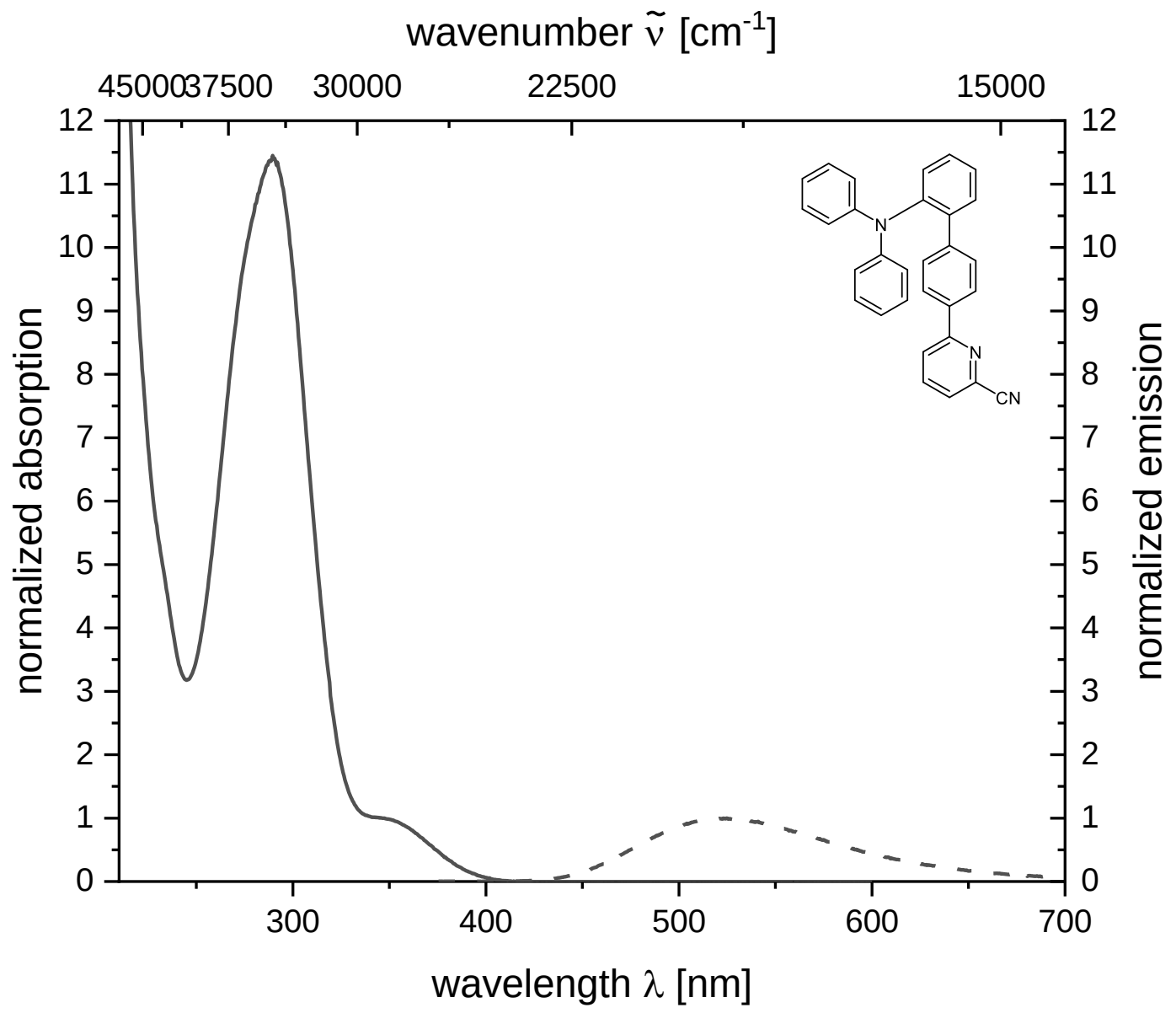

Figure S43. Normalized absorption (solid line) and emission spectrum (dashed line) of **6b** recorded in acetonitrile, T = 298 K, c_{abs}(**6b**) = 10^{-5} M, c_{em}(**6b**) = 10^{-6} M, $\lambda_{exc} = \lambda_{max}$.

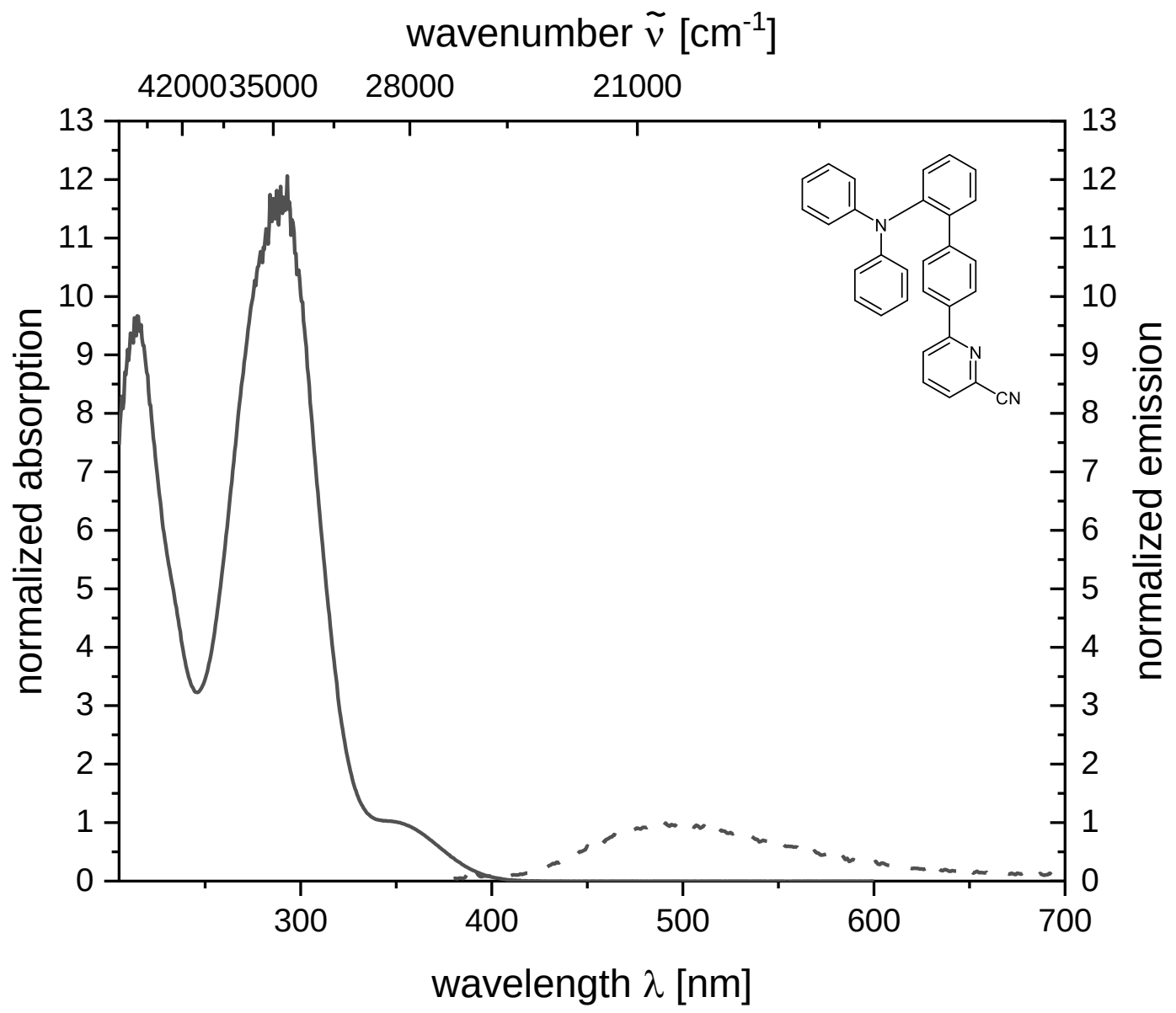

Figure S44. Normalized absorption (solid line) and emission spectrum (dashed line) of **6b** recorded in ethanol, T = 298 K, c_{abs}(**6b**) = 10^{-5} M, c_{em}(**6b**) = 10^{-6} M, $\lambda_{exc} = \lambda_{max}$.

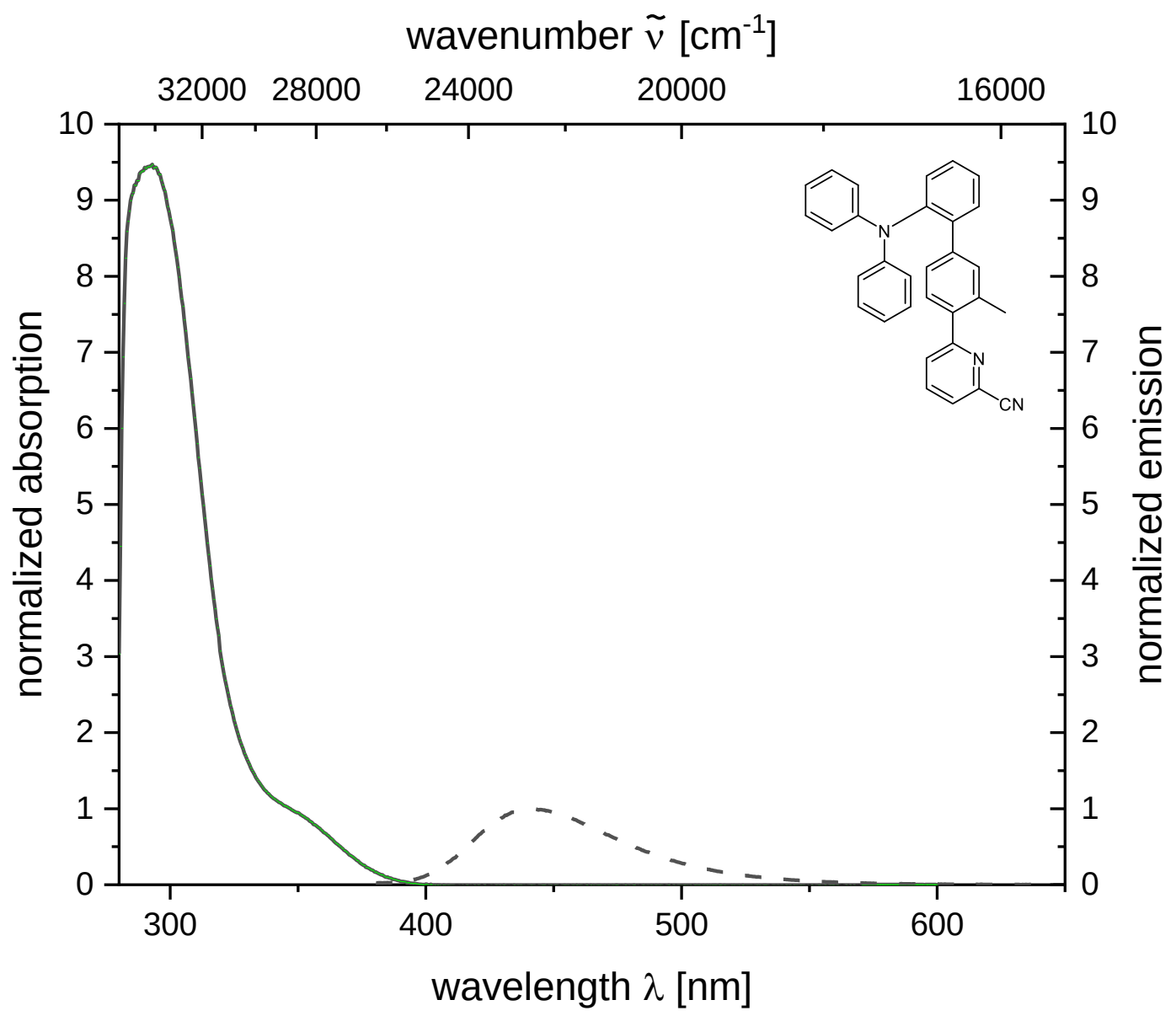

Figure S45. Normalized absorption (solid line) and emission spectrum (dashed line) of **6c** recorded in toluene, T = 298 K, c_{abs}(**6c**) = 10^{-5} M, c_{em}(**6c**) = 10^{-6} M, $\lambda_{exc} = \lambda_{max}$.

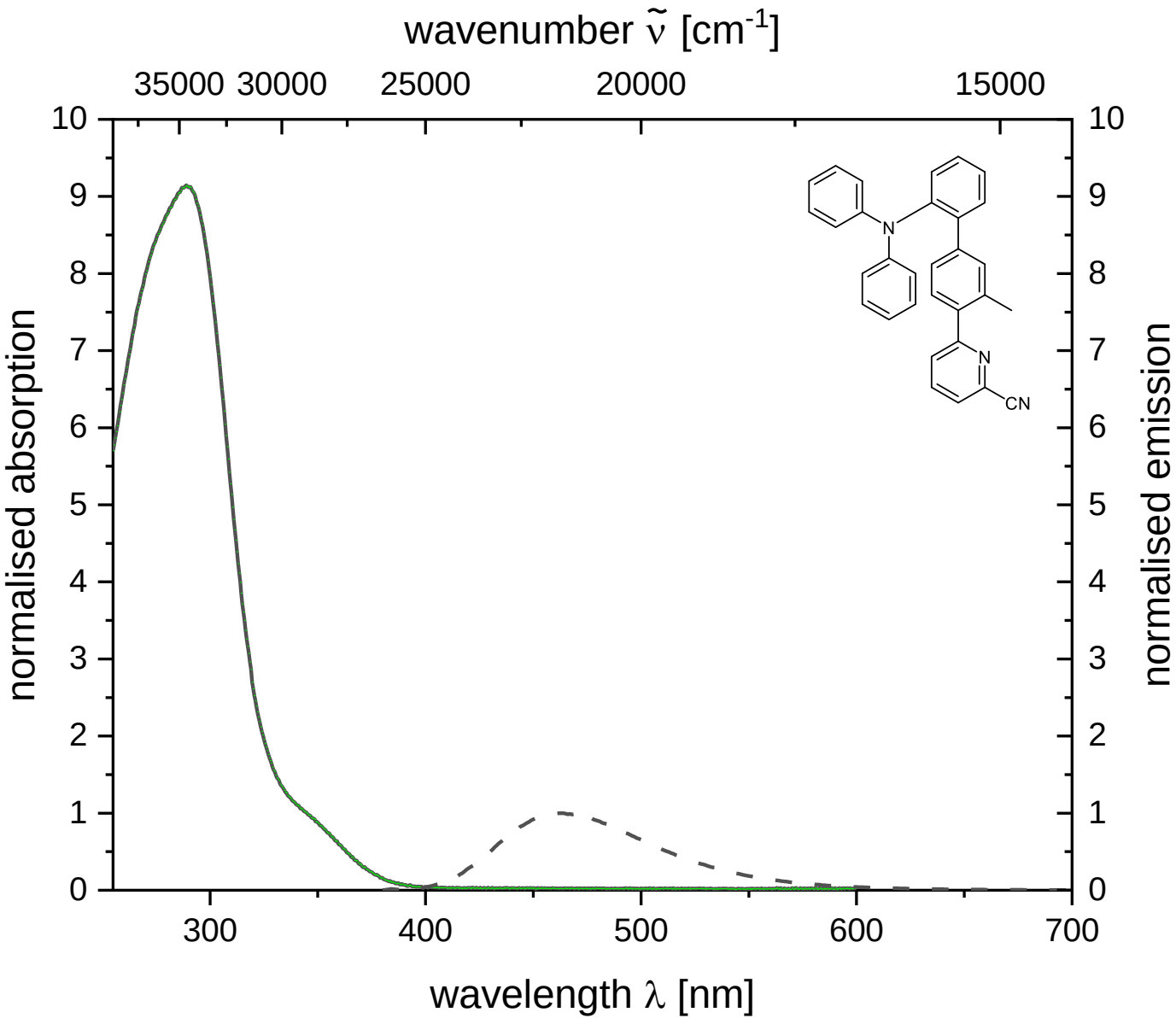

Figure S46. Normalized absorption (solid line) and emission spectrum (dashed line) of **6c** recorded in ethyl acetate, T = 298 K, c_{abs}(**6c**) = 10^{-5} M, c_{em}(**6c**) = 10^{-6} M, $\lambda_{exc} = \lambda_{max}$.

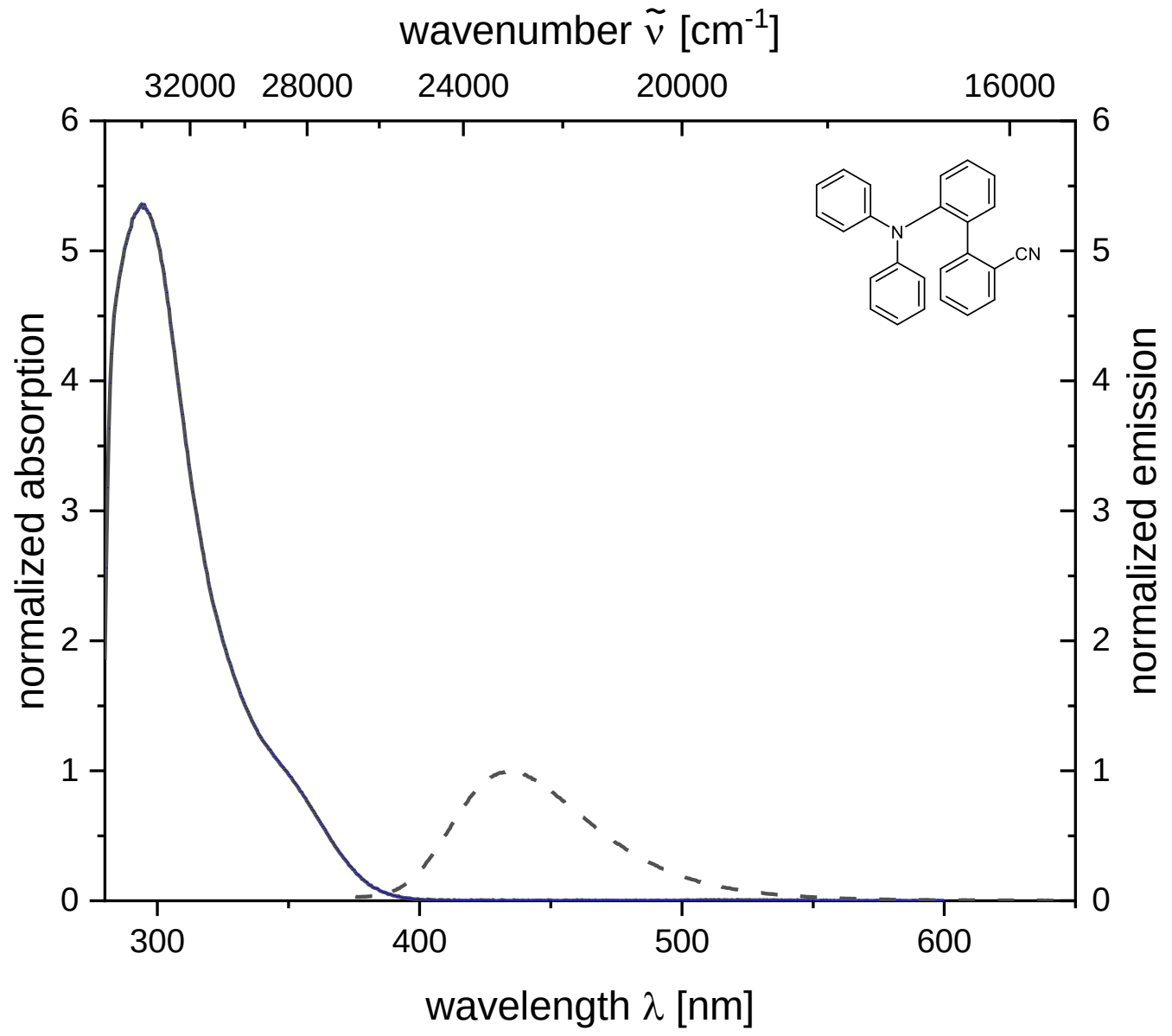

Figure S47. Normalized absorption (solid line) and emission spectrum (dashed line) of **7a** recorded in toluene, T = 298 K, c_{abs}(**7a**) = 10^{-5} M, c_{em}(**7a**) = 10^{-6} M, $\lambda_{exc} = \lambda_{max}$.

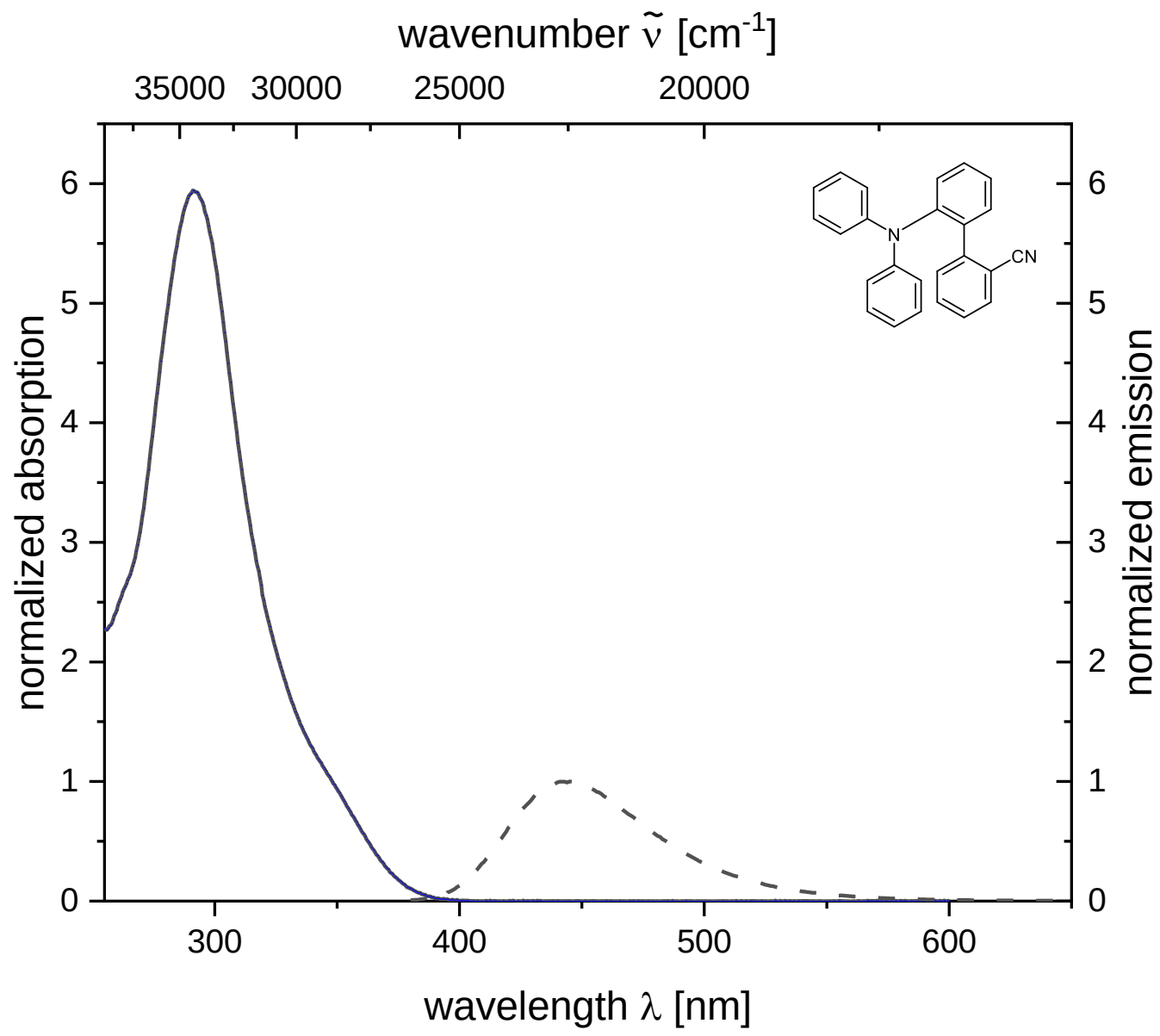

Figure S48. Normalized absorption (solid line) and emission spectrum (dashed line) of **7a** recorded in ethyl acetate, T = 298 K, c_{abs}(**7a**) = 10^{-5} M, c_{em}(**7a**) = 10^{-6} M, $\lambda_{exc} = \lambda_{max}$.

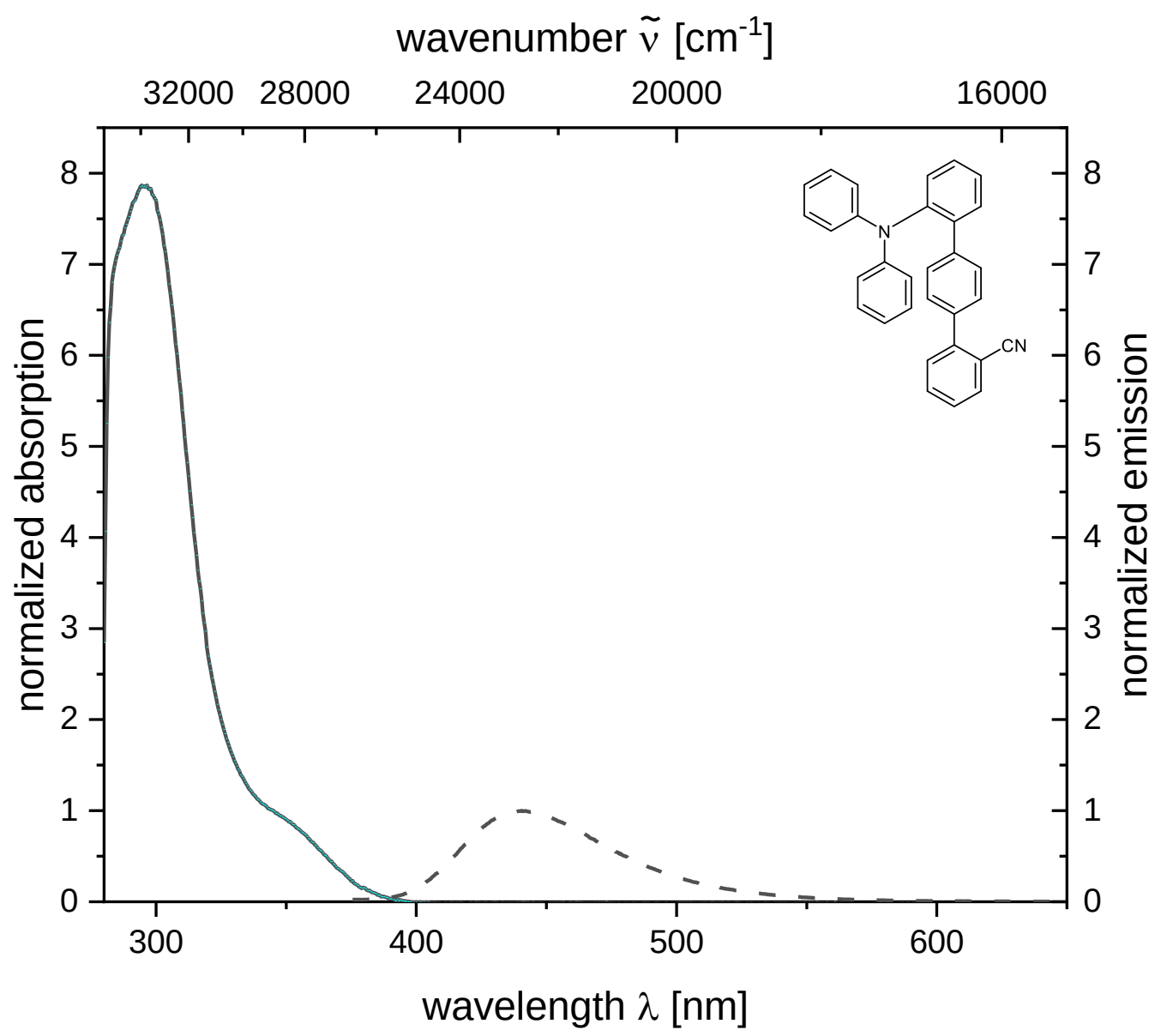

Figure S49. Normalized absorption (solid line) and emission spectrum (dashed line) of **7b** recorded in toluene, T = 298 K, c_{abs}(**7b**) = 10^{-5} M, c_{em}(**7b**) = 10^{-6} M, $\lambda_{exc} = \lambda_{max}$.

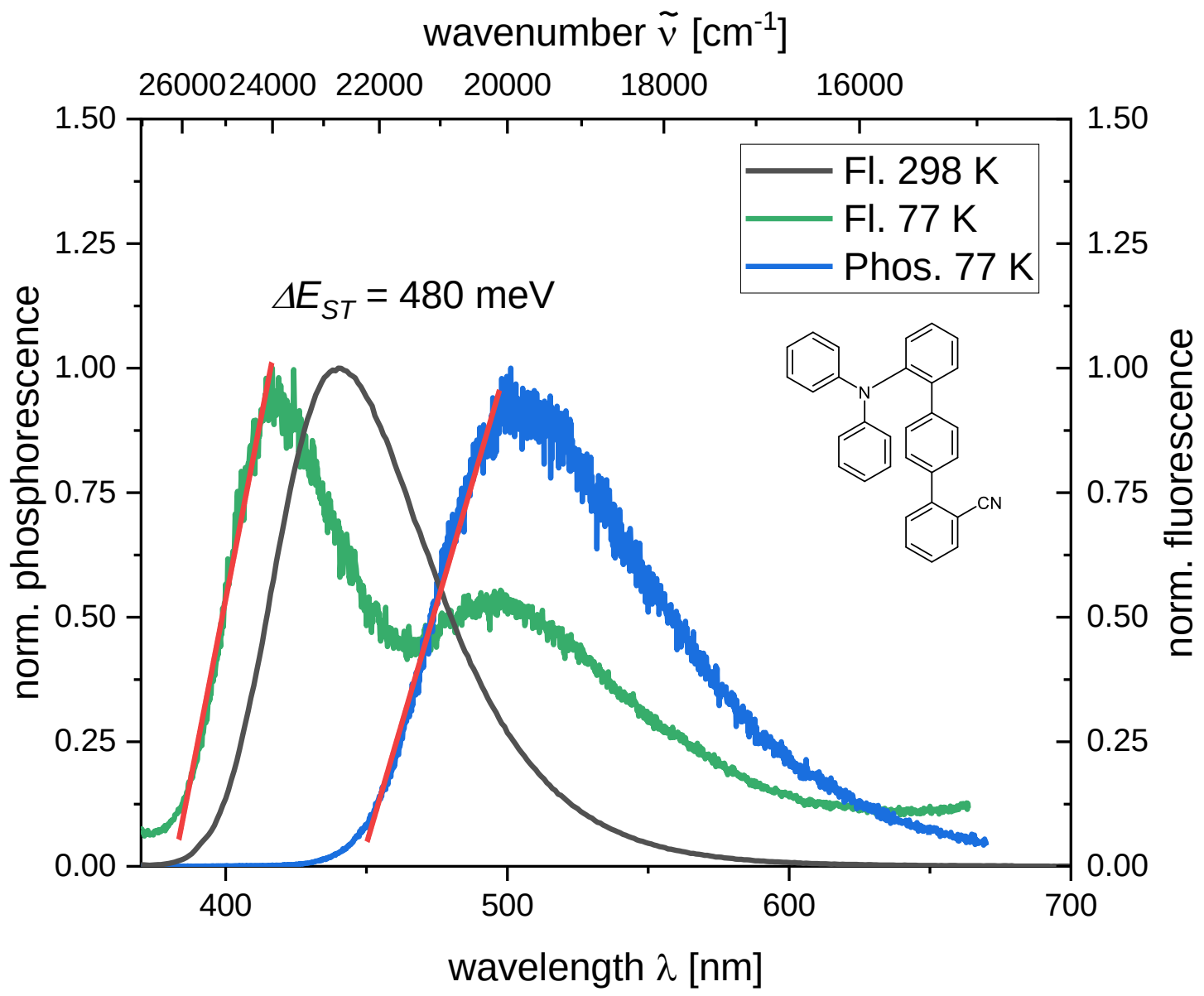

Figure S50. Normalized phosphorescence detected with a delay time of 1 µs (blue line, T = 77 K) and fluorescence spectrum (green (T = 77 K) and black (T = 298 K) line) of **7b** recorded in toluene, T = 298–77 K, c_{em}(**7b**) = 10^{-6} M, $\lambda_{exc} = \lambda_{max}$.

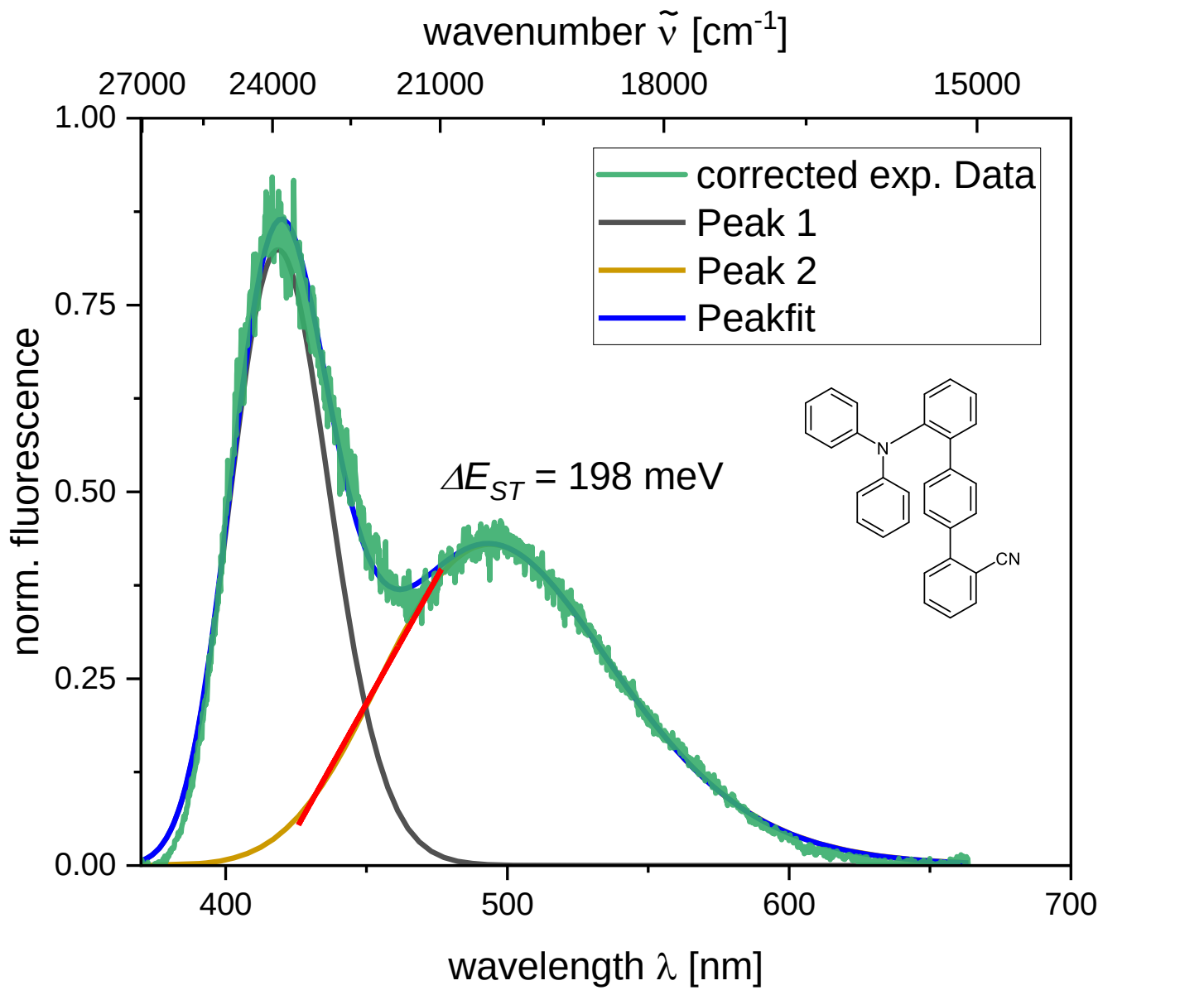

Figure S51. Normalized fluorescence spectrum (green) of **7b** recorded in toluene and peak analysis with gaussian curves (peak 1 black, peak 2 gold and peakfit blue), T = 77 K, c_{em}(**7b**) = 10^{-6} M, $\lambda_{exc} = \lambda_{max}$.

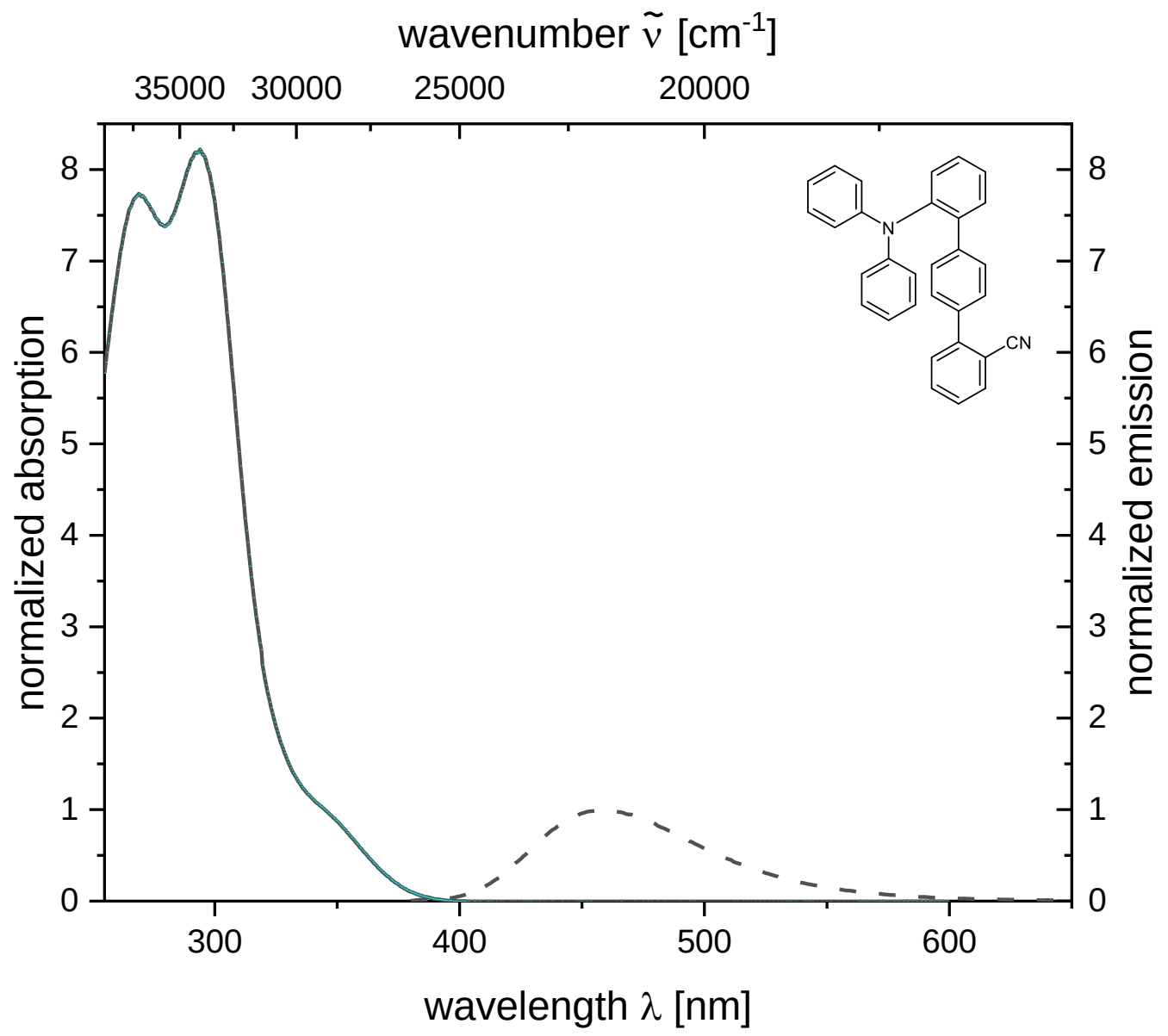

Figure S52. Normalized absorption (solid line) and emission spectrum (dashed line) of **7b** recorded in ethyl acetate, T = 298 K, c_{abs}(**7b**) = 10^{-5} M, c_{em}(**7b**) = 10^{-6} M, $\lambda_{exc} = \lambda_{max}$.

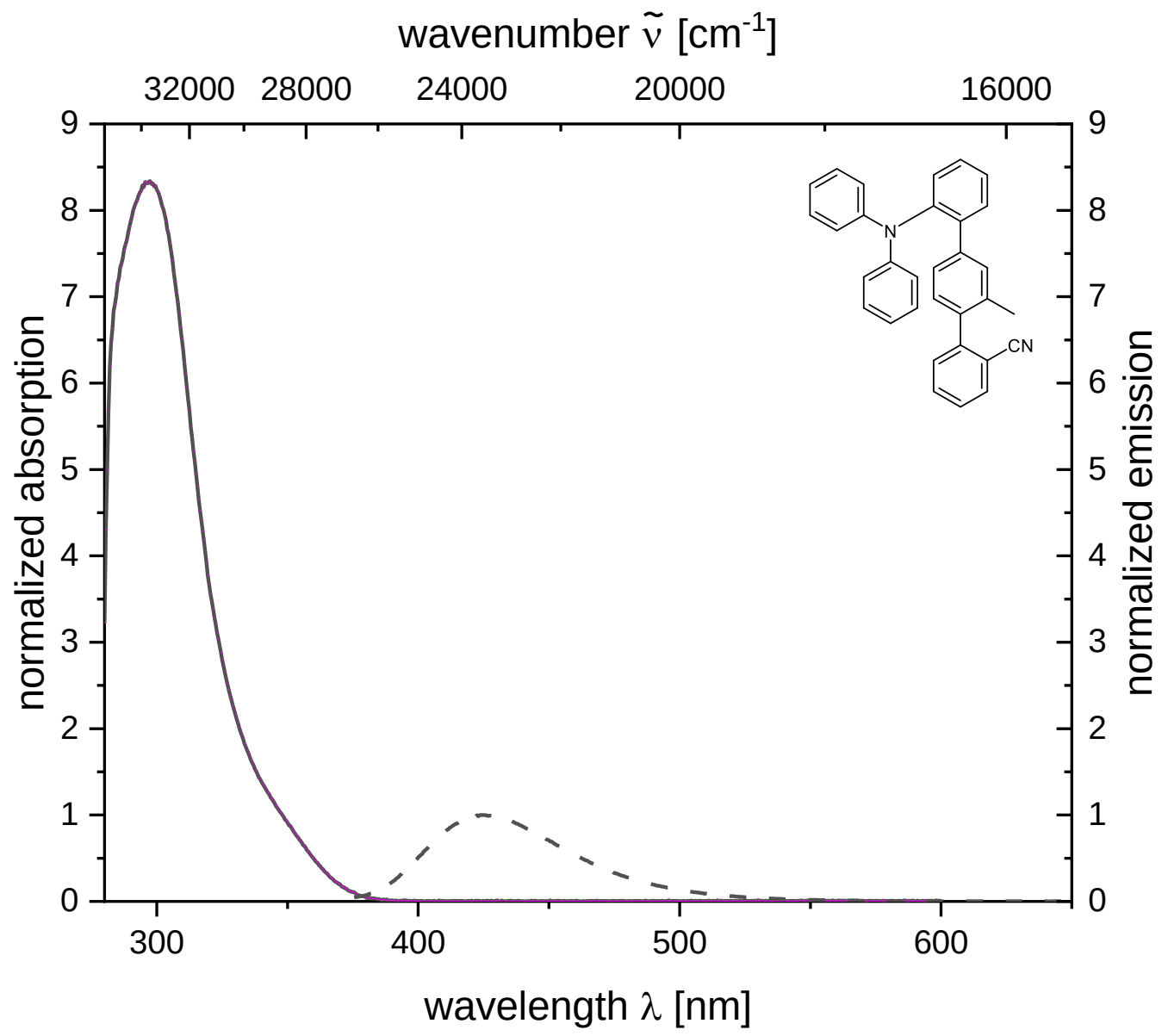

Figure S53. Normalized absorption (solid line) and emission spectrum (dashed line) of **7c** recorded in toluene, T = 298 K, c_{abs}(**7c**) = 10^{-5} M, c_{em}(**7c**) = 10^{-6} M, $\lambda_{exc} = \lambda_{max}$.

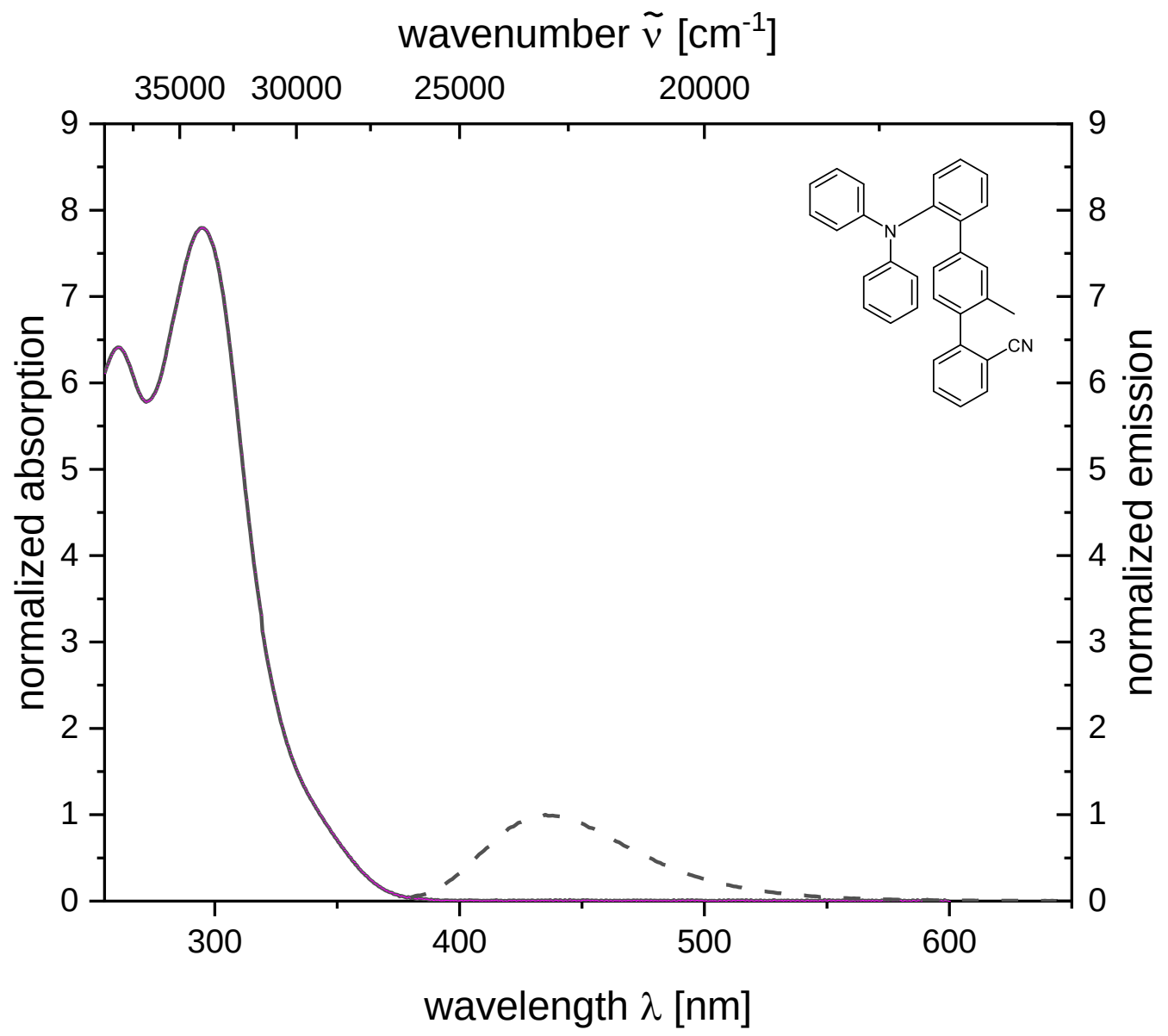

Figure S54. Normalized absorption (solid line) and emission spectrum (dashed line) of **7c** recorded in ethyl acetate, T = 298 K, c_{abs}(**7c**) = 10^{-5} M, c_{em}(**7c**) = 10^{-6} M, $\lambda_{exc} = \lambda_{max}$.

Table S5. Selected photophysical properties (absorption and emission maxima, photoluminescence quantum yields, FWHM, fluorescence lifetimes, radiative and non-radiative rate constants) of compound **6** and **7** in solution (toluene and ethyl acetate).

compound	$\lambda_{max,abs}$ / nm (ε / L · mol^{-1} · cm^{-1})[a]	$\lambda_{max,em}$ / nm (Φ_{PL})[b]	$\Delta\tilde{\nu}$ / cm^{-1} [c]	FWHM / cm^{-1} (nm)[d]	τ / ns (χ^2)[e]	k_r^{exp} / s^{-1} [f]	k_{nr}^{exp} / s^{-1} [g]
in toluene							
**6a**	291 (22500), 351sh (3800)	457 (0.18) 469sh	7200	4740 (99)	4.5, 10.8 (1.05)	–[h]	–[h]
**6b**	295 (39200), 353 (3900)	448 (0.29) (0.28)[i]	6000	3340 (67)	7.2 (1.04) 10.1 (1.10)[i]	$4.0\cdot10^7$ $2.8\cdot10^7$[i]	$9.9\cdot10^7$ $7.1\cdot10^7$[i]
**6c**	293 (43100), 347sh (4800)	442 (0.24)	6200	3380 (66)	6.9 (1.14)	$3.5\cdot10^7$	$1.1\cdot10^8$
**7a**	294 (17100), 349sh (3200)	434 (0.16)	5600	3240 (61)	5.8 (1.17)	$2.8\cdot10^7$	$1.4\cdot10^8$
**7b**	284sh (25400), 296 (29200), 345sh (3700)	441 (0.21) (0.28)[i]	6300	3340 (65)	6.5 (1.04) 9.4 (1.08)[i]	$3.2\cdot10^7$ $3.0\cdot10^7$[i]	$1.2\cdot10^8$ $7.7\cdot10^7$[i]
**7c**	297 (29900), 348sh (4100)	424 (0.19)	5200	3390 (61)	5.1 (1.09)	$3.7\cdot10^7$	$1.6\cdot10^8$
in ethyl acetate							
**6a**	268sh (24900), 287 (32200), 350sh (5800)	452sh 485 (0.09)	8000	4250 (100)	8.7 (1.13)	$1.0\cdot10^7$	$1.0\cdot10^8$
**6b**	291 (46400), 349sh (4100)	474 (0.16)	7600	3740 (84)	7.9 (1.19)	$2.0\cdot10^7$	$1.1\cdot10^8$
**6c**	274sh (26000), 289 (28300), 345sh (2900)	463 (0.25)	7400	3830 (82)	7.7 (1.06)	$3.2\cdot10^7$	$9.7\cdot10^7$
**7a**	263sh (7700), 292 (18500), 348sh (3100)	445 (0.15)	6300	3430 (68)	7.0 (1.09)	$2.1\cdot10^7$	$1.2\cdot10^8$
**7b**	269 (41300), 294 (43900), 345sh (5500)	458 (0.16)	7200	3340 (70)	7.4 (1.08)	$2.2\cdot10^7$	$1.1\cdot10^8$
**7c**	261 (17600), 295 (21200), 343sh (2800)	435 (0.15)	6200	3650 (69)	6.0 (1.12)	$2.5\cdot10^7$	$1.4\cdot10^8$

[a] Recorded in different solvents, T = 293 K, c_{abs} = 10^{-5} M. [b] Recorded in different solvents, T = 293 K, c_{em} = 10^{-7} M, absolute quantum yields determined using an integrating sphere, λ_{exc} = $\lambda_{max,abs}$. [c] $\Delta\tilde{\nu} = \left(\frac{1}{\lambda_{max,abs}} - \frac{1}{\lambda_{max,em}}\right) \cdot 10^7$ (λ_{max} in nm) [cm^{-1}]. [d] FWHM [cm^{-1}] determined using $10^7 \cdot$ (FWHM [nm]/(λ_{em} [nm])^2). [e] Residuals χ^2. [f] $k_r^{exp} = \frac{\Phi_{PL}}{\tau}$. [g] $k_{nr}^{exp} = \frac{1-\Phi_{PL}}{\tau}$.[h] Can not be determined. [i] Sample degassed with nitrogen for one hour prior to measurement.

5.3 Emission spectra in a PMMA matrix

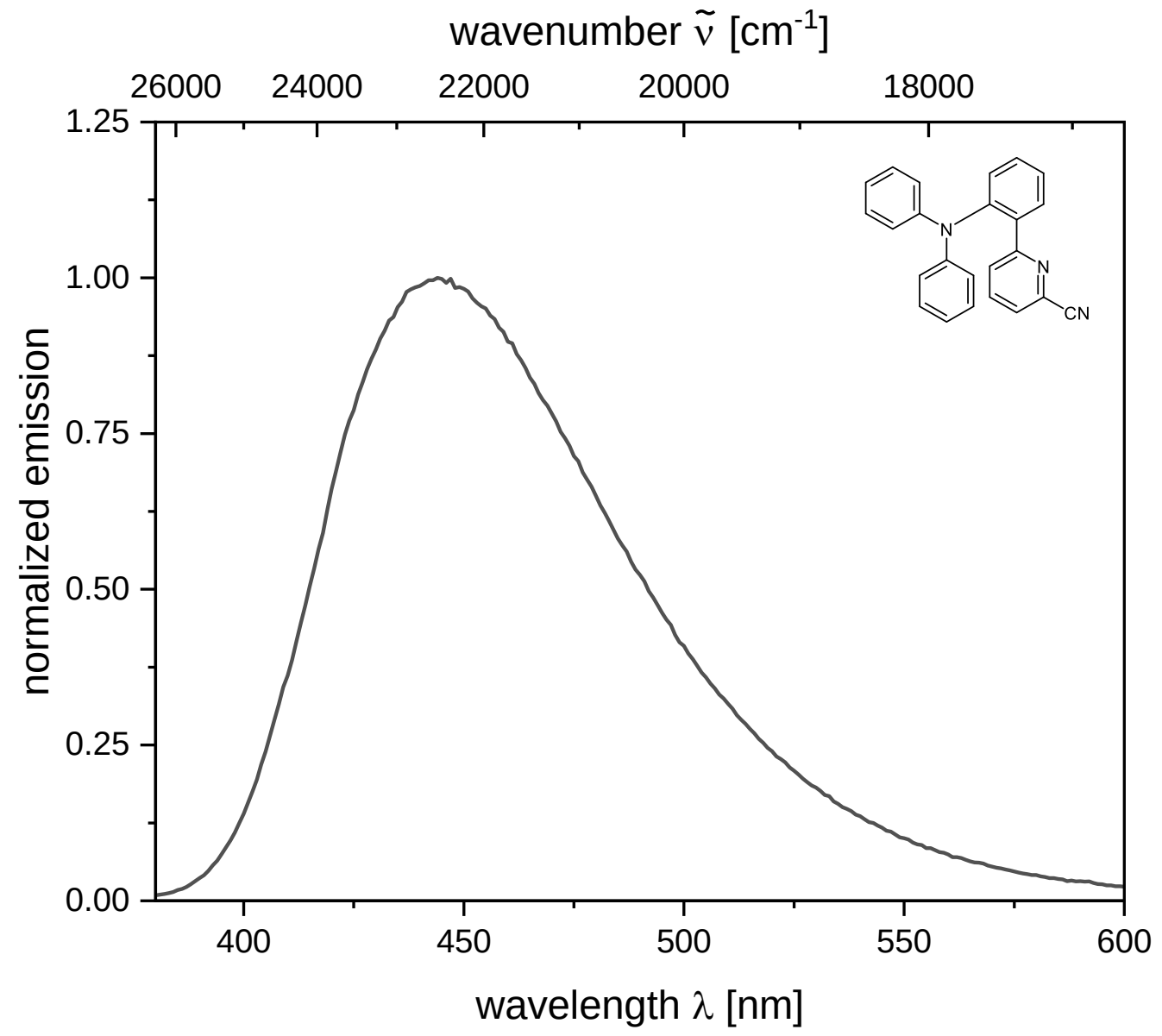

Figure S55. Normalized emission spectrum of **6a** recorded in a PMMA matrix, T = 298 K, c(**6a**) = 1 wt%, λ_{exc} = 351 nm.

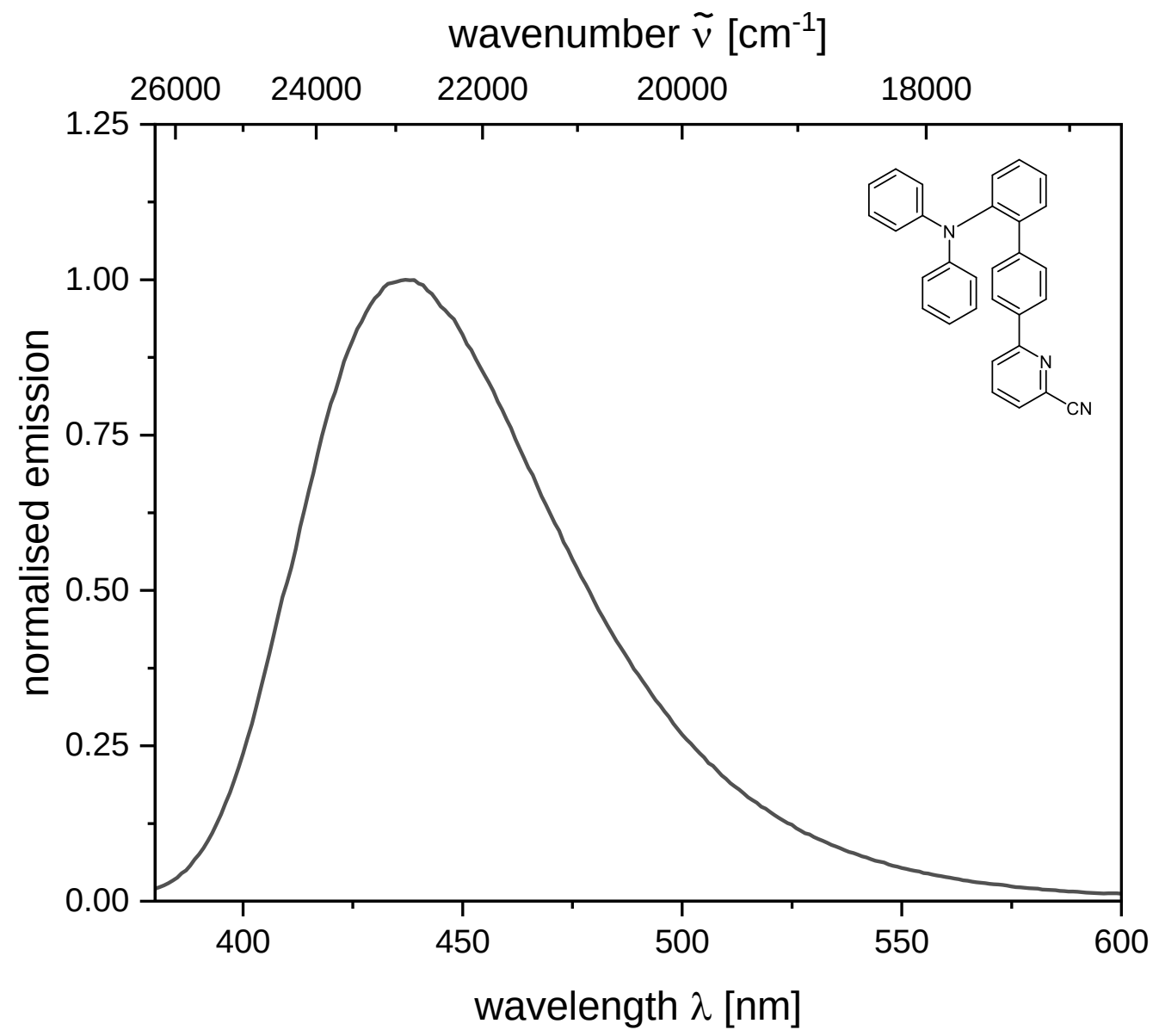

Figure S56. Normalized emission spectrum of **6b** recorded in a PMMA matrix, T = 298 K, c(**6b**) = 1 wt%, λ_{exc} = 353 nm.

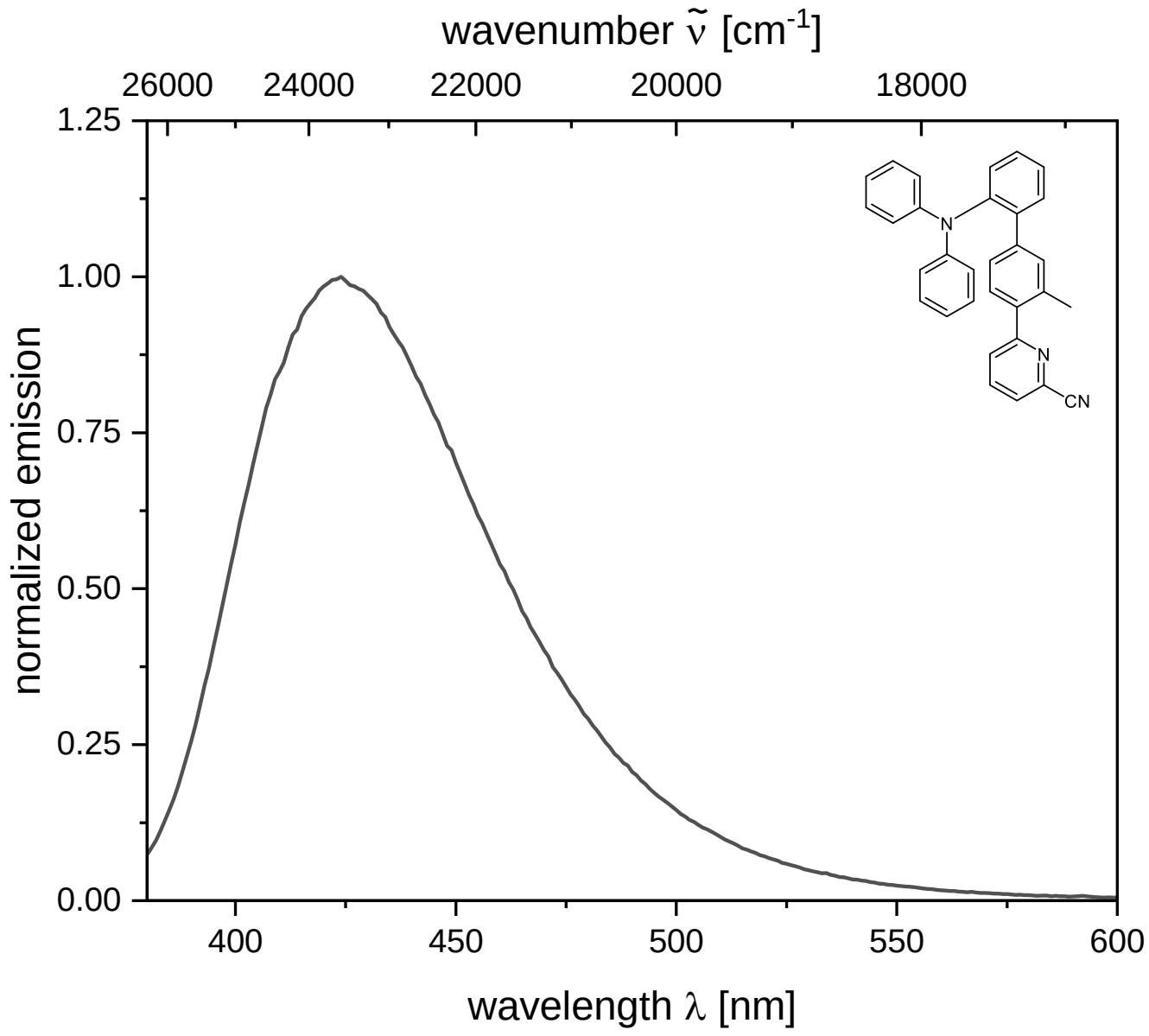

Figure S57. Normalized emission spectrum of **6c** recorded in a PMMA matrix, T = 298 K, c(**6c**) = 1 wt%, λ_{exc} = 347 nm.

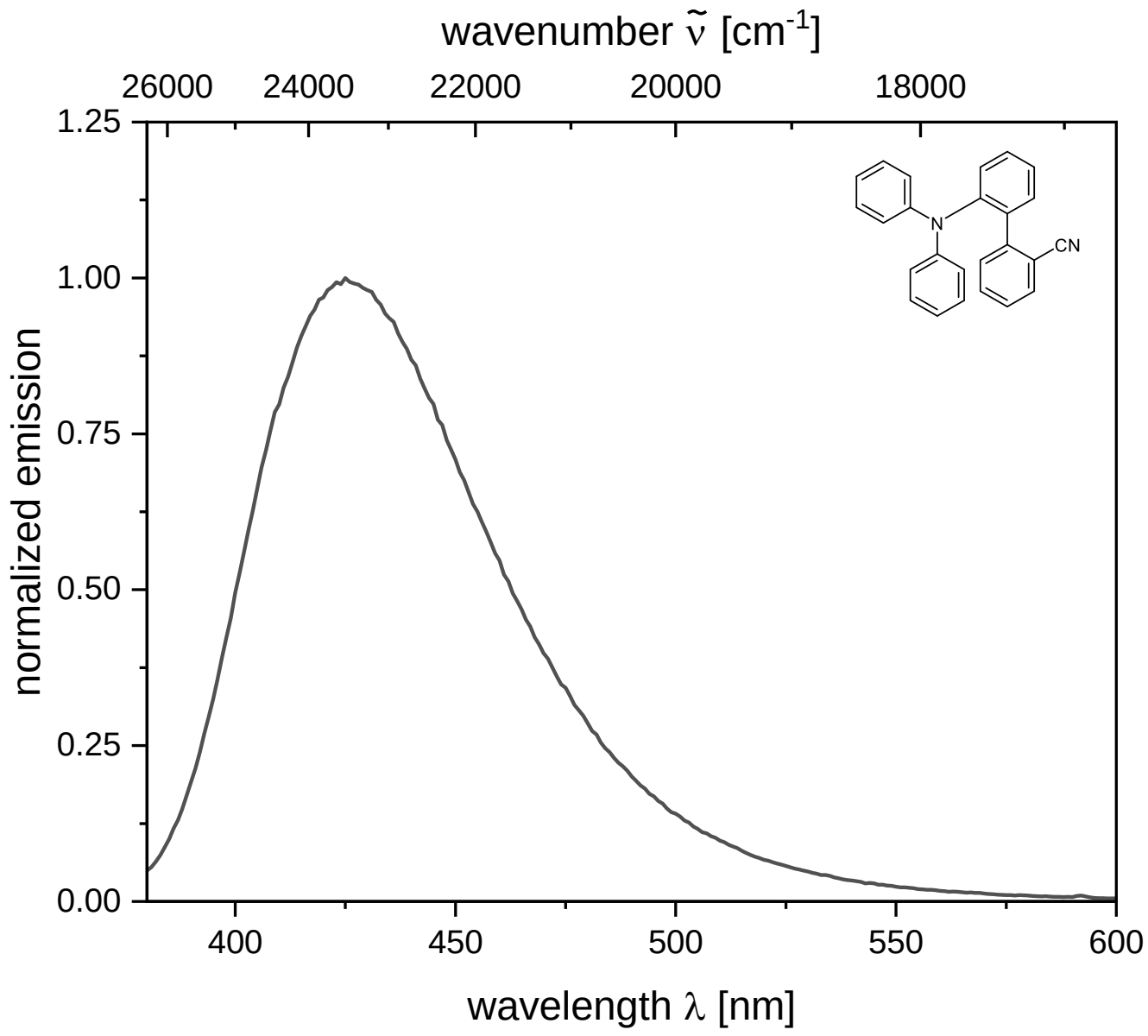

Figure S58. Normalized emission spectrum of **7a** recorded in a PMMA matrix, T = 298 K, c(**7a**) = 1 wt%, λ_{exc} = 349 nm.

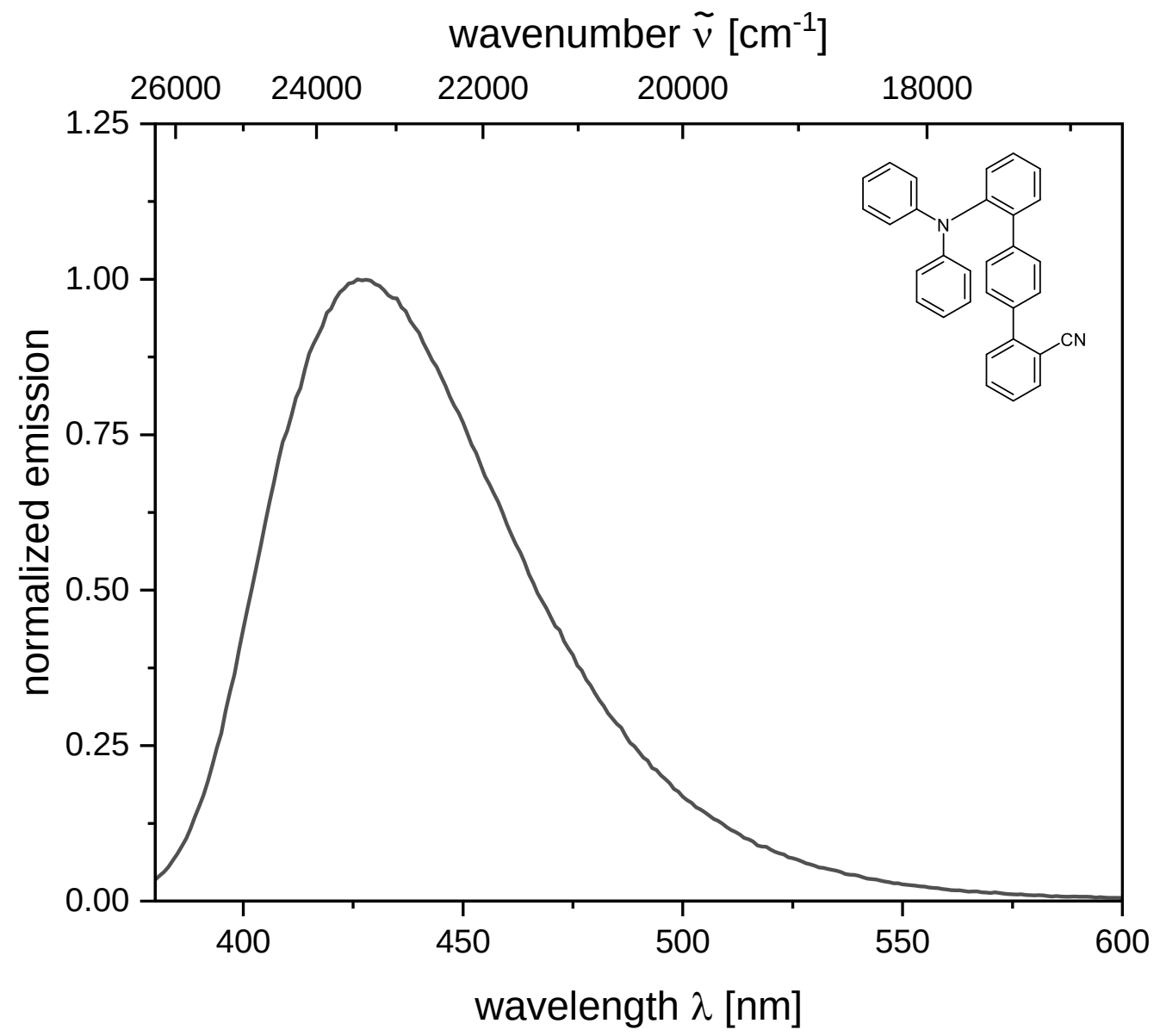

Figure S59. Normalized emission spectrum of **7b** recorded in a PMMA matrix, T = 298 K, c(**7b**) = 1 wt%, λ_{exc} = 345 nm.

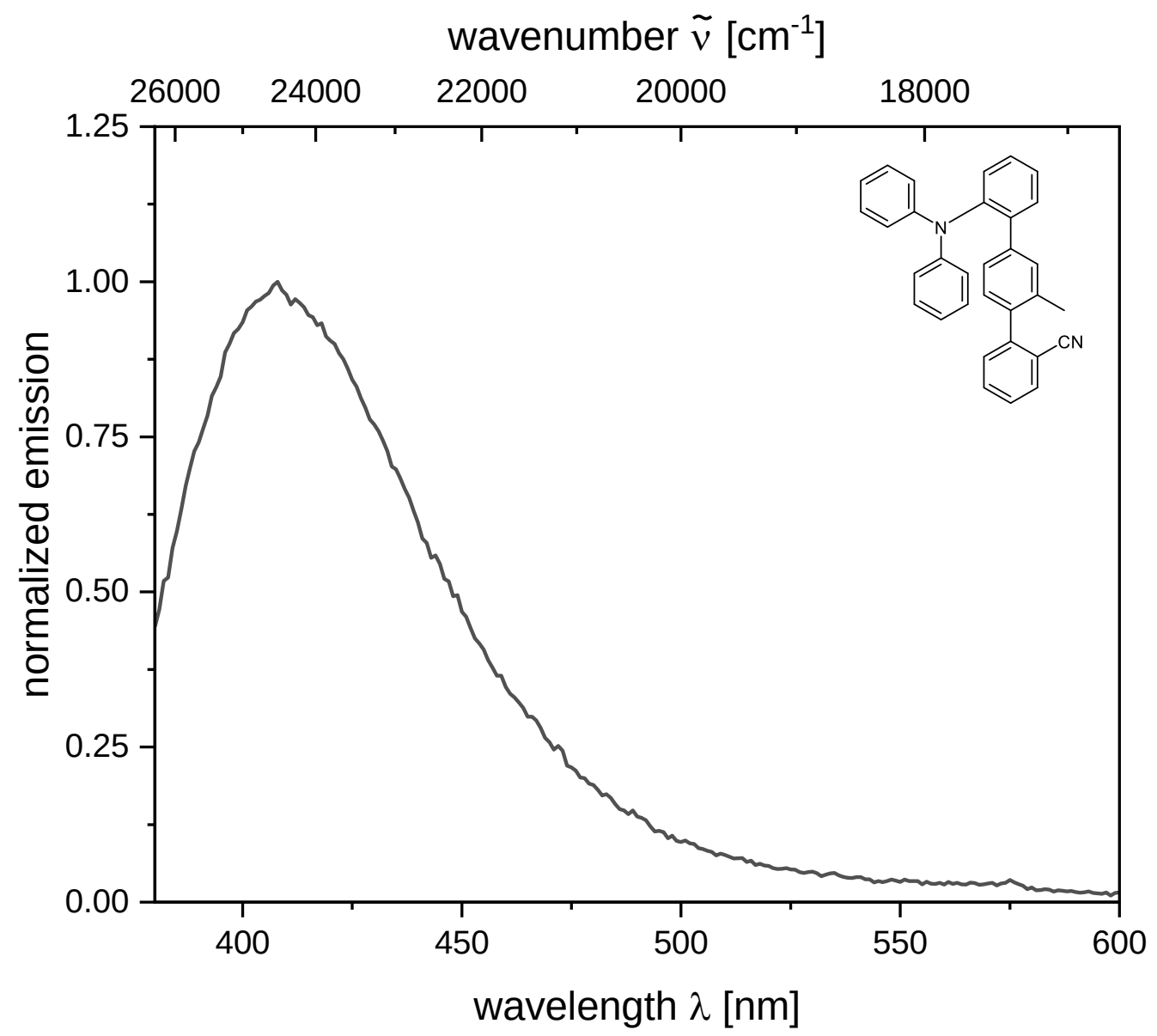

Figure S60. Normalized emission spectrum of **7c** recorded in a PMMA matrix, T = 298 K, c(**7c**) = 1 wt%, λ_{exc} = 348 nm.

Table S6. Selected photophysical properties (emission maxima, photoluminescence quantum yields, FWHM, fluorescence lifetimes, and CIE 1931 coordinates) of compound **6** and **7** in a PMMA matrix (T = 293 K, $c_{comp.}$ = 1 wt%).

compound	$\lambda_{max,em}$ / nm (Φ_{PL})[a]	FWHM / cm^{-1} (nm)[b]	τ / ns (χ^2)[c]	CIE 1931 (x,y)
**6a**	444 (0.26)	3860 (76)	7.5, 14.9 (1.04)	0.155, 0.109
**6b**	438 (0.33)	3540 (68)	5.7, 11.6 (1.14)	0.154, 0.078
**6c**	424 (0.29)	3450 (62)	4.9, 10.2 (1.19)	0.156, 0.053
**7a**	426 (0.19)	3360 (61)	4.7, 10.5 (1.19)	0.156, 0.056
**7b**	428 (0.27)	3490 (64)	5.4, 11.3 (1.18)	0.155, 0.057
**7c**	408 (0.45)	3300 (55)	3.5, 7.6 (1.15)	0.166, 0.064

[a] Emission maxima and absolute photoluminescence quantum yield Φ_{PL} determined with an integrating sphere (matrix sample, T = 293 K). [b] FWHM [cm^{-1}] determined using $10^7 \cdot$ (FWHM [nm]/(λ_{em} [nm])^2). [c] Residuals χ^2.

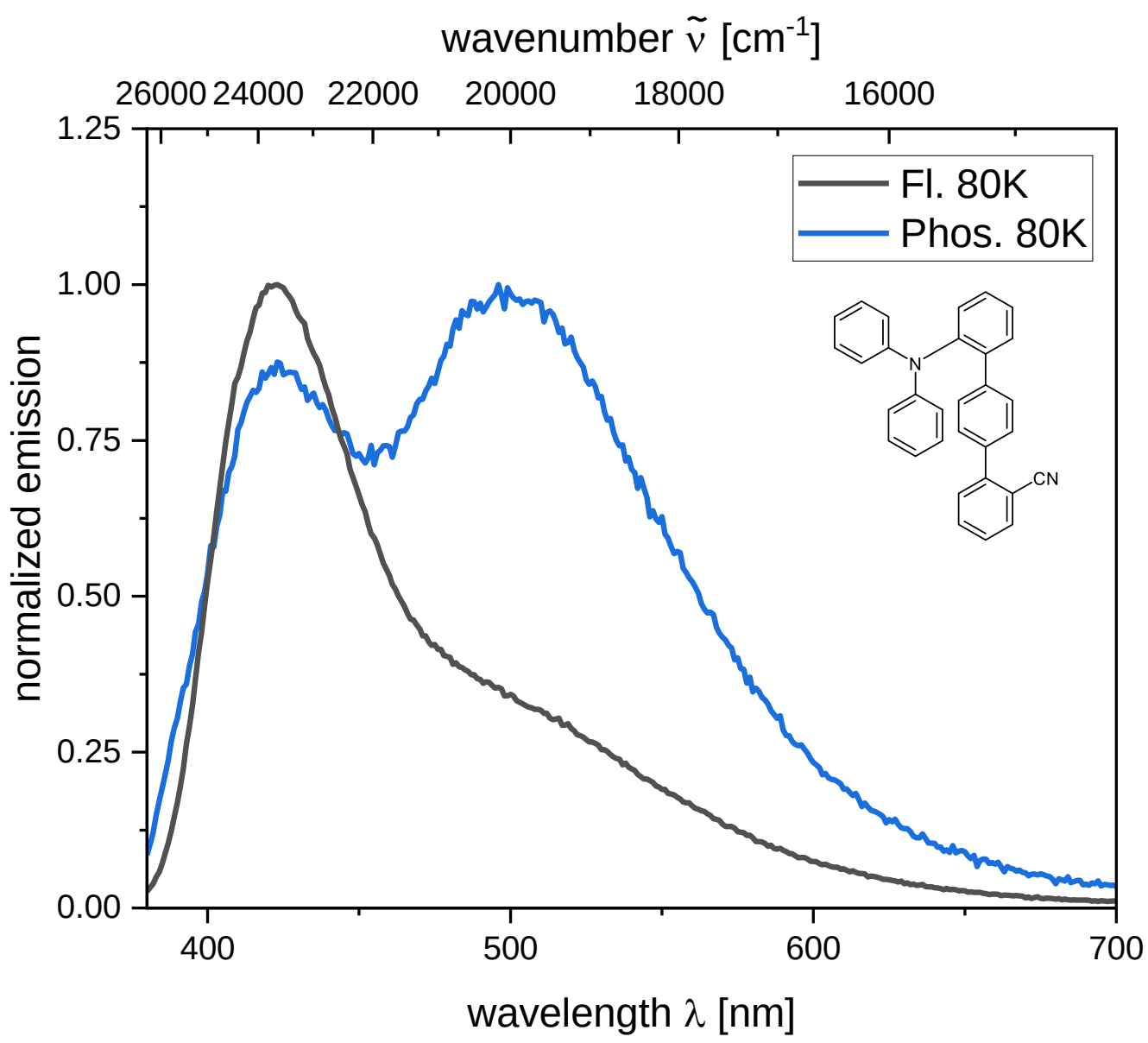

Figure S61. Normalized emission spectrum of the photoluminescence behavior predominantly fluorescence Fl. (black) and gated emission predominantly phosphorescence Phos. detected with a delay time of 1 µs in the range of 1–10000 µs (blue) of **7b** recorded in a PMMA matrix, T = 80 K, c(**7b**) = 1 wt%, λ_{exc} = 345 nm.

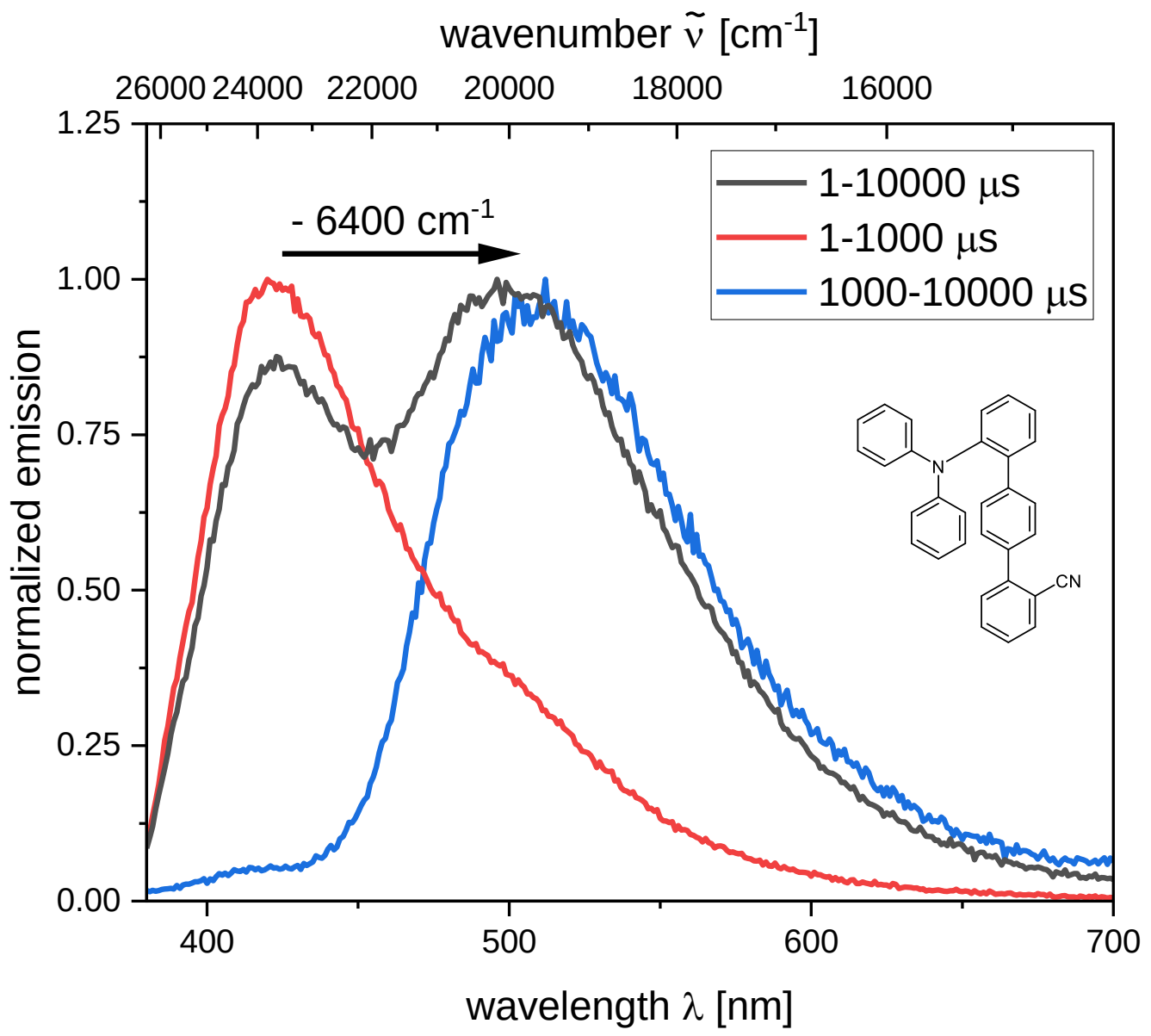

Figure S62. Normalized gated emission spectra in different detection delay times in the range of 1–10000 µs (black), 1–1000 µs (red) and 1000–10000 µs (blue) of **7b** recorded in a PMMA matrix, T = 80 K, c(**7b**) = 1 wt%, λ_{exc} = 345 nm.

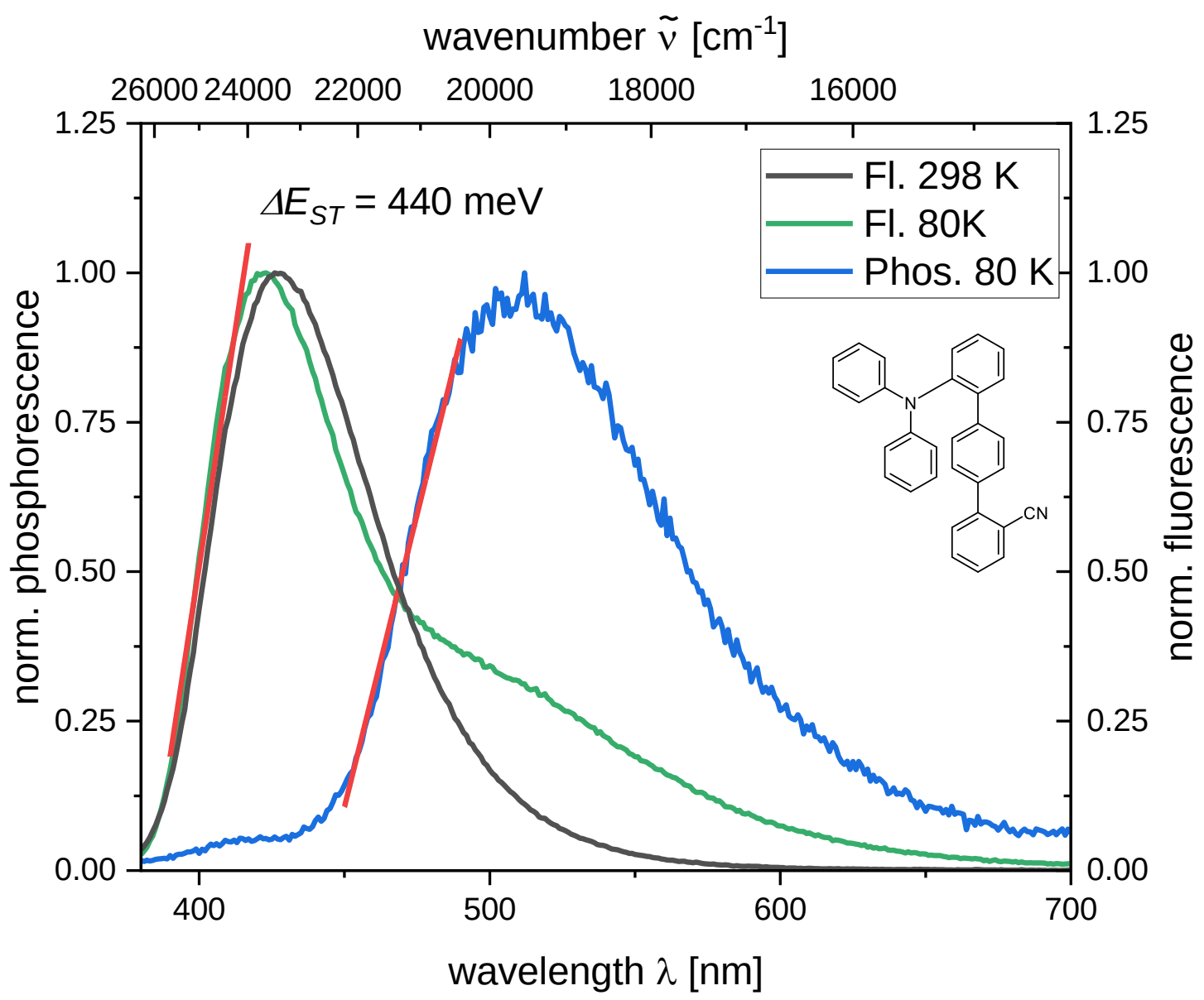

Figure S63. Normalized emission spectrum of the photoluminescence behavior predominantly fluorescence Fl. (black, T = 298 K and green T = 80 K) and gated emission predominantly phosphorescence Phos. detected with a delay time of 1 µs in the range of 1000–10000 µs (blue) of **7b** recorded in a PMMA matrix, T = 80 K, c(**7b**) = 1 wt%, λ_{exc} = 345 nm.

Table S7. Temperature resolved emission data of compound **7b** recorded in a PMMA matrix, T = 300–80 K, c(**7b**) = 1 wt%, λ_{exc} = 345 nm.

temperature [K]	$\lambda_{max,em}$ [nm]	relative peak intensity	difference peak intensity relative to value at 300 K
**300**	428	93020	0
**260**	426	96650	+3630
**220**	425	97240	+4220
**180**	423	92230	–790
**140**	422	91810	–1210
**100**	424	87360	–5660
**80**	423	78840	–14180

5.4 Rotamer analysis

To investigate the possible contribution of different conformers to the photophysical behavior in frozen solution and PMMA matrix, the rotational potentials of both the acceptor moiety (Figure S64) and the phenylene-bridged acceptor unit (Figure S65) are examined by relaxed potential energy surface scans in 10 degree increments using Gaussian 16[3] (BMK/6-31+G*) in the gas phase. Rotation of the acceptor unit reveals a single global minimum, whereas rotation of the phenylene-bridged acceptor unit results in two distinct minima of comparable energy. Since charge density difference (CDD) analysis indicates that the longest wavelength absorption exhibit pronounced charge transfer character between the triphenylamine donor and the phenylene-bridged acceptor moiety (see Figure S85), the rotational coordinate is investigated in greater detail. The two minima identified from the rotational scan (Figure S65) are subsequently reoptimized in toluene using the same level of theory and confirmed as true minima by vibrational frequency analysis (NImag = 0).

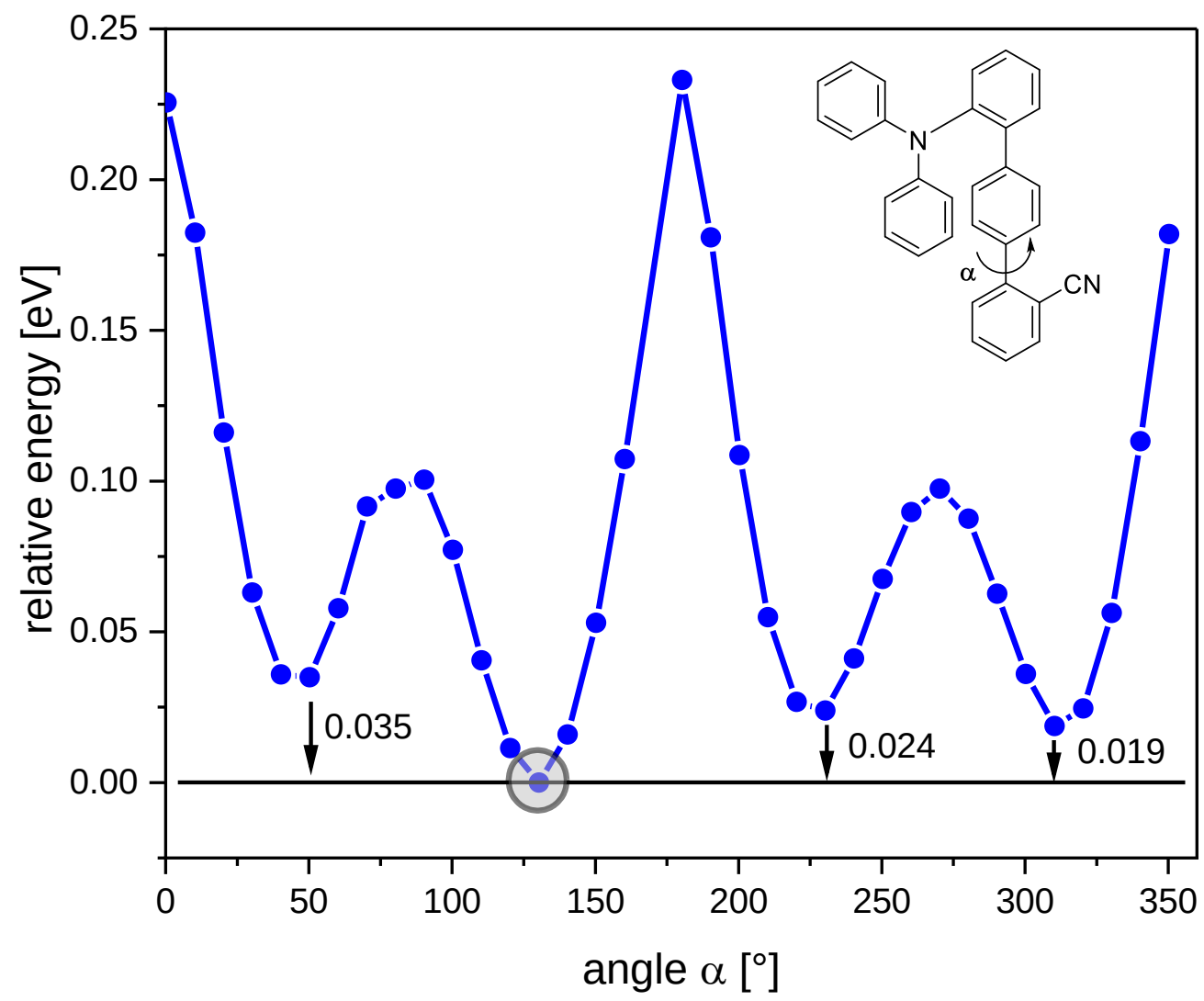

Figure S64. Rotational profile of compound **7b** along the torsion angle α, rotation of the acceptor unit. Relative energy compared to the global minimum at 0 eV (most stable rotamer marked grey). Calculated values using Gaussian 16 (BMK/6-31+G*, gasphase).

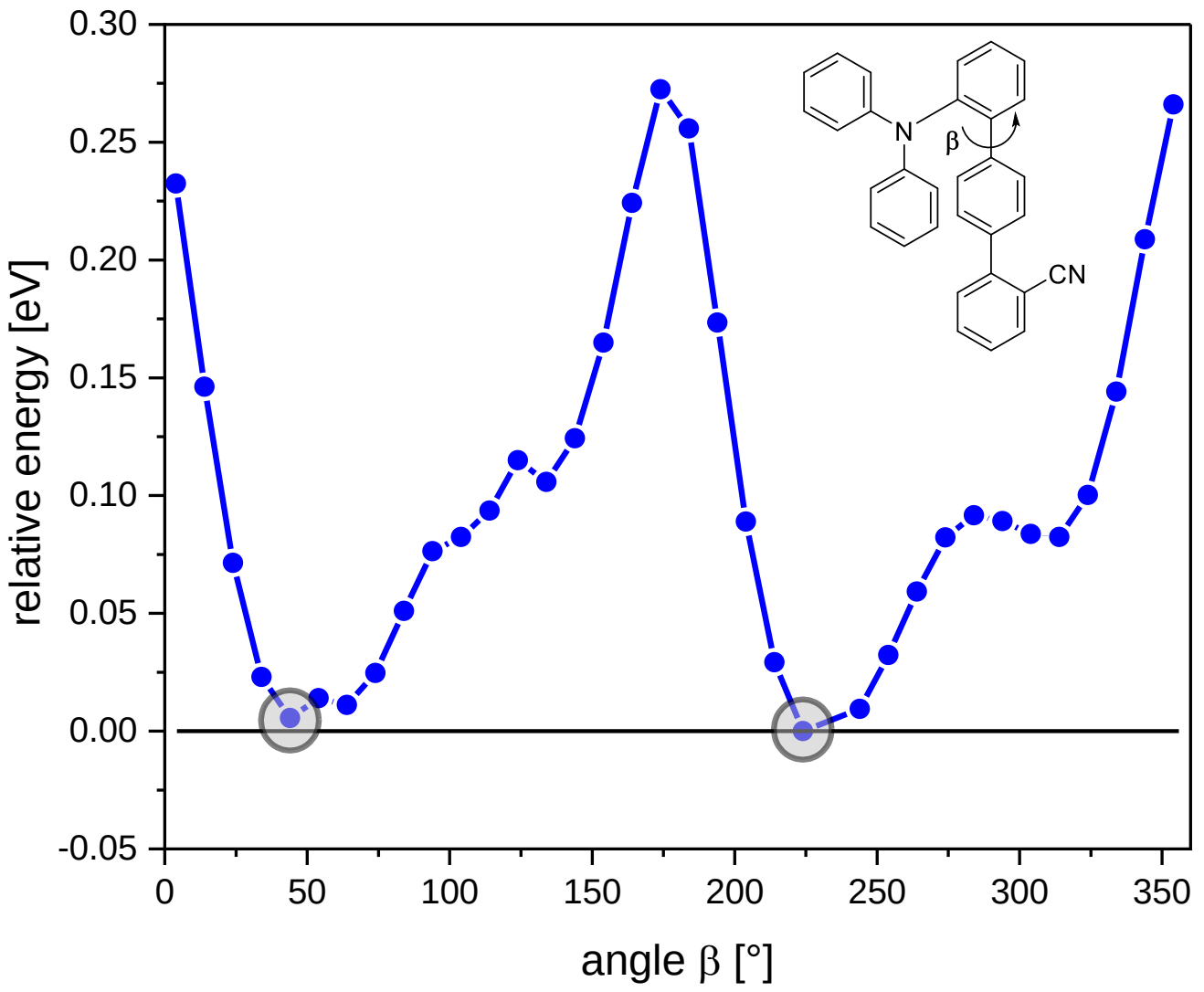

Figure S65. Rotational profile of compound **7b** along the torsion angle β, rotation of the bridged acceptor unit. Relative energy compared to the global minimum at 0 eV (most two stable rotamers marked grey). Calculated values using Gaussian 16 (BMK/6-31+G*, gasphase).

Geometry optimizations in toluene identified two stable rotamers. Rotamer 2 represents the global minimum, while rotamer 1 is higher in free energy by 3.80 kJ/mol (equation 1).

$$\Delta G = G_1 - G_2 = 0.001448\, Hartree = 3.80\, kJ/mol \tag{1}$$

Based on the Gibbs free energy difference, the Boltzmann distribution at 298 K predicts populations of around 82% for rotamer 2 and 18% for rotamer 1, indicating that both conformers are populated at room temperature (equation 2–5).[8] Rapid cooling into a frozen glass is expected to suppress conformational interconversion, thereby preserving the room temperature rotamer distribution.

$$\frac{\Delta G}{RT} = \frac{3800\, J/mol}{2478\, J/mol} = 1.535 \tag{2}$$

$$e^{-1.535} + e^{0} = 0.215 + 1 = 1.215 \tag{3}$$

$$p_1 = \frac{0.215}{1.215} = 0.177 \tag{4}$$

$$p_2 = \frac{1}{1.215} = 0.823 \tag{5}$$

To evaluate the interconversion barrier between the two rotamers, the transitional state (TS) is optimized using the maximum obtained from the rotamer analysis as initial geometry (Figure S65). The global maximum is confirmed by frequency analysiscas a first order saddle point (NImag = 1), and visualization of the imaginary normal mode verified it as the rotation of the phenylene bridge. The Gibbs free activation energy for the rotation is calculated relative to the more stable rotamer 2 and amounts to around 8 kcal/mol (equation 6).

$$\Delta G^{\ddagger} = G_{TS} - G_2 = 0.012654\, Hartree = 33.22\frac{kJ}{mol} = 7.94\frac{kcal}{mol} \tag{6}$$

The calculated rotational barrier of around 8 kcal/mol is sufficiently low to allow rapid interconversion between the two rotamers at room temperature in a dynamic equilibrium.[9] In contrast, at low

temperature like 77 K, the rate of interconversion is expected to decrease drastically, leading to effective conformational freezing. Consequently, the rotamers are likely to coexist in frozen solution and may contribute independently to the observed photophysical properties.

5.5 Solvatochromism

Table S8. Selected photophysical properties (absorption and emission maxima, photoluminescence quantum yields, FWHM, fluorescence lifetimes, radiative and non-radiative rate constants) of conjugate **6b** recorded in different solvents.

solvent	$\lambda_{max,abs}$ / nm (ε / L · mol^{-1} · cm^{-1})[a]	$\lambda_{max,em}$ / nm (Φ_{PL})[b]	$\Delta\tilde{\nu}$ / cm^{-1} [c]	FWHM / cm^{-1} (nm)[d]	τ / ns (χ^2)[e]	k_r^{exp} / s^{-1} [f]	k_{nr}^{exp} / s^{-1} [g]
cyclohexane	236sh (23800), 294 (57400), 357 (4900)	423 (0.20)	4400	3240 (58)	4.6 (1.17)	$4.3\cdot10^7$	$1.7\cdot10^8$
toluene	295 (39200), 353 (3900)	448 (0.29)	6000	3340 (67)	7.2 (1.04)	$4.0\cdot10^7$	$9.9\cdot10^7$
ethyl acetate	291 (46400), 349sh (4100)	476 (0.16)	7600	3740 (84)	7.9 (1.19)	$2.0\cdot10^7$	$1.1\cdot10^8$
THF	281sh (40300), 292 (62700), 345sh (6200)	478 (0.20)	8100	3630 (83)	9.2 (1.11)	$2.2\cdot10^7$	$8.7\cdot10^7$
dichloromethane	293 (58900), 349sh (4900)	490 (0.23)	8200	3750 (90)	12.7 (1.10)	$1.8\cdot10^7$	$6.1\cdot10^7$
acetonitrile	233sh (28500), 289 (65300), 346sh (5900)	525 (0.12)	9900	4170 (115)	11.0 (1.10)	$1.1\cdot10^7$	$8.0\cdot10^7$

[a] Recorded in different solvents, T = 293 K, c_{abs} = 10^{-5} M. [b] Recorded in different solvents, T = 293 K, c_{em} = 10^{-7} M, absolute quantum yields determined using an integrating sphere, λ_{exc} = $\lambda_{max,abs}$. [c] $\Delta\tilde{\nu} = \left(\frac{1}{\lambda_{max,abs}} - \frac{1}{\lambda_{max,em}}\right) \cdot 10^7$ (λ_{max} in nm) [cm^{-1}]. [d] FWHM [cm^{-1}] determined using 10^7 · (FWHM [nm]/(λ_{em} [nm])^2). [e] Residuals χ^2. [f] $k_r^{exp} = \frac{\Phi_{PL}}{\tau}$. [g] $k_{nr}^{exp} = \frac{1-\Phi_{PL}}{\tau}$.

Table S9. Overview of the utilized values of compound **6b** depending on the solvent polarity characterized by the $E_T(30)$ values[10].

solvent	$\lambda_{max,em}$ [nm]	$\tilde{\nu}_{max,em}$ [cm^{-1}]	$E_T(30)$[10] [kcal/mol]
**cyclohexane**	423	23697	30.9
**toluene**	448	22321	33.9
**ethyl acetate**	476	21008	38.1
**THF**	478	20921	37.4
**dichloromethane**	490	20408	40.7
**acetonitrile**	525	19048	45.6

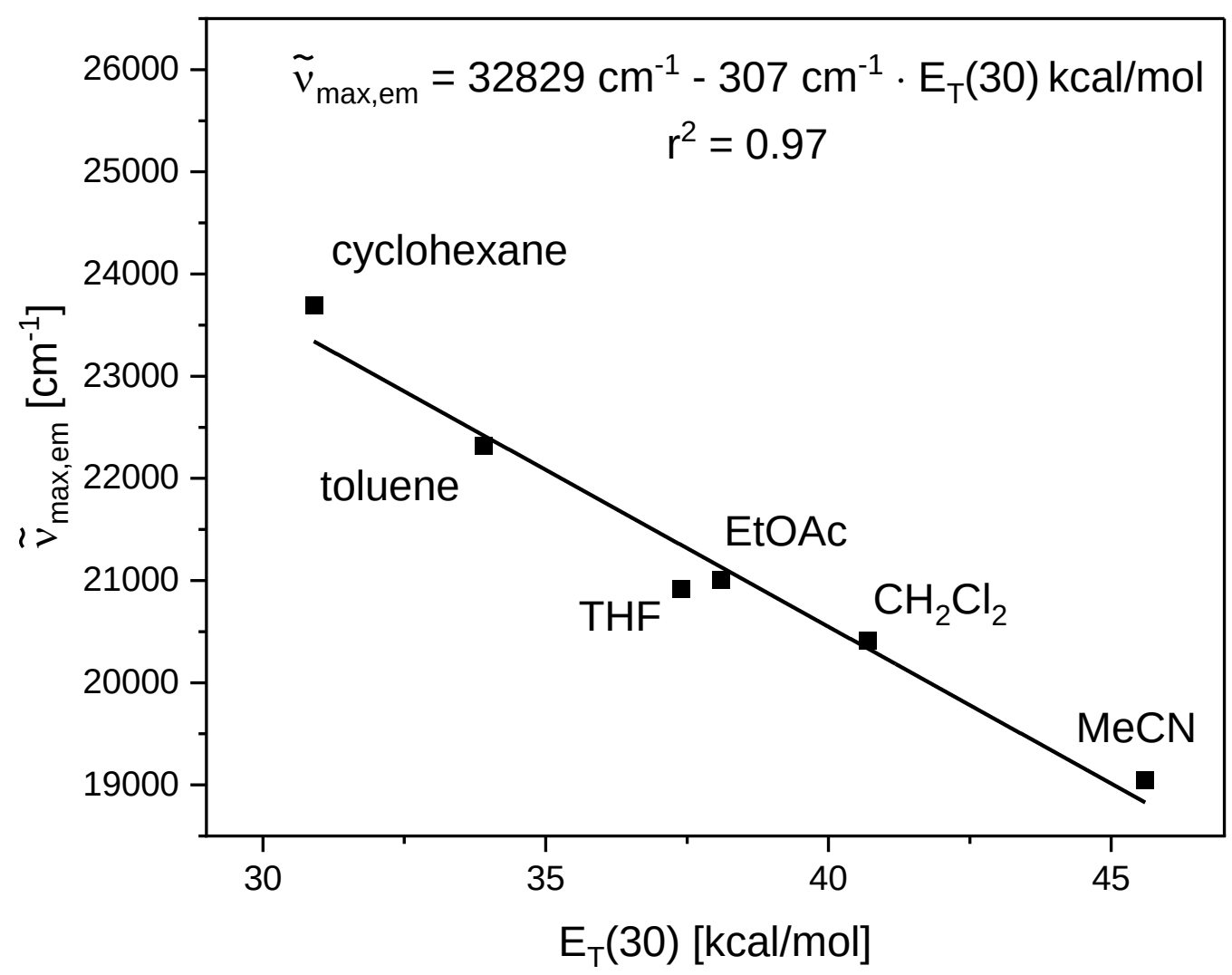

Figure S66. Correlation of the emission maxima compared to the E_T(30) values of compound **6b** in solvents of different polarity.

Table S10. Overview of the utilized values for the Lippert-Mataga plot of compound **6b**.

solvent	$\lambda_{max,em}$ [nm]	$\Delta\tilde{v}$ [cm^{-1}]	refractive index[11] n_D^{20}	permittivity[11] ε_r	orientation polarizability Δf[a]	Onsager radius a[b] [Å]	$\Delta f/a^3$ [10^{56} C^{-2} cm^{-3}]
**cyclohexane**	423	4400	1.4262	2.02	-0.001649728	5.71	-0.008019209
**toluene**	448	6000	1.4969	2.38	0.01323509	6.02	0.054899055
**ethyl acetate**	476	7600	1.3724	6.02	0.199635043	5.68	0.985868986
**THF**	478	8100	1.4072	7.58	0.209571742	6.11	0.831450745
**dichloromethane**	490	8200	1.4242	8.93	0.217103254	6.19	0.828365147
**acetonitrile**	525	9900	1.3441	35.94	0.30457145	6.11	1.208350686

[a] $\Delta f = \frac{\varepsilon_r - 1}{2\,\varepsilon_r + 1} - \frac{n^2 - 1}{2\,n^2 + 1}$. [b] TD-DFT calculations of the Onsager radius a (Gaussian 16, cam-B3LYP/6-311+G*, dipole, a_0 = 6.05 Å[c], volume) with different solvents. [c] TD-DFT calculated Onsager radius a_0 of the gas phase geometry (Gaussian 16, cam-B3LYP/6-311+G*).

The ground state geometries of compound **6b** in different solvents optimized by DFT calculations with the cam-B3LYP functional and the 6-311+G* basis set in the program package Gaussian 16[3]. The minima structures were confirmed by analytical frequency analysis (Nimag = 0). The keyword volume requests the computation of the molecule volume in angstroms (≈ Onsager-Radius a) and the keyword SCRF requests the computation in the presence of a solvent (vide infra).

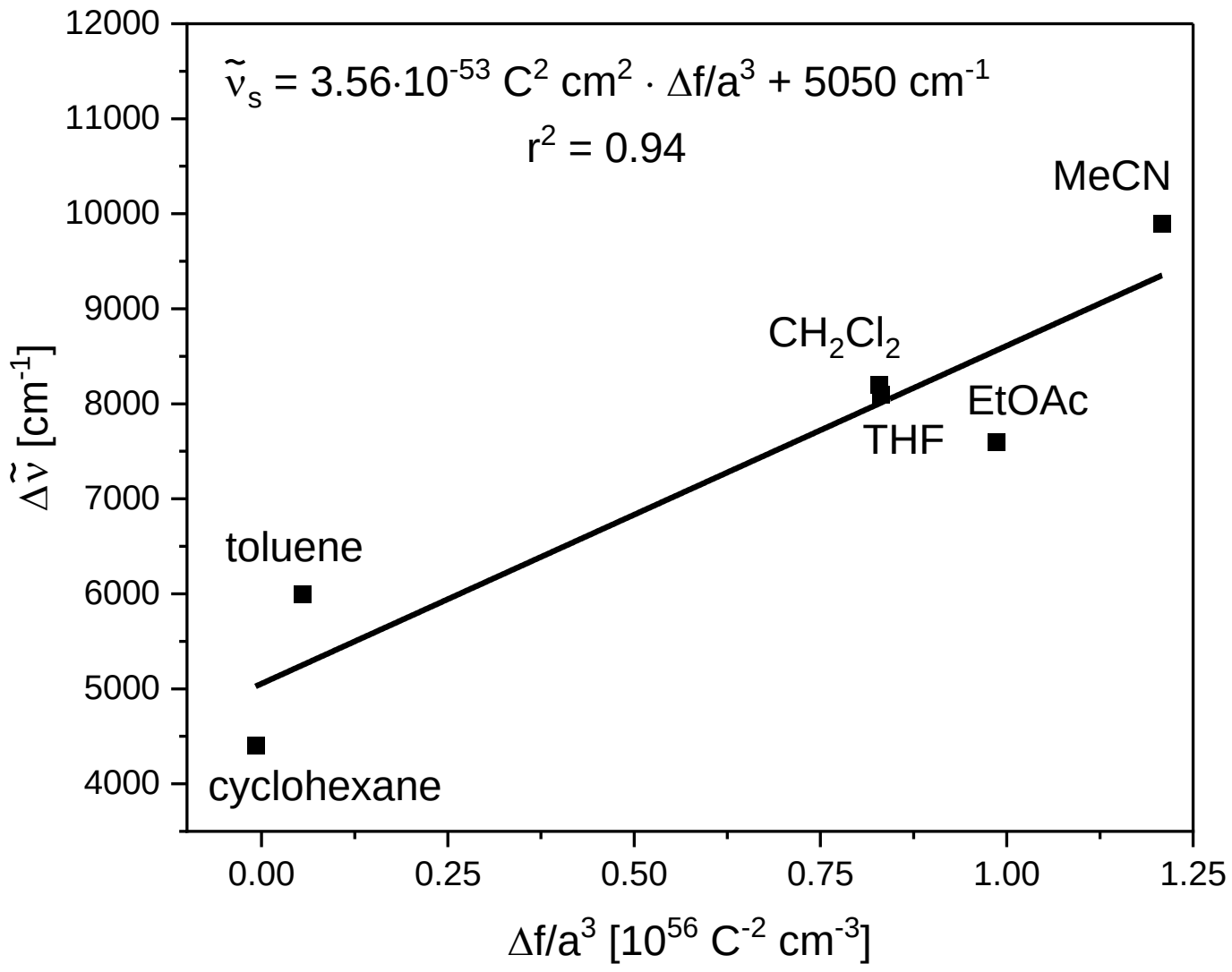

Figure S67. Lippert-Mataga plot of compound **6b** in solvents of different polarity.

The change in dipole moment can be calculated using the Lippert-Mataga equation 7.[12]

$$\tilde{\nu}_{abs} - \tilde{\nu}_{abs} = \Delta\tilde{\nu}_s = \frac{2\,\Delta f}{4\,\pi\,\varepsilon_0\,h\,c\,a^3}\,(\mu_{AZ} - \mu_{GZ})^2 + const. \tag{7}$$

Considering the Onsager radius *a* of the solvents assuming a spherical dipole into the correlation, the plot of the Stokes shift $\Delta\tilde{\nu}_S$ against the orientation polarizability Δ*f*, the dipole moment change $\Delta\mu$ can be determined using the slope m (equation 8).[12]

$$\Delta\mu = \mu_{AZ} - \mu_{GZ} = \sqrt{m} = \sqrt{3.56 \cdot 10^{-57}(Cm)^2} = 5.96657 \cdot 10^{-29}\,Cm = 17.9\,D \tag{8}$$

The molecule radius *a* can additionally be estimated from bond lengths. In this approach, the terphenyl unit is used as a representative structural motif due to the twisted conformation of the molecule. By this method a radius of 5.67 Å can be obtained (Figure S68).

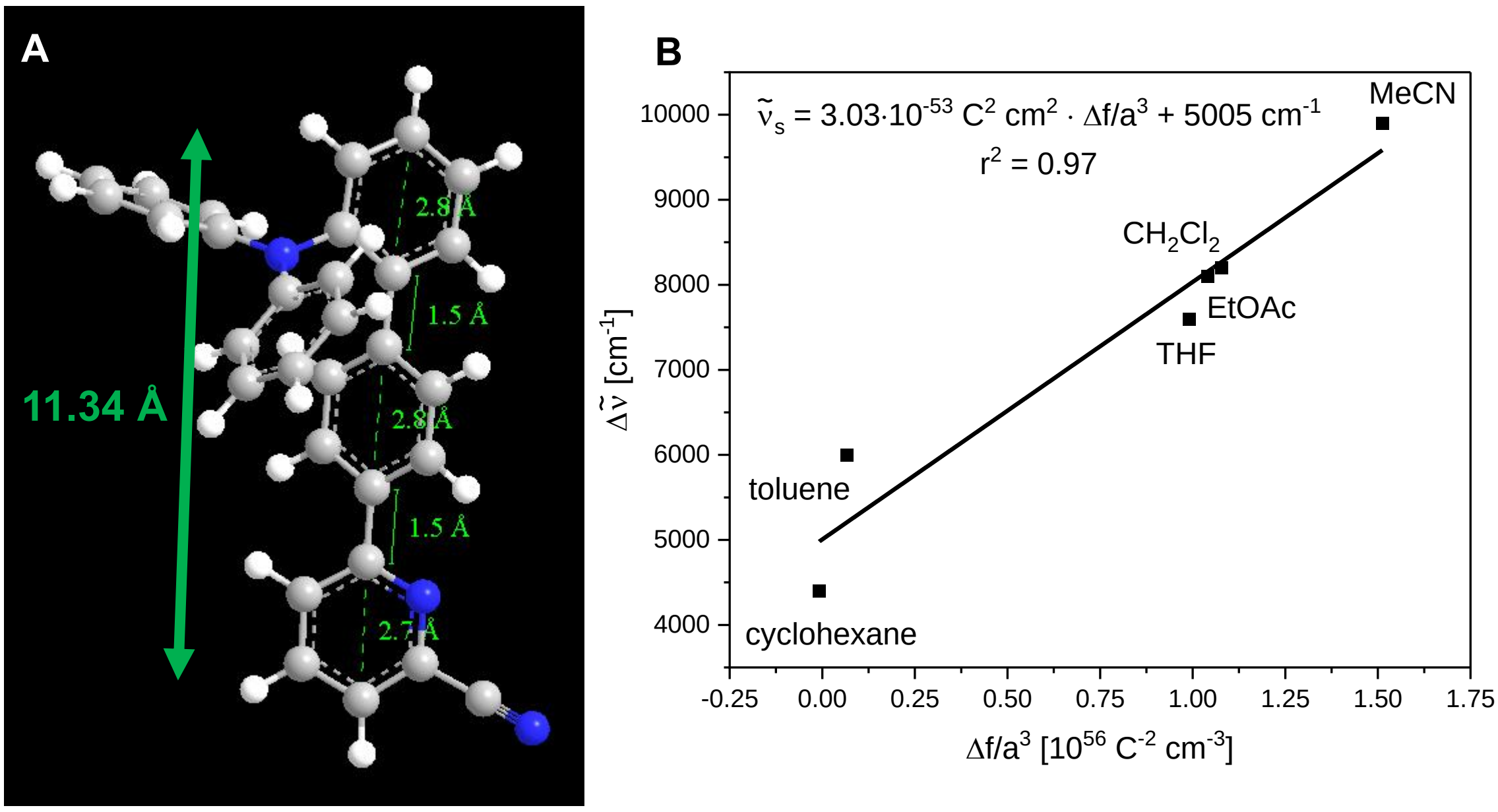

Figure S68. Estimation of molecule radius a by bond length obtained by the program Chem3D (**A**). Lippert-Mataga plot of compound **6b** in solvents of different polarity utilizing the molecule radius determined by bond length (**B**).

Considering the molecule radius *a* determined by bond length, the plot of the Stokes shift $\Delta\tilde{\nu}_S$ against the orientation polarizability Δf, the dipole moment change $\Delta\mu$ can be determined using the slope m (equation 8).[10]

$$\Delta\mu = \sqrt{3.03 \cdot 10^{-57} (Cm)^2} = 5.504544 \cdot 10^{-29}\, Cm = 16.5\, D$$

5.6 Emission spectra upon induced aggregation

In addition to the qualitative AIE-Study of compound **6a** (Figure S69) and **7b** (Figure S70), shake-flask experiments were performed for the remaining compounds investigated in this work to qualitatively assess their aggregation behavior in aqueous mixtures. Upon increasing the water content, visible turbidity was observed for these compounds, indicating a pronounced decrease in solubility and the formation of dispersed species or aggregates. These observations further support the general tendency of the investigated molecular structures to undergo aggregation upon increasing the water fraction. However, as no DLS or SEM measurements were performed, the size and morphology of the resulting species cannot be conclusively determined.

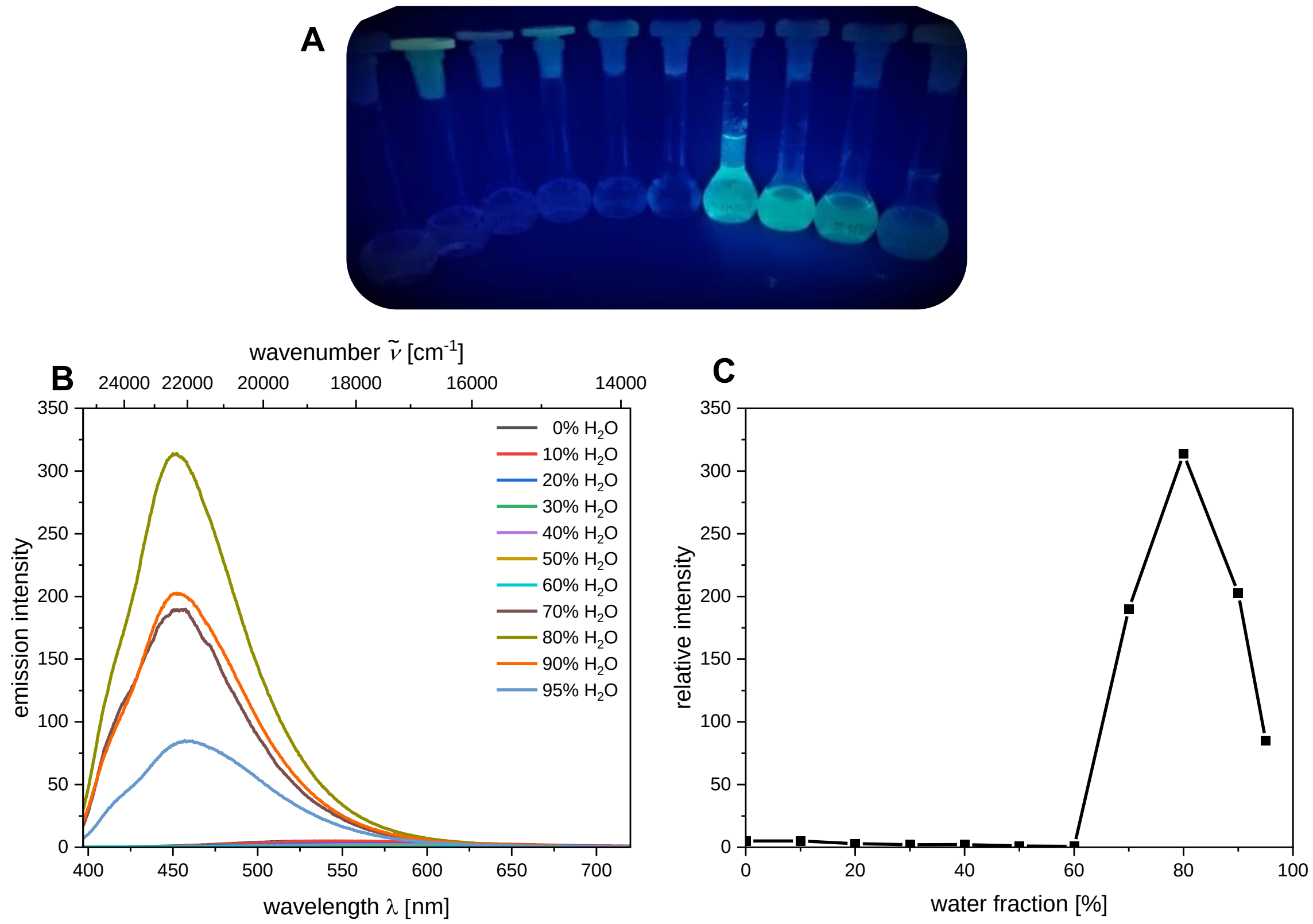

Figure S69. Qualitative AIE study of compound **6a** recorded in different ethanol/water mixtures (water fraction from left to right: 10%, 20%, 30%, 40%, 50%, 60%, 70%, 80%, 90%, 95%, λ_{exc} = 365 nm, T = 293 K, c = 10^{-4} M) (**A**), emission spectra for compound **6a** in different ethanol/water mixtures (T = 293 K, c_{em} = 10^{-6} M , λ_{exc} = $\lambda_{max,em}$) (**B**) and the change in emission intensity as a function of the water fraction (**C**).

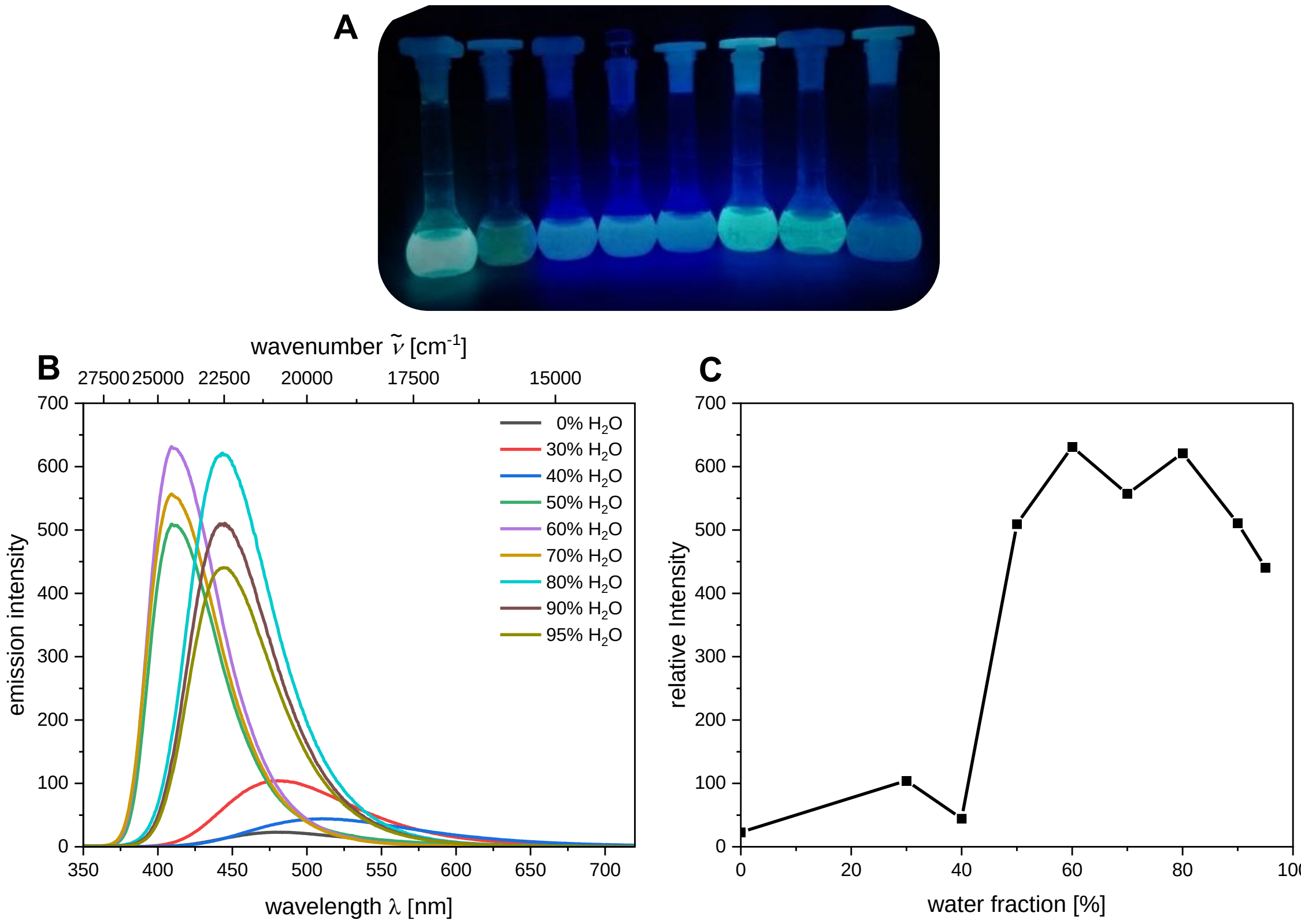

Figure S70. Qualitative AIE study of compound **7b** recorded in different ethanol/water mixtures (water fraction from left to right: 30%, 40%, 50%, 60%, 70%, 80%, 90%, 95%, λ_{exc} = 365 nm, T = 293 K, c = 10^{-4} M) (**A**), emission spectra for compound **7b** in different ethanol/water mixtures (T = 293 K, c_{em} = 10^{-6} M , λ_{exc} = $\lambda_{max,em}$) (**B**) and the change in emission intensity as a function of the water fraction (**C**).

6 Photoluminescence lifetime measurements

6.1 Decay spectra in the solid state

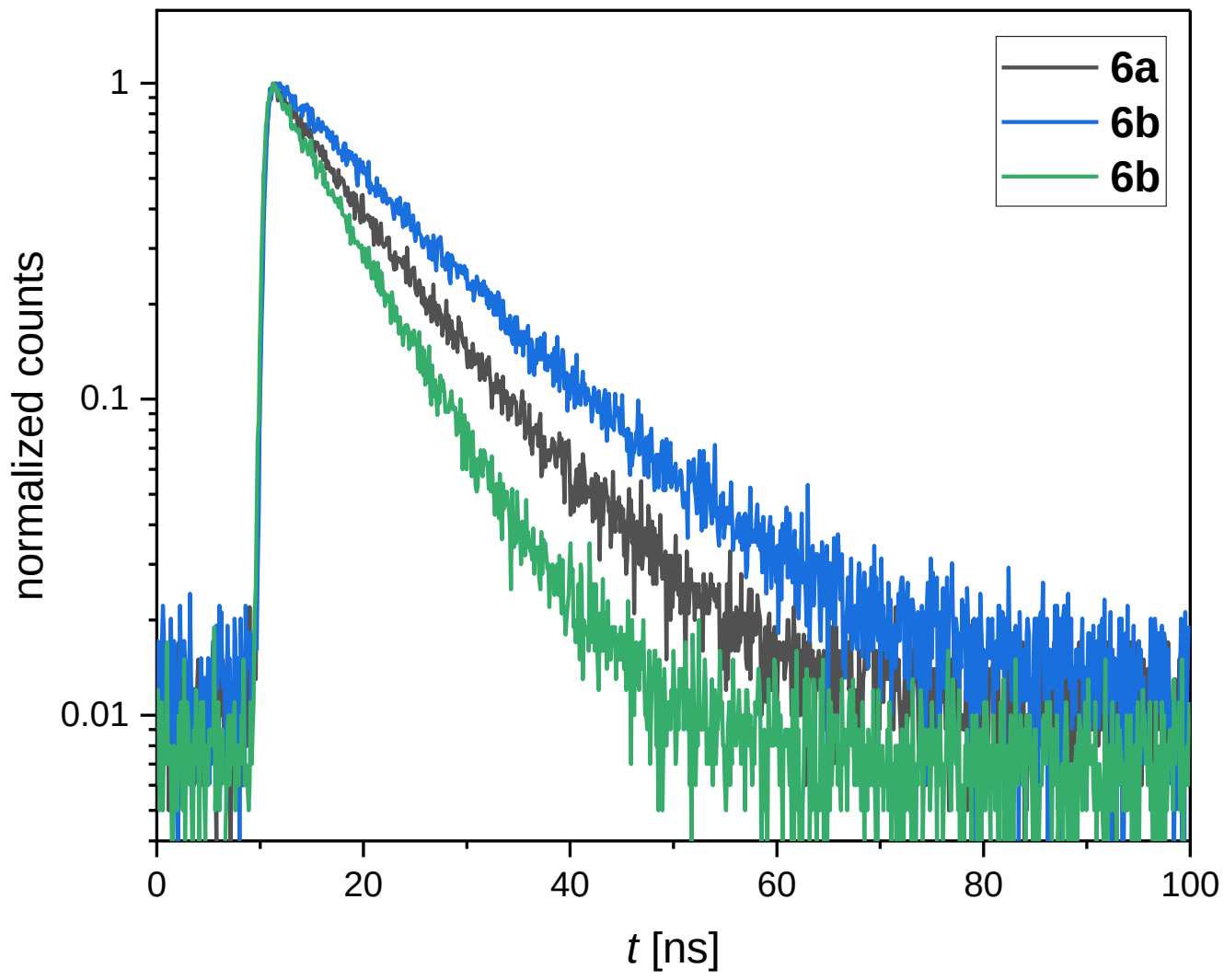

Figure S71. Normalized emission decay spectra in the TCSPC mode of compound **6a** (black line), **6b** (blue line) and **6c** (green line) recorded in the solid state, T = 298 K, λ_{exc} = 313.5 nm, λ_{det}(**6a**) = 440 nm, λ_{det}(**6b**) = 457 nm, λ_{det}(**6c**) = 434 nm.

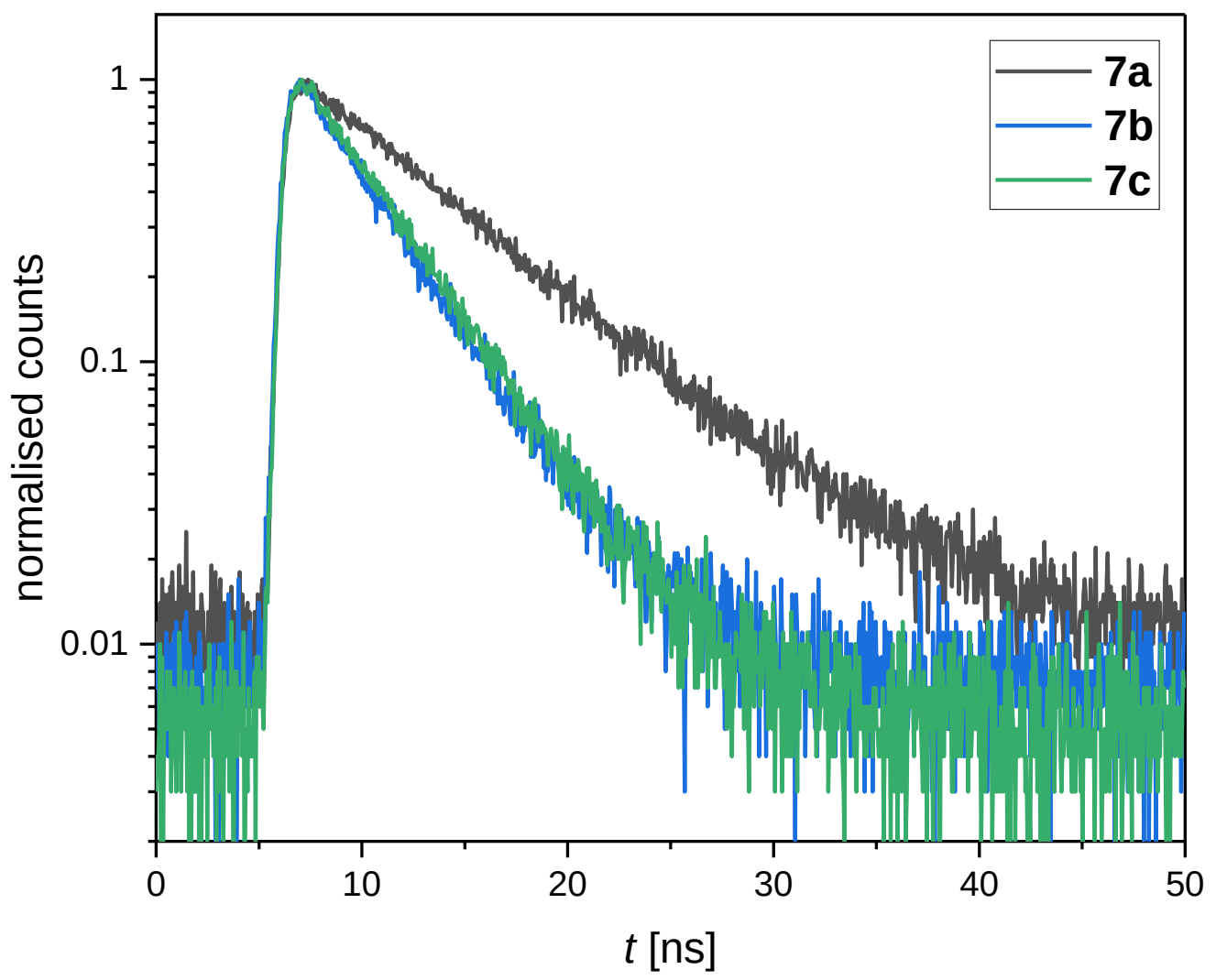

Figure S72. Normalized emission decay spectra in the TCSPC mode of compound **7a** (black line), **7b** (blue line) and **7c** (green line) recorded in the solid state, T = 298 K, λ_{exc} = 313.5 nm, λ_{det}(**7a**) = 435 nm, λ_{det}(**7b**) = 413 nm, λ_{det}(**7c**) = 408 nm.

6.2 Decay spectra in solution

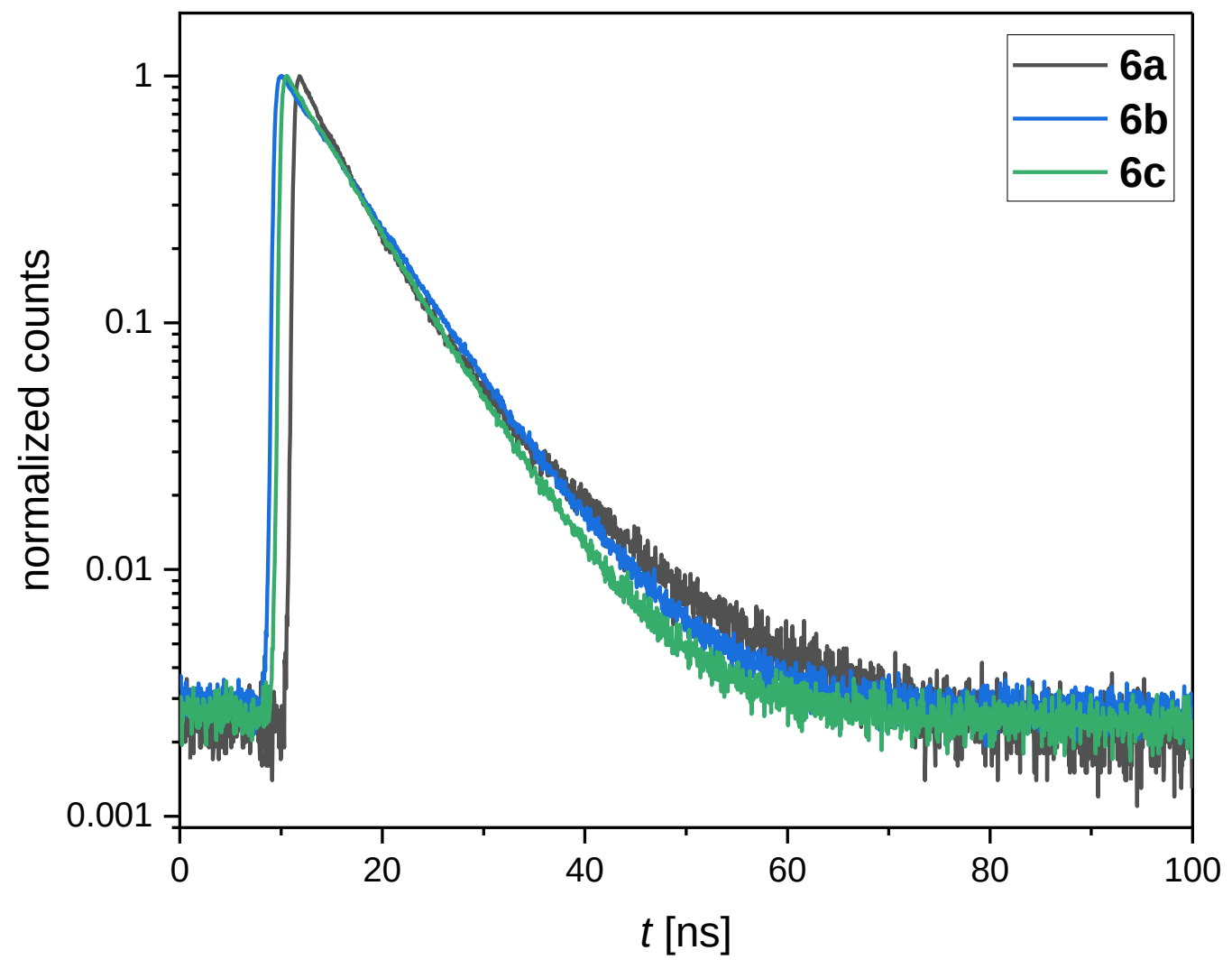

Figure S73. Normalized emission decay spectra in the TCSPC mode of compound **6a** (black line), **6b** (blue line) and **6c** (green line) recorded in toluene, T = 298 K, c_{em} = 10^{-6} M, λ_{exc} = 372.2 nm, λ_{det}(**6a**) = 457 nm, λ_{det}(**6b**) = 448 nm, λ_{det}(**6c**) = 442 nm.

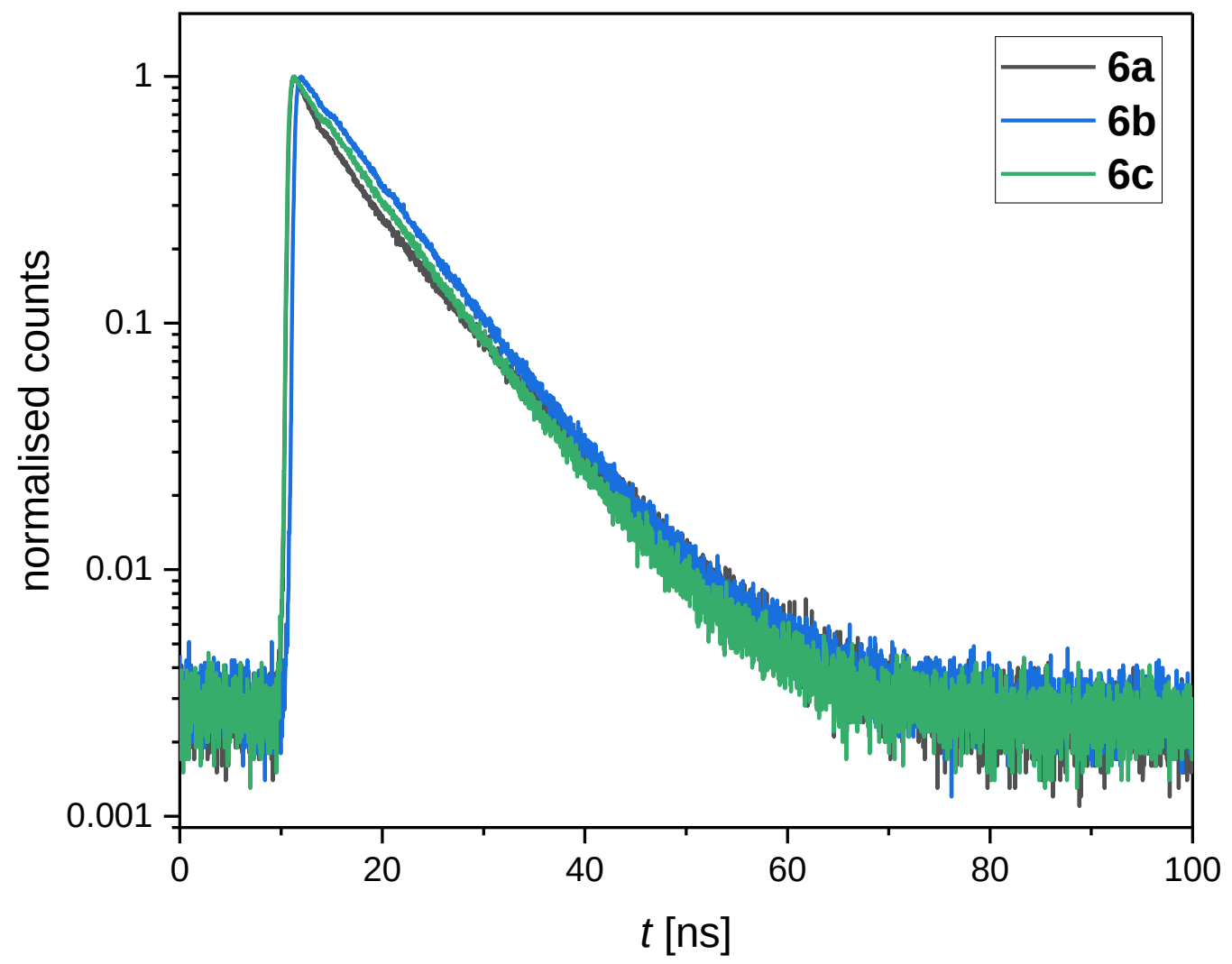

Figure S74. Normalized emission decay spectra in the TCSPC mode of compound **6a** (black line), **6b** (blue line) and **6c** (green line) recorded in ethyl acetate, T = 298 K, c_{em} = 10^{-6} M, λ_{exc} = 372.2 nm, λ_{det}(**6a**) = 485 nm, λ_{det}(**6b**) = 474 nm, λ_{det}(**6c**) = 463 nm.

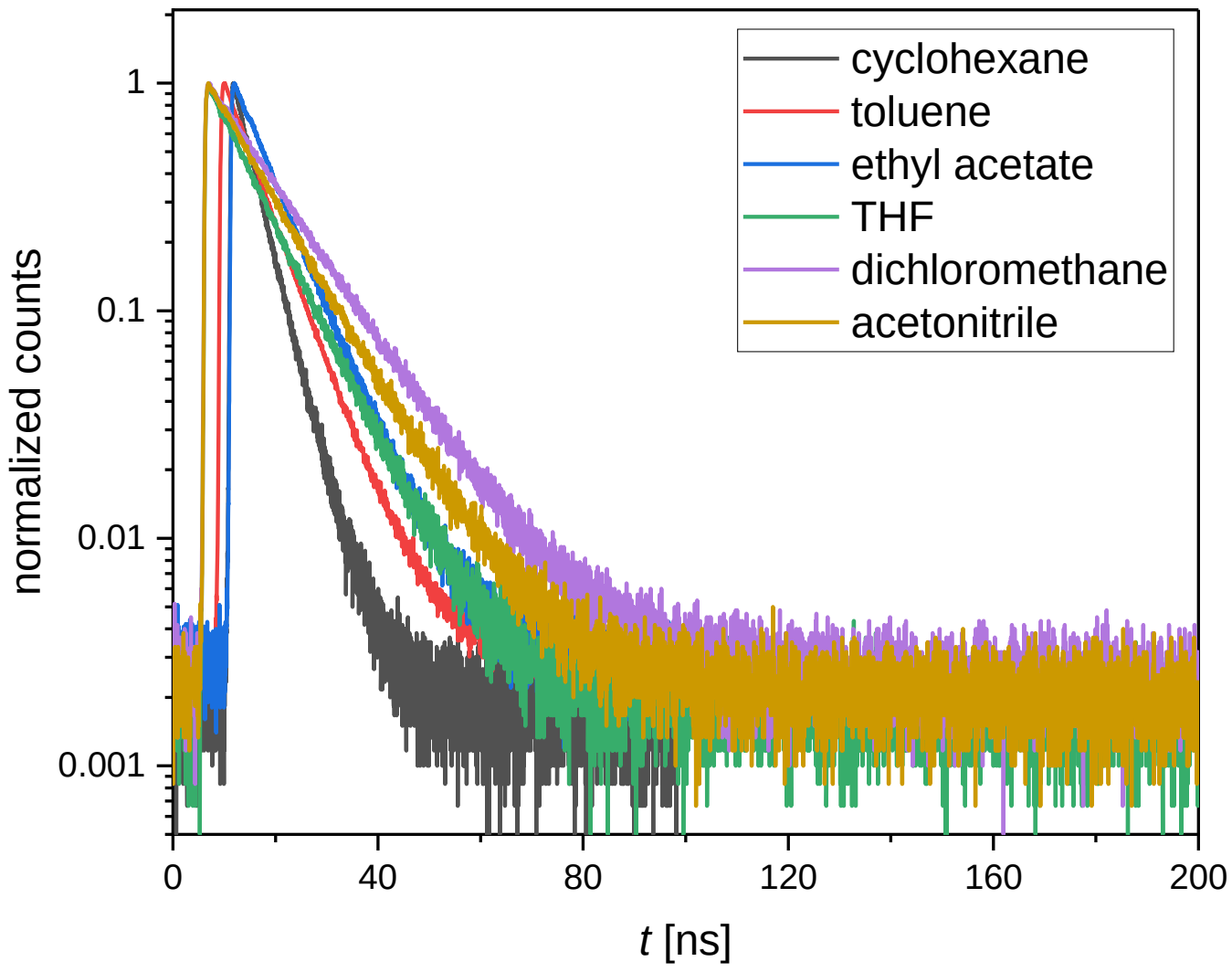

Figure S75. Normalized emission decay spectra in the TCSPC mode of compound **6b** recorded in cyclohexane (black line), toluene (red line), ethyl acetate (blue line), THF (green line), dichloromethane (purple line) and acetonitrile (yellow line), $T = 298$ K, $c_{em} = 10^{-6}$ M, $\lambda_{exc} = 372.2$ nm, λ_{det}(Cy) = 423 nm, λ_{det}(Tol) = 448 nm, λ_{det}(EE) = 476 nm, λ_{det}(THF) = 478 nm, λ_{det}(CH_2Cl_2) = 490 nm, λ_{det}(MeCN) = 525 nm.

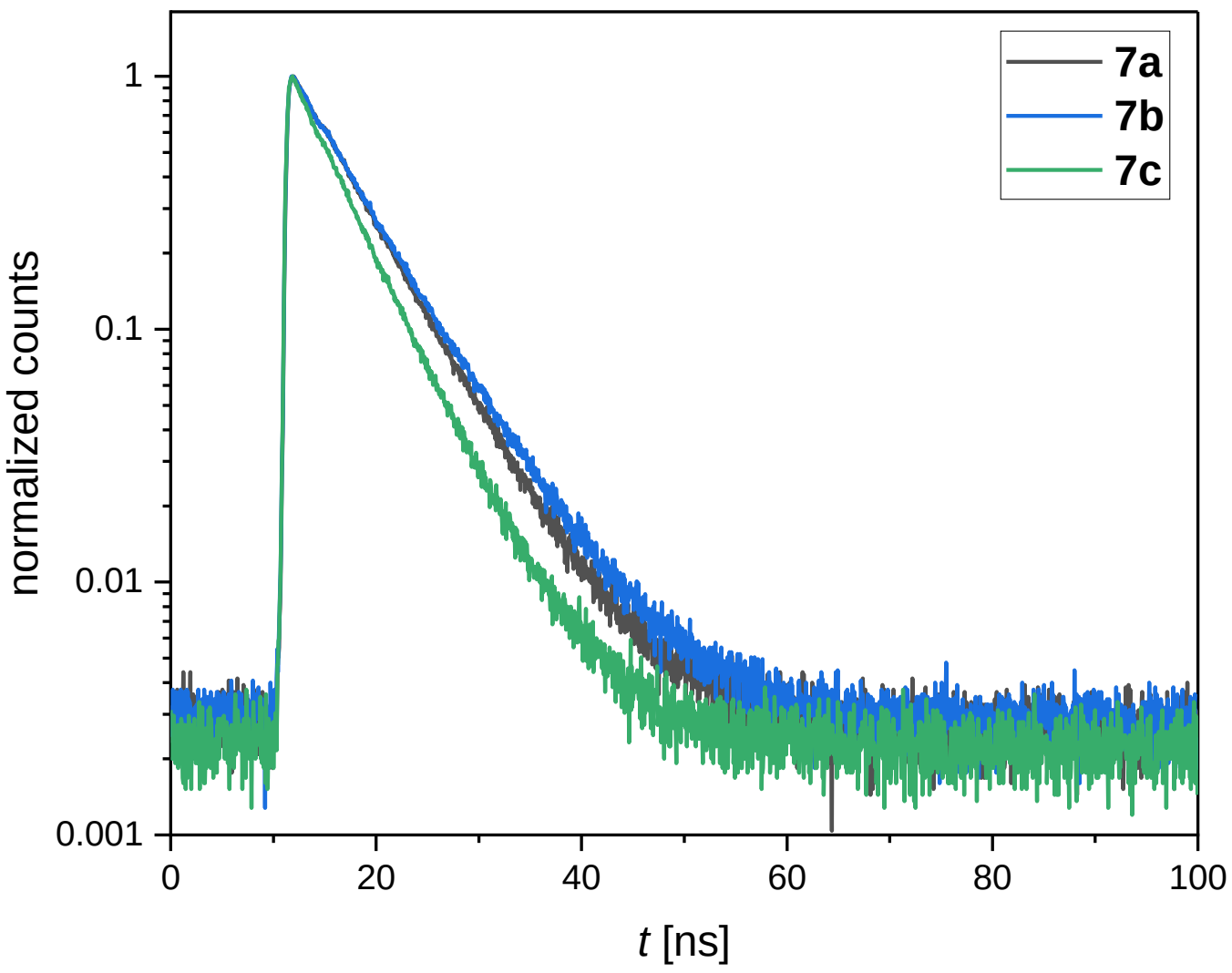

Figure S76. Normalized emission decay spectra in the TCSPC mode of compound **7a** (black line), **7b** (blue line) and **7c** (green line) recorded in toluene, $T = 298$ K, $c_{em} = 10^{-6}$ M, $\lambda_{exc} = 372.2$ nm, λ_{det}(**7a**) = 434 nm, λ_{det}(**7b**) = 441 nm, λ_{det}(**7c**) = 424 nm.

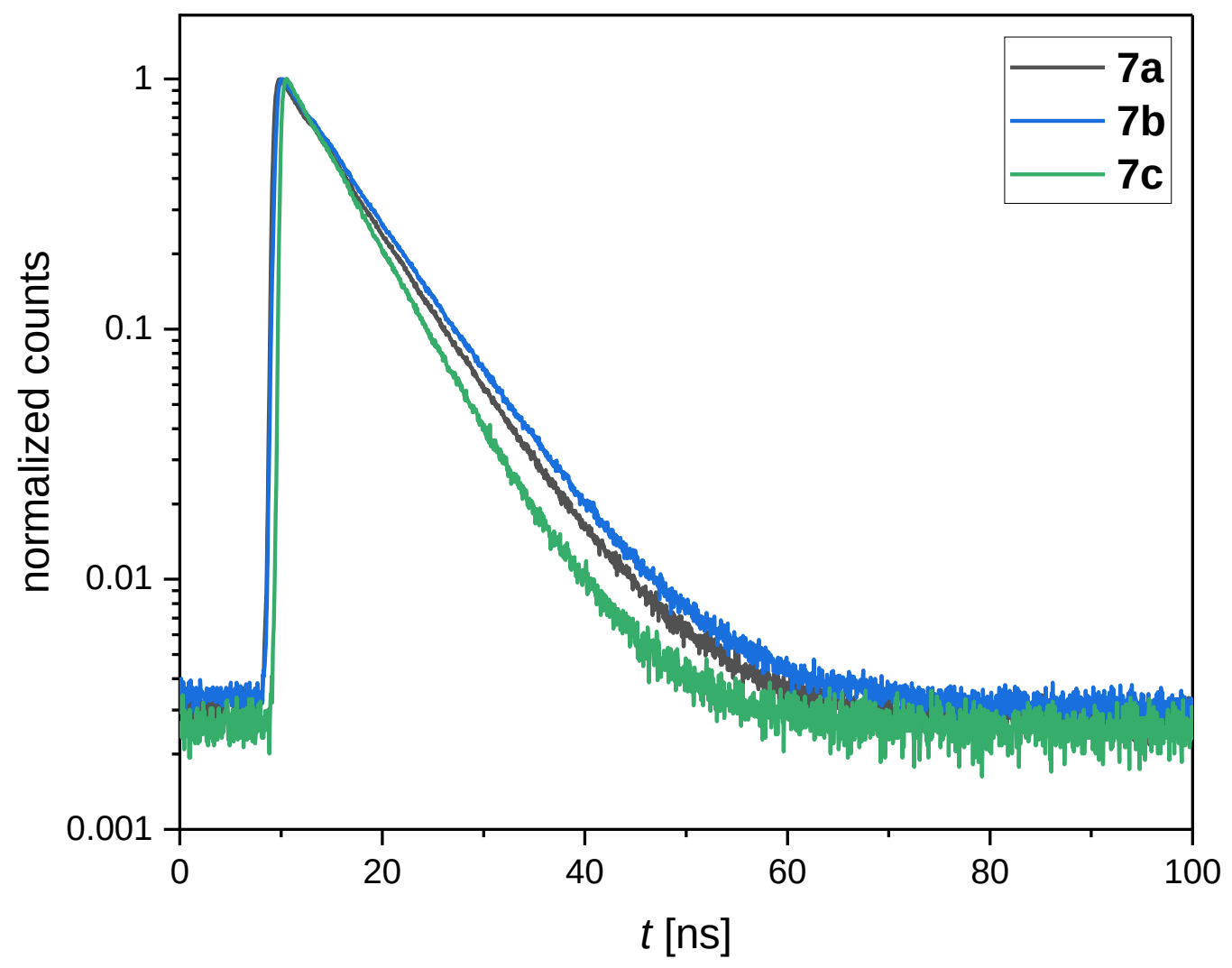

Figure S77. Normalized emission decay spectra in the TCSPC mode of compound **7a** (black line), **7b** (blue line) and **7c** (green line) recorded in ethyl acetate, $T = 298$ K, $c_{em} = 10^{-6}$ M, $\lambda_{exc} = 372.2$ nm, λ_{det}(**7a**) = 445 nm, λ_{det}(**7b**) = 458 nm, λ_{det}(**7c**) = 435 nm.

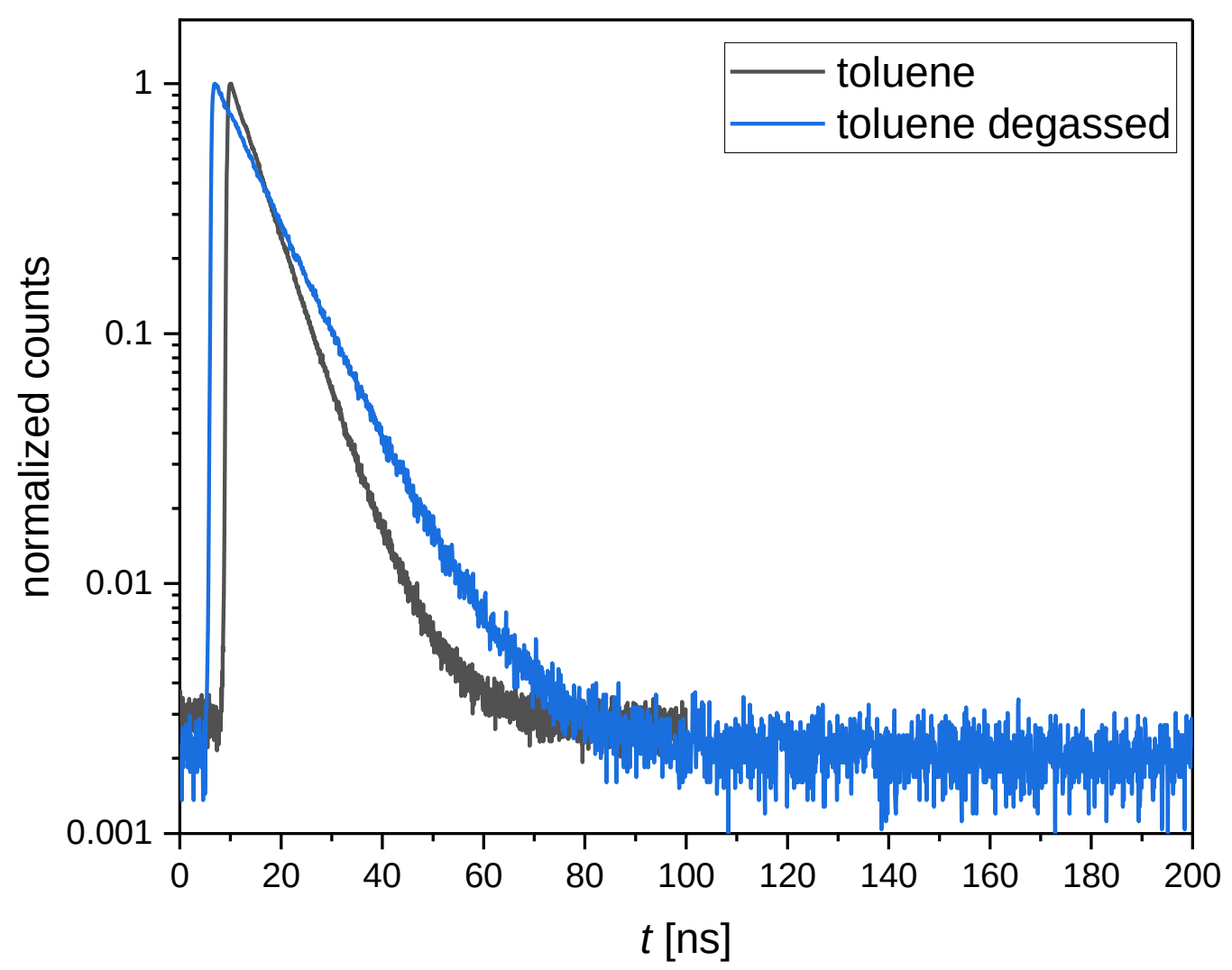

Figure S78. Normalized emission decay spectra in the TCSPC mode of compound **6b** recorded in toluene (black line) and degassed toluene with argon (blue line), $T = 298$ K, $c_{em} = 10^{-6}$ M, $\lambda_{exc} = 372.2$ nm, λ_{det}(Tol) = 448 nm.

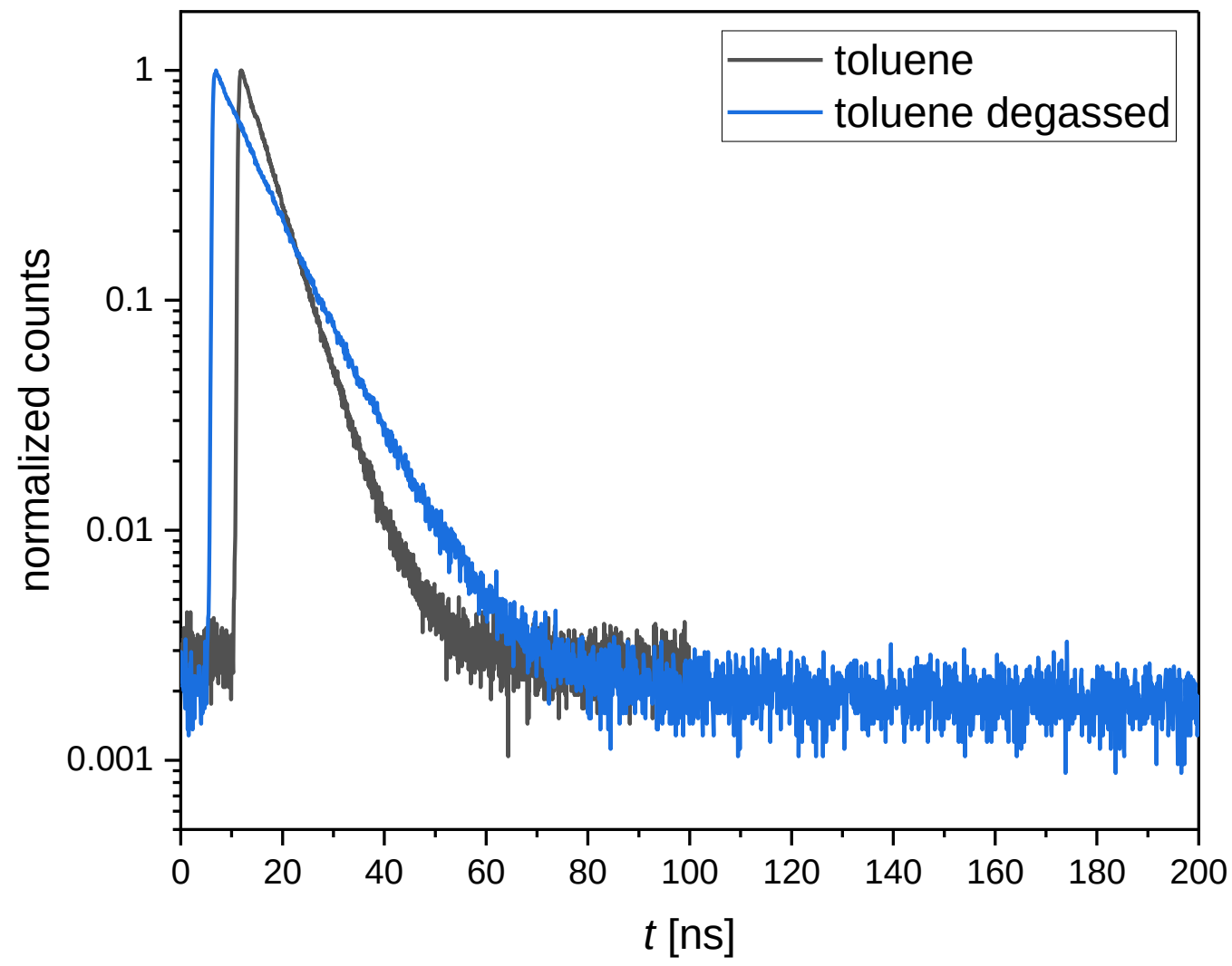

Figure S79. Normalized emission decay spectra in the TCSPC mode of compound **7b** recorded in toluene (black line) and degassed toluene with argon (blue line), T = 298 K, c_{em} = 10^{-6} M, λ_{exc} = 372.2 nm, λ_{det}(Tol) = 441 nm.

6.3 Decay spectra in a PMMA matrix

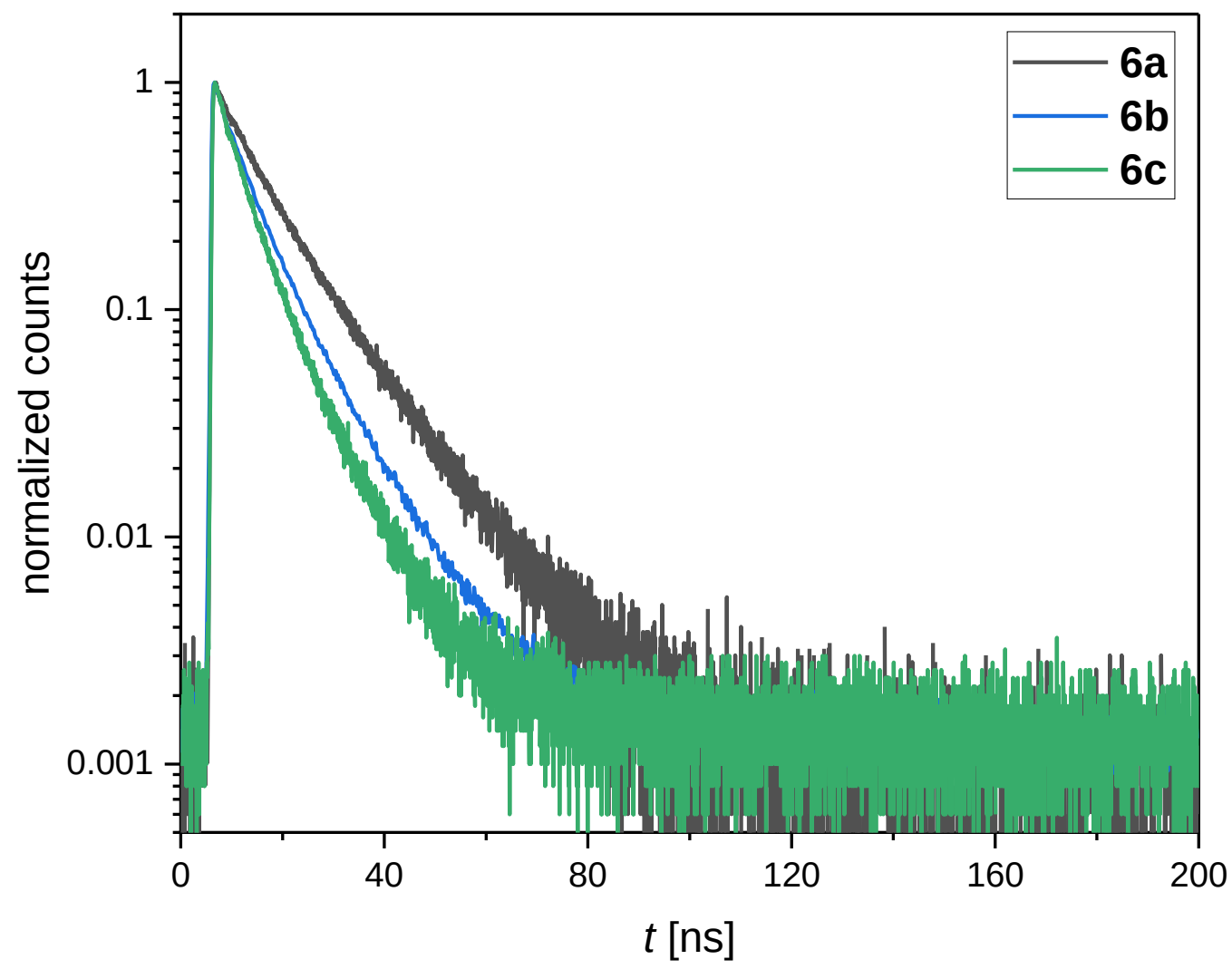

Figure S80. Normalized emission decay spectra in the TCSPC mode of compound **6a** (black line), **6b** (blue line) and **6c** (green line) recorded in a PMMA matrix, T = 298 K, c(**6**) = 1 wt%, λ_{exc} = 372.2 nm, λ_{det}(**6a**) = 444 nm, λ_{det}(**6b**) = 438 nm, λ_{det}(**6c**) = 424 nm.

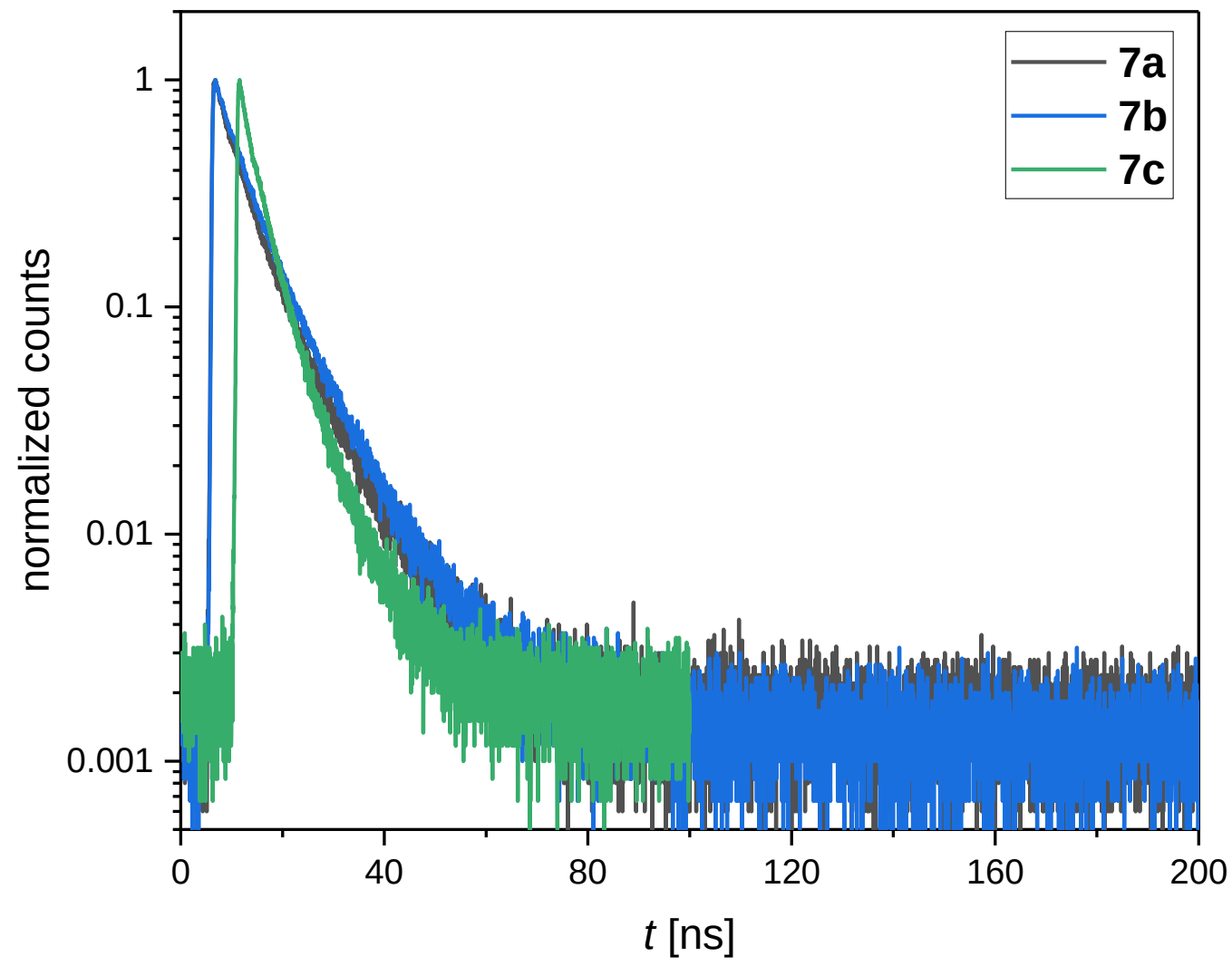

Figure S81. Normalized emission decay spectra in the TCSPC mode of compound **7a** (black line), **7b** (blue line) and **7c** (green line) recorded in a PMMA matrix, T = 298 K, c(**7**) = 1 wt%, λ_{exc} = 372.2 nm, λ_{det}(**7a**) = 426 nm, λ_{det}(**7b**) = 428 nm, λ_{det}(**7c**) = 408 nm.

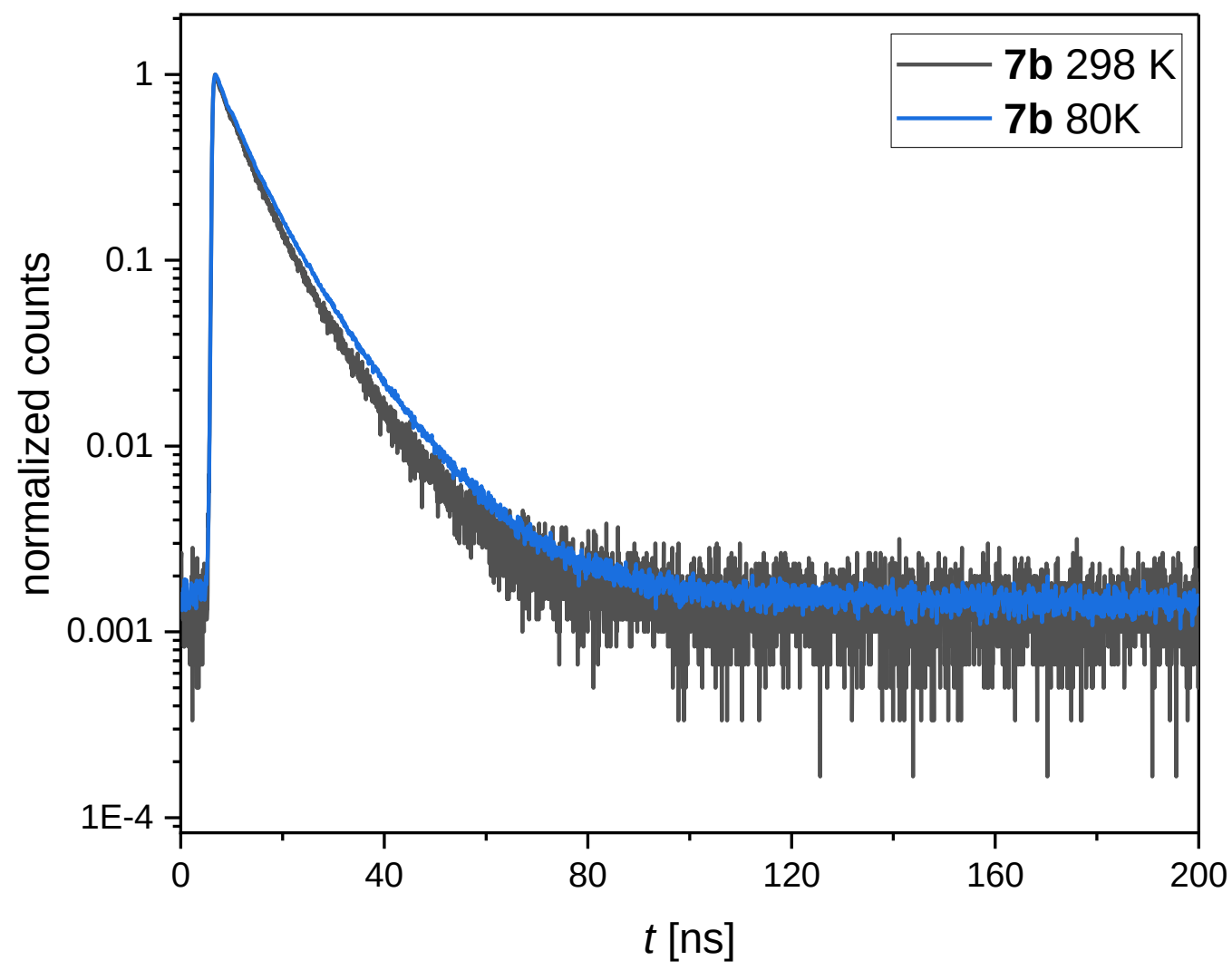

Figure S82. Normalized emission decay spectra in the TCSPC mode of compound **7b** at 298 K (black line) and 80 K (blue line) recorded in a PMMA matrix, T = 298–80 K, c(**7b**) = 1 wt%, λ_{exc} = 372.2 nm, λ_{det}(**7b**) = 428 nm.

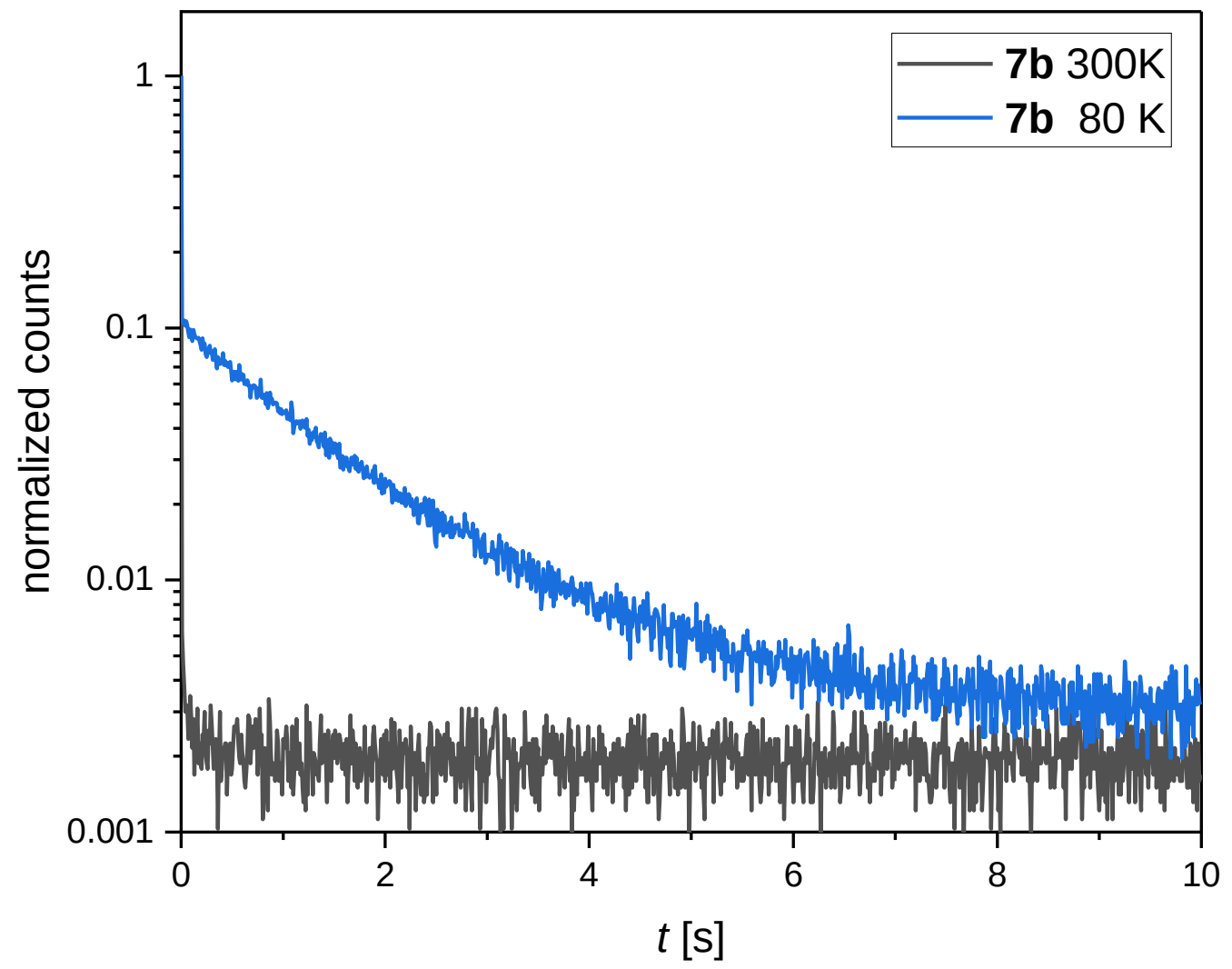

Figure S83. Normalized emission decay spectra in MCS mode of compound **7b** at 300 K (black line) and 80 K (blue line) recorded in a PMMA matrix, T = 300–80 K, c(**7b**) = 1 wt%, λ_{exc} = 345 nm, λ_{det}(**7b**) = 428 nm.

7 Quantum chemical calculations

7.1 Quantum chemical calculation data of 6-(2-(diphenylamino)phenyl)picolinonitrile (6a)

7.1.1 Computed xyz-coordinates (S_0 geometry) of compound 6a

(CAM-B3LYP/6-311+G* PCM Toluene)

N	-0.923198	0.256334	0.514453
C	-2.105660	-0.320964	-0.012246
C	-0.171462	-0.399148	1.511389
C	-0.572601	1.573936	0.118301
C	0.698468	1.866473	-0.396153
C	1.021011	3.189331	-0.693673
C	0.094139	4.208359	-0.536046
C	-1.176264	3.908310	-0.063236
C	-1.499537	2.603002	0.270675
C	-3.011458	-0.983758	0.816425
C	-4.170731	-1.531123	0.287050
C	-4.452438	-1.411541	-1.068246
C	-3.554537	-0.745633	-1.892757
C	-2.384325	-0.211547	-1.373909
C	-0.014832	-1.787752	1.485254
C	0.723581	-2.428650	2.466922
C	1.335464	-1.702991	3.482989
C	1.191233	-0.322752	3.505881
C	0.440583	0.326476	2.535514
C	1.701074	0.803507	-0.670734
C	2.994762	0.885580	-0.150118
C	3.913056	-0.101623	-0.456570
C	3.522877	-1.150552	-1.274267
C	2.215630	-1.144272	-1.743407
N	1.321164	-0.199651	-1.458647
C	1.764913	-2.212658	-2.603386
N	1.435684	-3.074324	-3.286590
H	2.003804	3.414671	-1.093659
H	0.359204	5.227506	-0.792326
H	-1.912921	4.693727	0.062714
H	-2.480164	2.366053	0.666645
H	-2.804405	-1.069473	1.876555
H	-4.865054	-2.044198	0.943571
H	-5.361737	-1.836124	-1.478109
H	-3.755710	-0.654877	-2.954518
H	-1.671124	0.282426	-2.022245
H	-0.474458	-2.361605	0.689975
H	0.831845	-3.507190	2.428660
H	1.915725	-2.208344	4.246415
H	1.655012	0.259579	4.294856
H	0.321741	1.402784	2.575035
H	3.263182	1.709832	0.498753
H	4.920114	-0.061465	-0.058913
H	4.201706	-1.949866	-1.541827

SCF Done: E(RCAM-B3LYP) = -1088.66884035 A.U. after 1 cycles
Zero-point correction= 0.348561 (Hartree/Particle)
Thermal correction to Energy= 0.369653
Thermal correction to Enthalpy= 0.370597
Thermal correction to Gibbs Free Energy= 0.295780
Sum of electronic and zero-point Energies= -1088.320279

Sum of electronic and thermal Energies= -1088.299187
Sum of electronic and thermal Enthalpies= -1088.298243
Sum of electronic and thermal Free Energies= -1088.373060

(BMK/6-311+G* PCM Toluene)

N	-0.933852	0.288402	0.593177
C	-2.102215	-0.360027	0.128841
C	-0.055853	-0.340013	1.500334
C	-0.642524	1.597178	0.130100
C	0.599960	1.901088	-0.466070
C	0.881878	3.226696	-0.824058
C	-0.065106	4.236248	-0.650196
C	-1.311252	3.922757	-0.100685
C	-1.591060	2.616160	0.296100
C	-2.899248	-1.116548	1.003855
C	-4.055745	-1.740359	0.536274
C	-4.444962	-1.607491	-0.800288
C	-3.655189	-0.850157	-1.669472
C	-2.486900	-0.237299	-1.215095
C	0.188722	-1.722602	1.423600
C	1.067042	-2.332075	2.318369
C	1.730940	-1.579838	3.293199
C	1.497846	-0.204341	3.364379
C	0.609464	0.413278	2.482617
C	1.606686	0.835375	-0.755700
C	2.927653	0.944854	-0.286963
C	3.840816	-0.060435	-0.587795
C	3.416761	-1.148981	-1.348657
C	2.083038	-1.157221	-1.767136
N	1.196438	-0.201688	-1.485495
C	1.601232	-2.265356	-2.570536
N	1.255453	-3.157095	-3.207970
H	1.840235	3.457247	-1.282784
H	0.163454	5.254128	-0.952262
H	-2.059475	4.698342	0.037733
H	-2.546293	2.370519	0.751484
H	-2.607914	-1.213396	2.045551
H	-4.662111	-2.322497	1.225306
H	-5.349180	-2.090530	-1.159276
H	-3.937565	-0.748512	-2.713966
H	-1.858096	0.326485	-1.897304
H	-0.310534	-2.311044	0.660026
H	1.242656	-3.402036	2.242667
H	2.417087	-2.059113	3.985406
H	1.998986	0.395445	4.119804
H	0.425537	1.481301	2.554080
H	3.216566	1.799010	0.317397
H	4.864886	-0.004568	-0.230745
H	4.083576	-1.963467	-1.611221

SCF Done: E(RBMK) = -1088.53153093 A.U. after 1 cycles
Zero-point correction= 0.346211 (Hartree/Particle)
Thermal correction to Energy= 0.367290
Thermal correction to Enthalpy= 0.368235
Thermal correction to Gibbs Free Energy= 0.294146
Sum of electronic and zero-point Energies= -1088.185319
Sum of electronic and thermal Energies= -1088.164240
Sum of electronic and thermal Enthalpies= -1088.163296
Sum of electronic and thermal Free Energies= -1088.237385

7.1.2 Computed excitations energies of compound 6a

(CAM-B3LYP/6-311+G* PCM Toluene, TD=(Nstates=25,root=1))

```
Excited State   1:      Singlet-A      3.7725 eV  328.66 nm  f=0.0290  <S**2>=0.000
      91 -> 92        -0.34775
      91 -> 93         0.58551
 This state for optimization and/or second-order correction.
 Total Energy, E(TD-HF/TD-DFT) =  -1088.53020460
 Copying the excited state density for this state as the 1-particle RhoCI density.

 Excited State   2:      Singlet-A      3.9345 eV  315.12 nm  f=0.0840  <S**2>=0.000
      91 -> 92         0.60075
      91 -> 93         0.33303

 Excited State   3:      Singlet-A      4.3277 eV  286.49 nm  f=0.1701  <S**2>=0.000
      91 -> 94         0.66314
      91 -> 97         0.11184

 Excited State   4:      Singlet-A      4.5248 eV  274.01 nm  f=0.2907  <S**2>=0.000
      91 -> 95        -0.34604
      91 -> 96         0.57796

 Excited State   5:      Singlet-A      4.7065 eV  263.43 nm  f=0.0542  <S**2>=0.000
      90 -> 96         0.10658
      91 -> 93        -0.12232
      91 -> 95         0.28682
      91 -> 96         0.19282
      91 -> 97         0.45032
      91 -> 98         0.24848
      91 ->100        -0.15245

 Excited State   6:      Singlet-A      4.9403 eV  250.96 nm  f=0.0370  <S**2>=0.000
      84 -> 92         0.17671
      87 -> 96        -0.10293
      88 -> 94        -0.11476
      91 -> 95         0.11501
      91 -> 97         0.12736
      91 -> 98        -0.23985
      91 ->100         0.25436
      91 ->101        -0.12623
      91 ->102        -0.39096

 Excited State   7:      Singlet-A      4.9522 eV  250.36 nm  f=0.0139  <S**2>=0.000
      82 -> 92        -0.12238
      83 -> 92         0.14005
      84 -> 92         0.57370
      84 -> 93        -0.15502
      85 -> 92         0.16626
      88 -> 92        -0.10216
      91 -> 98         0.11701

 Excited State   8:      Singlet-A      5.0399 eV  246.01 nm  f=0.0249  <S**2>=0.000
      86 -> 93         0.13212
      89 -> 94         0.14752
      91 -> 95         0.18099
```

```
     91 -> 96         0.12156
     91 -> 97         0.14396
     91 -> 98        -0.32654
     91 ->100         0.13092
     91 ->101         0.12146
     91 ->102         0.39469

 Excited State   9:      Singlet-A      5.0618 eV  244.94 nm  f=0.1933  <S**2>=0.000
     83 -> 92        -0.15063
     83 -> 93        -0.11083
     85 -> 92         0.18946
     85 -> 93        -0.15750
     88 -> 92         0.38613
     88 -> 93        -0.19803
     89 -> 92        -0.35444
     89 -> 93         0.18266

 Excited State  10:      Singlet-A      5.3304 eV  232.60 nm  f=0.0032  <S**2>=0.000
     91 -> 95         0.39170
     91 -> 96         0.19327
     91 -> 97        -0.30966
     91 -> 98         0.19000
     91 -> 99         0.26081
     91 ->100         0.15791
     91 ->101         0.12516

 Excited State  11:      Singlet-A      5.4349 eV  228.13 nm  f=0.3471  <S**2>=0.000
     85 -> 92        -0.11578
     88 -> 92        -0.23597
     88 -> 93        -0.34422
     89 -> 92         0.13172
     89 -> 93         0.47114
     91 -> 98        -0.10195

 Excited State  12:      Singlet-A      5.4567 eV  227.22 nm  f=0.0012  <S**2>=0.000
     82 -> 93        -0.10417
     83 -> 93         0.10165
     84 -> 92         0.15878
     84 -> 93         0.56427
     84 -> 97        -0.11582
     85 -> 93         0.17514

 Excited State  13:      Singlet-A      5.6282 eV  220.29 nm  f=0.0229  <S**2>=0.000
     84 -> 93        -0.11681
     85 -> 92        -0.10038
     86 -> 93         0.30775
     86 -> 98        -0.10288
     88 -> 93        -0.17094
     88 -> 94        -0.11943
     89 -> 94         0.27948
     90 -> 93         0.18851
     91 -> 98         0.24639

 Excited State  14:      Singlet-A      5.6779 eV  218.36 nm  f=0.0107  <S**2>=0.000
     87 -> 92         0.16773
     88 -> 92         0.22030
     89 -> 92         0.30454
```

90 -> 92 0.29641
91 -> 97 -0.15598
91 -> 98 -0.11962
91 -> 99 -0.24115
91 ->104 0.16210

Excited State 15: Singlet-A 5.7089 eV 217.18 nm f=0.0143 <S**2>=0.000
87 -> 92 0.14659
88 -> 92 0.17888
89 -> 92 0.23354
90 -> 92 0.25998
91 -> 95 -0.13958
91 -> 97 0.14588
91 -> 98 0.14247
91 -> 99 0.34692
91 ->100 0.13807
91 ->101 -0.13675
91 ->104 -0.16648

Excited State 16: Singlet-A 5.7981 eV 213.84 nm f=0.0014 <S**2>=0.000
90 -> 93 -0.14116
90 -> 96 -0.10797
91 -> 98 0.18952
91 -> 99 -0.22167
91 ->100 0.42204
91 ->101 -0.19567
91 ->102 0.12609
91 ->104 0.12202
91 ->105 -0.17002
91 ->108 -0.14411

Excited State 17: Singlet-A 5.8247 eV 212.86 nm f=0.0402 <S**2>=0.000
88 -> 93 0.18635
89 -> 92 -0.13704
89 -> 93 0.17972
90 -> 92 0.12781
90 -> 93 0.22500
91 -> 99 -0.18917
91 ->100 0.23511
91 ->101 0.27489
91 ->107 -0.11302
91 ->108 0.15635

Excited State 18: Singlet-A 5.8338 eV 212.53 nm f=0.0564 <S**2>=0.000
85 -> 93 -0.14386
87 -> 92 -0.11266
88 -> 93 -0.22133
89 -> 92 0.24750
89 -> 93 -0.22600
90 -> 93 -0.13010
91 -> 99 -0.13269
91 ->101 0.32020
91 ->104 -0.10554
91 ->107 -0.10254
91 ->108 0.14936

Excited State 19: Singlet-A 5.8895 eV 210.52 nm f=0.0471 <S**2>=0.000

```
     85 -> 92        -0.11894
     85 -> 93        -0.10348
     86 -> 92         0.17117
     87 -> 92         0.18017
     88 -> 92        -0.25492
     88 -> 93        -0.15670
     89 -> 92        -0.24843
     89 -> 93        -0.17227
     90 -> 92         0.36367
     90 -> 93        -0.23167

 Excited State  20:      Singlet-A      5.9374 eV  208.82 nm  f=0.0846  <S**2>=0.000
     85 -> 93        -0.11416
     86 -> 93        -0.12385
     87 -> 92        -0.12729
     89 -> 93        -0.14541
     90 -> 93         0.19453
     90 -> 96         0.27707
     90 -> 97         0.11488
     91 -> 97        -0.12761
     91 ->101        -0.18281
     91 ->102         0.24758
     91 ->108         0.13416

 Excited State  21:      Singlet-A      6.0049 eV  206.47 nm  f=0.0785  <S**2>=0.000
     86 -> 93         0.11509
     90 -> 96        -0.15251
     90 -> 97        -0.11592
     91 -> 95         0.11289
     91 -> 99        -0.17430
     91 ->101        -0.27338
     91 ->107        -0.16437
     91 ->108         0.33309
     91 ->109         0.11903
     91 ->111         0.11026

 Excited State  22:      Singlet-A      6.0530 eV  204.83 nm  f=0.0810  <S**2>=0.000
     83 -> 92         0.11367
     85 -> 93         0.14151
     87 -> 93         0.18006
     87 -> 95         0.10188
     87 -> 96        -0.12470
     88 -> 93        -0.15443
     88 -> 94        -0.14559
     88 -> 96        -0.12947
     88 -> 97        -0.11023
     89 -> 94        -0.20589
     89 -> 95         0.10401
     90 -> 92         0.13223
     90 -> 93         0.15190
     90 -> 94        -0.13828
     90 -> 95         0.11193
     90 -> 96        -0.12158
     91 ->100        -0.11308
     91 ->102         0.20495

 Excited State  23:      Singlet-A      6.1051 eV  203.08 nm  f=0.1030  <S**2>=0.000
```

82 -> 92 0.11500
83 -> 92 0.26469
85 -> 92 0.17600
85 -> 93 0.26619
86 -> 94 -0.10063
87 -> 93 -0.17804
88 -> 93 -0.20923
90 -> 92 -0.17175
90 -> 93 -0.19688
90 -> 96 0.15382

Excited State 24: Singlet-A 6.1596 eV 201.29 nm f=0.2323 <S**2>=0.000
85 -> 92 0.29590
87 -> 92 0.33678
88 -> 92 -0.20420
88 -> 93 -0.13011
88 -> 94 0.10562
89 -> 93 -0.10075
89 -> 94 -0.12414
90 -> 92 -0.14864
90 -> 93 0.19429
90 -> 94 0.11274

Excited State 25: Singlet-A 6.1866 eV 200.41 nm f=0.0129 <S**2>=0.000
91 ->103 0.56504
91 ->107 -0.11803
91 ->109 -0.17188
91 ->110 -0.11024

(BMK/6-311+G* PCM Toluene, TD=(Nstates=25,root=1))

Excited State 1: Singlet-A 3.4755 eV 356.74 nm f=0.0041 <S**2>=0.000
91 -> 92 0.58681
91 -> 93 0.38083
This state for optimization and/or second-order correction.
Total Energy, E(TD-HF/TD-DFT) = -1088.40381022
Copying the excited state density for this state as the 1-particle RhoCI density.

Excited State 2: Singlet-A 3.5823 eV 346.10 nm f=0.0783 <S**2>=0.000
91 -> 92 -0.38647
91 -> 93 0.57797

Excited State 3: Singlet-A 4.1683 eV 297.44 nm f=0.1674 <S**2>=0.000
91 -> 94 0.68715

Excited State 4: Singlet-A 4.4080 eV 281.27 nm f=0.3000 <S**2>=0.000
91 -> 95 0.66705
91 -> 96 0.18199

Excited State 5: Singlet-A 4.5588 eV 271.97 nm f=0.0723 <S**2>=0.000
91 -> 95 -0.15978
91 -> 96 0.61126
91 -> 97 0.24016

Excited State 6: Singlet-A 4.7960 eV 258.52 nm f=0.0704 <S**2>=0.000
88 -> 94 0.10925
91 -> 96 -0.21894

91 -> 97 0.57896
91 -> 98 0.18287

Excited State 7: Singlet-A 4.8395 eV 256.19 nm f=0.0090 <S**2>=0.000
82 -> 92 -0.12519
83 -> 92 0.11794
84 -> 92 0.56352
84 -> 93 0.18408
85 -> 92 -0.22818
91 -> 97 0.10202

Excited State 8: Singlet-A 4.9134 eV 252.34 nm f=0.0067 <S**2>=0.000
87 -> 95 0.11565
89 -> 94 0.10563
91 -> 96 0.10051
91 -> 97 -0.15698
91 -> 98 0.57773
91 -> 99 -0.19481

Excited State 9: Singlet-A 5.0000 eV 247.97 nm f=0.1436 <S**2>=0.000
83 -> 92 -0.13594
85 -> 93 -0.12393
88 -> 92 0.56825
88 -> 93 0.25765
89 -> 92 -0.14039

Excited State 10: Singlet-A 5.2595 eV 235.73 nm f=0.0232 <S**2>=0.000
87 -> 92 0.22713
88 -> 92 0.17489
88 -> 93 -0.13224
89 -> 92 0.42952
90 -> 92 0.44606

Excited State 11: Singlet-A 5.3165 eV 233.20 nm f=0.2558 <S**2>=0.000
84 -> 93 0.26780
88 -> 92 -0.17692
88 -> 93 0.41281
89 -> 92 0.17708
89 -> 93 -0.32506

Excited State 12: Singlet-A 5.3742 eV 230.70 nm f=0.1040 <S**2>=0.000
84 -> 92 0.14958
84 -> 93 -0.43955
84 -> 96 -0.10509
85 -> 93 0.16080
88 -> 92 -0.16422
88 -> 93 0.35627
90 -> 92 0.18575
90 -> 93 -0.10321

Excited State 13: Singlet-A 5.4298 eV 228.34 nm f=0.0078 <S**2>=0.000
84 -> 92 0.10646
86 -> 92 -0.13764
87 -> 92 -0.21823
88 -> 93 0.10813
89 -> 92 0.48059
89 -> 93 0.12819

```
     90 -> 92       -0.36640

 Excited State  14:      Singlet-A      5.4715 eV  226.60 nm  f=0.0202  <S**2>=0.000
     84 -> 93       -0.13773
     86 -> 93        0.28323
     87 -> 93        0.19159
     88 -> 94        0.16402
     89 -> 93        0.11270
     89 -> 94       -0.14028
     90 -> 93        0.46437
     91 -> 97       -0.12573

 Excited State  15:      Singlet-A      5.5224 eV  224.51 nm  f=0.0311  <S**2>=0.000
     84 -> 93        0.16395
     85 -> 93       -0.11334
     86 -> 93       -0.17708
     88 -> 92       -0.13210
     88 -> 93        0.21533
     89 -> 93        0.48393
     90 -> 93        0.21670

 Excited State  16:      Singlet-A      5.5553 eV  223.18 nm  f=0.0007  <S**2>=0.000
     91 -> 98        0.19043
     91 -> 99        0.57355
     91 ->100        0.22599
     91 ->101       -0.14162
     91 ->102        0.11031

 Excited State  17:      Singlet-A      5.6533 eV  219.31 nm  f=0.0120  <S**2>=0.000
     87 -> 92        0.56706
     87 -> 93        0.13494
     90 -> 92       -0.30013

 Excited State  18:      Singlet-A      5.7173 eV  216.86 nm  f=0.0476  <S**2>=0.000
     86 -> 93        0.23642
     87 -> 92        0.21962
     87 -> 93       -0.22731
     88 -> 94        0.19481
     89 -> 93        0.27986
     89 -> 94       -0.17473
     90 -> 93       -0.25445
     91 -> 98        0.10315
     91 ->103       -0.15695

 Excited State  19:      Singlet-A      5.7329 eV  216.27 nm  f=0.0173  <S**2>=0.000
     86 -> 93        0.19739
     90 -> 93       -0.14444
     91 ->100       -0.25105
     91 ->102       -0.19435
     91 ->103        0.50082

 Excited State  20:      Singlet-A      5.8310 eV  212.63 nm  f=0.0354  <S**2>=0.000
     85 -> 93       -0.10739
     86 -> 92        0.15732
     87 -> 93        0.50848
     90 -> 93       -0.21380
     90 -> 95        0.12521
```

90 -> 96 0.14865
91 ->103 -0.11089

Excited State 21: Singlet-A 5.8548 eV 211.76 nm f=0.0084 <S**2>=0.000
86 -> 92 0.65645
87 -> 93 -0.11207

Excited State 22: Singlet-A 5.9033 eV 210.02 nm f=0.0091 <S**2>=0.000
91 -> 99 -0.19230
91 ->100 0.46170
91 ->101 -0.26733
91 ->102 -0.26468
91 ->103 0.15694
91 ->104 0.11085
91 ->105 -0.13118

Excited State 23: Singlet-A 5.9654 eV 207.84 nm f=0.0181 <S**2>=0.000
83 -> 92 0.10012
84 -> 93 0.10356
85 -> 92 0.18280
85 -> 93 0.13372
87 -> 93 0.28182
87 -> 95 -0.16030
88 -> 94 -0.11679
89 -> 94 -0.25722
89 -> 95 -0.11163
90 -> 93 -0.10305
90 -> 94 -0.15768
90 -> 95 -0.23282
91 -> 98 0.10487
91 ->101 0.13090

Excited State 24: Singlet-A 5.9746 eV 207.52 nm f=0.0069 <S**2>=0.000
85 -> 92 0.11459
89 -> 94 0.12513
90 -> 95 0.14172
91 ->100 0.28214
91 ->101 0.52308
91 ->106 -0.11741

Excited State 25: Singlet-A 5.9832 eV 207.22 nm f=0.0402 <S**2>=0.000
83 -> 92 0.18305
84 -> 92 0.12858
85 -> 92 0.36375
85 -> 93 0.22351
88 -> 92 0.16580
89 -> 94 0.14396
90 -> 95 0.17423
91 ->100 -0.12371
91 ->101 -0.23375

7.1.3 Computed xyz-coordinates (S_1 geometry) of compound 6a (CAM-B3LYP/6-311+G* PCM Toluene)

N 0.813946 -0.405316 0.499543

C	2.048749	0.007338	-0.035240
C	0.157838	0.333362	1.478340
C	0.288716	-1.660302	0.103372
C	-1.061636	-1.758014	-0.383411
C	-1.558801	-3.079725	-0.510137
C	-0.781316	-4.185262	-0.264057
C	0.567755	-4.057578	0.127523
C	1.080126	-2.790674	0.313316
C	3.030385	0.568912	0.788003
C	4.250379	0.932055	0.245153
C	4.497703	0.745457	-1.110118
C	3.519888	0.183592	-1.925487
C	2.300703	-0.194204	-1.395249
C	0.304065	1.730503	1.543978
C	-0.377852	2.445869	2.502601
C	-1.222571	1.792964	3.403942
C	-1.381938	0.416574	3.332190
C	-0.705202	-0.318892	2.373338
C	-1.785924	-0.610124	-0.842262
C	-3.204775	-0.591629	-0.938515
C	-3.860985	0.538006	-1.338919
C	-3.111279	1.694195	-1.655902
C	-1.739234	1.589468	-1.563060
N	-1.060197	0.496118	-1.203062
C	-0.927675	2.741394	-1.887821
N	-0.289379	3.664139	-2.136731
H	-2.565175	-3.226768	-0.883789
H	-1.205932	-5.172938	-0.408323
H	1.184188	-4.931553	0.295172
H	2.095265	-2.656198	0.671663
H	2.843300	0.688354	1.847550
H	5.016594	1.352519	0.885628
H	5.453428	1.035008	-1.530920
H	3.706414	0.049199	-2.984185
H	1.520036	-0.607419	-2.018731
H	0.910793	2.241529	0.808811
H	-0.274914	3.523692	2.537735
H	-1.759529	2.363916	4.152174
H	-2.035965	-0.092558	4.029962
H	-0.804456	-1.394473	2.331357
H	-3.774062	-1.467698	-0.652502
H	-4.942885	0.555150	-1.396846
H	-3.580408	2.615941	-1.972465

SCF Done: E(RCAM-B3LYP) = -1088.65456872 A.U. after 1 cycles
Zero-point correction= 0.346090 (Hartree/Particle)
Thermal correction to Energy= 0.367206
Thermal correction to Enthalpy= 0.368150
Thermal correction to Gibbs Free Energy= 0.295006
Sum of electronic and zero-point Energies= -1088.202783
Sum of electronic and thermal Energies= -1088.181667
Sum of electronic and thermal Enthalpies= -1088.180723
Sum of electronic and thermal Free Energies= -1088.253867

7.1.4 Computed emission energies of compound 6a (CAM-B3LYP/6-311+G* PCM Toluene, TD=(Nstates=6,root=1))

Excited State 1: Singlet-A 2.6820 eV 462.29 nm f=0.0438 <S**2>=0.000
91 -> 92 0.68433

This state for optimization and/or second-order correction.
Total Energy, E(TD-HF/TD-DFT) = -1088.55460354
Copying the excited state density for this state as the 1-particle RhoCI density.

Excited State 2: Singlet-A 3.3967 eV 365.01 nm f=0.0230 <S**2>=0.000
91 -> 92 0.10075
91 -> 93 0.68712

Excited State 3: Singlet-A 4.1174 eV 301.12 nm f=0.0417 <S**2>=0.000
88 -> 92 -0.12001
91 -> 94 -0.62704
91 -> 95 -0.17745

Excited State 4: Singlet-A 4.4498 eV 278.63 nm f=0.1384 <S**2>=0.000
90 -> 92 0.15320
91 -> 96 0.14156
91 -> 97 0.61953
91 ->100 0.12730

Excited State 5: Singlet-A 4.5213 eV 274.22 nm f=0.3967 <S**2>=0.000
87 -> 92 -0.15699
88 -> 92 0.44852
88 -> 93 -0.15986
90 -> 92 0.36842
90 -> 93 -0.14201
91 -> 94 -0.13016
91 -> 97 -0.14973

Excited State 6: Singlet-A 4.5706 eV 271.26 nm f=0.0743 <S**2>=0.000
85 -> 92 -0.13098
90 -> 97 -0.10185
91 -> 95 0.15500
91 -> 96 -0.29063
91 -> 97 0.19221
91 -> 98 0.40559
91 ->100 -0.30580

7.2 Quantum chemical calculation data of 6-(2′-(diphenylamino)-[1,1′-biphenyl]-4-yl)picolinonitrile (6b)

7.2.1 Computed xyz-coordinates (S_0 geometry) of compound 6b

(wB97XD/6-311+G* PCM Toluene)

N	-2.531741	-0.084935	0.421034
C	-3.205677	-1.197125	-0.111195
C	-1.591576	-0.208999	1.473565
C	-2.770414	1.212348	-0.113208
C	-1.710911	1.969105	-0.635681
C	-1.966688	3.266899	-1.079079
C	-3.251502	3.793275	-1.051488
C	-4.302678	3.023020	-0.564402
C	-4.058971	1.743518	-0.086927
C	-3.488477	-2.315397	0.682237
C	-4.151117	-3.409095	0.143507
C	-4.561922	-3.409896	-1.185938
C	-4.296363	-2.294834	-1.972565
C	-3.620473	-1.200913	-1.448103
C	-0.651732	-1.242098	1.478410

C	0.303149	-1.312672	2.481147
C	0.345730	-0.350883	3.486860
C	-0.588777	0.677475	3.483368
C	-1.557348	0.746271	2.489231
C	-0.340805	1.399627	-0.709862
C	0.700027	1.951244	0.035302
C	1.961176	1.375737	0.025013
C	2.210545	0.230109	-0.730988
C	1.176953	-0.303047	-1.503647
C	-0.082686	0.274947	-1.492290
C	3.550256	-0.405177	-0.689276
C	3.714244	-1.785074	-0.876223
C	4.982428	-2.334694	-0.824125
C	6.068695	-1.505704	-0.574257
C	5.804233	-0.154939	-0.382308
N	4.590188	0.391998	-0.436804
C	6.896540	0.749574	-0.102556
N	7.784739	1.446096	0.120937
H	-1.146124	3.856417	-1.476000
H	-3.432687	4.798943	-1.415398
H	-5.309927	3.425184	-0.538801
H	-4.865359	1.141941	0.319314
H	-3.189455	-2.323991	1.724165
H	-4.360448	-4.264773	0.777634
H	-5.085408	-4.264617	-1.600199
H	-4.610099	-2.274157	-3.011421
H	-3.422101	-0.339096	-2.075814
H	-0.660918	-1.975625	0.680334
H	1.033923	-2.115041	2.465224
H	1.102031	-0.402412	4.262661
H	-0.570477	1.432876	4.262326
H	-2.281616	1.554028	2.487867
H	0.508836	2.817743	0.659934
H	2.758824	1.800879	0.622719
H	1.351710	-1.168297	-2.134824
H	-0.878460	-0.160940	-2.087345
H	2.852261	-2.420393	-1.039198
H	5.127081	-3.399801	-0.965108
H	7.080965	-1.886580	-0.521385

SCF Done: E(RwB97XD) = -1319.92084025 A.U. after 2 cycles
Zero-point correction= 0.429707 (Hartree/Particle)
Thermal correction to Energy= 0.455408
Thermal correction to Enthalpy= 0.456353
Thermal correction to Gibbs Free Energy= 0.370599
Sum of electronic and zero-point Energies= -1319.491133
Sum of electronic and thermal Energies= -1319.465432
Sum of electronic and thermal Enthalpies= -1319.464488
Sum of electronic and thermal Free Energies= -1319.550242

(CAM-B3LYP/6-311+G* PCM Toluene)

N	-2.568508	-0.069004	0.240564
C	-3.394907	-0.958187	-0.479426
C	-1.916858	-0.466922	1.434753
C	-2.590599	1.318083	-0.092083
C	-1.403744	2.013527	-0.366032
C	-1.483314	3.385013	-0.618059
C	-2.698248	4.051211	-0.634690

C	-3.870025	3.348034	-0.387159
C	-3.809763	1.992855	-0.108801
C	-4.040530	-2.027704	0.147031
C	-4.845722	-2.888643	-0.583696
C	-5.039426	-2.698084	-1.945729
C	-4.411760	-1.627882	-2.569906
C	-3.594222	-0.769684	-1.850558
C	-1.249816	-1.691444	1.504545
C	-0.606371	-2.074050	2.669972
C	-0.598327	-1.238129	3.781042
C	-1.248675	-0.014774	3.710684
C	-1.909361	0.368179	2.550983
C	-0.075153	1.350048	-0.411458
C	0.997694	1.868246	0.313387
C	2.253657	1.289207	0.244289
C	2.475708	0.166268	-0.550591
C	1.406254	-0.348607	-1.284013
C	0.151732	0.232925	-1.214180
C	3.823399	-0.450029	-0.604164
C	4.005625	-1.802988	-0.917530
C	5.282274	-2.328519	-0.957371
C	6.362187	-1.504982	-0.674194
C	6.084401	-0.183088	-0.355983
N	4.860159	0.338215	-0.320341
C	7.169411	0.715175	-0.038503
N	8.050010	1.407752	0.211258
H	-0.570720	3.928027	-0.837254
H	-2.730163	5.113364	-0.848971
H	-4.827946	3.855662	-0.396011
H	-4.715240	1.438001	0.108542
H	-3.914658	-2.183262	1.210886
H	-5.337789	-3.710549	-0.074903
H	-5.673862	-3.370623	-2.511239
H	-4.551634	-1.459311	-3.632181
H	-3.115445	0.062328	-2.352862
H	-1.236093	-2.341400	0.638129
H	-0.093187	-3.028941	2.705239
H	-0.086821	-1.537043	4.688742
H	-1.253190	0.649258	4.568148
H	-2.421059	1.322220	2.510746
H	0.843382	2.729404	0.953959
H	3.075138	1.702614	0.815307
H	1.550590	-1.196577	-1.943501
H	-0.657498	-0.179964	-1.804185
H	3.151548	-2.439422	-1.107208
H	5.438885	-3.373621	-1.196664
H	7.381026	-1.869251	-0.690450

SCF Done: E(RCAM-B3LYP) = -1319.64112199 A.U. after 2 cycles
Zero-point correction= 0.430414 (Hartree/Particle)
Thermal correction to Energy= 0.456086
Thermal correction to Enthalpy= 0.457030
Thermal correction to Gibbs Free Energy= 0.371066
Sum of electronic and zero-point Energies= -1319.210708
Sum of electronic and thermal Energies= -1319.185036
Sum of electronic and thermal Enthalpies= -1319.184092
Sum of electronic and thermal Free Energies= -1319.270056

(LC-wHPBE/6-311+G* PCM Toluene)

N	-2.556997	-0.073140	0.256189
C	-3.381426	-0.982629	-0.431695
C	-1.839675	-0.452186	1.414056
C	-2.612705	1.306491	-0.082037
C	-1.443571	2.018214	-0.365806
C	-1.541603	3.382930	-0.623666
C	-2.765442	4.027650	-0.638147
C	-3.922408	3.307963	-0.382403
C	-3.841213	1.958067	-0.096090
C	-3.959605	-2.073894	0.215230
C	-4.765423	-2.955833	-0.483891
C	-5.025964	-2.764189	-1.831705
C	-4.465143	-1.671703	-2.474366
C	-3.647065	-0.791831	-1.787642
C	-1.144194	-1.658412	1.452472
C	-0.436284	-2.019968	2.583527
C	-0.391710	-1.179755	3.687008
C	-1.070469	0.026170	3.645343
C	-1.796063	0.387709	2.521437
C	-0.111637	1.363822	-0.414549
C	0.945741	1.859079	0.340223
C	2.199375	1.279896	0.271285
C	2.425567	0.183114	-0.551158
C	1.371545	-0.307926	-1.315177
C	0.119931	0.274383	-1.247054
C	3.772307	-0.436796	-0.604128
C	3.941391	-1.794308	-0.885950
C	5.212261	-2.325675	-0.928702
C	6.296680	-1.500523	-0.679849
C	6.027272	-0.174972	-0.390681
N	4.809681	0.353349	-0.351316
C	7.123223	0.726784	-0.107500
N	8.009074	1.419920	0.114451
H	-0.635705	3.938010	-0.847221
H	-2.816330	5.089083	-0.856981
H	-4.888925	3.801129	-0.390297
H	-4.736970	1.388391	0.129424
H	-3.781311	-2.228468	1.273357
H	-5.206271	-3.797957	0.040522
H	-5.662731	-3.455266	-2.373575
H	-4.658758	-1.501930	-3.528935
H	-3.216155	0.059453	-2.304234
H	-1.159024	-2.310094	0.585523
H	0.100042	-2.963683	2.597186
H	0.171832	-1.462866	4.569783
H	-1.046143	0.694590	4.500251
H	-2.332532	1.330500	2.501064
H	0.780673	2.703063	1.002740
H	3.016426	1.674414	0.864571
H	1.527663	-1.137886	-1.996949
H	-0.686270	-0.116337	-1.858954
H	3.078617	-2.428816	-1.048477
H	5.362283	-3.377801	-1.144376
H	7.315120	-1.868465	-0.700009

SCF Done: E(RLC-wHPBE) = -1319.36081274   A.U. after   2 cycles
Zero-point correction=   0.434008 (Hartree/Particle)
Thermal correction to Energy=   0.459593
Thermal correction to Enthalpy=   0.460537
Thermal correction to Gibbs Free Energy=   0.374822
Sum of electronic and zero-point Energies=   -1318.926805

Sum of electronic and thermal Energies= -1318.901220
Sum of electronic and thermal Enthalpies= -1318.900276
Sum of electronic and thermal Free Energies= -1318.985990

(BMK/6-311+G* PCM Toluene)

N	-2.612376	-0.059364	0.272801
C	-3.381806	-0.990402	-0.452536
C	-1.930919	-0.414138	1.461863
C	-2.637684	1.313188	-0.110996
C	-1.442981	2.009529	-0.396577
C	-1.525926	3.380904	-0.693617
C	-2.751693	4.042650	-0.746524
C	-3.930690	3.336092	-0.492272
C	-3.868182	1.982837	-0.167518
C	-3.992438	-2.090238	0.178849
C	-4.749153	-2.998341	-0.560812
C	-4.929910	-2.829257	-1.936768
C	-4.338230	-1.730723	-2.565145
C	-3.568409	-0.822656	-1.837870
C	-1.227096	-1.627020	1.552070
C	-0.550934	-1.963113	2.723241
C	-0.543935	-1.092073	3.817756
C	-1.227525	0.121523	3.724483
C	-1.921333	0.458957	2.560539
C	-0.107939	1.342374	-0.416086
C	0.981414	1.911848	0.261256
C	2.244952	1.328437	0.207116
C	2.456120	0.150134	-0.523393
C	1.369149	-0.419403	-1.206216
C	0.107422	0.166673	-1.151825
C	3.812938	-0.465577	-0.567405
C	3.996328	-1.842556	-0.811824
C	5.283157	-2.365442	-0.850760
C	6.368813	-1.515532	-0.635118
C	6.080169	-0.172561	-0.380923
N	4.852061	0.343260	-0.346963
C	7.170104	0.754024	-0.134675
N	8.052565	1.464815	0.057598
H	-0.611222	3.921711	-0.921591
H	-2.786771	5.099612	-0.994909
H	-4.893000	3.838982	-0.530721
H	-4.774457	1.426726	0.056051
H	-3.876543	-2.228603	1.248623
H	-5.212906	-3.838230	-0.049865
H	-5.524934	-3.536952	-2.506361
H	-4.468228	-1.577681	-3.633342
H	-3.121199	0.029334	-2.340922
H	-1.210950	-2.300495	0.700938
H	-0.012079	-2.905635	2.774252
H	-0.008863	-1.354360	4.725812
H	-1.232430	0.811187	4.564426
H	-2.455958	1.402108	2.500493
H	0.833942	2.813617	0.849540
H	3.078077	1.778030	0.737268
H	1.505668	-1.308970	-1.814378
H	-0.713265	-0.284960	-1.701664
H	3.139878	-2.493529	-0.947508
H	5.443012	-3.423489	-1.036530
H	7.393776	-1.870205	-0.653544

SCF Done: E(RBMK) = -1319.47495849   A.U. after   2 cycles
Zero-point correction=   0.427832 (Hartree/Particle)
Thermal correction to Energy=   0.453485
Thermal correction to Enthalpy=   0.454429
Thermal correction to Gibbs Free Energy=   0.369149
Sum of electronic and zero-point Energies=   -1319.047127
Sum of electronic and thermal Energies=   -1319.021474
Sum of electronic and thermal Enthalpies=   -1319.020530
Sum of electronic and thermal Free Energies=   -1319.105809

(M062X/6-311+G* PCM Toluene)

N	-2.543247	-0.080848	0.389709
C	-3.261196	-1.166840	-0.144261
C	-1.610802	-0.242886	1.445655
C	-2.753543	1.232054	-0.119193
C	-1.666052	1.991988	-0.579911
C	-1.889395	3.311463	-0.979531
C	-3.168489	3.855215	-0.974831
C	-4.247101	3.079827	-0.556137
C	-4.037296	1.778696	-0.118917
C	-3.567857	-2.284980	0.642687
C	-4.275292	-3.350295	0.101444
C	-4.705088	-3.322661	-1.222865
C	-4.413576	-2.207778	-2.002577
C	-3.694824	-1.140962	-1.476397
C	-0.717138	-1.318187	1.452516
C	0.232677	-1.429048	2.458706
C	0.314395	-0.468327	3.465221
C	-0.575055	0.600816	3.459197
C	-1.537073	0.712873	2.460739
C	-0.304403	1.402455	-0.646854
C	0.751742	1.958600	0.076116
C	2.011510	1.375834	0.051497
C	2.241910	0.220214	-0.697889
C	1.191293	-0.321350	-1.444472
C	-0.067120	0.261721	-1.416352
C	3.582364	-0.415197	-0.679393
C	3.743646	-1.792855	-0.894706
C	5.013346	-2.343523	-0.867352
C	6.102699	-1.517917	-0.614099
C	5.838156	-0.170528	-0.392416
N	4.624686	0.378711	-0.421640
C	6.938998	0.727326	-0.107197
N	7.831206	1.415432	0.118966
H	-1.047797	3.900943	-1.328400
H	-3.324285	4.875648	-1.305149
H	-5.248701	3.494467	-0.550746
H	-4.863089	1.172866	0.238763
H	-3.254153	-2.312105	1.679516
H	-4.503850	-4.204935	0.728784
H	-5.262066	-4.154251	-1.637894
H	-4.740593	-2.165946	-3.035730
H	-3.475561	-0.278213	-2.095555
H	-0.760918	-2.051050	0.654569
H	0.926550	-2.262616	2.446310
H	1.064338	-0.553053	4.242931
H	-0.526227	1.353892	4.237881
H	-2.226446	1.550388	2.454900
H	0.573508	2.835103	0.690479
H	2.823516	1.802061	0.627984

H 1.354208 -1.193049 -2.068906
H -0.877580 -0.174584 -1.991645
H 2.878692 -2.422966 -1.058510
H 5.156756 -3.405145 -1.029895
H 7.116558 -1.895001 -0.579511

SCF Done: E(RM062X) = -1319.86115120 A.U. after 2 cycles
Zero-point correction= 0.429569 (Hartree/Particle)
Thermal correction to Energy= 0.455308
Thermal correction to Enthalpy= 0.456252
Thermal correction to Gibbs Free Energy= 0.371052
Sum of electronic and zero-point Energies= -1319.431582
Sum of electronic and thermal Energies= -1319.405843
Sum of electronic and thermal Enthalpies= -1319.404899
Sum of electronic and thermal Free Energies= -1319.490099

7.2.2 Computed excitations energies of compound 6b

(wB97XD/6-311+G* PCM Toluene, TD=(Nstates=25,root=1))

Excited State 1: Singlet-A 4.1639 eV 297.76 nm f=0.1665 <S**2>=0.000
111 ->112 -0.10255
111 ->113 0.50175
111 ->114 0.26721
111 ->115 -0.35270
This state for optimization and/or second-order correction.
Total Energy, E(TD-HF/TD-DFT) = -1319.76782097
Copying the excited state density for this state as the 1-particle RhoCI density.

Excited State 2: Singlet-A 4.3624 eV 284.21 nm f=0.0590 <S**2>=0.000
111 ->114 0.56035
111 ->115 0.31970

Excited State 3: Singlet-A 4.5072 eV 275.08 nm f=0.2938 <S**2>=0.000
111 ->114 0.10066
111 ->115 -0.10025
111 ->116 0.63805
111 ->118 -0.15727

Excited State 4: Singlet-A 4.6858 eV 264.59 nm f=0.6529 <S**2>=0.000
104 ->112 -0.10419
104 ->113 -0.15495
110 ->112 0.23226
110 ->113 0.56555
111 ->115 -0.10832

Excited State 5: Singlet-A 4.7895 eV 258.86 nm f=0.1337 <S**2>=0.000
111 ->113 -0.29885
111 ->115 -0.34609
111 ->119 0.41747
111 ->121 0.10852

Excited State 6: Singlet-A 4.8536 eV 255.45 nm f=0.2943 <S**2>=0.000
103 ->112 0.17356

104 ->112 -0.14677
110 ->112 0.48789
110 ->113 -0.29848

Excited State 7: Singlet-A 4.9262 eV 251.68 nm f=0.0117 <S**2>=0.000
102 ->112 -0.10077
103 ->112 0.57200
103 ->113 0.28785
110 ->112 -0.12022

Excited State 8: Singlet-A 4.9509 eV 250.43 nm f=0.0343 <S**2>=0.000
106 ->116 -0.11464
109 ->116 0.12897
111 ->119 -0.20084
111 ->121 0.53385

Excited State 9: Singlet-A 5.0137 eV 247.29 nm f=0.0394 <S**2>=0.000
105 ->115 -0.11263
106 ->113 0.17210
107 ->113 0.12539
109 ->113 0.11608
110 ->112 -0.13587
110 ->114 0.13988
110 ->118 0.10215
111 ->112 -0.10846
111 ->113 0.19682
111 ->114 -0.15272
111 ->115 0.17923
111 ->117 0.16160
111 ->118 -0.14211
111 ->119 0.22020
111 ->120 -0.11689
111 ->123 0.10225
111 ->125 -0.12962
111 ->126 -0.14648

Excited State 10: Singlet-A 5.0633 eV 244.87 nm f=0.0265 <S**2>=0.000
106 ->113 0.19856
107 ->113 0.29236
109 ->113 0.22735
110 ->112 -0.11004
110 ->116 0.12067
110 ->117 -0.11881
110 ->118 0.18447
111 ->113 -0.13072
111 ->115 -0.13789
111 ->119 -0.18460
111 ->126 0.10204

Excited State 11: Singlet-A 5.1780 eV 239.45 nm f=0.0052 <S**2>=0.000
111 ->112 0.60145
111 ->113 0.18580
111 ->117 -0.13481
111 ->118 0.16339

Excited State 12: Singlet-A 5.3452 eV 231.95 nm f=0.0031 <S**2>=0.000
102 ->113 -0.11940

103 ->112 -0.28171
103 ->113 0.57829
103 ->115 0.14347

Excited State 13: Singlet-A 5.4793 eV 226.28 nm f=0.0019 <S**2>=0.000
111 ->112 -0.24706
111 ->114 0.15437
111 ->116 0.15002
111 ->117 -0.34811
111 ->118 0.41216
111 ->119 0.10546

Excited State 14: Singlet-A 5.6236 eV 220.47 nm f=0.0089 <S**2>=0.000
111 ->117 0.35369
111 ->118 0.16688
111 ->120 0.40407
111 ->122 0.16381
111 ->124 0.10030
111 ->129 0.10687

Excited State 15: Singlet-A 5.6985 eV 217.57 nm f=0.1227 <S**2>=0.000
104 ->114 0.12643
105 ->113 0.15987
105 ->115 -0.17906
108 ->113 0.11806
108 ->115 -0.21436
110 ->115 0.28611
111 ->118 -0.19009
111 ->119 -0.24640
111 ->122 -0.10742

Excited State 16: Singlet-A 5.7612 eV 215.21 nm f=0.0569 <S**2>=0.000
100 ->112 0.11062
101 ->112 0.12381
102 ->112 -0.12273
102 ->113 0.16818
104 ->113 0.23499
109 ->113 -0.24161
110 ->114 0.30754
110 ->115 -0.14912
110 ->119 -0.13628

Excited State 17: Singlet-A 5.8828 eV 210.76 nm f=0.0083 <S**2>=0.000
102 ->113 -0.11608
107 ->114 0.10999
109 ->116 0.21920
109 ->121 -0.10541
110 ->114 -0.10566
111 ->119 0.13452
111 ->121 -0.13329
111 ->122 -0.10143
111 ->124 0.17382
111 ->126 0.17891
111 ->129 0.10978
111 ->130 0.22295
111 ->131 -0.20866

Excited State 18: Singlet-A 5.9321 eV 209.01 nm f=0.0402 <S**2>=0.000
104 ->114 -0.10194
108 ->116 -0.12337
109 ->112 0.13474
109 ->113 -0.24730
109 ->114 -0.14220
109 ->116 0.28796
109 ->121 0.10305
110 ->118 0.10602
111 ->121 -0.12797
111 ->124 -0.10536
111 ->126 -0.12801
111 ->130 -0.14153
111 ->131 0.15250

Excited State 19: Singlet-A 5.9781 eV 207.40 nm f=0.2212 <S**2>=0.000
102 ->112 -0.21965
102 ->113 -0.13102
104 ->112 -0.19133
106 ->112 0.17795
106 ->113 0.13158
107 ->112 0.26617
107 ->113 0.21177
109 ->112 0.23646
109 ->113 0.14638
110 ->112 -0.12770
110 ->116 -0.13089
110 ->118 -0.14315
110 ->119 -0.10115

Excited State 20: Singlet-A 6.0297 eV 205.62 nm f=0.2370 <S**2>=0.000
100 ->112 0.14184
101 ->112 0.15328
102 ->112 -0.22030
102 ->113 0.20046
108 ->113 0.13718
109 ->113 0.24768
109 ->115 -0.11927
109 ->116 0.17892
110 ->114 -0.18646

Excited State 21: Singlet-A 6.0658 eV 204.40 nm f=0.0644 <S**2>=0.000
102 ->112 0.10577
102 ->113 0.14435
106 ->112 0.24726
107 ->112 0.30645
107 ->113 -0.16834
108 ->113 0.11026
108 ->114 0.10527
109 ->112 0.25075
110 ->116 0.17251
110 ->118 0.14713
110 ->119 0.11592

Excited State 22: Singlet-A 6.1171 eV 202.69 nm f=0.0417 <S**2>=0.000
102 ->112 0.11057
108 ->114 -0.11038

109 ->113 0.15137
111 ->117 0.10301
111 ->120 -0.21655
111 ->122 0.34002
111 ->123 -0.12968
111 ->125 0.16954
111 ->130 0.14757
111 ->132 -0.10712

Excited State 23: Singlet-A 6.1432 eV 201.82 nm f=0.0440 <S**2>=0.000
102 ->112 0.31119
102 ->113 0.11631
104 ->112 0.12947
107 ->116 0.10782
108 ->113 -0.14989
108 ->114 -0.14455
108 ->115 0.10748
110 ->112 0.18277
110 ->116 -0.10499
110 ->118 -0.10434
111 ->120 0.11964
111 ->121 -0.14448
111 ->122 -0.14395
111 ->130 -0.14791

Excited State 24: Singlet-A 6.1557 eV 201.41 nm f=0.0287 <S**2>=0.000
111 ->117 0.13343
111 ->118 0.15042
111 ->120 -0.21082
111 ->121 -0.16385
111 ->123 0.43271
111 ->124 -0.10192
111 ->127 0.12127

Excited State 25: Singlet-A 6.1685 eV 200.99 nm f=0.1104 <S**2>=0.000
102 ->112 -0.17230
104 ->115 0.10821
105 ->114 0.15643
106 ->116 -0.10981
107 ->116 0.11567
108 ->115 0.16708
108 ->116 0.20187
108 ->119 -0.12723
109 ->113 -0.14487
110 ->115 0.19309
111 ->121 -0.15658

(CAM-B3LYP/6-311+G* PCM Toluene, TD=(Nstates=25,root=1))

Excited State 1: Singlet-A 3.9809 eV 311.45 nm f=0.2074 <S**2>=0.000
110 ->113 -0.10095
111 ->112 -0.26779
111 ->113 0.52219
111 ->114 -0.23695
111 ->115 -0.26093
This state for optimization and/or second-order correction.
Total Energy, E(TD-HF/TD-DFT) = -1319.49482744

Copying the excited state density for this state as the 1-particle RhoCI density.

```
Excited State   2:      Singlet-A      4.3303 eV  286.32 nm  f=0.0404  <S**2>=0.000
    110 ->113       -0.11205
    111 ->114        0.53780
    111 ->115       -0.35956
    111 ->116       -0.10972
    111 ->120        0.10550

Excited State   3:      Singlet-A      4.4822 eV  276.62 nm  f=0.2758  <S**2>=0.000
    110 ->113        0.12124
    111 ->112       -0.11058
    111 ->115       -0.10039
    111 ->116        0.62337
    111 ->118       -0.12775
    111 ->119        0.12674

Excited State   4:      Singlet-A      4.5720 eV  271.18 nm  f=0.6804  <S**2>=0.000
    104 ->113        0.17077
    110 ->113        0.57070
    111 ->112       -0.18891
    111 ->115       -0.14055
    111 ->116       -0.16813

Excited State   5:      Singlet-A      4.6678 eV  265.61 nm  f=0.3199  <S**2>=0.000
    104 ->112        0.11058
    110 ->112        0.38060
    110 ->113       -0.12829
    111 ->112       -0.37179
    111 ->115        0.28431
    111 ->119       -0.18302

Excited State   6:      Singlet-A      4.7208 eV  262.63 nm  f=0.1430  <S**2>=0.000
    110 ->112        0.27146
    110 ->113       -0.17616
    111 ->113       -0.29054
    111 ->114       -0.16502
    111 ->115       -0.27910
    111 ->119        0.23962
    111 ->120       -0.25322
    111 ->122       -0.11953

Excited State   7:      Singlet-A      4.8662 eV  254.78 nm  f=0.0512  <S**2>=0.000
    104 ->112        0.11929
    110 ->112        0.36826
    111 ->112        0.44432
    111 ->113        0.22910

Excited State   8:      Singlet-A      4.9366 eV  251.15 nm  f=0.0328  <S**2>=0.000
    103 ->112        0.25305
    103 ->113        0.24599
    106 ->113       -0.12844
    107 ->113        0.13938
    111 ->118        0.16202
    111 ->120       -0.14036
    111 ->121       -0.11726
    111 ->123        0.35682

Excited State   9:      Singlet-A      4.9452 eV  250.72 nm  f=0.0093  <S**2>=0.000
    103 ->112       -0.36950
    103 ->113       -0.34786
```

111 ->123 0.32063

Excited State 10: Singlet-A 4.9875 eV 248.59 nm f=0.0117 <S**2>=0.000
103 ->112 -0.20238
103 ->113 -0.16933
106 ->112 0.12407
106 ->113 -0.21423
107 ->112 -0.14122
107 ->113 0.23491
109 ->112 -0.11354
109 ->113 0.14222
110 ->112 -0.15485
110 ->114 -0.10854
110 ->116 -0.11471
110 ->118 -0.15866
111 ->120 -0.11636
111 ->122 -0.10970
111 ->123 -0.12829

Excited State 11: Singlet-A 5.0320 eV 246.39 nm f=0.0271 <S**2>=0.000
105 ->113 0.10618
107 ->113 0.14380
110 ->115 -0.10085
111 ->113 -0.20927
111 ->114 -0.19513
111 ->115 -0.21612
111 ->117 -0.10197
111 ->119 -0.21109
111 ->120 0.24429
111 ->122 0.12632
111 ->126 0.20646

Excited State 12: Singlet-A 5.2929 eV 234.25 nm f=0.0068 <S**2>=0.000
111 ->117 0.48925
111 ->118 0.36489
111 ->119 0.11343
111 ->120 0.14836
111 ->121 0.11149

Excited State 13: Singlet-A 5.3563 eV 231.47 nm f=0.0025 <S**2>=0.000
103 ->112 -0.44566
103 ->113 0.45688
103 ->114 0.13776
103 ->115 0.13830

Excited State 14: Singlet-A 5.4665 eV 226.81 nm f=0.0081 <S**2>=0.000
110 ->115 -0.12347
111 ->114 0.12499
111 ->116 0.16912
111 ->118 0.28535
111 ->119 -0.23637
111 ->120 -0.25865
111 ->121 -0.17107
111 ->122 -0.18087
111 ->123 -0.19895

Excited State 15: Singlet-A 5.5502 eV 223.39 nm f=0.0831 <S**2>=0.000
104 ->113 0.13837
104 ->115 -0.11403
105 ->113 -0.15502
105 ->114 0.10029

```
   108 ->112       -0.10616
   108 ->113        0.16843
   108 ->115       -0.15464
   110 ->115        0.34241
   110 ->116        0.10472
   111 ->114       -0.12976
   111 ->118        0.17210
   111 ->119       -0.23042

Excited State  16:      Singlet-A      5.6387 eV  219.88 nm  f=0.0304  <S**2>=0.000
   102 ->112        0.10028
   104 ->112       -0.12993
   104 ->113        0.23606
   105 ->115       -0.10765
   109 ->113        0.12989
   110 ->113       -0.10395
   110 ->114        0.33863
   110 ->119        0.12494
   110 ->120       -0.10002
   111 ->117        0.11026
   111 ->120       -0.16355
   111 ->124       -0.13845
   111 ->126       -0.15833

Excited State  17:      Singlet-A      5.7107 eV  217.11 nm  f=0.0017  <S**2>=0.000
   104 ->113        0.13287
   109 ->113        0.10449
   110 ->114        0.14903
   111 ->117       -0.16468
   111 ->118        0.13563
   111 ->119        0.25393
   111 ->120        0.11874
   111 ->121       -0.17694
   111 ->124        0.28675
   111 ->125       -0.15208
   111 ->126        0.11171
   111 ->131        0.10567
   111 ->132       -0.11545

Excited State  18:      Singlet-A      5.7693 eV  214.90 nm  f=0.0021  <S**2>=0.000
   111 ->117       -0.11999
   111 ->118        0.12368
   111 ->119        0.15896
   111 ->120       -0.14609
   111 ->121       -0.12353
   111 ->122        0.54958
   111 ->123       -0.14936

Excited State  19:      Singlet-A      5.8380 eV  212.38 nm  f=0.1139  <S**2>=0.000
   102 ->113       -0.18317
   104 ->112        0.12147
   106 ->112       -0.21363
   106 ->113       -0.16269
   107 ->112        0.28842
   107 ->113        0.25837
   108 ->112        0.12561
   109 ->112        0.26739
   109 ->113        0.14427
   110 ->112       -0.14491

Excited State  20:      Singlet-A      5.8503 eV  211.93 nm  f=0.0644  <S**2>=0.000
```

```
    111 ->119       -0.11635
    111 ->120       -0.23089
    111 ->121        0.37950
    111 ->122        0.11204
    111 ->124        0.19397
    111 ->126        0.24607
    111 ->127        0.12384
    111 ->132       -0.13811
    111 ->133       -0.16816

 Excited State  21:      Singlet-A      5.8979 eV  210.22 nm  f=0.0340  <S**2>=0.000
    104 ->112        0.10570
    109 ->112       -0.20354
    109 ->113        0.27997
    109 ->114       -0.15999
    109 ->116        0.27453
    110 ->114       -0.12352
    110 ->118        0.15407
    110 ->119       -0.13027
    111 ->123        0.11908

 Excited State  22:      Singlet-A      5.9300 eV  209.08 nm  f=0.0614  <S**2>=0.000
    102 ->113        0.13264
    106 ->112       -0.10388
    107 ->112        0.17381
    108 ->113        0.14068
    109 ->113        0.11372
    109 ->116        0.10817
    111 ->117       -0.14853
    111 ->121        0.18831
    111 ->124        0.30489
    111 ->126       -0.10956
    111 ->127        0.11504
    111 ->130       -0.10896
    111 ->132        0.12465
    111 ->133        0.10735

 Excited State  23:      Singlet-A      5.9604 eV  208.01 nm  f=0.0985  <S**2>=0.000
    102 ->112        0.11543
    102 ->113        0.19486
    104 ->112       -0.15153
    106 ->112       -0.19228
    107 ->112        0.24786
    109 ->112        0.10048
    110 ->115        0.10819
    110 ->116       -0.15079
    110 ->118       -0.13548
    111 ->118       -0.10979
    111 ->124       -0.12803
    111 ->126        0.11008
    111 ->132       -0.14517
    111 ->133       -0.12310

 Excited State  24:      Singlet-A      6.0118 eV  206.24 nm  f=0.1788  <S**2>=0.000
    102 ->112       -0.21686
    104 ->112        0.10073
    108 ->112       -0.13338
    108 ->113        0.27604
    108 ->114       -0.11326
    108 ->115       -0.14211
    109 ->113        0.16703
```

109 ->116 -0.22932
110 ->114 0.12487
110 ->115 -0.12902
111 ->120 -0.12927

Excited State 25: Singlet-A 6.0266 eV 205.73 nm f=0.0107 <S**2>=0.000
102 ->112 -0.11326
102 ->113 -0.16266
104 ->113 0.15376
107 ->113 -0.13325
108 ->113 0.18489
108 ->114 -0.16442
109 ->112 0.10937
109 ->113 -0.23333
110 ->112 -0.13303
110 ->114 -0.10046
110 ->115 -0.10066
110 ->116 -0.21382
110 ->118 -0.15617
111 ->123 0.17491

(LC-wHPBE/6-311+G* PCM Toluene, TD=(Nstates=25,root=1))

Excited State 1: Singlet-A 4.5252 eV 273.99 nm f=0.3331 <S**2>=0.000
110 ->113 -0.11110
111 ->112 -0.30419
111 ->113 0.30451
111 ->114 0.32352
111 ->115 -0.28192
111 ->116 0.20210
This state for optimization and/or second-order correction.
Total Energy, E(TD-HF/TD-DFT) = -1319.19451522
Copying the excited state density for this state as the 1-particle RhoCI density.

Excited State 2: Singlet-A 4.6919 eV 264.25 nm f=0.0716 <S**2>=0.000
110 ->112 -0.10243
110 ->113 0.12246
111 ->114 0.44754
111 ->115 0.37946
111 ->117 -0.15096
111 ->123 0.11395

Excited State 3: Singlet-A 4.7948 eV 258.58 nm f=0.2933 <S**2>=0.000
111 ->114 0.10698
111 ->116 -0.16406
111 ->117 0.57531
111 ->121 0.19369

Excited State 4: Singlet-A 4.9547 eV 250.24 nm f=0.7410 <S**2>=0.000
101 ->112 -0.11659
104 ->113 0.22474
110 ->112 -0.17744
110 ->113 0.51036
111 ->115 -0.19716
111 ->117 0.12901

Excited State 5: Singlet-A 5.1381 eV 241.30 nm f=0.2074 <S**2>=0.000
102 ->113 0.13968
103 ->112 0.11546
103 ->113 0.13789

```
    104 ->113       -0.11951
    110 ->112       -0.29262
    110 ->121        0.11208
    111 ->112        0.13050
    111 ->115       -0.17256
    111 ->121       -0.12552
    111 ->122       -0.13912
    111 ->123        0.24565

 Excited State   6:      Singlet-A      5.1490 eV  240.79 nm  f=0.0451  <S**2>=0.000
    102 ->113       -0.12602
    103 ->112       -0.14411
    103 ->113       -0.23073
    105 ->114       -0.10911
    110 ->112        0.25979
    110 ->115        0.11324
    111 ->122       -0.13502
    111 ->123        0.25745
    111 ->125        0.20703

 Excited State   7:      Singlet-A      5.1603 eV  240.26 nm  f=0.0210  <S**2>=0.000
    103 ->112        0.21659
    103 ->113        0.50417
    104 ->113        0.10011
    106 ->113       -0.10777
    107 ->113        0.12056
    109 ->113       -0.10577
    110 ->112        0.14729
    110 ->121       -0.10486

 Excited State   8:      Singlet-A      5.1981 eV  238.52 nm  f=0.0373  <S**2>=0.000
    104 ->114        0.11116
    106 ->117        0.13662
    108 ->114       -0.13585
    109 ->117       -0.14860
    110 ->114       -0.10135
    111 ->121        0.18627
    111 ->125        0.42120

 Excited State   9:      Singlet-A      5.2771 eV  234.95 nm  f=0.1047  <S**2>=0.000
    101 ->112       -0.12113
    102 ->113       -0.12555
    103 ->113        0.14475
    104 ->112        0.16671
    104 ->113        0.10589
    106 ->112       -0.12387
    106 ->113        0.14963
    107 ->112        0.17415
    107 ->113       -0.16319
    109 ->112       -0.17205
    109 ->113        0.10045
    110 ->112        0.30714
    110 ->117       -0.12871
    110 ->121        0.15675

 Excited State  10:      Singlet-A      5.6223 eV  220.52 nm  f=0.0039  <S**2>=0.000
    102 ->112        0.16060
    103 ->112        0.55949
    103 ->113       -0.23290
    103 ->114        0.14444
    103 ->115       -0.13873
```

```
Excited State  11:      Singlet-A      5.6790 eV  218.32 nm  f=0.0195  <S**2>=0.000
    108 ->112         0.10051
    108 ->115         0.14659
    110 ->112         0.10688
    110 ->115        -0.14784
    111 ->112         0.29170
    111 ->113        -0.27214
    111 ->115        -0.16141
    111 ->116         0.11291
    111 ->129         0.15899
    111 ->130        -0.13964
    111 ->131        -0.11259

Excited State  12:      Singlet-A      5.9824 eV  207.25 nm  f=0.1370  <S**2>=0.000
    101 ->113         0.12959
    102 ->112        -0.21900
    104 ->112         0.25697
    104 ->113        -0.13685
    110 ->113         0.12876
    110 ->114         0.21392
    110 ->115        -0.19499
    110 ->116         0.12130
    110 ->123        -0.11753
    111 ->112        -0.18042
    111 ->114        -0.13399

Excited State  13:      Singlet-A      6.0799 eV  203.92 nm  f=0.0594  <S**2>=0.000
    105 ->115         0.13955
    108 ->115        -0.15327
    109 ->117        -0.17370
    110 ->114         0.14499
    110 ->115         0.10092
    111 ->114         0.22819
    111 ->118        -0.12982
    111 ->122         0.13783
    111 ->129         0.16133
    111 ->130        -0.10814
    111 ->134        -0.13348

Excited State  14:      Singlet-A      6.1093 eV  202.94 nm  f=0.0441  <S**2>=0.000
    111 ->112         0.12949
    111 ->113         0.17064
    111 ->115         0.19028
    111 ->116         0.30055
    111 ->117         0.11846
    111 ->118         0.38489

Excited State  15:      Singlet-A      6.2091 eV  199.68 nm  f=0.2107  <S**2>=0.000
    108 ->117         0.10027
    109 ->117         0.13108
    111 ->112        -0.25009
    111 ->113        -0.18336
    111 ->114        -0.10335
    111 ->116         0.14917
    111 ->120        -0.11922
    111 ->121         0.28583
    111 ->130        -0.11470
    111 ->134        -0.11198
    111 ->136         0.10103
```

Excited State 16:   Singlet-A   6.2365 eV 198.80 nm f=0.0352 <S**2>=0.000
    102 ->112   0.19398
    104 ->115   0.10925
    108 ->115   0.12545
    109 ->121   0.10459
    110 ->115   -0.12964
    110 ->121   0.10020
    111 ->113   0.19360
    111 ->114   -0.13322
    111 ->116   -0.13520
    111 ->121   -0.16375
    111 ->134   -0.12348
    111 ->137   0.12603

Excited State 17:   Singlet-A   6.3501 eV 195.25 nm f=0.0634 <S**2>=0.000
    102 ->112   -0.19235
    104 ->115   -0.10794
    109 ->112   0.12824
    109 ->113   -0.10137
    109 ->114   -0.12461
    109 ->117   0.28342
    110 ->117   -0.10747
    111 ->112   0.13696
    111 ->113   0.10413
    111 ->122   0.12350
    111 ->123   -0.16173
    111 ->125   0.13626

Excited State 18:   Singlet-A   6.3809 eV 194.31 nm f=0.0944 <S**2>=0.000
    102 ->112   -0.14520
    107 ->112   0.11335
    107 ->113   -0.13469
    108 ->114   0.14576
    110 ->117   0.12124
    110 ->121   -0.20107
    111 ->112   0.17183
    111 ->113   0.30666

Excited State 19:   Singlet-A   6.3997 eV 193.73 nm f=0.4754 <S**2>=0.000
    101 ->113   -0.10875
    102 ->112   0.18354
    108 ->114   -0.13388
    109 ->117   0.11699
    109 ->125   -0.13616
    110 ->114   0.17479
    111 ->112   0.27306
    111 ->113   0.22945
    111 ->115   -0.10574
    111 ->121   0.17112

Excited State 20:   Singlet-A   6.5356 eV 189.71 nm f=0.0695 <S**2>=0.000
    102 ->113   -0.10359
    107 ->117   -0.12778
    108 ->115   -0.13575
    108 ->116   0.11861
    108 ->117   -0.10619
    109 ->114   -0.11853
    109 ->125   0.10320
    110 ->121   0.11038
    111 ->119   -0.16596
    111 ->121   0.20022

111 ->122 -0.14940
111 ->123 0.19835

Excited State 21: Singlet-A 6.5422 eV 189.51 nm f=0.4344 <S**2>=0.000
101 ->112 -0.12561
106 ->125 0.17824
107 ->112 -0.11596
107 ->121 0.10828
107 ->123 0.13817
108 ->125 0.13292
109 ->114 0.28092
109 ->117 0.14749
109 ->121 -0.14729
109 ->123 0.10014
109 ->125 -0.10406

Excited State 22: Singlet-A 6.5663 eV 188.82 nm f=0.5791 <S**2>=0.000
102 ->113 -0.19772
106 ->121 0.13824
107 ->117 0.15912
110 ->112 -0.11299
111 ->118 -0.10504
111 ->119 0.14937
111 ->120 -0.14109
111 ->121 0.24962
111 ->125 -0.15260

Excited State 23: Singlet-A 6.6031 eV 187.77 nm f=0.5350 <S**2>=0.000
101 ->112 0.13249
106 ->117 -0.17277
107 ->114 0.13170
107 ->121 -0.12344
107 ->125 0.10989
108 ->114 0.13451
108 ->125 0.10575
109 ->123 0.11978
111 ->119 -0.14064
111 ->120 0.16753
111 ->123 -0.13972
111 ->125 0.20165

Excited State 24: Singlet-A 6.6394 eV 186.74 nm f=0.1127 <S**2>=0.000
108 ->117 -0.11068
111 ->119 0.27380
111 ->120 -0.20697
111 ->123 0.18000
111 ->124 0.17688
111 ->125 0.25578
111 ->127 0.10598
111 ->128 -0.13622
111 ->138 -0.10898

Excited State 25: Singlet-A 6.6783 eV 185.65 nm f=0.0261 <S**2>=0.000
111 ->116 0.21261
111 ->118 -0.23687
111 ->119 0.13996
111 ->120 0.38322
111 ->122 0.13836
111 ->124 0.13708
111 ->126 0.12390
111 ->131 0.11697

111 ->137 -0.11132

(BMK/6-311+G* PCM Toluene, TD=(Nstates=25,root=1))

```
Excited State   1:      Singlet-A      3.6154 eV  342.94 nm  f=0.1152  <S**2>=0.000
     111 ->112        -0.46359
     111 ->113         0.46813
     111 ->114         0.16853
     111 ->115         0.16538
 This state for optimization and/or second-order correction.
 Total Energy, E(TD-HF/TD-DFT) =  -1319.34209592
 Copying the excited state density for this state as the 1-particle RhoCI density.

 Excited State   2:      Singlet-A      4.0752 eV  304.24 nm  f=0.0010  <S**2>=0.000
     111 ->112         0.50273
     111 ->113         0.46862
     111 ->114         0.10106

 Excited State   3:      Singlet-A      4.1824 eV  296.44 nm  f=0.0451  <S**2>=0.000
     111 ->114         0.49653
     111 ->115        -0.44915

 Excited State   4:      Singlet-A      4.3732 eV  283.51 nm  f=0.3284  <S**2>=0.000
     111 ->114        -0.10613
     111 ->116         0.64256
     111 ->117         0.21416

 Excited State   5:      Singlet-A      4.4465 eV  278.84 nm  f=0.1330  <S**2>=0.000
     111 ->112         0.11526
     111 ->113        -0.18286
     111 ->114         0.36190
     111 ->115         0.45986
     111 ->117         0.18807
     111 ->118        -0.21606

 Excited State   6:      Singlet-A      4.4675 eV  277.52 nm  f=0.9688  <S**2>=0.000
     104 ->113         0.10607
     110 ->112        -0.31244
     110 ->113         0.59086
     111 ->115         0.10263

 Excited State   7:      Singlet-A      4.5892 eV  270.16 nm  f=0.1061  <S**2>=0.000
     104 ->113         0.13378
     110 ->112         0.58112
     110 ->113         0.27261

 Excited State   8:      Singlet-A      4.7993 eV  258.34 nm  f=0.0299  <S**2>=0.000
     111 ->114        -0.11113
     111 ->115        -0.13407
     111 ->116        -0.15453
     111 ->117         0.46572
     111 ->118        -0.13317
     111 ->119         0.33686

 Excited State   9:      Singlet-A      4.8102 eV  257.75 nm  f=0.0434  <S**2>=0.000
     111 ->114         0.15103
     111 ->118         0.56298
     111 ->119         0.23067

 Excited State  10:      Singlet-A      4.8382 eV  256.26 nm  f=0.0066  <S**2>=0.000
     103 ->112         0.30487
```

103 ->113 0.57681

Excited State 11: Singlet-A 4.9114 eV 252.44 nm f=0.0064 <S**2>=0.000
103 ->112 -0.10422
103 ->113 -0.12760
106 ->112 0.20110
106 ->113 -0.19543
107 ->112 0.22758
107 ->113 -0.21383
109 ->112 0.22556
109 ->113 -0.18932
110 ->116 -0.17116
110 ->117 0.19415
111 ->119 0.24425

Excited State 12: Singlet-A 5.0995 eV 243.13 nm f=0.0126 <S**2>=0.000
107 ->112 -0.10314
109 ->112 -0.10531
109 ->113 0.10088
111 ->116 0.17155
111 ->117 -0.40374
111 ->118 -0.18357
111 ->119 0.41873

Excited State 13: Singlet-A 5.2675 eV 235.38 nm f=0.0013 <S**2>=0.000
103 ->112 0.57893
103 ->113 -0.30038
103 ->114 0.14796
103 ->115 0.11152

Excited State 14: Singlet-A 5.3647 eV 231.11 nm f=0.0182 <S**2>=0.000
104 ->115 -0.11589
105 ->112 -0.14956
105 ->113 0.17471
105 ->114 0.13403
108 ->112 -0.24558
108 ->113 0.23592
108 ->115 0.15636
110 ->115 0.35947
111 ->114 -0.12175
111 ->117 0.11378
111 ->118 -0.13673

Excited State 15: Singlet-A 5.4289 eV 228.38 nm f=0.0192 <S**2>=0.000
104 ->112 -0.11058
104 ->113 0.13220
106 ->113 0.20957
107 ->113 0.19610
108 ->112 0.15036
108 ->113 -0.12179
109 ->112 0.41926
109 ->113 -0.21648
110 ->114 -0.24108

Excited State 16: Singlet-A 5.4671 eV 226.78 nm f=0.0538 <S**2>=0.000
103 ->112 0.11120
104 ->113 -0.12104
106 ->112 0.21536
106 ->113 0.15381
107 ->112 0.28976
107 ->113 0.21730

108 ->113 0.13078
109 ->112 0.23673
109 ->113 0.39960

Excited State 17: Singlet-A 5.5630 eV 222.87 nm f=0.0068 <S**2>=0.000
111 ->120 0.48692
111 ->121 0.33136
111 ->122 -0.29023

Excited State 18: Singlet-A 5.5766 eV 222.33 nm f=0.0236 <S**2>=0.000
104 ->112 0.16611
104 ->113 -0.25001
106 ->112 -0.13510
107 ->112 -0.13410
109 ->112 0.20713
109 ->113 -0.19772
110 ->114 0.37193
110 ->117 -0.10217
111 ->121 -0.18622

Excited State 19: Singlet-A 5.6089 eV 221.05 nm f=0.0391 <S**2>=0.000
106 ->113 -0.10203
107 ->112 -0.13540
107 ->113 0.10404
108 ->112 0.27476
108 ->113 -0.20962
109 ->113 0.10471
110 ->115 0.29125
110 ->116 -0.15617
111 ->121 0.22098
111 ->122 0.16667
111 ->124 0.10674

Excited State 20: Singlet-A 5.6470 eV 219.56 nm f=0.0051 <S**2>=0.000
108 ->112 -0.22169
108 ->113 0.22840
109 ->112 0.12989
111 ->120 -0.10278
111 ->121 0.30394
111 ->122 0.31734
111 ->124 0.14464
111 ->126 0.13418
111 ->127 0.14080

Excited State 21: Singlet-A 5.7300 eV 216.38 nm f=0.0464 <S**2>=0.000
100 ->112 -0.15820
102 ->113 -0.26360
104 ->112 0.18205
104 ->113 0.29578
106 ->112 0.13837
106 ->113 0.10486
107 ->112 0.13937
108 ->113 -0.11711
110 ->113 -0.16010
110 ->114 0.28797
110 ->115 0.18210

Excited State 22: Singlet-A 5.7882 eV 214.20 nm f=0.1216 <S**2>=0.000
104 ->112 0.18222
104 ->113 -0.15989
107 ->112 0.28174

107 ->113 -0.25706
108 ->112 0.14760
108 ->113 -0.12100
109 ->116 0.10350
110 ->114 -0.16557
110 ->115 0.15057
110 ->116 0.26764
110 ->117 -0.17208

Excited State 23: Singlet-A 5.8208 eV 213.00 nm f=0.0588 <S**2>=0.000
102 ->112 -0.12536
104 ->112 0.34963
104 ->113 -0.19470
106 ->112 0.25444
107 ->113 0.26221
108 ->112 -0.11493
108 ->113 -0.10562
109 ->112 -0.10761
109 ->113 -0.16683
110 ->114 -0.24125
110 ->115 -0.14343

Excited State 24: Singlet-A 5.8449 eV 212.13 nm f=0.0381 <S**2>=0.000
102 ->113 -0.14977
104 ->112 0.27082
106 ->113 -0.25693
107 ->112 -0.23629
107 ->113 -0.15100
109 ->112 0.29171
109 ->113 0.29966
109 ->116 -0.11323
110 ->114 -0.10535

Excited State 25: Singlet-A 5.8696 eV 211.23 nm f=0.0425 <S**2>=0.000
105 ->112 -0.12809
105 ->113 0.13549
106 ->112 0.21146
106 ->113 -0.16327
106 ->116 -0.10979
107 ->112 -0.15374
108 ->112 0.10859
108 ->114 0.12893
109 ->116 0.21701
110 ->116 0.28445
111 ->119 0.16419
111 ->121 0.10752
111 ->124 -0.12576

(M062X/6-311+G* PCM Toluene, TD=(Nstates=25,root=1))

Excited State 1: Singlet-A 3.9015 eV 317.79 nm f=0.1186 <S**2>=0.000
111 ->112 0.37236
111 ->113 0.48607
111 ->114 0.18512
111 ->116 0.25936
This state for optimization and/or second-order correction.
Total Energy, E(TD-HF/TD-DFT) = -1319.71777350
Copying the excited state density for this state as the 1-particle RhoCI density.

Excited State 2: Singlet-A 4.2380 eV 292.55 nm f=0.0559 <S**2>=0.000
111 ->114 0.55330

```
     111 ->115         0.20099
     111 ->116        -0.30601
     111 ->117         0.10529

 Excited State   3:      Singlet-A      4.3694 eV  283.76 nm  f=0.2279  <S**2>=0.000
     111 ->112         0.24751
     111 ->113        -0.12405
     111 ->114        -0.16464
     111 ->115         0.18372
     111 ->117         0.54967

 Excited State   4:      Singlet-A      4.5275 eV  273.85 nm  f=0.1392  <S**2>=0.000
     110 ->113         0.16150
     111 ->112         0.50233
     111 ->113        -0.28897
     111 ->116        -0.14065
     111 ->117        -0.22181
     111 ->120         0.13810

 Excited State   5:      Singlet-A      4.6037 eV  269.31 nm  f=0.2730  <S**2>=0.000
     110 ->113         0.28559
     111 ->113        -0.25022
     111 ->114         0.18332
     111 ->116         0.43641
     111 ->120        -0.12997
     111 ->122        -0.20683

 Excited State   6:      Singlet-A      4.6297 eV  267.80 nm  f=0.6396  <S**2>=0.000
     104 ->113        -0.10659
     110 ->112         0.20229
     110 ->113         0.53826
     111 ->113         0.25370
     111 ->114        -0.13007
     111 ->116        -0.12926

 Excited State   7:      Singlet-A      4.7724 eV  259.79 nm  f=0.1556  <S**2>=0.000
     102 ->113        -0.12239
     104 ->112        -0.10552
     104 ->113         0.11636
     110 ->112         0.58918
     110 ->113        -0.10805
     111 ->112         0.10142

 Excited State   8:      Singlet-A      4.8211 eV  257.17 nm  f=0.0086  <S**2>=0.000
     102 ->112        -0.30686
     102 ->113         0.56667

 Excited State   9:      Singlet-A      4.9071 eV  252.66 nm  f=0.0334  <S**2>=0.000
     111 ->113        -0.13080
     111 ->114         0.11780
     111 ->115         0.19415
     111 ->116         0.15286
     111 ->118         0.19940
     111 ->119         0.16373
     111 ->122         0.33469
     111 ->125        -0.28491
     111 ->129        -0.14198

 Excited State  10:      Singlet-A      4.9138 eV  252.32 nm  f=0.0465  <S**2>=0.000
     109 ->117         0.11531
     110 ->114        -0.10240
```

```
     111 ->116        0.10001
     111 ->120        0.18365
     111 ->121        0.18506
     111 ->122        0.27039
     111 ->124        0.18208
     111 ->125        0.41858

 Excited State  11:      Singlet-A      5.0060 eV  247.67 nm  f=0.0049  <S**2>=0.000
     104 ->112        0.10262
     105 ->113        0.12606
     106 ->112       -0.11415
     106 ->113       -0.16805
     107 ->112       -0.17089
     107 ->113       -0.20552
     109 ->112        0.23319
     109 ->113        0.25384
     110 ->112       -0.14437
     110 ->114       -0.11214
     110 ->117        0.17483
     110 ->120        0.14214
     111 ->118        0.11941
     111 ->120       -0.11855

 Excited State  12:      Singlet-A      5.0454 eV  245.74 nm  f=0.0220  <S**2>=0.000
     111 ->114       -0.10822
     111 ->115        0.42478
     111 ->117       -0.21116
     111 ->118        0.32207
     111 ->121        0.10338
     111 ->122       -0.16654
     111 ->125        0.14413

 Excited State  13:      Singlet-A      5.2628 eV  235.59 nm  f=0.0113  <S**2>=0.000
     111 ->114       -0.12207
     111 ->115        0.15928
     111 ->117        0.14808
     111 ->118       -0.19481
     111 ->119       -0.18327
     111 ->120        0.33602
     111 ->121        0.30542
     111 ->122       -0.20372
     111 ->125       -0.18529

 Excited State  14:      Singlet-A      5.4024 eV  229.50 nm  f=0.0020  <S**2>=0.000
     102 ->112        0.48307
     102 ->113        0.24367
     102 ->116       -0.10526
     111 ->115       -0.13500
     111 ->118        0.19981
     111 ->119       -0.21301
     111 ->123        0.14614

 Excited State  15:      Singlet-A      5.4044 eV  229.41 nm  f=0.0010  <S**2>=0.000
     102 ->112        0.32735
     102 ->113        0.16463
     111 ->115        0.19127
     111 ->118       -0.26698
     111 ->119        0.33075
     111 ->123       -0.21558
     111 ->124       -0.10409
```

Excited State 16:   Singlet-A   5.4856 eV 226.02 nm f=0.0057 <S**2>=0.000
    111 ->115   -0.10628
    111 ->118   0.23379
    111 ->119   0.22862
    111 ->120   0.45891
    111 ->121   -0.18847
    111 ->122   -0.19903
    111 ->124   -0.11043
    111 ->126   -0.11374

Excited State 17:   Singlet-A   5.5715 eV 222.53 nm f=0.0132 <S**2>=0.000
    108 ->113   0.11975
    108 ->116   0.11364
    110 ->116   -0.17719
    111 ->119   0.21507
    111 ->121   0.29225
    111 ->123   0.36233
    111 ->126   0.16394

Excited State 18:   Singlet-A   5.5828 eV 222.08 nm f=0.0474 <S**2>=0.000
    104 ->113   0.11358
    105 ->113   -0.13703
    108 ->112   -0.12796
    108 ->113   -0.15919
    108 ->116   -0.14297
    109 ->112   0.12175
    109 ->113   0.11119
    110 ->116   0.27201
    111 ->119   0.27799
    111 ->120   -0.10325
    111 ->122   -0.17726
    111 ->123   0.16871

Excited State 19:   Singlet-A   5.6182 eV 220.68 nm f=0.0364 <S**2>=0.000
    104 ->113   0.11879
    105 ->116   0.10082
    106 ->113   0.12333
    108 ->116   0.11230
    109 ->112   0.23518
    109 ->113   0.27360
    110 ->114   0.35018
    110 ->122   0.12496
    111 ->119   -0.12173
    111 ->122   0.13464

Excited State 20:   Singlet-A   5.7272 eV 216.48 nm f=0.0446 <S**2>=0.000
    104 ->113   -0.16528
    106 ->113   0.12161
    107 ->112   -0.11793
    107 ->113   0.24074
    109 ->112   0.40195
    109 ->113   -0.18356
    109 ->117   0.19569
    110 ->114   -0.12679
    111 ->124   -0.13391

Excited State 21:   Singlet-A   5.7721 eV 214.80 nm f=0.0455 <S**2>=0.000
    104 ->112   0.15875
    106 ->112   -0.11920
    107 ->112   -0.22977
    109 ->112   0.13631

109 ->113 -0.32529
109 ->117 -0.10135
110 ->114 0.15831
111 ->124 0.26998
111 ->128 0.17243

Excited State 22: Singlet-A 5.7999 eV 213.77 nm f=0.0406 <S**2>=0.000
108 ->113 0.10163
110 ->116 0.13961
111 ->116 0.10451
111 ->127 0.16323
111 ->128 -0.12191
111 ->129 0.34146
111 ->130 0.17742
111 ->131 -0.11775
111 ->134 0.13168
111 ->135 0.19903
111 ->136 -0.12240
111 ->137 -0.12819

Excited State 23: Singlet-A 5.8061 eV 213.54 nm f=0.0066 <S**2>=0.000
104 ->112 -0.13401
104 ->113 -0.12847
109 ->112 0.10386
109 ->113 0.22376
110 ->114 -0.14530
111 ->121 -0.14738
111 ->124 0.32969
111 ->125 -0.10607
111 ->128 0.22633
111 ->130 0.17363
111 ->134 0.15693

Excited State 24: Singlet-A 5.8602 eV 211.57 nm f=0.0421 <S**2>=0.000
103 ->113 -0.10320
104 ->112 -0.11737
107 ->112 -0.14962
107 ->113 -0.16775
108 ->112 0.21360
108 ->113 0.26513
108 ->114 0.11854
110 ->117 -0.28122
110 ->120 -0.14604
111 ->129 -0.13595
111 ->134 -0.11507
111 ->135 -0.10788

Excited State 25: Singlet-A 5.9163 eV 209.56 nm f=0.0100 <S**2>=0.000
101 ->112 -0.11220
103 ->113 -0.13813
104 ->113 0.17964
109 ->112 -0.11434
109 ->113 -0.13401
109 ->115 0.14173
109 ->117 0.28248
111 ->122 -0.12011
111 ->123 0.11819
111 ->124 0.13648
111 ->125 -0.18169
111 ->127 0.17934

7.2.3 Computed xyz-coordinates (S_1 geometry) of compound 6b

(CAM-B3LYP/6-311+G* PCM Toluene)

N	-2.594289	-0.041147	0.229283
C	-3.445804	-0.991104	-0.376383
C	-1.911649	-0.349760	1.421700
C	-2.589476	1.277546	-0.251646
C	-1.346066	2.010931	-0.380302
C	-1.490597	3.414581	-0.550872
C	-2.710821	4.009619	-0.735061
C	-3.901352	3.246226	-0.742241
C	-3.825885	1.898094	-0.490830
C	-4.204742	-1.872601	0.400622
C	-5.043542	-2.784343	-0.217182
C	-5.138180	-2.828429	-1.603318
C	-4.391719	-1.943508	-2.375252
C	-3.553902	-1.024149	-1.770695
C	-1.404042	-1.636427	1.640720
C	-0.718372	-1.915193	2.805181
C	-0.515945	-0.918290	3.759438
C	-1.014799	0.358099	3.541418
C	-1.713347	0.649048	2.380767
C	-0.065991	1.381165	-0.467003
C	1.132687	2.062965	-0.099175
C	2.355543	1.457119	-0.154591
C	2.510920	0.121669	-0.614010
C	1.337402	-0.539217	-1.034016
C	0.104129	0.056764	-0.965501
C	3.822792	-0.492946	-0.659366
C	4.036100	-1.823708	-1.095432
C	5.304752	-2.351347	-1.118806
C	6.383888	-1.569552	-0.705378
C	6.088416	-0.279010	-0.288811
N	4.873547	0.254779	-0.259638
C	7.164979	0.578168	0.155972
N	8.037344	1.238370	0.505010
H	-0.596948	4.020714	-0.629825
H	-2.760242	5.078809	-0.909265
H	-4.861639	3.722228	-0.896771
H	-4.730917	1.307670	-0.403986
H	-4.150746	-1.823372	1.480334
H	-5.638411	-3.456661	0.389859
H	-5.796821	-3.544218	-2.080769
H	-4.459133	-1.973291	-3.456266
H	-2.973404	-0.333736	-2.369437
H	-1.522581	-2.397860	0.880670
H	-0.317036	-2.908922	2.964515
H	0.032405	-1.140238	4.667271
H	-0.866650	1.134431	4.282639
H	-2.130241	1.634633	2.221604
H	1.073659	3.071658	0.293029
H	3.237428	1.994887	0.167463
H	1.390058	-1.537745	-1.451692
H	-0.746703	-0.482280	-1.360194
H	3.203182	-2.436050	-1.412441
H	5.465993	-3.369363	-1.454724
H	7.400271	-1.939767	-0.704254

SCF Done: E(RCAM-B3LYP) = -1319.62593683 A.U. after 2 cycles

Zero-point correction= 0.427883 (Hartree/Particle)
Thermal correction to Energy= 0.453663
Thermal correction to Enthalpy= 0.454607
Thermal correction to Gibbs Free Energy= 0.369780
Sum of electronic and zero-point Energies= -1319.083915
Sum of electronic and thermal Energies= -1319.058135
Sum of electronic and thermal Enthalpies= -1319.057191
Sum of electronic and thermal Free Energies= -1319.142017

(BMK/6-311+G* PCM Toluene)

N	-2.642793	-0.042955	0.275579
C	-3.440188	-0.997436	-0.374400
C	-1.962088	-0.339151	1.455847
C	-2.630990	1.286688	-0.215694
C	-1.383040	1.985368	-0.426980
C	-1.518512	3.378582	-0.693361
C	-2.748158	3.998629	-0.824919
C	-3.952742	3.266736	-0.698689
C	-3.877165	1.917909	-0.389522
C	-4.175588	-1.950366	0.362624
C	-4.974749	-2.870884	-0.306710
C	-5.054457	-2.857777	-1.703800
C	-4.335947	-1.902794	-2.434969
C	-3.540662	-0.969742	-1.781678
C	-1.449084	-1.636571	1.691051
C	-0.757810	-1.901733	2.862391
C	-0.552169	-0.887451	3.810656
C	-1.045846	0.399901	3.575265
C	-1.743735	0.682874	2.407602
C	-0.085281	1.342193	-0.470855
C	1.107102	2.047170	-0.107365
C	2.348268	1.456122	-0.154930
C	2.525179	0.106973	-0.595069
C	1.347177	-0.584242	-0.994263
C	0.099551	0.001532	-0.932632
C	3.847600	-0.493745	-0.640509
C	4.076400	-1.840782	-1.064207
C	5.356965	-2.355363	-1.090825
C	6.439622	-1.548821	-0.694195
C	6.119045	-0.249013	-0.291496
N	4.898799	0.272691	-0.258116
C	7.193905	0.633636	0.134819
N	8.063560	1.308936	0.467190
H	-0.617393	3.956577	-0.872286
H	-2.785297	5.059070	-1.060600
H	-4.916158	3.752155	-0.814205
H	-4.781689	1.338886	-0.222533
H	-4.141599	-1.937755	1.446680
H	-5.551624	-3.591351	0.265483
H	-5.680698	-3.579636	-2.219520
H	-4.393647	-1.888954	-3.519118
H	-2.982223	-0.227595	-2.342701
H	-1.551132	-2.404070	0.932132
H	-0.348033	-2.893096	3.029013
H	-0.000164	-1.100281	4.721216
H	-0.888899	1.186082	4.307263
H	-2.149682	1.672245	2.230411
H	1.030380	3.065797	0.263529
H	3.223220	2.016324	0.155967
H	1.410222	-1.594145	-1.387713

H -0.747340 -0.564160 -1.306530
H 3.246045 -2.467768 -1.367482
H 5.528719 -3.378088 -1.415361
H 7.464426 -1.901592 -0.695347

SCF Done: E(RBMK) = -1319.46390972 A.U. after 1 cycles
Zero-point correction= 0.426635 (Hartree/Particle)
Thermal correction to Energy= 0.452220
Thermal correction to Enthalpy= 0.453164
Thermal correction to Gibbs Free Energy= 0.369479
Sum of electronic and zero-point Energies= -1318.929416
Sum of electronic and thermal Energies= -1318.903832
Sum of electronic and thermal Enthalpies= -1318.902887
Sum of electronic and thermal Free Energies= -1318.986573

7.2.4 Computed emission energies of compound 6b

(CAM-B3LYP/6-311+G* PCM Toluene, TD=(Nstates=6,root=1))

Excited State 1: Singlet-A 2.9481 eV 420.55 nm f=0.2214 <S**2>=0.000
111 ->112 0.65214
111 ->114 -0.22642
This state for optimization and/or second-order correction.
Total Energy, E(TD-HF/TD-DFT) = -1319.51762118
Copying the excited state density for this state as the 1-particle RhoCI density.

Excited State 2: Singlet-A 3.9029 eV 317.67 nm f=0.5456 <S**2>=0.000
110 ->112 0.60699
110 ->113 0.14197
111 ->113 -0.15487
111 ->115 0.18501

Excited State 3: Singlet-A 4.0189 eV 308.50 nm f=0.1540 <S**2>=0.000
110 ->112 -0.23766
110 ->113 0.23765
111 ->113 -0.55083
111 ->114 0.10134
111 ->115 0.15615

Excited State 4: Singlet-A 4.1899 eV 295.92 nm f=0.2446 <S**2>=0.000
110 ->112 -0.13172
110 ->113 -0.14413
111 ->113 0.13963
111 ->114 0.10893
111 ->115 0.54398
111 ->116 -0.15917
111 ->117 0.18816
111 ->119 0.14275

Excited State 5: Singlet-A 4.3287 eV 286.42 nm f=0.0536 <S**2>=0.000
110 ->112 0.11754
110 ->113 -0.14380
111 ->112 0.17701
111 ->114 0.47972
111 ->117 -0.35624
111 ->121 0.10282

Excited State 6: Singlet-A 4.4317 eV 279.77 nm f=0.1739 <S**2>=0.000
111 ->114 -0.22175

111 ->115 0.10453
111 ->117 -0.44482
111 ->118 -0.28677
111 ->119 0.33028

(BMK/6-311+G* PCM Toluene, TD=(Nstates=6,root=1))

Excited State 1: Singlet-A 2.5663 eV 483.12 nm f=0.0951 <S**2>=0.000
111 ->112 0.68351
111 ->114 -0.15097
This state for optimization and/or second-order correction.
Total Energy, E(TD-HF/TD-DFT) = -1319.36942249
Copying the excited state density for this state as the 1-particle RhoCI density.

Excited State 2: Singlet-A 3.3416 eV 371.03 nm f=0.0013 <S**2>=0.000
111 ->113 0.69317

Excited State 3: Singlet-A 3.7795 eV 328.05 nm f=0.6869 <S**2>=0.000
110 ->112 -0.64791
111 ->114 -0.10449
111 ->115 -0.18191

Excited State 4: Singlet-A 3.9646 eV 312.73 nm f=0.1335 <S**2>=0.000
110 ->112 -0.13809
111 ->112 0.13602
111 ->114 0.62228
111 ->115 0.16216

Excited State 5: Singlet-A 4.0367 eV 307.14 nm f=0.2173 <S**2>=0.000
110 ->112 -0.17762
111 ->114 -0.13022
111 ->115 0.56291
111 ->116 0.29796
111 ->117 0.11234

Excited State 6: Singlet-A 4.1571 eV 298.24 nm f=0.0198 <S**2>=0.000
104 ->113 0.14753
110 ->112 -0.10322
110 ->113 0.63926

7.3 Quantum chemical calculation data of 6-(2′-(diphenylamino)-3-methyl-[1,1′-biphenyl]-4-yl)picolinonitrile (6c)

7.3.1 Computed xyz-coordinates (S_0 geometry) of compound 6c

(CAM-B3LYP/6-311+G* PCM Toluene)

N -2.641684 -0.027516 0.258385
C -3.467375 -0.847715 -0.541902
C -2.024885 -0.523727 1.432157
C -2.630801 1.378801 0.017933
C -1.427371 2.073961 -0.173917
C -1.485551 3.458969 -0.347159
C -2.691562 4.140924 -0.363431
C -3.878274 3.439736 -0.194449
C -3.841125 2.069903 0.003915
C -4.175643 -1.923304 0.000182
C -4.980412 -2.713509 -0.806928
C -5.110952 -2.443488 -2.163084
C -4.420521 -1.366400 -2.703571
C -3.602827 -0.579315 -1.907090

C	-1.414421	-1.779884	1.435838
C	-0.805590	-2.260767	2.583266
C	-0.774977	-1.494541	3.743174
C	-1.368138	-0.240503	3.738885
C	-1.994776	0.241326	2.597392
C	-0.101612	1.403289	-0.209890
C	0.953619	1.887498	0.560456
C	2.199133	1.292424	0.487275
C	2.430012	0.193067	-0.338832
C	1.384779	-0.298313	-1.135407
C	0.141442	0.322328	-1.051469
C	3.794208	-0.398100	-0.330478
C	4.004789	-1.777270	-0.221716
C	5.294757	-2.270785	-0.182146
C	6.358771	-1.383400	-0.246812
C	6.054667	-0.032309	-0.335702
N	4.816041	0.456770	-0.375393
C	1.549871	-1.436583	-2.111462
C	7.124720	0.935406	-0.390277
N	7.995011	1.682704	-0.431594
H	-0.560682	4.002368	-0.505015
H	-2.704556	5.214060	-0.516234
H	-4.830040	3.958833	-0.203252
H	-4.759260	1.514711	0.158011
H	-4.098689	-2.138787	1.058333
H	-5.522330	-3.541865	-0.363464
H	-5.745442	-3.060529	-2.788662
H	-4.510841	-1.136945	-3.759769
H	-3.073748	0.259475	-2.343435
H	-1.418970	-2.377855	0.532624
H	-0.338731	-3.239755	2.566939
H	-0.291335	-1.870848	4.637264
H	-1.354980	0.370684	4.634722
H	-2.462379	1.218431	2.609806
H	0.796659	2.726794	1.228451
H	3.016388	1.676833	1.085551
H	-0.660428	-0.044525	-1.682186
H	3.160136	-2.448474	-0.146825
H	5.474154	-3.335641	-0.092730
H	7.387215	-1.719304	-0.221387
H	1.373236	-2.407261	-1.639772
H	0.828929	-1.344747	-2.924401
H	2.546383	-1.465677	-2.552852

SCF Done: E(RCAM-B3LYP) = -1358.93693509   A.U. after   2 cycles
Zero-point correction=   0.458356 (Hartree/Particle)
Thermal correction to Energy=   0.485625
Thermal correction to Enthalpy=   0.486569
Thermal correction to Gibbs Free Energy=   0.397644
Sum of electronic and zero-point Energies=   -1358.478579
Sum of electronic and thermal Energies=   -1358.451310
Sum of electronic and thermal Enthalpies=   -1358.450366
Sum of electronic and thermal Free Energies=   -1358.539291

(BMK/6-311+G* PCM Toluene)

N	-2.683425	-0.014009	0.290821
C	-3.460133	-0.876931	-0.509597
C	-2.034724	-0.465380	1.463097
C	-2.673358	1.380133	-0.006306
C	-1.461489	2.073738	-0.217160

C	-1.519968	3.460869	-0.439296
C	-2.734522	4.142970	-0.488141
C	-3.929213	3.441031	-0.304919
C	-3.892739	2.070896	-0.056669
C	-4.135872	-1.980753	0.042648
C	-4.899456	-2.819228	-0.768738
C	-5.022032	-2.573416	-2.139565
C	-4.365426	-1.469672	-2.689472
C	-3.588158	-0.631680	-1.889510
C	-1.404628	-1.721489	1.496622
C	-0.761641	-2.156528	2.653723
C	-0.713404	-1.344656	3.791948
C	-1.322759	-0.089242	3.755395
C	-1.983705	0.348275	2.605871
C	-0.130884	1.396417	-0.230929
C	0.945673	1.936440	0.487334
C	2.198598	1.337279	0.425231
C	2.415714	0.179207	-0.337500
C	1.347605	-0.370955	-1.080646
C	0.095935	0.253040	-1.007584
C	3.791150	-0.405949	-0.321797
C	4.015251	-1.788562	-0.166539
C	5.317920	-2.271808	-0.123788
C	6.378522	-1.371640	-0.230075
C	6.051899	-0.019735	-0.359568
N	4.807157	0.456587	-0.402842
C	1.496714	-1.571025	-2.000652
C	7.116834	0.961786	-0.455535
N	7.981816	1.715106	-0.528603
H	-0.592495	4.000177	-0.612095
H	-2.748374	5.212464	-0.678164
H	-4.883619	3.958994	-0.340128
H	-4.812111	1.516313	0.109780
H	-4.064187	-2.176744	1.107371
H	-5.414626	-3.664081	-0.318931
H	-5.622512	-3.226698	-2.765682
H	-4.449985	-1.258141	-3.752228
H	-3.089258	0.225888	-2.330800
H	-1.425382	-2.352266	0.613397
H	-0.283076	-3.132440	2.660794
H	-0.205453	-1.684944	4.689618
H	-1.295608	0.555979	4.629557
H	-2.460792	1.323420	2.591680
H	0.799221	2.819435	1.103187
H	3.030793	1.761437	0.978778
H	-0.720542	-0.156447	-1.596979
H	3.175841	-2.465250	-0.056901
H	5.509117	-3.333678	0.000504
H	7.414328	-1.693041	-0.205218
H	1.342209	-2.515338	-1.465817
H	0.747785	-1.527398	-2.795880
H	2.483667	-1.612010	-2.468761

SCF Done: E(RBMK) = -1358.76490779 A.U. after 1 cycles
Zero-point correction= 0.456298 (Hartree/Particle)
Thermal correction to Energy= 0.483376
Thermal correction to Enthalpy= 0.484320
Thermal correction to Gibbs Free Energy= 0.396245
Sum of electronic and zero-point Energies= -1358.308610
Sum of electronic and thermal Energies= -1358.281532
Sum of electronic and thermal Enthalpies= -1358.280587

Sum of electronic and thermal Free Energies= -1358.368663

7.3.2 Computed excitations energies of compound 6c

(CAM-B3LYP/6-311+G* PCM Toluene, TD=(Nstates=25,root=1))

Excited State 1: Singlet-A 4.0616 eV 305.26 nm f=0.1868 <S**2>=0.000
115 ->117 0.52793
115 ->118 -0.39222
115 ->119 -0.17834
This state for optimization and/or second-order correction.
Total Energy, E(TD-HF/TD-DFT) = -1358.78767583
Copying the excited state density for this state as the 1-particle RhoCI density.

Excited State 2: Singlet-A 4.3419 eV 285.55 nm f=0.0646 <S**2>=0.000
115 ->118 -0.34634
115 ->119 0.55243
115 ->124 -0.10259

Excited State 3: Singlet-A 4.4743 eV 277.10 nm f=0.3353 <S**2>=0.000
115 ->120 0.61758
115 ->121 -0.23117
115 ->123 -0.12192

Excited State 4: Singlet-A 4.6829 eV 264.76 nm f=0.3356 <S**2>=0.000
108 ->116 -0.16798
108 ->117 0.12521
114 ->116 -0.31824
114 ->117 0.36572
115 ->116 0.33661
115 ->117 -0.10553
115 ->118 -0.10060
115 ->119 -0.16444
115 ->123 0.10958

Excited State 5: Singlet-A 4.7383 eV 261.66 nm f=0.1332 <S**2>=0.000
114 ->116 0.15990
115 ->116 -0.30642
115 ->117 -0.21483
115 ->118 -0.17238
115 ->119 -0.24394
115 ->122 0.14319
115 ->123 0.30113
115 ->124 -0.25404

Excited State 6: Singlet-A 4.8001 eV 258.30 nm f=0.4206 <S**2>=0.000
113 ->117 0.12091
114 ->117 0.48290
115 ->116 -0.32699
115 ->117 0.14087
115 ->123 -0.16495
115 ->124 0.13289

Excited State 7: Singlet-A 4.9105 eV 252.49 nm f=0.0293 <S**2>=0.000
106 ->116 -0.21743
111 ->117 -0.11190
113 ->117 0.31886
114 ->116 0.13411
114 ->122 -0.10233

115 ->116 0.24127
115 ->122 0.15687
115 ->124 -0.15663
115 ->126 0.11580
115 ->127 0.22161

Excited State 8: Singlet-A 4.9167 eV 252.17 nm f=0.0028 <S**2>=0.000
106 ->116 0.21443
108 ->116 -0.10618
110 ->120 -0.10900
114 ->116 -0.23286
115 ->116 -0.25650
115 ->122 0.13127
115 ->123 -0.16618
115 ->124 -0.14538
115 ->126 0.10695
115 ->127 0.36169

Excited State 9: Singlet-A 4.9514 eV 250.40 nm f=0.0024 <S**2>=0.000
106 ->116 0.52990
106 ->117 -0.14549
107 ->116 0.16531
113 ->116 0.15120
113 ->117 0.19120
114 ->116 0.13710

Excited State 10: Singlet-A 4.9695 eV 249.49 nm f=0.1460 <S**2>=0.000
108 ->116 0.16139
112 ->117 0.10866
113 ->117 -0.27012
114 ->116 0.34840
114 ->117 0.16072
114 ->123 -0.10311
115 ->116 0.21662
115 ->127 0.20023

Excited State 11: Singlet-A 5.0952 eV 243.33 nm f=0.0430 <S**2>=0.000
109 ->118 -0.10784
113 ->117 -0.13146
114 ->117 0.13084
114 ->119 0.12457
115 ->117 0.31826
115 ->118 0.27409
115 ->119 0.15738
115 ->123 0.19550
115 ->124 -0.16017
115 ->130 -0.23652
115 ->132 0.11110

Excited State 12: Singlet-A 5.2962 eV 234.10 nm f=0.0047 <S**2>=0.000
115 ->120 0.13548
115 ->121 0.43386
115 ->122 0.40947
115 ->124 0.23523
115 ->136 -0.10281

Excited State 13: Singlet-A 5.4125 eV 229.07 nm f=0.0154 <S**2>=0.000
106 ->117 -0.15655
113 ->117 -0.13397
114 ->119 -0.11131
115 ->118 0.15684

```
    115 ->120       -0.16867
    115 ->121       -0.13406
    115 ->122        0.31328
    115 ->123       -0.22043
    115 ->124       -0.19291
    115 ->125       -0.13152
    115 ->127       -0.23883

 Excited State  14:      Singlet-A      5.4461 eV  227.66 nm  f=0.0087  <S**2>=0.000
    106 ->116        0.17820
    106 ->117        0.55222
    106 ->118        0.19270
    107 ->117        0.13334

 Excited State  15:      Singlet-A      5.5544 eV  223.22 nm  f=0.0734  <S**2>=0.000
    108 ->117        0.16810
    108 ->119       -0.11257
    109 ->117        0.11824
    109 ->118       -0.11087
    111 ->117        0.13025
    112 ->117       -0.10132
    114 ->118        0.18943
    114 ->119        0.34903
    115 ->118       -0.10819
    115 ->122        0.14516
    115 ->123       -0.26343

 Excited State  16:      Singlet-A      5.6319 eV  220.15 nm  f=0.0312  <S**2>=0.000
    105 ->116        0.12220
    107 ->116       -0.10542
    107 ->117        0.10276
    108 ->117        0.15819
    111 ->116       -0.12514
    112 ->116       -0.12280
    113 ->116        0.45545
    114 ->116        0.11423
    114 ->118        0.18867
    114 ->123        0.11262

 Excited State  17:      Singlet-A      5.6497 eV  219.45 nm  f=0.0342  <S**2>=0.000
    107 ->116        0.12577
    108 ->117        0.15618
    111 ->116        0.11135
    113 ->116       -0.34084
    113 ->117        0.12804
    114 ->118        0.33639
    114 ->123        0.10721
    115 ->124       -0.11123

 Excited State  18:      Singlet-A      5.7073 eV  217.24 nm  f=0.0054  <S**2>=0.000
    113 ->117       -0.10153
    114 ->118       -0.16127
    115 ->118       -0.10942
    115 ->121        0.15932
    115 ->123       -0.24712
    115 ->124       -0.11164
    115 ->125        0.20522
    115 ->126        0.11495
    115 ->128        0.26041
    115 ->129        0.16714
    115 ->131       -0.10989
```

115 ->132 0.13731
115 ->134 0.11681
115 ->137 -0.11147

Excited State 19: Singlet-A 5.7446 eV 215.83 nm f=0.0011 <S**2>=0.000
115 ->121 0.14716
115 ->122 -0.16883
115 ->125 -0.26166
115 ->126 0.51509

Excited State 20: Singlet-A 5.7919 eV 214.06 nm f=0.0225 <S**2>=0.000
105 ->116 0.18573
106 ->116 0.14765
107 ->116 -0.25444
107 ->117 0.22731
108 ->116 -0.22629
113 ->116 -0.23476
113 ->117 -0.10406
114 ->116 0.27609
114 ->118 -0.16638
115 ->125 0.10262

Excited State 21: Singlet-A 5.8516 eV 211.88 nm f=0.0684 <S**2>=0.000
115 ->124 -0.12562
115 ->125 0.39076
115 ->126 0.12814
115 ->127 -0.11142
115 ->128 -0.19218
115 ->130 0.31013
115 ->137 0.24459

Excited State 22: Singlet-A 5.9221 eV 209.36 nm f=0.0109 <S**2>=0.000
112 ->120 -0.15782
113 ->117 0.19708
113 ->120 -0.13402
114 ->120 -0.10998
114 ->123 -0.13632
115 ->121 0.15280
115 ->125 -0.11086
115 ->126 -0.13938
115 ->127 0.11539
115 ->128 0.24544
115 ->130 0.15904
115 ->131 -0.12167
115 ->132 -0.16819
115 ->137 0.12808

Excited State 23: Singlet-A 5.9435 eV 208.60 nm f=0.1094 <S**2>=0.000
107 ->116 0.20684
108 ->116 0.13176
111 ->118 0.13111
111 ->120 0.13388
113 ->117 -0.14779
113 ->120 0.15852
114 ->122 -0.11184
115 ->121 0.10892
115 ->125 -0.12199
115 ->128 0.27568
115 ->130 0.10158
115 ->132 -0.13314
115 ->137 0.15405

```
Excited State  24:      Singlet-A      5.9625 eV  207.94 nm  f=0.2341  <S**2>=0.000
    105 ->116        0.10323
    107 ->116        0.22751
    108 ->116        0.14657
    111 ->117        0.12366
    112 ->117        0.12173
    112 ->120       -0.19456
    113 ->117       -0.11469
    113 ->118       -0.10590
    113 ->120       -0.14331
    114 ->118       -0.16776
    114 ->120        0.17403
    114 ->123        0.12336
    115 ->124        0.14452
    115 ->127        0.15175

Excited State  25:      Singlet-A      6.0321 eV  205.54 nm  f=0.0252  <S**2>=0.000
    107 ->117       -0.14571
    111 ->117        0.25258
    111 ->118       -0.22026
    112 ->117       -0.14465
    112 ->118        0.15523
    114 ->120        0.12760
    114 ->122       -0.13004
    114 ->123        0.18030
    115 ->127        0.21094
    115 ->130        0.11373
```

(BMK/6-311+G* PCM Toluene, TD=(Nstates=25,root=1))

```
Excited State   1:      Singlet-A      3.7250 eV  332.84 nm  f=0.1191  <S**2>=0.000
    115 ->116        0.15012
    115 ->117        0.60991
    115 ->118       -0.27878
    115 ->119       -0.12811
 This state for optimization and/or second-order correction.
 Total Energy, E(TD-HF/TD-DFT) =  -1358.62801583
 Copying the excited state density for this state as the 1-particle RhoCI density.

Excited State   2:      Singlet-A      4.1133 eV  301.42 nm  f=0.0040  <S**2>=0.000
    115 ->116        0.68388
    115 ->117       -0.15160

Excited State   3:      Singlet-A      4.1887 eV  296.00 nm  f=0.0533  <S**2>=0.000
    115 ->118       -0.36261
    115 ->119        0.57418

Excited State   4:      Singlet-A      4.3590 eV  284.43 nm  f=0.3504  <S**2>=0.000
    115 ->120        0.66905
    115 ->121        0.14407

Excited State   5:      Singlet-A      4.4516 eV  278.52 nm  f=0.1001  <S**2>=0.000
    115 ->117        0.26880
    115 ->118        0.43298
    115 ->119        0.34993
    115 ->121       -0.17608
    115 ->122       -0.23949

Excited State   6:      Singlet-A      4.5831 eV  270.53 nm  f=0.7292  <S**2>=0.000
    108 ->117        0.11718
```

```
     114 ->116        -0.16709
     114 ->117         0.64021

 Excited State   7:      Singlet-A      4.6648 eV  265.79 nm  f=0.1924  <S**2>=0.000
     108 ->116         0.15047
     114 ->116         0.62492
     114 ->117         0.19231

 Excited State   8:      Singlet-A      4.7823 eV  259.26 nm  f=0.0465  <S**2>=0.000
     113 ->117        -0.18711
     115 ->121        -0.26690
     115 ->122         0.46095
     115 ->123         0.28783

 Excited State   9:      Singlet-A      4.8082 eV  257.86 nm  f=0.0215  <S**2>=0.000
     113 ->117         0.12596
     114 ->119        -0.11623
     115 ->117         0.12705
     115 ->118         0.21202
     115 ->121         0.39694
     115 ->122         0.34043
     115 ->123        -0.16178
     115 ->125        -0.12216
     115 ->126         0.10063

 Excited State  10:      Singlet-A      4.8254 eV  256.94 nm  f=0.0238  <S**2>=0.000
     106 ->116         0.48700
     106 ->117        -0.26984
     107 ->116         0.12714
     113 ->116         0.13478
     113 ->117        -0.20795
     114 ->116         0.10201
     115 ->121         0.12121
     115 ->123        -0.14892

 Excited State  11:      Singlet-A      4.8593 eV  255.15 nm  f=0.0334  <S**2>=0.000
     106 ->116         0.25787
     111 ->117        -0.11694
     112 ->117        -0.13490
     113 ->116         0.18620
     113 ->117         0.42234
     114 ->120        -0.11974
     114 ->121         0.17837
     115 ->123         0.21193

 Excited State  12:      Singlet-A      5.1412 eV  241.16 nm  f=0.0109  <S**2>=0.000
     113 ->117        -0.13326
     115 ->120        -0.12306
     115 ->121         0.38685
     115 ->122        -0.18790
     115 ->123         0.46983

 Excited State  13:      Singlet-A      5.2635 eV  235.55 nm  f=0.0021  <S**2>=0.000
     106 ->117         0.24433
     110 ->116         0.10662
     111 ->116        -0.15063
     112 ->116        -0.13647
     113 ->116         0.57511
     113 ->117        -0.14550

 Excited State  14:      Singlet-A      5.3565 eV  231.46 nm  f=0.0062  <S**2>=0.000
```

106 ->116 0.33163
106 ->117 0.44626
106 ->118 0.17194
107 ->117 0.10774
113 ->116 -0.17248
114 ->119 -0.16474

Excited State 15: Singlet-A 5.3807 eV 230.42 nm f=0.0269 <S**2>=0.000
106 ->116 0.13127
106 ->117 0.19092
108 ->119 -0.11788
109 ->117 0.17054
109 ->118 -0.15111
111 ->117 0.12135
112 ->117 -0.19251
114 ->118 0.10663
114 ->119 0.38870
115 ->118 0.11167
115 ->121 0.17408

Excited State 16: Singlet-A 5.4721 eV 226.57 nm f=0.0416 <S**2>=0.000
105 ->116 0.10321
108 ->117 0.22012
111 ->117 0.12669
113 ->117 0.18640
114 ->118 0.53879
114 ->122 0.13469

Excited State 17: Singlet-A 5.5547 eV 223.20 nm f=0.0027 <S**2>=0.000
115 ->124 0.49469
115 ->125 0.38550
115 ->126 0.24382

Excited State 18: Singlet-A 5.5828 eV 222.08 nm f=0.0484 <S**2>=0.000
114 ->119 0.19060
115 ->124 0.11228
115 ->125 -0.36893
115 ->126 0.41534
115 ->127 -0.13017
115 ->129 -0.10378
115 ->130 0.10646
115 ->131 -0.17449

Excited State 19: Singlet-A 5.6162 eV 220.76 nm f=0.0398 <S**2>=0.000
105 ->116 -0.21446
107 ->116 0.19558
107 ->117 -0.25913
108 ->116 0.33793
108 ->117 -0.23208
111 ->116 0.13412
113 ->117 0.11583
114 ->116 -0.18985
114 ->118 0.11969

Excited State 20: Singlet-A 5.6505 eV 219.42 nm f=0.0306 <S**2>=0.000
110 ->117 -0.13868
111 ->116 0.14069
111 ->117 0.50416
111 ->118 -0.15429
113 ->117 0.21550
114 ->118 -0.24024

```
Excited State  21:      Singlet-A      5.7278 eV  216.46 nm  f=0.0144  <S**2>=0.000
    110 ->117        -0.14903
    111 ->117        -0.19024
    111 ->118         0.10568
    112 ->116         0.12693
    112 ->117         0.41797
    112 ->118        -0.14783
    113 ->117         0.17268
    114 ->119         0.21777
    114 ->120         0.12004
    114 ->121        -0.14922
    115 ->126        -0.10615

Excited State  22:      Singlet-A      5.8112 eV  213.35 nm  f=0.1698  <S**2>=0.000
    107 ->116        -0.12322
    108 ->116        -0.14615
    112 ->117        -0.22591
    113 ->117         0.17413
    113 ->118         0.19317
    114 ->119        -0.13447
    114 ->120         0.33230
    114 ->121        -0.28800
    115 ->131         0.11798

Excited State  23:      Singlet-A      5.8649 eV  211.40 nm  f=0.0127  <S**2>=0.000
    108 ->117         0.17510
    110 ->116        -0.20049
    111 ->116         0.47743
    111 ->117        -0.16488
    112 ->116         0.22092
    113 ->116         0.21816

Excited State  24:      Singlet-A      5.8730 eV  211.11 nm  f=0.0212  <S**2>=0.000
    107 ->116        -0.10988
    108 ->116        -0.16795
    108 ->117        -0.14667
    110 ->117         0.13654
    110 ->120        -0.11796
    111 ->116         0.14482
    111 ->118        -0.11919
    111 ->119         0.13828
    111 ->120         0.12619
    112 ->116         0.12372
    112 ->120         0.11773
    113 ->119         0.12959
    113 ->120         0.26845
    114 ->118         0.10872
    114 ->119         0.10561
    115 ->123        -0.16231
    115 ->129         0.10213
    115 ->130        -0.10032

Excited State  25:      Singlet-A      5.8963 eV  210.27 nm  f=0.1072  <S**2>=0.000
    105 ->116        -0.10662
    107 ->116        -0.22159
    108 ->116        -0.27623
    108 ->117        -0.33953
    111 ->116         0.11731
    113 ->118        -0.13498
    113 ->120        -0.16038
```

114 ->118 0.17596

7.3.3 Computed xyz-coordinates (S_1 geometry) of compound 6c (CAM-B3LYP/6-311+G* PCM Toluene)

N	-2.650975	0.017864	0.226543
C	-3.494556	-0.878970	-0.466076
C	-2.008942	-0.378779	1.415295
C	-2.616755	1.364119	-0.165979
C	-1.360614	2.092648	-0.194296
C	-1.488874	3.507157	-0.267477
C	-2.695700	4.125852	-0.458130
C	-3.892085	3.376740	-0.570307
C	-3.837368	2.013848	-0.410375
C	-4.292174	-1.794083	0.228671
C	-5.124327	-2.650292	-0.472526
C	-5.173908	-2.605085	-1.860829
C	-4.388924	-1.686139	-2.550616
C	-3.557589	-0.821494	-1.862126
C	-1.526663	-1.685004	1.565175
C	-0.881859	-2.050243	2.728994
C	-0.693507	-1.119913	3.751997
C	-1.164435	0.176021	3.601521
C	-1.823366	0.553597	2.441505
C	-0.085921	1.455514	-0.287911
C	1.111155	2.098348	0.138587
C	2.313783	1.457706	0.079459
C	2.453912	0.129568	-0.406308
C	1.289887	-0.498698	-0.923084
C	0.080735	0.163835	-0.860456
C	3.786730	-0.463332	-0.375535
C	4.026079	-1.851054	-0.257103
C	5.314504	-2.334660	-0.213297
C	6.384354	-1.445511	-0.271126
C	6.064224	-0.096764	-0.347574
N	4.830816	0.390949	-0.398373
C	1.306435	-1.829707	-1.635872
C	7.131748	0.878160	-0.375145
N	7.998728	1.630878	-0.394095
H	-0.589126	4.109436	-0.264964
H	-2.730090	5.205326	-0.556233
H	-4.841232	3.872587	-0.730989
H	-4.750163	1.428892	-0.400578
H	-4.273012	-1.813904	1.310555
H	-5.749514	-3.348907	0.071099
H	-5.827789	-3.277346	-2.403685
H	-4.421615	-1.645869	-3.632943
H	-2.948529	-0.103145	-2.396101
H	-1.634122	-2.393679	0.754035
H	-0.504020	-3.060097	2.836949
H	-0.178555	-1.409849	4.660321
H	-1.026976	0.900696	4.395351
H	-2.221573	1.553760	2.334784
H	1.068986	3.088748	0.576139
H	3.202687	1.959312	0.438908
H	-0.766856	-0.303233	-1.345319
H	3.199128	-2.537576	-0.161138
H	5.493962	-3.399378	-0.116376
H	7.414168	-1.775483	-0.239830

H 1.245036 -2.673663 -0.942664
H 0.447159 -1.906710 -2.303398
H 2.206187 -1.968052 -2.236782

SCF Done: E(RCAM-B3LYP) = -1358.92021679 A.U. after 2 cycles
Zero-point correction= 0.456049 (Hartree/Particle)
Thermal correction to Energy= 0.483257
Thermal correction to Enthalpy= 0.484201
Thermal correction to Gibbs Free Energy= 0.396805
Sum of electronic and zero-point Energies= -1358.348437
Sum of electronic and thermal Energies= -1358.321230
Sum of electronic and thermal Enthalpies= -1358.320285
Sum of electronic and thermal Free Energies= -1358.407681

7.3.4 Computed emission energies of compound 6c (CAM-B3LYP/6-311+G* PCM Toluene, TD=(Nstates=6,root=1))

Excited State 1: Singlet-A 3.0353 eV 408.48 nm f=0.2179 <S**2>=0.000
115 ->116 0.64564
115 ->118 -0.24846
This state for optimization and/or second-order correction.
Total Energy, E(TD-HF/TD-DFT) = -1358.80877133
Copying the excited state density for this state as the 1-particle RhoCI density.

Excited State 2: Singlet-A 3.9848 eV 311.14 nm f=0.4540 <S**2>=0.000
114 ->116 0.59478
115 ->117 0.10508
115 ->119 0.25307

Excited State 3: Singlet-A 4.1590 eV 298.11 nm f=0.0058 <S**2>=0.000
108 ->117 -0.11700
114 ->117 0.28944
115 ->117 -0.55098
115 ->119 0.23657

Excited State 4: Singlet-A 4.1967 eV 295.43 nm f=0.4333 <S**2>=0.000
114 ->116 -0.27804
115 ->117 0.22330
115 ->118 0.16927
115 ->119 0.53457

Excited State 5: Singlet-A 4.3613 eV 284.28 nm f=0.0800 <S**2>=0.000
114 ->116 -0.13368
115 ->116 -0.16508
115 ->118 -0.40006
115 ->121 0.46114
115 ->125 0.11657

Excited State 6: Singlet-A 4.4193 eV 280.55 nm f=0.1367 <S**2>=0.000
115 ->118 0.26326
115 ->121 0.42621
115 ->122 -0.23328
115 ->123 -0.32656

7.4 Quantum chemical calculation data of 2′-(diphenylamino)-[1,1′-biphenyl]-2-carbonitrile (7a)

7.4.1 Computed xyz-coordinates (S_0 geometry) of compound 7a

(CAM-B3LYP/6-311+G* PCM Toluene)

N	0.795145	-0.208431	-0.286923
C	2.128606	-0.325266	0.191714
C	0.060148	-1.396182	-0.559972
C	0.390828	0.994765	-0.927578
C	-0.903558	1.505367	-0.738754
C	-1.286164	2.652647	-1.431308
C	-0.405253	3.318221	-2.269674
C	0.881909	2.825085	-2.432024
C	1.269905	1.669230	-1.775043
C	2.904652	-1.447838	-0.097146
C	4.202526	-1.546492	0.386662
C	4.751742	-0.525476	1.149122
C	3.982142	0.597581	1.430513
C	2.681002	0.697599	0.964589
C	-0.153625	-2.323918	0.458868
C	-0.863518	-3.486461	0.207550
C	-1.381357	-3.735847	-1.059271
C	-1.172567	-2.812527	-2.073738
C	-0.449762	-1.651531	-1.830202
C	-1.889048	0.868825	0.180743
C	-2.977814	0.167908	-0.327610
C	-3.918590	-0.408272	0.513417
C	-3.791221	-0.292251	1.891185
C	-2.726384	0.414050	2.421820
C	-1.782921	0.995798	1.572676
C	-0.726816	1.767315	2.157438
N	0.094750	2.397426	2.656178
H	-2.288190	3.040477	-1.283757
H	-0.720113	4.218303	-2.784707
H	1.584317	3.332018	-3.084127
H	2.267914	1.273188	-1.918295
H	2.493312	-2.247489	-0.700443
H	4.789788	-2.427946	0.152951
H	5.766274	-0.603689	1.522875
H	4.391160	1.400768	2.033572
H	2.083197	1.565769	1.210590
H	0.239921	-2.123104	1.448528
H	-1.024779	-4.197756	1.010086
H	-1.941828	-4.643597	-1.251824
H	-1.564225	-2.998479	-3.067924
H	-0.281371	-0.938404	-2.628858
H	-3.076740	0.061410	-1.401083
H	-4.753406	-0.954490	0.089510
H	-4.522506	-0.744193	2.550615
H	-2.621164	0.530263	3.493649

SCF Done: E(RCAM-B3LYP) = -1072.62369990 A.U. after 2 cycles
Zero-point correction= 0.360903 (Hartree/Particle)
Thermal correction to Energy= 0.381986
Thermal correction to Enthalpy= 0.382930
Thermal correction to Gibbs Free Energy= 0.309260
Sum of electronic and zero-point Energies= -1072.262797
Sum of electronic and thermal Energies= -1072.241714

Sum of electronic and thermal Enthalpies= -1072.240770
Sum of electronic and thermal Free Energies= -1072.314440

(BMK/6-311+G* PCM Toluene)

N	0.803109	-0.168647	-0.358899
C	2.125461	-0.448712	0.068532
C	-0.055409	-1.245947	-0.716974
C	0.458432	1.133999	-0.804766
C	-0.818940	1.672700	-0.522499
C	-1.161277	2.928557	-1.042816
C	-0.253516	3.673185	-1.795801
C	1.017329	3.150424	-2.046908
C	1.364202	1.889707	-1.566100
C	2.773264	-1.636601	-0.309596
C	4.072618	-1.901254	0.129012
C	4.753333	-0.986089	0.934094
C	4.111831	0.200649	1.304363
C	2.810338	0.468458	0.884787
C	-0.353450	-2.241171	0.225300
C	-1.212170	-3.286662	-0.108410
C	-1.790712	-3.349925	-1.381596
C	-1.491342	-2.361435	-2.321151
C	-0.622937	-1.316607	-1.995569
C	-1.821218	0.939802	0.313027
C	-2.995577	0.440948	-0.262181
C	-3.935406	-0.253468	0.500990
C	-3.722198	-0.459382	1.866558
C	-2.572096	0.044058	2.467764
C	-1.628616	0.741436	1.695810
C	-0.473179	1.284326	2.360350
N	0.423242	1.726810	2.930708
H	-2.146261	3.332838	-0.823838
H	-0.533001	4.652048	-2.174172
H	1.736482	3.715734	-2.633382
H	2.343460	1.473905	-1.782539
H	2.261632	-2.352369	-0.944620
H	4.555176	-2.826836	-0.174117
H	5.764997	-1.193663	1.270555
H	4.620430	0.920313	1.940364
H	2.313834	1.379491	1.201184
H	0.087497	-2.180746	1.216453
H	-1.440977	-4.048129	0.632361
H	-2.465038	-4.162587	-1.636520
H	-1.926901	-2.404320	-3.316011
H	-0.390254	-0.548623	-2.727935
H	-3.156903	0.580485	-1.327143
H	-4.833451	-0.638993	0.026560
H	-4.450252	-1.002364	2.461687
H	-2.397517	-0.089300	3.531128

SCF Done: E(RBMK) = -1072.48771468 A.U. after 1 cycles
Zero-point correction= 0.358559 (Hartree/Particle)
Thermal correction to Energy= 0.379504
Thermal correction to Enthalpy= 0.380448
Thermal correction to Gibbs Free Energy= 0.308277
Sum of electronic and zero-point Energies= -1072.129156
Sum of electronic and thermal Energies= -1072.108211
Sum of electronic and thermal Enthalpies= -1072.107267
Sum of electronic and thermal Free Energies= -1072.179437

7.4.2 Computed excitations energies of compound 7a

(CAM-B3LYP/6-311+G* PCM Toluene, TD=(Nstates=25,root=1))

```
Excited State   1:      Singlet-A      4.0818 eV  303.75 nm  f=0.0295  <S**2>=0.000
      91 -> 92         0.64943
      91 -> 93         0.16337
      91 -> 94        -0.16166
 This state for optimization and/or second-order correction.
 Total Energy, E(TD-HF/TD-DFT) =  -1072.47369511
 Copying the excited state density for this state as the 1-particle RhoCI density.

 Excited State   2:      Singlet-A      4.3961 eV  282.04 nm  f=0.1949  <S**2>=0.000
      91 -> 93         0.42886
      91 -> 94         0.44509
      91 -> 95         0.23111
      91 -> 96         0.11586
      91 -> 97        -0.12937

 Excited State   3:      Singlet-A      4.4828 eV  276.58 nm  f=0.1733  <S**2>=0.000
      91 -> 92        -0.11626
      91 -> 94        -0.32737
      91 -> 95         0.56947

 Excited State   4:      Singlet-A      4.5764 eV  270.92 nm  f=0.2221  <S**2>=0.000
      91 -> 92        -0.21367
      91 -> 93         0.37395
      91 -> 94        -0.31246
      91 -> 95        -0.26601
      91 -> 97        -0.31273

 Excited State   5:      Singlet-A      4.9263 eV  251.68 nm  f=0.0186  <S**2>=0.000
      87 -> 95        -0.10639
      91 -> 98         0.58086

 Excited State   6:      Singlet-A      5.0549 eV  245.27 nm  f=0.0173  <S**2>=0.000
      87 -> 95         0.10165
      88 -> 92         0.11554
      88 -> 94        -0.13612
      90 -> 97        -0.11341
      91 ->100         0.19152
      91 ->101         0.15775
      91 ->102         0.49365

 Excited State   7:      Singlet-A      5.0823 eV  243.95 nm  f=0.0304  <S**2>=0.000
      84 -> 92         0.23843
      85 -> 93         0.15002
      86 -> 92        -0.12861
      86 -> 93        -0.10239
      88 -> 92         0.23151
      89 -> 92         0.25412
      89 -> 93        -0.12990
      91 -> 93         0.19848
      91 -> 97         0.32702
      91 ->102        -0.15893

 Excited State   8:      Singlet-A      5.1855 eV  239.10 nm  f=0.0818  <S**2>=0.000
      84 -> 92        -0.16059
      85 -> 93        -0.11385
      88 -> 92        -0.17008
```

89 -> 92 -0.24753
91 -> 93 0.26407
91 -> 95 -0.11130
91 -> 96 -0.11383
91 -> 97 0.43173

Excited State 9: Singlet-A 5.4627 eV 226.96 nm f=0.0041 <S**2>=0.000
91 -> 94 -0.11425
91 -> 96 0.51230
91 -> 97 0.15085
91 -> 99 0.32622
91 ->100 -0.14462

Excited State 10: Singlet-A 5.5179 eV 224.70 nm f=0.1100 <S**2>=0.000
85 -> 92 -0.33451
86 -> 92 0.27578
87 -> 92 0.13809
88 -> 93 0.11335
89 -> 92 0.40468
89 -> 93 0.19851
90 -> 92 0.11594

Excited State 11: Singlet-A 5.7646 eV 215.08 nm f=0.0049 <S**2>=0.000
87 -> 92 -0.11203
87 -> 94 0.10589
88 -> 92 -0.10113
89 -> 92 0.12027
91 -> 96 -0.21136
91 -> 99 0.29959
91 ->101 0.10402
91 ->104 -0.28834
91 ->105 -0.16294
91 ->107 0.25045
91 ->108 0.16017

Excited State 12: Singlet-A 5.7763 eV 214.64 nm f=0.1187 <S**2>=0.000
86 -> 92 0.11663
86 -> 93 0.11814
86 -> 98 0.11555
88 -> 92 0.10529
88 -> 95 -0.12426
89 -> 93 0.27055
89 -> 94 0.11907
89 -> 95 0.22830
90 -> 92 -0.18655
90 -> 93 -0.14560
90 -> 97 0.14080
91 -> 94 0.15488
91 -> 98 -0.13599
91 ->100 -0.10402

Excited State 13: Singlet-A 5.8184 eV 213.09 nm f=0.1598 <S**2>=0.000
84 -> 92 -0.14961
85 -> 92 0.31365
87 -> 92 -0.18630
88 -> 92 -0.29204
89 -> 92 0.30382
89 -> 93 0.13038
90 -> 92 -0.10957
91 -> 99 -0.17843

```
Excited State  14:      Singlet-A      5.8501 eV  211.94 nm  f=0.0162  <S**2>=0.000
     87 -> 95         0.13113
     88 -> 94        -0.11610
     90 -> 95         0.16682
     90 -> 97         0.10575
     91 -> 96         0.20907
     91 -> 98         0.25159
     91 -> 99        -0.17654
     91 ->100         0.22185
     91 ->101         0.20778
     91 ->102        -0.19523

Excited State  15:      Singlet-A      5.9110 eV  209.75 nm  f=0.0383  <S**2>=0.000
     91 -> 99         0.26001
     91 ->100         0.52354
     91 ->102        -0.12972
     91 ->106         0.11711

Excited State  16:      Singlet-A      5.9282 eV  209.14 nm  f=0.0265  <S**2>=0.000
     84 -> 92         0.14558
     87 -> 95        -0.17727
     88 -> 95         0.17223
     89 -> 92        -0.11835
     89 -> 94         0.10937
     90 -> 95        -0.13179
     91 -> 98        -0.13666
     91 -> 99        -0.21696
     91 ->101         0.29104
     91 ->106        -0.10338
     91 ->107         0.12504

Excited State  17:      Singlet-A      6.0638 eV  204.46 nm  f=0.2567  <S**2>=0.000
     85 -> 92         0.14106
     86 -> 92        -0.26951
     87 -> 92         0.15595
     88 -> 95        -0.15413
     89 -> 93         0.15844
     90 -> 92         0.42831
     90 -> 94        -0.13885
     91 ->101         0.14181
     91 ->102        -0.11476

Excited State  18:      Singlet-A      6.0758 eV  204.06 nm  f=0.0615  <S**2>=0.000
     88 -> 92        -0.15246
     88 -> 94         0.14448
     90 -> 93        -0.26067
     90 -> 97         0.23506
     91 ->101         0.24214
     91 ->102         0.23196
     91 ->108        -0.17218

Excited State  19:      Singlet-A      6.0961 eV  203.38 nm  f=0.0394  <S**2>=0.000
     87 -> 92         0.11860
     88 -> 92        -0.17584
     88 -> 93        -0.11317
     90 -> 92         0.18128
     90 -> 93        -0.10195
     90 -> 94        -0.10471
     90 ->102        -0.10131
     91 -> 96         0.11822
     91 ->100         0.10598
```

91 ->101 -0.32345
91 ->102 0.18316
91 ->107 0.14157
91 ->108 0.26376

Excited State 20: Singlet-A 6.1543 eV 201.46 nm f=0.3142 <S**2>=0.000
85 -> 93 -0.14574
86 -> 92 -0.23439
87 -> 92 -0.11651
87 -> 94 0.11241
87 -> 95 -0.12754
88 -> 92 0.30165
88 -> 93 0.14209
89 -> 93 0.31664
89 -> 94 -0.10273
89 -> 98 0.10414
90 -> 97 0.11806
91 ->102 0.17362

Excited State 21: Singlet-A 6.2608 eV 198.03 nm f=0.1220 <S**2>=0.000
84 -> 92 -0.10543
85 -> 94 0.11995
86 -> 93 0.12747
87 -> 92 -0.12108
87 -> 98 0.15762
88 -> 94 -0.19229
88 -> 95 0.26195
89 -> 94 0.33428
90 -> 92 0.10850
91 ->103 0.10230

Excited State 22: Singlet-A 6.2791 eV 197.46 nm f=0.1347 <S**2>=0.000
87 -> 92 -0.16241
89 -> 94 0.15001
89 -> 95 -0.14071
89 -> 98 -0.13407
90 -> 94 -0.28910
91 ->103 -0.32960
91 ->104 -0.19602
91 ->109 0.10335
91 ->111 -0.10530

Excited State 23: Singlet-A 6.2905 eV 197.10 nm f=0.0969 <S**2>=0.000
86 -> 92 -0.15354
87 -> 92 0.10435
88 -> 92 -0.15045
90 -> 94 0.28736
90 -> 98 0.10251
90 ->102 0.10589
91 ->103 -0.38364
91 ->104 -0.15273

Excited State 24: Singlet-A 6.3278 eV 195.93 nm f=0.3078 <S**2>=0.000
84 -> 92 -0.20022
86 -> 93 0.20792
87 -> 92 0.17268
87 -> 93 0.17547
87 -> 94 -0.10996
88 -> 92 0.23658
89 -> 92 0.11895
89 -> 93 -0.15333

90 -> 93 -0.10164
90 -> 94 0.19283
90 -> 95 -0.11718
90 ->102 0.13651
91 ->108 0.15108

Excited State 25: Singlet-A 6.3812 eV 194.30 nm f=0.0041 <S**2>=0.000
88 -> 93 -0.18492
88 -> 95 0.15721
89 -> 95 0.11372
91 ->101 0.17077
91 ->105 -0.10046
91 ->106 0.41445
91 ->107 -0.20154

(BMK/6-311+G* PCM Toluene, TD=(Nstates=25,root=1))

Excited State 1: Singlet-A 3.6977 eV 335.30 nm f=0.0148 <S**2>=0.000
91 -> 92 0.68664
91 -> 93 0.12077
This state for optimization and/or second-order correction.
Total Energy, E(TD-HF/TD-DFT) = -1072.35182585
Copying the excited state density for this state as the 1-particle RhoCI density.

Excited State 2: Singlet-A 4.2042 eV 294.90 nm f=0.1855 <S**2>=0.000
91 -> 93 -0.34481
91 -> 94 0.59465

Excited State 3: Singlet-A 4.3020 eV 288.20 nm f=0.2342 <S**2>=0.000
91 -> 93 0.50111
91 -> 94 0.29534
91 -> 95 0.31579
91 -> 96 0.20234

Excited State 4: Singlet-A 4.3493 eV 285.07 nm f=0.1029 <S**2>=0.000
91 -> 92 0.10656
91 -> 93 -0.23801
91 -> 94 -0.18109
91 -> 95 0.60936

Excited State 5: Singlet-A 4.7365 eV 261.76 nm f=0.0158 <S**2>=0.000
91 -> 93 -0.15162
91 -> 96 0.53032
91 -> 97 -0.38221

Excited State 6: Singlet-A 4.8733 eV 254.42 nm f=0.1062 <S**2>=0.000
89 -> 92 0.10447
91 -> 93 -0.14869
91 -> 96 0.35609
91 -> 97 0.51245
91 -> 98 -0.12784

Excited State 7: Singlet-A 4.9928 eV 248.33 nm f=0.0286 <S**2>=0.000
88 -> 94 0.13096
90 -> 96 0.11845
91 -> 96 0.12630
91 -> 98 0.61102

Excited State 8: Singlet-A 5.0390 eV 246.05 nm f=0.1018 <S**2>=0.000
84 -> 92 0.16428
85 -> 92 -0.13371

85 -> 93 -0.16099
87 -> 92 0.18908
89 -> 92 0.52026
89 -> 93 -0.10900
90 -> 92 -0.17073
90 -> 93 0.10112
91 -> 97 -0.11902

Excited State 9: Singlet-A 5.3556 eV 231.50 nm f=0.0552 <S**2>=0.000
84 -> 92 -0.11375
85 -> 92 0.20472
86 -> 92 -0.12769
87 -> 92 -0.23682
88 -> 92 0.32437
89 -> 92 0.23486
89 -> 93 0.24510
90 -> 92 -0.32089
90 -> 93 -0.12998

Excited State 10: Singlet-A 5.5146 eV 224.83 nm f=0.0635 <S**2>=0.000
85 -> 92 -0.12359
86 -> 92 -0.20740
87 -> 92 0.22159
88 -> 92 0.40914
89 -> 92 0.11640
90 -> 92 0.40288
91 -> 97 0.11424

Excited State 11: Singlet-A 5.5713 eV 222.54 nm f=0.0492 <S**2>=0.000
85 -> 92 -0.17791
86 -> 92 0.22975
88 -> 92 0.38797
88 -> 94 0.14394
89 -> 92 -0.21201
89 -> 93 -0.25004
90 -> 92 -0.23726

Excited State 12: Singlet-A 5.6684 eV 218.73 nm f=0.0007 <S**2>=0.000
91 -> 99 0.59397
91 ->100 0.29522
91 ->102 -0.12363

Excited State 13: Singlet-A 5.7326 eV 216.28 nm f=0.0305 <S**2>=0.000
84 -> 92 -0.16383
85 -> 92 0.12815
86 -> 92 0.12968
86 -> 93 0.10962
86 -> 95 -0.13153
87 -> 94 0.13928
88 -> 93 -0.10563
88 -> 96 -0.10262
88 -> 97 0.11000
89 -> 92 0.11276
89 -> 94 0.29596
90 -> 92 0.12354
90 -> 95 0.18069
91 -> 97 -0.16804
91 ->101 -0.14810
91 ->103 0.25845

Excited State 14: Singlet-A 5.7684 eV 214.94 nm f=0.0199 <S**2>=0.000

86 -> 92 -0.16966
86 -> 95 0.10145
87 -> 95 -0.11914
90 -> 92 -0.11064
91 ->100 -0.18105
91 ->101 -0.26598
91 ->103 0.47596

Excited State 15: Singlet-A 5.7756 eV 214.67 nm f=0.0044 <S**2>=0.000
85 -> 92 0.22843
86 -> 93 0.10845
86 -> 97 0.10934
87 -> 92 0.10101
87 -> 95 -0.15637
88 -> 92 -0.13916
88 -> 94 0.33190
88 -> 95 0.14034
89 -> 92 0.13992
89 -> 96 -0.13051
90 -> 92 0.14210
90 -> 93 -0.18926
90 -> 95 -0.19105

Excited State 16: Singlet-A 5.8553 eV 211.75 nm f=0.2573 <S**2>=0.000
84 -> 92 -0.15395
87 -> 92 0.48293
89 -> 92 -0.11001
89 -> 93 0.20721
90 -> 92 -0.23859
90 -> 93 -0.21730
91 -> 98 0.11344

Excited State 17: Singlet-A 5.9363 eV 208.86 nm f=0.2984 <S**2>=0.000
85 -> 92 -0.24180
86 -> 92 0.44242
87 -> 92 -0.13543
88 -> 93 0.13739
89 -> 93 0.31772

Excited State 18: Singlet-A 5.9659 eV 207.82 nm f=0.0431 <S**2>=0.000
84 -> 92 0.11846
85 -> 92 0.31630
87 -> 92 0.22827
87 -> 93 0.11380
89 -> 92 -0.13174
89 -> 93 0.15640
89 -> 94 -0.14072
90 -> 93 0.29799
90 -> 96 0.18400
91 -> 98 -0.15956

Excited State 19: Singlet-A 5.9827 eV 207.24 nm f=0.0030 <S**2>=0.000
91 -> 99 -0.28059
91 ->100 0.50914
91 ->101 -0.19373
91 ->102 -0.18808
91 ->103 0.10486
91 ->105 0.10863

Excited State 20: Singlet-A 6.0492 eV 204.96 nm f=0.0064 <S**2>=0.000
84 -> 92 0.22429

```
     85 -> 92         0.16374
     85 -> 93        -0.10614
     86 -> 92         0.18901
     87 -> 93         0.13875
     88 -> 92         0.11659
     88 -> 94        -0.11786
     90 -> 93        -0.13228
     90 -> 94         0.10216
     90 -> 96        -0.11528
     91 -> 98         0.11493
     91 ->100         0.18486
     91 ->101        -0.13120
     91 ->102         0.38029

 Excited State  21:      Singlet-A      6.0618 eV  204.53 nm  f=0.0241  <S**2>=0.000
     84 -> 92        -0.22793
     86 -> 92        -0.11302
     87 -> 93        -0.11646
     88 -> 93         0.10647
     89 -> 93        -0.12445
     90 -> 93         0.14073
     90 -> 96         0.10734
     91 ->100         0.12829
     91 ->102         0.48362

 Excited State  22:      Singlet-A      6.1064 eV  203.04 nm  f=0.1051  <S**2>=0.000
     84 -> 92         0.15824
     85 -> 92        -0.26537
     86 -> 92        -0.22744
     86 -> 93         0.13487
     87 -> 93         0.11003
     88 -> 93        -0.30229
     88 -> 94         0.15139
     88 -> 95        -0.11514
     89 -> 93         0.29516
     89 -> 94         0.16517

 Excited State  23:      Singlet-A      6.1165 eV  202.70 nm  f=0.0086  <S**2>=0.000
     84 -> 92         0.17798
     88 -> 93         0.36641
     88 -> 95        -0.16534
     89 -> 94         0.20762
     89 -> 95         0.19443
     90 -> 94        -0.37604

 Excited State  24:      Singlet-A      6.1446 eV  201.78 nm  f=0.0294  <S**2>=0.000
     84 -> 92        -0.18987
     86 -> 94         0.11571
     87 -> 94        -0.14556
     88 -> 93        -0.28654
     88 -> 94        -0.11174
     88 -> 97        -0.10425
     89 -> 95         0.30493
     89 -> 97        -0.11076
     90 -> 94        -0.17100
     90 -> 95        -0.24014
     91 ->101        -0.15861

 Excited State  25:      Singlet-A      6.1765 eV  200.74 nm  f=0.0021  <S**2>=0.000
     88 -> 93        -0.12511
     91 -> 99        -0.10280
```

91 ->101 0.51628
91 ->102 0.11189
91 ->103 0.33583
91 ->107 -0.11857

7.4.3 Computed xyz-coordinates (S_1 geometry) of compound 7a (CAM-B3LYP/6-311+G* PCM Toluene)

N	0.847587	-0.014061	-0.388176
C	2.180706	-0.288037	-0.010028
C	0.036128	-1.059026	-0.859470
C	0.417643	1.317658	-0.515610
C	-0.943004	1.692268	-0.211957
C	-1.301054	3.004768	-0.595798
C	-0.408683	3.894098	-1.147906
C	0.930986	3.527007	-1.368909
C	1.320992	2.245038	-1.060910
C	2.908192	-1.279383	-0.681919
C	4.221578	-1.525321	-0.328396
C	4.822556	-0.792453	0.690188
C	4.103326	0.198032	1.348524
C	2.790301	0.460135	1.002037
C	0.078548	-2.315523	-0.240942
C	-0.730821	-3.332398	-0.704606
C	-1.590991	-3.109042	-1.780243
C	-1.634787	-1.863340	-2.391429
C	-0.828622	-0.832906	-1.936302
C	-1.910709	0.858903	0.467016
C	-3.251958	0.854691	0.014493
C	-4.204423	0.014430	0.528948
C	-3.868312	-0.882671	1.575017
C	-2.605489	-0.861241	2.096956
C	-1.611284	0.021411	1.600379
C	-0.441161	0.164983	2.365168
N	0.491214	0.279905	3.047688
H	-2.305490	3.342535	-0.371840
H	-0.739120	4.898690	-1.387940
H	1.636375	4.226139	-1.799917
H	2.326600	1.916722	-1.292833
H	2.450956	-1.827202	-1.495178
H	4.784615	-2.283328	-0.859855
H	5.851554	-0.990852	0.966218
H	4.562950	0.764164	2.149533
H	2.221095	1.206634	1.534163
H	0.714855	-2.465657	0.621531
H	-0.709876	-4.298885	-0.215915
H	-2.228896	-3.909437	-2.136395
H	-2.297271	-1.692198	-3.231390
H	-0.831790	0.131307	-2.426535
H	-3.514765	1.492773	-0.822596
H	-5.208644	0.022866	0.120673
H	-4.611863	-1.563187	1.973450
H	-2.350841	-1.504478	2.932439

SCF Done: E(RCAM-B3LYP) = -1072.60783312 A.U. after 2 cycles
Zero-point correction= 0.358547 (Hartree/Particle)
Thermal correction to Energy= 0.379525
Thermal correction to Enthalpy= 0.380469
Thermal correction to Gibbs Free Energy= 0.308465

Sum of electronic and zero-point Energies= -1072.136042
Sum of electronic and thermal Energies= -1072.115065
Sum of electronic and thermal Enthalpies= -1072.114120
Sum of electronic and thermal Free Energies= -1072.186125

7.4.4 Computed emission energies of compound 7a (CAM-B3LYP/6-311+G* PCM Toluene, TD=(Nstates=6,root=1))

```
Excited State   1:      Singlet-A      2.8927 eV  428.60 nm  f=0.0330  <S**2>=0.000
      91 -> 92         0.69082
 This state for optimization and/or second-order correction.
 Total Energy, E(TD-HF/TD-DFT) =  -1072.50129867
 Copying the excited state density for this state as the 1-particle RhoCI density.

 Excited State   2:      Singlet-A      4.0844 eV  303.56 nm  f=0.0521  <S**2>=0.000
      90 -> 92         0.14949
      91 -> 93        -0.57682
      91 -> 94         0.29287
      91 -> 97         0.12036

 Excited State   3:      Singlet-A      4.2055 eV  294.81 nm  f=0.1054  <S**2>=0.000
      90 -> 92         0.27511
      91 -> 93         0.34092
      91 -> 94         0.49696
      91 -> 96        -0.12917

 Excited State   4:      Singlet-A      4.4115 eV  281.05 nm  f=0.2375  <S**2>=0.000
      89 -> 92        -0.15718
      90 -> 92         0.37448
      90 -> 93         0.11879
      91 -> 94        -0.26433
      91 -> 95         0.18625
      91 -> 96        -0.39162
      91 -> 97        -0.11872

 Excited State   5:      Singlet-A      4.4218 eV  280.39 nm  f=0.1158  <S**2>=0.000
      90 -> 92         0.42283
      91 -> 94        -0.15841
      91 -> 95        -0.11316
      91 -> 96         0.46685

 Excited State   6:      Singlet-A      4.6165 eV  268.57 nm  f=0.0305  <S**2>=0.000
      86 -> 92         0.10683
      91 -> 95         0.19498
      91 -> 97         0.44775
      91 -> 98         0.33105
      91 ->100         0.10887
      91 ->101        -0.13908
```

7.5 Quantum chemical calculation data of 2″-(diphenylamino)-[1,1′:4′,1″-terphenyl]-2-carbonitrile (7b)

7.5.1 Computed xyz-coordinates (S_0 geometry) of compound 7b

(CAM-B3LYP/6-311+G* PCM Toluene)

N   -2.361618 -0.213337   0.184919

C	-3.113309	-1.038774	-0.674809
C	-1.642466	-0.749170	1.286233
C	-2.517029	1.201868	0.108285
C	-1.403098	2.040798	-0.043057
C	-1.608641	3.422146	-0.036190
C	-2.877309	3.967207	0.081065
C	-3.976566	3.127090	0.202671
C	-3.790912	1.754988	0.225249
C	-3.597279	-2.282168	-0.257277
C	-4.330757	-3.078746	-1.123716
C	-4.611899	-2.655128	-2.416185
C	-4.144005	-1.415209	-2.831725
C	-3.399078	-0.616668	-1.977627
C	-0.808636	-1.854895	1.116824
C	-0.095593	-2.364483	2.189468
C	-0.187609	-1.772790	3.444060
C	-1.008463	-0.667637	3.613365
C	-1.737419	-0.160701	2.545514
C	-0.023935	1.518254	-0.226743
C	1.019106	1.977274	0.574749
C	2.318279	1.531871	0.383780
C	2.615059	0.617737	-0.624281
C	1.571866	0.161393	-1.431734
C	0.273863	0.598817	-1.232224
C	4.003563	0.159944	-0.876045
C	4.520449	0.208887	-2.170708
C	5.814098	-0.201815	-2.451397
C	6.633919	-0.677642	-1.436183
C	6.146378	-0.743507	-0.143919
C	4.841087	-0.331670	0.138970
C	4.387389	-0.470134	1.492103
N	4.076124	-0.607822	2.589781
H	-0.752745	4.076724	-0.158187
H	-3.007595	5.043352	0.068359
H	-4.975226	3.538803	0.295655
H	-4.638522	1.089765	0.343917
H	-3.401168	-2.624457	0.750696
H	-4.696625	-4.038609	-0.775270
H	-5.189516	-3.279687	-3.087688
H	-4.352692	-1.063912	-3.836527
H	-3.044203	0.348376	-2.318932
H	-0.716270	-2.307960	0.137174
H	0.555288	-3.218676	2.039209
H	0.385510	-2.163709	4.276577
H	-1.089628	-0.194021	4.585611
H	-2.379961	0.700008	2.688322
H	0.812192	2.685523	1.369165
H	3.107170	1.913757	1.021137
H	1.776848	-0.557209	-2.217703
H	-0.517301	0.222736	-1.869737
H	3.896602	0.597748	-2.966861
H	6.186522	-0.142836	-3.467761
H	7.646606	-0.997586	-1.650034
H	6.766851	-1.122840	0.658869

SCF Done: E(RCAM-B3LYP) = -1303.59400253 A.U. after 2 cycles
Zero-point correction= 0.442452 (Hartree/Particle)
Thermal correction to Energy= 0.468224
Thermal correction to Enthalpy= 0.469169
Thermal correction to Gibbs Free Energy= 0.383522
Sum of electronic and zero-point Energies= -1303.151551

Sum of electronic and thermal Energies=	-1303.125778
Sum of electronic and thermal Enthalpies=	-1303.124834
Sum of electronic and thermal Free Energies=	-1303.210480

(BMK/6-311+G* PCM Toluene)

N	-2.427354	-0.112162	0.289224
C	-3.163032	-1.181860	-0.257538
C	-1.706845	-0.240328	1.501780
C	-2.538493	1.179368	-0.302954
C	-1.395258	1.884627	-0.739552
C	-1.572107	3.182510	-1.251506
C	-2.835623	3.760023	-1.362410
C	-3.962142	3.041833	-0.951924
C	-3.807686	1.764449	-0.418760
C	-3.702491	-2.195725	0.556308
C	-4.427236	-3.244237	-0.009075
C	-4.645434	-3.304379	-1.388706
C	-4.123932	-2.292893	-2.198984
C	-3.386528	-1.245576	-1.646263
C	-0.946399	-1.391887	1.766024
C	-0.231992	-1.505040	2.956965
C	-0.244724	-0.469686	3.897086
C	-0.986121	0.682103	3.629657
C	-1.717666	0.797793	2.445693
C	-0.019334	1.305187	-0.706217
C	1.057040	2.065018	-0.224881
C	2.360011	1.568908	-0.253018
C	2.627271	0.292861	-0.768578
C	1.549215	-0.472019	-1.246292
C	0.248353	0.022019	-1.210415
C	4.015618	-0.241267	-0.865245
C	4.442191	-0.842226	-2.060897
C	5.732209	-1.350806	-2.204199
C	6.642910	-1.273475	-1.146795
C	6.249725	-0.688151	0.052089
C	4.947560	-0.176897	0.196668
C	4.603411	0.375037	1.482020
N	4.389198	0.801672	2.529170
H	-0.699916	3.728385	-1.601026
H	-2.940549	4.758994	-1.776200
H	-4.953770	3.478463	-1.032323
H	-4.671713	1.202620	-0.074719
H	-3.556376	-2.157243	1.630586
H	-4.836102	-4.013697	0.640771
H	-5.215010	-4.120975	-1.822413
H	-4.283613	-2.317888	-3.273833
H	-2.994134	-0.464269	-2.290091
H	-0.916795	-2.192803	1.033752
H	0.354601	-2.400996	3.141953
H	0.325377	-0.556151	4.817260
H	-1.003960	1.498078	4.347244
H	-2.296023	1.695565	2.249621
H	0.875050	3.055591	0.182815
H	3.170820	2.191503	0.112690
H	1.729989	-1.469277	-1.638498
H	-0.563879	-0.592257	-1.587668
H	3.751313	-0.887011	-2.897656
H	6.029780	-1.801205	-3.147119
H	7.649436	-1.666144	-1.254296
H	6.937734	-0.627558	0.889901

SCF Done: E(RBMK) = -1303.42826076 A.U. after 2 cycles
Zero-point correction= 0.439691 (Hartree/Particle)
Thermal correction to Energy= 0.465395
Thermal correction to Enthalpy= 0.466339
Thermal correction to Gibbs Free Energy= 0.381769
Sum of electronic and zero-point Energies= -1302.988570
Sum of electronic and thermal Energies= -1302.962866
Sum of electronic and thermal Enthalpies= -1302.961922
Sum of electronic and thermal Free Energies= -1303.046492

7.5.2 Computed excitations energies of compound 7b

(CAM-B3LYP/6-311+G* PCM Toluene, TD=(Nstates=25,root=1))

```
Excited State   1:      Singlet-A      4.0636 eV  305.11 nm  f=0.1853  <S**2>=0.000
     111 ->112         0.46852
     111 ->113        -0.37838
     111 ->115         0.31100
 This state for optimization and/or second-order correction.
 Total Energy, E(TD-HF/TD-DFT) =  -1303.44466934
 Copying the excited state density for this state as the 1-particle RhoCI density.

 Excited State   2:      Singlet-A      4.3177 eV  287.16 nm  f=0.0554  <S**2>=0.000
     111 ->114         0.64291
     111 ->115        -0.11173
     111 ->116        -0.12824
     111 ->120        -0.10751

 Excited State   3:      Singlet-A      4.5149 eV  274.61 nm  f=0.3071  <S**2>=0.000
     111 ->115        -0.10446
     111 ->116         0.52023
     111 ->117        -0.32479
     111 ->118        -0.23507
     111 ->119         0.12376

 Excited State   4:      Singlet-A      4.6805 eV  264.90 nm  f=0.4401  <S**2>=0.000
     103 ->112        -0.12719
     104 ->112         0.10943
     110 ->112         0.48687
     111 ->112        -0.21388
     111 ->113        -0.13454
     111 ->115         0.23738
     111 ->116         0.12680
     111 ->119        -0.11282
     111 ->120        -0.13366

 Excited State   5:      Singlet-A      4.7600 eV  260.47 nm  f=0.1594  <S**2>=0.000
     110 ->112         0.34677
     111 ->112         0.23252
     111 ->115        -0.20764
     111 ->118        -0.15319
     111 ->119         0.22955
     111 ->120         0.30299
     111 ->122        -0.13279

 Excited State   6:      Singlet-A      4.9324 eV  251.36 nm  f=0.0926  <S**2>=0.000
     110 ->112         0.14455
     111 ->112         0.29139
```

```
     111 ->113         0.21913
     111 ->116        -0.12733
     111 ->117        -0.18537
     111 ->118        -0.12497
     111 ->120        -0.25257
     111 ->123        -0.22322
     111 ->124         0.18440

 Excited State   7:      Singlet-A      4.9619 eV  249.87 nm  f=0.0383  <S**2>=0.000
     109 ->116        -0.10239
     111 ->112        -0.23344
     111 ->113        -0.19665
     111 ->115         0.11393
     111 ->118        -0.16016
     111 ->119         0.13328
     111 ->122        -0.21278
     111 ->123        -0.23334
     111 ->124         0.30383
     111 ->125        -0.10166

 Excited State   8:      Singlet-A      5.0613 eV  244.97 nm  f=0.0583  <S**2>=0.000
     103 ->112         0.16928
     104 ->113        -0.11432
     106 ->112         0.19504
     107 ->112         0.18063
     107 ->113        -0.11169
     109 ->112        -0.11068
     109 ->113         0.10313
     110 ->113        -0.30830
     110 ->116         0.20046
     110 ->118         0.14537
     111 ->112        -0.11671
     111 ->115         0.17027
     111 ->118        -0.12123

 Excited State   9:      Singlet-A      5.0980 eV  243.20 nm  f=0.1287  <S**2>=0.000
     102 ->112         0.10425
     103 ->112        -0.26013
     104 ->113         0.13320
     104 ->115         0.11293
     106 ->112         0.14162
     106 ->113        -0.14455
     107 ->112         0.14526
     110 ->113         0.35285
     110 ->114         0.13982
     110 ->116         0.15752
     110 ->118         0.13259
     111 ->116        -0.10232

 Excited State  10:      Singlet-A      5.2469 eV  236.30 nm  f=0.0260  <S**2>=0.000
     106 ->112         0.11911
     111 ->113        -0.14159
     111 ->115        -0.22216
     111 ->116         0.23224
     111 ->117         0.38967
     111 ->118         0.15619
     111 ->119         0.14769
     111 ->120        -0.11940
     111 ->124         0.13473
     111 ->127        -0.10265
```

```
Excited State  11:      Singlet-A      5.3424 eV  232.08 nm  f=0.0155  <S**2>=0.000
    103 ->112       -0.12134
    110 ->114       -0.10264
    111 ->113        0.37618
    111 ->115        0.17262
    111 ->116        0.10880
    111 ->117        0.22645
    111 ->119        0.12140
    111 ->120       -0.10247
    111 ->121       -0.21883
    111 ->126       -0.16683
    111 ->127        0.13604

Excited State  12:      Singlet-A      5.4426 eV  227.80 nm  f=0.0154  <S**2>=0.000
    106 ->112        0.10123
    107 ->112        0.11004
    111 ->113        0.14991
    111 ->114        0.11621
    111 ->116        0.21721
    111 ->118        0.29834
    111 ->119       -0.27152
    111 ->120        0.25591
    111 ->121        0.18352
    111 ->122       -0.10572
    111 ->124        0.13742

Excited State  13:      Singlet-A      5.5317 eV  224.13 nm  f=0.3337  <S**2>=0.000
    103 ->112       -0.26825
    103 ->113       -0.16614
    104 ->112       -0.30389
    105 ->112       -0.11461
    108 ->112        0.11742
    110 ->113       -0.23804
    110 ->115        0.26831
    110 ->116        0.10744
    111 ->113       -0.12261
    111 ->114       -0.10632
    111 ->115       -0.13336
    111 ->120       -0.10155

Excited State  14:      Singlet-A      5.6080 eV  221.09 nm  f=0.0665  <S**2>=0.000
    103 ->112        0.16471
    104 ->112        0.19084
    104 ->114        0.12121
    105 ->113        0.11384
    105 ->115       -0.11614
    108 ->115        0.10446
    110 ->113        0.15669
    110 ->114       -0.29683
    110 ->116        0.11523
    111 ->115       -0.13521
    111 ->118        0.10146
    111 ->120       -0.15697
    111 ->126        0.15717

Excited State  15:      Singlet-A      5.7421 eV  215.92 nm  f=0.0134  <S**2>=0.000
    111 ->115       -0.14443
    111 ->119       -0.12555
    111 ->122        0.30432
    111 ->123       -0.25344
    111 ->124       -0.21311
```

```
   111 ->125       -0.13046
   111 ->126       -0.21547
   111 ->129        0.13742
   111 ->132        0.19831

Excited State  16:      Singlet-A      5.7908 eV  214.11 nm  f=0.0348  <S**2>=0.000
   111 ->115        0.13381
   111 ->119        0.22154
   111 ->120        0.10360
   111 ->122        0.44817
   111 ->124        0.14971
   111 ->125       -0.20791
   111 ->127       -0.11956
   111 ->132       -0.15678

Excited State  17:      Singlet-A      5.8181 eV  213.10 nm  f=0.0276  <S**2>=0.000
   111 ->119        0.14646
   111 ->120       -0.24017
   111 ->121        0.48668
   111 ->126       -0.17284
   111 ->128        0.21314
   111 ->132        0.14222

Excited State  18:      Singlet-A      5.8829 eV  210.75 nm  f=0.1395  <S**2>=0.000
   103 ->112        0.18131
   107 ->112       -0.11290
   108 ->112        0.10740
   109 ->112        0.33101
   109 ->113       -0.12243
   109 ->116       -0.14047
   110 ->114        0.14392
   110 ->116        0.20689
   110 ->117        0.10874
   110 ->118        0.11290
   111 ->118        0.11415
   111 ->121       -0.10873
   111 ->123        0.14919

Excited State  19:      Singlet-A      5.9049 eV  209.97 nm  f=0.0146  <S**2>=0.000
   102 ->112        0.31621
   103 ->113        0.25535
   103 ->115        0.14818
   104 ->112        0.24423
   104 ->113        0.13165
   106 ->112       -0.13801
   110 ->112       -0.22162
   110 ->113       -0.30752

Excited State  20:      Singlet-A      5.9118 eV  209.72 nm  f=0.0589  <S**2>=0.000
   103 ->112        0.16314
   104 ->113        0.10917
   107 ->112        0.13550
   109 ->112       -0.19014
   109 ->116        0.13483
   110 ->114        0.10840
   110 ->115        0.16938
   110 ->118       -0.13354
   111 ->117       -0.11274
   111 ->118        0.13056
   111 ->123        0.12476
   111 ->124        0.21846
```

111 ->127 0.15750
111 ->130 0.10079
111 ->132 0.12293

Excited State 21: Singlet-A 5.9508 eV 208.35 nm f=0.0922 <S**2>=0.000
106 ->112 -0.15771
107 ->112 -0.21512
109 ->116 0.23649
109 ->117 -0.15337
109 ->118 -0.12079
110 ->114 0.17060
110 ->116 0.22835
111 ->117 0.10002
111 ->120 0.13496
111 ->121 0.11261
111 ->123 -0.16793
111 ->124 0.11497

Excited State 22: Singlet-A 5.9853 eV 207.15 nm f=0.1439 <S**2>=0.000
103 ->112 0.16705
104 ->113 0.15637
106 ->112 0.15586
107 ->112 0.15592
109 ->113 -0.10404
110 ->114 0.10103
110 ->115 0.25737
111 ->115 0.10643
111 ->118 -0.12812
111 ->124 -0.11165
111 ->127 -0.14657
111 ->132 -0.24619

Excited State 23: Singlet-A 6.0661 eV 204.39 nm f=0.0711 <S**2>=0.000
107 ->114 0.12841
107 ->116 0.10916
108 ->112 0.26894
108 ->113 -0.19815
108 ->114 0.10366
108 ->115 0.21430
110 ->115 -0.15793
111 ->120 0.13663
111 ->123 0.11969
111 ->124 -0.16189
111 ->126 0.18826

Excited State 24: Singlet-A 6.0981 eV 203.32 nm f=0.0127 <S**2>=0.000
106 ->112 -0.15066
109 ->112 -0.20838
109 ->113 0.11417
109 ->114 -0.11419
111 ->115 0.11248
111 ->125 -0.15470
111 ->126 0.29982
111 ->129 0.24458
111 ->130 0.14645
111 ->136 0.12332
111 ->139 0.10686

Excited State 25: Singlet-A 6.1109 eV 202.89 nm f=0.0070 <S**2>=0.000
106 ->112 0.19228
107 ->112 0.12951

```
   108 ->114       -0.13197
   109 ->112        0.30126
   109 ->113       -0.18649
   109 ->115        0.11545
   109 ->116        0.19252
   110 ->115       -0.10196
   111 ->125       -0.12685
   111 ->126        0.13486
   111 ->129        0.16089
   111 ->130        0.12288
   111 ->132        0.10140
```

(BMK/6-311+G* PCM Toluene, TD=(Nstates=25,root=1))

```
Excited State   1:      Singlet-A      3.6750 eV  337.37 nm  f=0.1143  <S**2>=0.000
   111 ->112        0.59804
   111 ->113       -0.29661
   111 ->115        0.19687
 This state for optimization and/or second-order correction.
 Total Energy, E(TD-HF/TD-DFT) =  -1303.29320762
 Copying the excited state density for this state as the 1-particle RhoCI density.

 Excited State   2:      Singlet-A      4.1759 eV  296.91 nm  f=0.0473  <S**2>=0.000
   111 ->114        0.62085
   111 ->115       -0.26122

 Excited State   3:      Singlet-A      4.3290 eV  286.40 nm  f=0.0655  <S**2>=0.000
   111 ->112        0.35163
   111 ->113        0.46420
   111 ->115       -0.33805
   111 ->116       -0.12466
   111 ->118        0.10223

 Excited State   4:      Singlet-A      4.3859 eV  282.69 nm  f=0.3250  <S**2>=0.000
   111 ->116        0.57455
   111 ->117       -0.36644

 Excited State   5:      Singlet-A      4.5042 eV  275.26 nm  f=0.6952  <S**2>=0.000
   110 ->112        0.65716

 Excited State   6:      Singlet-A      4.6373 eV  267.36 nm  f=0.0660  <S**2>=0.000
   111 ->113       -0.32183
   111 ->115       -0.26817
   111 ->116       -0.14005
   111 ->117       -0.18711
   111 ->118        0.46020

 Excited State   7:      Singlet-A      4.7711 eV  259.87 nm  f=0.0506  <S**2>=0.000
   111 ->116        0.24972
   111 ->117        0.43811
   111 ->118        0.28600
   111 ->119        0.31713

 Excited State   8:      Singlet-A      4.8981 eV  253.13 nm  f=0.0499  <S**2>=0.000
   103 ->112       -0.12676
   110 ->113       -0.25605
   111 ->113        0.21632
   111 ->114        0.18467
   111 ->115        0.34897
   111 ->117       -0.15586
   111 ->118        0.26329
```

111 ->122 0.15545

Excited State 9: Singlet-A 4.9600 eV 249.97 nm f=0.0239 <S**2>=0.000
106 ->112 0.24122
106 ->113 -0.10258
107 ->112 0.20608
109 ->112 -0.19783
110 ->116 0.22816
110 ->117 0.16036
111 ->115 -0.10292
111 ->118 -0.11577
111 ->119 0.36969

Excited State 10: Singlet-A 4.9894 eV 248.49 nm f=0.1564 <S**2>=0.000
103 ->112 0.25477
104 ->113 0.15189
110 ->113 0.50884
111 ->113 0.15516
111 ->114 0.11597
111 ->115 0.14122
111 ->118 0.10256

Excited State 11: Singlet-A 5.0806 eV 244.03 nm f=0.0087 <S**2>=0.000
106 ->112 -0.20954
107 ->112 -0.19485
109 ->112 0.20380
110 ->116 -0.14233
110 ->117 -0.13088
111 ->116 -0.19840
111 ->117 -0.27709
111 ->119 0.38036

Excited State 12: Singlet-A 5.3664 eV 231.04 nm f=0.0451 <S**2>=0.000
103 ->112 0.10605
104 ->112 -0.15460
105 ->112 -0.15463
105 ->113 0.13278
105 ->114 -0.10825
108 ->112 0.22521
108 ->113 -0.13020
108 ->115 0.13216
110 ->114 -0.23007
110 ->115 0.33837
111 ->114 -0.12293
111 ->115 -0.10077
111 ->118 -0.15199

Excited State 13: Singlet-A 5.4479 eV 227.58 nm f=0.1229 <S**2>=0.000
103 ->112 0.29707
103 ->113 0.11040
104 ->112 -0.23056
105 ->112 0.10730
106 ->112 0.13007
109 ->112 0.27969
110 ->113 -0.21940
110 ->114 0.28677
110 ->115 0.12205
110 ->118 0.11445

Excited State 14: Singlet-A 5.5121 eV 224.93 nm f=0.0918 <S**2>=0.000
103 ->112 -0.23016

106 ->112 0.11362
108 ->112 0.17606
109 ->112 0.48304
110 ->113 0.19769
110 ->116 0.15809
110 ->117 0.14726

Excited State 15: Singlet-A 5.5684 eV 222.66 nm f=0.0055 <S**2>=0.000
111 ->120 0.48969
111 ->121 -0.36582
111 ->122 -0.18464
111 ->123 -0.11875
111 ->124 0.10507

Excited State 16: Singlet-A 5.6078 eV 221.09 nm f=0.0628 <S**2>=0.000
103 ->112 -0.13634
108 ->112 0.15526
109 ->112 -0.10651
110 ->114 0.14639
110 ->116 -0.12775
111 ->121 -0.17673
111 ->122 0.47113
111 ->124 0.12537
111 ->125 -0.11311
111 ->127 0.15235

Excited State 17: Singlet-A 5.6503 eV 219.43 nm f=0.0060 <S**2>=0.000
104 ->112 -0.15530
106 ->112 0.11050
107 ->112 0.16441
108 ->112 0.37717
108 ->113 -0.11631
109 ->112 -0.13536
110 ->115 -0.13614
110 ->116 -0.22359
110 ->117 -0.11923
111 ->119 0.10203
111 ->121 0.12837
111 ->122 -0.24364

Excited State 18: Singlet-A 5.6920 eV 217.82 nm f=0.0171 <S**2>=0.000
102 ->112 0.18692
103 ->112 0.17224
103 ->113 -0.18911
104 ->112 0.39167
105 ->112 -0.13284
107 ->112 0.20249
108 ->112 0.15939
110 ->113 -0.16369
110 ->115 -0.15164
110 ->116 -0.10128

Excited State 19: Singlet-A 5.7257 eV 216.54 nm f=0.0756 <S**2>=0.000
106 ->112 0.32549
106 ->113 0.10367
107 ->112 0.22962
108 ->112 -0.27841
108 ->113 0.13514
109 ->113 -0.18491
110 ->114 -0.25360
110 ->116 -0.19050

111 ->122 0.10839

Excited State 20: Singlet-A 5.7933 eV 214.01 nm f=0.1961 <S**2>=0.000
103 ->112 -0.25042
104 ->112 0.22318
107 ->113 0.10030
109 ->113 -0.16206
110 ->114 0.28800
110 ->115 0.38510
110 ->117 -0.11899

Excited State 21: Singlet-A 5.8644 eV 211.42 nm f=0.0054 <S**2>=0.000
105 ->112 -0.17903
106 ->112 0.17267
107 ->112 -0.12040
107 ->113 0.11081
111 ->120 -0.15668
111 ->122 -0.21134
111 ->124 0.24349
111 ->125 -0.26844
111 ->126 -0.20211
111 ->127 0.28339

Excited State 22: Singlet-A 5.8812 eV 210.82 nm f=0.0642 <S**2>=0.000
106 ->112 0.22874
107 ->112 -0.19364
107 ->113 0.20126
107 ->115 -0.10339
108 ->114 -0.10319
108 ->119 -0.11708
109 ->114 -0.10480
109 ->116 0.31284
109 ->117 -0.18065
110 ->115 -0.17844
111 ->119 -0.12057

Excited State 23: Singlet-A 5.9383 eV 208.79 nm f=0.0241 <S**2>=0.000
106 ->112 -0.23479
107 ->112 0.31411
107 ->113 -0.11761
108 ->114 -0.17958
109 ->114 -0.13329
109 ->116 0.15078
111 ->119 -0.17090
111 ->122 -0.15106
111 ->123 -0.11270
111 ->125 -0.10008
111 ->127 0.10667

Excited State 24: Singlet-A 5.9689 eV 207.72 nm f=0.0050 <S**2>=0.000
111 ->121 -0.12331
111 ->123 0.62751
111 ->126 -0.13129

Excited State 25: Singlet-A 5.9709 eV 207.65 nm f=0.1154 <S**2>=0.000
104 ->112 0.11359
105 ->112 0.28034
105 ->113 -0.13937
107 ->112 0.12034
107 ->114 0.11269
108 ->116 -0.10389

109 ->113	0.20577
109 ->117	-0.12922
110 ->114	-0.22809
110 ->115	0.19706
110 ->117	-0.18071

7.5.3 Computed xyz-coordinates (S_1 geometry) of compound 7b (CAM-B3LYP/6-311+G* PCM Toluene)

N	-2.426161	-0.066355	0.246275
C	-3.243051	-1.158827	-0.117681
C	-1.693449	-0.087840	1.447379
C	-2.493941	1.119201	-0.501371
C	-1.285598	1.865622	-0.806544
C	-1.501578	3.201175	-1.243483
C	-2.751630	3.693048	-1.511979
C	-3.906218	2.890488	-1.342750
C	-3.762056	1.625068	-0.828138
C	-3.935587	-1.889610	0.853585
C	-4.744314	-2.946159	0.470634
C	-4.874741	-3.283601	-0.871380
C	-4.195223	-2.548198	-1.837982
C	-3.388434	-1.487090	-1.469678
C	-1.114464	-1.276226	1.911197
C	-0.369164	-1.269122	3.071938
C	-0.177994	-0.081500	3.779279
C	-0.749177	1.096324	3.320056
C	-1.507779	1.102242	2.160252
C	0.018885	1.279719	-0.814956
C	1.193919	2.063656	-0.627494
C	2.441322	1.502061	-0.584170
C	2.643390	0.112661	-0.774548
C	1.493605	-0.657713	-1.032774
C	0.234349	-0.109684	-1.046203
C	3.968457	-0.496788	-0.771693
C	4.259774	-1.554170	-1.655596
C	5.506456	-2.140112	-1.719771
C	6.542654	-1.698106	-0.895119
C	6.293827	-0.675676	-0.003257
C	5.027261	-0.082134	0.075204
C	4.846618	0.888279	1.108275
N	4.755884	1.644457	1.971839
H	-0.640634	3.821611	-1.459018
H	-2.854707	4.704142	-1.890453
H	-4.890024	3.283028	-1.567583
H	-4.636595	1.025778	-0.600650
H	-3.854088	-1.612460	1.896519
H	-5.287860	-3.501507	1.226031
H	-5.509858	-4.111146	-1.164650
H	-4.291092	-2.806434	-2.885955
H	-2.860909	-0.911619	-2.219813
H	-1.222563	-2.187339	1.336987
H	0.089246	-2.187509	3.418823
H	0.420640	-0.079259	4.682389
H	-0.607758	2.019604	3.869115
H	-1.980857	2.011880	1.815372
H	1.105138	3.130675	-0.460990
H	3.292016	2.152415	-0.423420
H	1.593992	-1.723445	-1.208307
H	-0.602199	-0.754042	-1.281173

```
H    3.488095  -1.885663  -2.339466
H    5.684498  -2.938375  -2.432102
H    7.525779  -2.149768  -0.949206
H    7.074968  -0.329205   0.663356

SCF Done:  E(RCAM-B3LYP) =  -1303.57785820     A.U. after    2 cycles
Zero-point correction=                           0.439838 (Hartree/Particle)
Thermal correction to Energy=                    0.465661
Thermal correction to Enthalpy=                  0.466605
Thermal correction to Gibbs Free Energy=         0.382241
Sum of electronic and zero-point Energies=          -1303.021376
Sum of electronic and thermal Energies=             -1302.995553
Sum of electronic and thermal Enthalpies=           -1302.994608
Sum of electronic and thermal Free Energies=        -1303.078973
```

7.5.4 Computed emission energies of compound 7b (CAM-B3LYP/6-311+G* PCM Toluene, TD=(Nstates=6,root=1))

```
Excited State   1:      Singlet-A      3.0482 eV  406.74 nm  f=0.1881  <S**2>=0.000
    111 ->112       -0.62379
    111 ->113        0.22296
    111 ->114       -0.20036
 This state for optimization and/or second-order correction.
 Total Energy, E(TD-HF/TD-DFT) =  -1303.46577460
 Copying the excited state density for this state as the 1-particle RhoCI density.

 Excited State   2:      Singlet-A      3.9961 eV  310.26 nm  f=0.3637  <S**2>=0.000
    110 ->112       -0.58232
    111 ->113        0.13276
    111 ->115        0.26799
    111 ->117        0.11271

 Excited State   3:      Singlet-A      4.1449 eV  299.12 nm  f=0.3383  <S**2>=0.000
    110 ->112        0.23547
    111 ->112       -0.13438
    111 ->113       -0.15675
    111 ->114        0.33507
    111 ->115        0.44655
    111 ->116        0.12787
    111 ->117        0.12392

 Excited State   4:      Singlet-A      4.3022 eV  288.19 nm  f=0.0814  <S**2>=0.000
    110 ->112        0.22927
    110 ->113       -0.12723
    111 ->112        0.24661
    111 ->113        0.36705
    111 ->114       -0.25241
    111 ->115        0.18490
    111 ->117        0.29272

 Excited State   5:      Singlet-A      4.3930 eV  282.23 nm  f=0.2086  <S**2>=0.000
    110 ->113        0.11069
    111 ->113       -0.22906
    111 ->115       -0.14794
    111 ->117        0.51745
    111 ->119       -0.30413

 Excited State   6:      Singlet-A      4.4962 eV  275.75 nm  f=0.0082  <S**2>=0.000
```

105 ->112	0.11175
109 ->112	-0.10907
110 ->113	-0.19269
110 ->115	-0.14766
111 ->113	0.21653
111 ->114	0.22783
111 ->118	-0.10551
111 ->119	-0.43153
111 ->121	0.13411

7.6 Quantum chemical calculation data of 2″-(diphenylamino)-2′ -methyl-[1,1′:4′,1″-terphenyl]-2-carbonitrile (7c)

7.6.1 Computed xyz-coordinates (S_0 geometry) of compound 7c

(CAM-B3LYP/6-311+G* PCM Toluene)

N	-2.429771	-0.218381	0.175308
C	-3.157614	-0.829230	-0.864949
C	-1.748300	-0.987942	1.154976
C	-2.575494	1.182066	0.400688
C	-1.455283	2.024100	0.465020
C	-1.660022	3.373986	0.760358
C	-2.930305	3.892345	0.954895
C	-4.034332	3.054842	0.862969
C	-3.851156	1.708358	0.596972
C	-3.664014	-2.126487	-0.740270
C	-4.373601	-2.707986	-1.780220
C	-4.608544	-2.012661	-2.959355
C	-4.118583	-0.719021	-3.083433
C	-3.397072	-0.133080	-2.054685
C	-0.921173	-2.045602	0.775155
C	-0.243053	-2.782377	1.731813
C	-0.364342	-2.469698	3.080970
C	-1.178927	-1.413587	3.461074
C	-1.872666	-0.679417	2.507760
C	-0.068699	1.548546	0.215998
C	0.949811	1.838757	1.118674
C	2.252022	1.447252	0.852535
C	2.574078	0.768339	-0.318843
C	1.558439	0.453979	-1.233869
C	0.257344	0.849241	-0.944181
C	3.996535	0.418548	-0.589111
C	4.689287	1.052744	-1.618122
C	6.022143	0.769282	-1.873485
C	6.700148	-0.165626	-1.100925
C	6.036864	-0.810610	-0.073434
C	4.693248	-0.522266	0.182968
C	1.839254	-0.306261	-2.504414
C	4.047247	-1.230168	1.249763
N	3.575737	-1.825632	2.112126
H	-0.799761	4.032606	0.805387
H	-3.057741	4.947592	1.168230
H	-5.034692	3.445041	1.013056
H	-4.702957	1.039831	0.546222
H	-3.503998	-2.680086	0.175978
H	-4.757519	-3.715114	-1.658023
H	-5.167823	-2.470215	-3.767134
H	-4.291476	-0.156983	-3.994935
H	-3.024465	0.877793	-2.168879
H	-0.805379	-2.281073	-0.275936

H	0.406419	-3.592498	1.419917
H	0.183642	-3.036565	3.824362
H	-1.282738	-1.156278	4.509579
H	-2.510037	0.141396	2.814486
H	0.723759	2.361476	2.041110
H	3.035266	1.679883	1.565934
H	-0.527628	0.604852	-1.650949
H	4.172280	1.792970	-2.217598
H	6.536142	1.284537	-2.676962
H	7.741784	-0.389631	-1.297396
H	6.548804	-1.543717	0.537999
H	2.488560	-1.165419	-2.326096
H	0.912579	-0.671498	-2.947917
H	2.335776	0.316975	-3.252233

SCF Done: E(RCAM-B3LYP) = -1342.89191940 A.U. after 2 cycles
Zero-point correction= 0.470332 (Hartree/Particle)
Thermal correction to Energy= 0.497702
Thermal correction to Enthalpy= 0.498646
Thermal correction to Gibbs Free Energy= 0.411150
Sum of electronic and zero-point Energies= -1342.421588
Sum of electronic and thermal Energies= -1342.394218
Sum of electronic and thermal Enthalpies= -1342.393274
Sum of electronic and thermal Free Energies= -1342.480770

(BMK/6-311+G* PCM Toluene)

N	-2.503858	-0.032383	0.302831
C	-3.229185	-1.131908	-0.197074
C	-1.832157	-0.083564	1.547580
C	-2.584440	1.215815	-0.380581
C	-1.424789	1.884064	-0.832412
C	-1.583986	3.140422	-1.445736
C	-2.840865	3.711095	-1.636283
C	-3.982030	3.028783	-1.205737
C	-3.847105	1.794333	-0.575648
C	-3.815844	-2.079240	0.663091
C	-4.528188	-3.159423	0.143706
C	-4.686964	-3.317632	-1.236366
C	-4.118854	-2.372276	-2.093635
C	-3.393432	-1.293997	-1.586058
C	-1.111495	-1.229422	1.924570
C	-0.450722	-1.273959	3.150139
C	-0.476594	-0.174214	4.013778
C	-1.174325	0.972767	3.632550
C	-1.853275	1.020586	2.413081
C	-0.048313	1.313713	-0.720091
C	1.020116	2.123271	-0.309350
C	2.318279	1.620453	-0.293284
C	2.592692	0.303771	-0.690166
C	1.525141	-0.536448	-1.077925
C	0.227529	-0.012831	-1.080869
C	4.014103	-0.158289	-0.736840
C	4.566185	-0.610554	-1.944725
C	5.899374	-1.008218	-2.033860
C	6.725113	-0.966953	-0.906343
C	6.207422	-0.523236	0.305695
C	4.862498	-0.120440	0.391746
C	1.730093	-1.987322	-1.477174
C	4.383171	0.314372	1.679046
N	4.060054	0.653721	2.730090

H	-0.700773	3.657164	-1.811037
H	-2.928925	4.676226	-2.127199
H	-4.969204	3.460011	-1.346972
H	-4.722649	1.260268	-0.216777
H	-3.715418	-1.964123	1.737063
H	-4.974338	-3.875775	0.828789
H	-5.246819	-4.158600	-1.634764
H	-4.231927	-2.474035	-3.169886
H	-2.962996	-0.565535	-2.266554
H	-1.075036	-2.081512	1.252671
H	0.104506	-2.167340	3.423209
H	0.051918	-0.207514	4.961752
H	-1.199827	1.838770	4.288672
H	-2.399012	1.915190	2.128926
H	0.836947	3.146589	0.005685
H	3.135374	2.265814	0.018155
H	-0.591573	-0.660642	-1.381816
H	3.938345	-0.629941	-2.830407
H	6.297098	-1.344827	-2.987305
H	7.763945	-1.276049	-0.971501
H	6.830483	-0.486078	1.194164
H	2.530199	-2.458706	-0.900288
H	0.810571	-2.556505	-1.316447
H	1.990809	-2.083315	-2.537523

SCF Done: E(RBMK) = -1342.71972211   A.U. after   2 cycles
Zero-point correction=   0.467553 (Hartree/Particle)
Thermal correction to Energy=   0.494916
Thermal correction to Enthalpy=   0.495860
Thermal correction to Gibbs Free Energy=   0.408012
Sum of electronic and zero-point Energies=   -1342.252169
Sum of electronic and thermal Energies=   -1342.224806
Sum of electronic and thermal Enthalpies=   -1342.223862
Sum of electronic and thermal Free Energies=   -1342.311710

7.6.2 Computed excitations energies of compound 7c

(CAM-B3LYP/6-311+G* PCM Toluene, TD=(Nstates=25,root=1))

Excited State   1:   Singlet-A   4.1230 eV 300.71 nm f=0.1715 <S**2>=0.000
    115 ->116   0.28903
    115 ->117   0.51739
    115 ->118   -0.19117
    115 ->119   -0.26920
This state for optimization and/or second-order correction.
Total Energy, E(TD-HF/TD-DFT) = -1342.74040162
Copying the excited state density for this state as the 1-particle RhoCI density.

Excited State   2:   Singlet-A   4.3173 eV 287.18 nm f=0.0684 <S**2>=0.000
    115 ->118   0.52547
    115 ->119   -0.40359
    115 ->124   -0.11134

Excited State   3:   Singlet-A   4.5158 eV 274.55 nm f=0.3257 <S**2>=0.000
    115 ->120   0.55282
    115 ->121   0.32947
    115 ->123   0.19837

Excited State   4:   Singlet-A   4.7614 eV 260.39 nm f=0.1082 <S**2>=0.000

114 ->116 -0.15307
115 ->116 0.35831
115 ->119 0.18983
115 ->120 -0.12243
115 ->123 0.37544
115 ->124 -0.26790

Excited State 5: Singlet-A 4.8412 eV 256.10 nm f=0.0664 <S**2>=0.000
107 ->116 0.11946
108 ->116 0.11810
114 ->116 -0.32352
115 ->116 0.31499
115 ->117 -0.22290
115 ->122 0.12092
115 ->123 -0.14181
115 ->124 0.24920
115 ->126 -0.11309
115 ->130 -0.10073

Excited State 6: Singlet-A 4.9534 eV 250.30 nm f=0.1536 <S**2>=0.000
107 ->116 -0.10737
108 ->116 -0.10520
110 ->120 -0.10751
114 ->116 0.27632
115 ->116 0.14066
115 ->122 0.13236
115 ->123 0.20231
115 ->124 0.15342
115 ->127 0.27186
115 ->128 -0.24966
115 ->129 -0.15524

Excited State 7: Singlet-A 4.9649 eV 249.72 nm f=0.1739 <S**2>=0.000
107 ->116 -0.11078
108 ->116 -0.12196
114 ->116 0.33161
114 ->117 0.15870
115 ->116 0.34589
115 ->117 -0.14466
115 ->123 -0.12154
115 ->127 -0.18504
115 ->128 0.20449
115 ->129 0.10696

Excited State 8: Singlet-A 5.0789 eV 244.11 nm f=0.0447 <S**2>=0.000
112 ->116 0.14036
112 ->117 0.16598
113 ->116 -0.19879
113 ->117 -0.26143
114 ->117 0.28830
114 ->121 0.15252
114 ->122 -0.11339
114 ->123 -0.14411
115 ->116 -0.16567

Excited State 9: Singlet-A 5.1920 eV 238.80 nm f=0.1490 <S**2>=0.000
106 ->116 -0.15697
107 ->116 0.23542
108 ->117 -0.13644
108 ->119 -0.14633
110 ->117 -0.10411

112 ->117 -0.14883
113 ->116 0.10085
113 ->117 0.12621
114 ->117 0.33591
114 ->118 0.10832
114 ->119 -0.13132
115 ->117 -0.12032
115 ->119 -0.13521
115 ->130 0.13284

Excited State 10: Singlet-A 5.2674 eV 235.38 nm f=0.0667 <S**2>=0.000
107 ->116 0.11334
114 ->117 0.17307
115 ->117 0.11440
115 ->118 0.10140
115 ->119 0.13717
115 ->120 -0.20888
115 ->121 0.34376
115 ->122 -0.30554
115 ->124 0.15634
115 ->128 -0.11421
115 ->130 -0.15182

Excited State 11: Singlet-A 5.3344 eV 232.43 nm f=0.0214 <S**2>=0.000
107 ->116 0.10291
114 ->117 0.10374
115 ->117 0.21935
115 ->119 0.15309
115 ->120 0.16964
115 ->121 -0.16880
115 ->123 -0.11505
115 ->124 -0.22925
115 ->125 0.24012
115 ->129 -0.13745
115 ->130 -0.22219
115 ->131 0.11151

Excited State 12: Singlet-A 5.4997 eV 225.44 nm f=0.2547 <S**2>=0.000
106 ->116 -0.17697
107 ->116 0.25277
108 ->116 -0.12216
108 ->117 -0.10839
114 ->116 0.17858
114 ->117 -0.24634
114 ->119 0.25672
115 ->123 0.26371

Excited State 13: Singlet-A 5.5381 eV 223.88 nm f=0.0591 <S**2>=0.000
107 ->116 -0.11679
112 ->116 -0.10791
113 ->116 0.18562
114 ->117 0.17793
114 ->118 -0.13220
115 ->117 0.16312
115 ->118 0.14057
115 ->121 -0.16270
115 ->122 0.10565
115 ->123 0.29768
115 ->124 0.18787
115 ->126 -0.11920
115 ->130 -0.16531

Excited State 14:   Singlet-A   5.6086 eV 221.06 nm f=0.0315 <S**2>=0.000
107 ->116   0.12969
109 ->117   0.13966
114 ->117   -0.15256
114 ->118   0.25197
114 ->119   -0.17203
114 ->123   -0.13772
115 ->118   0.18519
115 ->124   0.14778
115 ->125   0.13354
115 ->130   -0.22246

Excited State 15:   Singlet-A   5.6483 eV 219.51 nm f=0.0694 <S**2>=0.000
106 ->116   -0.19533
107 ->116   -0.20650
107 ->117   0.17258
108 ->116   0.39871
110 ->116   0.13491
111 ->116   -0.11782
112 ->116   0.18599
113 ->116   -0.24126
114 ->116   0.15118
114 ->117   -0.11647

Excited State 16:   Singlet-A   5.7327 eV 216.28 nm f=0.0108 <S**2>=0.000
114 ->119   -0.11631
115 ->117   0.13442
115 ->118   0.16736
115 ->119   0.18872
115 ->122   0.14678
115 ->125   -0.23846
115 ->126   -0.14713
115 ->127   0.16837
115 ->128   0.31362
115 ->135   0.11494
115 ->136   -0.10697
115 ->137   0.13466

Excited State 17:   Singlet-A   5.7676 eV 214.97 nm f=0.0517 <S**2>=0.000
114 ->118   -0.11460
115 ->118   0.12928
115 ->119   0.12595
115 ->122   0.14335
115 ->124   0.20178
115 ->125   0.18515
115 ->126   0.36725
115 ->127   0.10607
115 ->129   0.24374
115 ->130   0.11619
115 ->131   -0.12594
115 ->137   0.14198

Excited State 18:   Singlet-A   5.8031 eV 213.65 nm f=0.0771 <S**2>=0.000
106 ->116   -0.16354
107 ->117   0.12325
108 ->116   0.17714
112 ->116   -0.18918
112 ->117   0.11475
113 ->116   0.33752
113 ->117   -0.15031

114 ->116 0.16350
115 ->125 0.22775

Excited State 19: Singlet-A 5.8124 eV 213.31 nm f=0.0571 <S**2>=0.000
113 ->116 0.13986
115 ->122 0.14461
115 ->124 -0.10958
115 ->125 -0.34982
115 ->126 0.35079
115 ->130 -0.20442
115 ->132 0.15190
115 ->137 -0.13246

Excited State 20: Singlet-A 5.8969 eV 210.25 nm f=0.0726 <S**2>=0.000
107 ->116 0.13303
111 ->117 -0.11553
114 ->118 -0.18586
115 ->119 0.10606
115 ->120 0.13406
115 ->121 -0.18905
115 ->122 -0.16568
115 ->125 -0.17159
115 ->127 -0.21177
115 ->128 -0.23423
115 ->130 0.19288
115 ->131 -0.12772
115 ->137 0.10978

Excited State 21: Singlet-A 5.9486 eV 208.43 nm f=0.1029 <S**2>=0.000
111 ->127 -0.10823
112 ->120 0.17949
112 ->121 0.11684
112 ->123 0.11914
113 ->117 -0.13677
113 ->118 0.15481
113 ->120 0.26018
113 ->121 0.17300
113 ->123 0.10525
115 ->124 -0.17177
115 ->127 0.13935
115 ->128 -0.15441

Excited State 22: Singlet-A 5.9855 eV 207.14 nm f=0.1268 <S**2>=0.000
107 ->116 -0.20054
108 ->117 -0.14666
111 ->117 0.12627
113 ->117 -0.11799
114 ->117 0.10906
114 ->118 0.32018
114 ->119 0.21875
115 ->118 0.10326
115 ->119 0.14833
115 ->121 -0.11723
115 ->130 0.12848
115 ->137 0.16553

Excited State 23: Singlet-A 6.0498 eV 204.94 nm f=0.0092 <S**2>=0.000
111 ->116 -0.10615
111 ->117 -0.15560
111 ->118 0.15177
111 ->123 0.10310

```
    112 ->117       -0.10360
    115 ->119       -0.12176
    115 ->127       -0.16090
    115 ->128        0.10410
    115 ->130       -0.23728
    115 ->133        0.13098
    115 ->137        0.24722
    115 ->138       -0.10901

 Excited State  24:      Singlet-A      6.0919 eV  203.52 nm  f=0.0626  <S**2>=0.000
    110 ->120       -0.10144
    111 ->117       -0.13063
    111 ->118        0.12367
    112 ->119        0.11198
    113 ->117       -0.19627
    114 ->121       -0.13234
    114 ->123        0.13163
    115 ->129        0.29819
    115 ->133       -0.22594
    115 ->137       -0.16115
    115 ->138        0.10431
    115 ->139       -0.10773
    115 ->140       -0.10343

 Excited State  25:      Singlet-A      6.1104 eV  202.91 nm  f=0.0285  <S**2>=0.000
    111 ->116       -0.10328
    111 ->119        0.10518
    113 ->116        0.12612
    113 ->117        0.31276
    113 ->118       -0.16001
    114 ->119        0.18885
    114 ->121        0.15498
    114 ->122       -0.10604
    114 ->123       -0.20997
    115 ->124       -0.10824
    115 ->129        0.12347
```

(BMK/6-311+G* PCM Toluene, TD=(Nstates=25,root=1))

```
Excited State   1:      Singlet-A      3.7430 eV  331.25 nm  f=0.1185  <S**2>=0.000
    115 ->116        0.52542
    115 ->117        0.40012
    115 ->118       -0.13609
    115 ->119        0.18243
 This state for optimization and/or second-order correction.
 Total Energy, E(TD-HF/TD-DFT) =  -1342.58217101
 Copying the excited state density for this state as the 1-particle RhoCI density.

 Excited State   2:      Singlet-A      4.1804 eV  296.58 nm  f=0.0519  <S**2>=0.000
    115 ->118        0.51356
    115 ->119        0.43580

 Excited State   3:      Singlet-A      4.2947 eV  288.69 nm  f=0.0417  <S**2>=0.000
    115 ->116       -0.44806
    115 ->117        0.44020
    115 ->119        0.24831
    115 ->120        0.12636

 Excited State   4:      Singlet-A      4.3898 eV  282.43 nm  f=0.3381  <S**2>=0.000
    115 ->119       -0.12019
    115 ->120        0.62631
```

115 ->121 -0.26406

Excited State 5: Singlet-A 4.5821 eV 270.59 nm f=0.5087 <S**2>=0.000
114 ->116 0.63645
115 ->122 -0.15103

Excited State 6: Singlet-A 4.6404 eV 267.18 nm f=0.1372 <S**2>=0.000
114 ->116 0.16131
115 ->117 0.24099
115 ->118 0.11913
115 ->119 -0.21271
115 ->120 -0.12905
115 ->121 -0.19753
115 ->122 0.50323

Excited State 7: Singlet-A 4.7934 eV 258.66 nm f=0.0357 <S**2>=0.000
113 ->116 0.10455
115 ->120 0.16537
115 ->121 0.44116
115 ->122 0.24852
115 ->123 -0.33113
115 ->124 0.14247

Excited State 8: Singlet-A 4.8870 eV 253.70 nm f=0.0377 <S**2>=0.000
107 ->116 -0.10394
111 ->116 -0.12261
112 ->116 0.15215
113 ->116 0.30223
113 ->117 0.17310
114 ->117 0.31605
114 ->120 0.13796
114 ->121 0.15817
115 ->118 0.13941
115 ->119 -0.14772
115 ->122 -0.10369
115 ->123 0.19357

Excited State 9: Singlet-A 4.9492 eV 250.52 nm f=0.0345 <S**2>=0.000
111 ->116 0.12311
113 ->116 -0.21488
115 ->117 0.22030
115 ->118 0.31721
115 ->119 -0.29976
115 ->121 0.15140
115 ->122 -0.17755
115 ->125 0.10215
115 ->126 -0.16453

Excited State 10: Singlet-A 5.0250 eV 246.73 nm f=0.2062 <S**2>=0.000
107 ->116 -0.21254
108 ->117 -0.13387
113 ->116 -0.18420
114 ->117 0.50681
114 ->121 -0.10085
115 ->118 -0.12188
115 ->123 -0.13116

Excited State 11: Singlet-A 5.1396 eV 241.23 nm f=0.0034 <S**2>=0.000
113 ->116 -0.17870
115 ->120 0.17342
115 ->121 0.34338

```
   115 ->122         0.19989
   115 ->123         0.39789
   115 ->124        -0.21636

Excited State  12:      Singlet-A      5.3464 eV  231.90 nm  f=0.0786  <S**2>=0.000
   106 ->116        -0.10931
   107 ->116         0.19957
   108 ->117         0.15975
   109 ->116        -0.12114
   109 ->117        -0.10429
   109 ->118         0.12846
   111 ->116         0.14472
   111 ->117         0.10512
   114 ->117         0.10104
   114 ->119         0.41163
   115 ->118        -0.13891
   115 ->121        -0.12217
   115 ->122         0.10849

Excited State  13:      Singlet-A      5.4035 eV  229.45 nm  f=0.0863  <S**2>=0.000
   106 ->116        -0.15283
   107 ->116         0.30768
   108 ->117         0.10446
   108 ->119        -0.11648
   109 ->117         0.14526
   111 ->117        -0.12477
   113 ->116        -0.20165
   114 ->117         0.23067
   114 ->118        -0.30603
   114 ->122         0.12681

Excited State  14:      Singlet-A      5.4938 eV  225.68 nm  f=0.0130  <S**2>=0.000
   107 ->116        -0.14217
   108 ->116        -0.14515
   111 ->116         0.10080
   111 ->117        -0.12579
   112 ->116        -0.13906
   112 ->117         0.13193
   113 ->116        -0.29081
   113 ->117         0.33935
   114 ->118         0.18740
   114 ->120         0.14414
   114 ->121         0.20677
   115 ->125        -0.11062
   115 ->126         0.10108

Excited State  15:      Singlet-A      5.5542 eV  223.22 nm  f=0.0132  <S**2>=0.000
   113 ->116        -0.11891
   115 ->123         0.19487
   115 ->124         0.37220
   115 ->125         0.43466
   115 ->127         0.11763

Excited State  16:      Singlet-A      5.5756 eV  222.37 nm  f=0.0458  <S**2>=0.000
   108 ->116        -0.14433
   112 ->116        -0.19115
   112 ->117        -0.12492
   113 ->116         0.18637
   114 ->116        -0.10220
   114 ->118         0.15973
   115 ->123         0.12137
```

115 ->124 0.17339
115 ->126 0.45993

Excited State 17: Singlet-A 5.5984 eV 221.46 nm f=0.0120 <S**2>=0.000
106 ->116 0.10105
107 ->117 -0.11107
108 ->116 0.22710
110 ->116 -0.14723
112 ->116 0.31550
112 ->117 0.11230
113 ->116 -0.19660
113 ->117 -0.13986
114 ->119 0.11483
115 ->125 -0.14257
115 ->126 0.32129

Excited State 18: Singlet-A 5.6824 eV 218.19 nm f=0.0687 <S**2>=0.000
106 ->116 0.14831
107 ->117 -0.17134
108 ->116 0.31770
109 ->116 0.14978
111 ->116 0.19759
112 ->116 -0.27111
113 ->116 0.15495
113 ->117 0.25207
114 ->118 -0.19295

Excited State 19: Singlet-A 5.7351 eV 216.18 nm f=0.0997 <S**2>=0.000
107 ->116 0.24069
108 ->116 0.12101
111 ->116 0.22283
112 ->116 0.19308
112 ->117 0.14685
113 ->117 0.17435
114 ->118 0.22963
114 ->119 -0.32051
114 ->120 -0.17306

Excited State 20: Singlet-A 5.7501 eV 215.62 nm f=0.1327 <S**2>=0.000
107 ->116 0.23251
108 ->116 0.21604
110 ->116 -0.11738
111 ->116 -0.27807
111 ->117 -0.17831
112 ->116 -0.18580
112 ->117 -0.13759
114 ->118 0.32264
115 ->126 -0.10559

Excited State 21: Singlet-A 5.8665 eV 211.34 nm f=0.0865 <S**2>=0.000
110 ->116 -0.12616
111 ->116 0.10239
111 ->117 0.12975
111 ->120 -0.11270
113 ->117 -0.19547
113 ->120 -0.15355
114 ->119 -0.13097
114 ->120 0.25301
114 ->121 0.17189
115 ->129 -0.17158
115 ->130 0.18830

```
   115 ->131         0.23319
   115 ->132        -0.12295

 Excited State  22:      Singlet-A      5.8789 eV  210.90 nm  f=0.0597  <S**2>=0.000
   111 ->116        -0.14382
   113 ->120         0.23667
   114 ->119         0.16181
   114 ->120        -0.13489
   115 ->124        -0.18531
   115 ->126         0.14614
   115 ->128        -0.10604
   115 ->129        -0.21201
   115 ->130         0.23350
   115 ->131         0.23161
   115 ->132        -0.11413

 Excited State  23:      Singlet-A      5.9297 eV  209.09 nm  f=0.0211  <S**2>=0.000
   110 ->116         0.15347
   111 ->116         0.13708
   112 ->116        -0.14877
   112 ->118        -0.15800
   113 ->117        -0.26872
   113 ->118         0.10981
   113 ->119         0.11924
   113 ->120         0.20534
   114 ->120         0.18159
   114 ->121         0.25399
   115 ->123         0.10730

 Excited State  24:      Singlet-A      5.9491 eV  208.41 nm  f=0.0092  <S**2>=0.000
   115 ->125        -0.12603
   115 ->127         0.61889
   115 ->130        -0.16572

 Excited State  25:      Singlet-A      5.9755 eV  207.49 nm  f=0.0365  <S**2>=0.000
   109 ->116        -0.12263
   109 ->117        -0.10732
   110 ->116         0.24725
   110 ->119         0.11624
   110 ->120        -0.15741
   111 ->117        -0.10256
   111 ->120         0.11530
   111 ->121        -0.10554
   112 ->118         0.12972
   112 ->119         0.12235
   114 ->119        -0.12653
   115 ->123        -0.16257
   115 ->125         0.11904
   115 ->126         0.12965
   115 ->128         0.23299
   115 ->129        -0.17722
```

7.6.3 Computed xyz-coordinates (S_1 geometry) of compound 7c (CAM-B3LYP/6-311+G* PCM Toluene)

```
N   -2.477516  -0.009975   0.268670
C   -3.278207  -1.133651  -0.029855
C   -1.731036   0.042875   1.457475
C   -2.560244   1.129106  -0.546325
C   -1.358978   1.881778  -0.879096
C   -1.595801   3.191359  -1.377360
```

C	-2.852200	3.649949	-1.675672
C	-3.996661	2.835665	-1.478827
C	-3.834569	1.599277	-0.902414
C	-3.959754	-1.813646	0.985213
C	-4.755373	-2.901678	0.667910
C	-4.883292	-3.320161	-0.651170
C	-4.215220	-2.634650	-1.661844
C	-3.422392	-1.542544	-1.359637
C	-1.135669	-1.112384	1.982849
C	-0.364599	-1.029056	3.122909
C	-0.161190	0.203646	3.747254
C	-0.750630	1.347072	3.229992
C	-1.536912	1.277401	2.090163
C	-0.043695	1.314862	-0.866451
C	1.115564	2.119854	-0.697464
C	2.361110	1.557482	-0.630540
C	2.575619	0.168068	-0.779973
C	1.447064	-0.649081	-1.006834
C	0.188251	-0.076251	-1.036326
C	3.951333	-0.351928	-0.734106
C	4.456344	-1.164385	-1.758996
C	5.766119	-1.606981	-1.766026
C	6.638301	-1.253806	-0.738586
C	6.174244	-0.462559	0.293701
C	4.846907	-0.019818	0.306360
C	1.551616	-2.143924	-1.188844
C	4.419404	0.725244	1.451468
N	4.126335	1.299655	2.404345
H	-0.743657	3.816020	-1.616042
H	-2.968732	4.641103	-2.100436
H	-4.984848	3.199669	-1.730615
H	-4.701010	0.997919	-0.649427
H	-3.880355	-1.472623	2.009248
H	-5.290929	-3.417673	1.456242
H	-5.508489	-4.171562	-0.893107
H	-4.309853	-2.956159	-2.692308
H	-2.904560	-1.004442	-2.143431
H	-1.251137	-2.058054	1.468973
H	0.106800	-1.921401	3.517084
H	0.462753	0.266355	4.630752
H	-0.599678	2.303643	3.715839
H	-2.026715	2.159912	1.701739
H	1.017993	3.189916	-0.557703
H	3.216315	2.206328	-0.475875
H	-0.651111	-0.729756	-1.237019
H	3.808806	-1.416123	-2.588989
H	6.118509	-2.219488	-2.588734
H	7.667032	-1.593531	-0.745206
H	6.829628	-0.187062	1.111568
H	2.323895	-2.580589	-0.554077
H	0.603270	-2.624416	-0.943926
H	1.791620	-2.418549	-2.219335

SCF Done: E(RCAM-B3LYP) = -1342.87544773 A.U. after 1 cycles
Zero-point correction= 0.467548 (Hartree/Particle)
Thermal correction to Energy= 0.495028
Thermal correction to Enthalpy= 0.495973
Thermal correction to Gibbs Free Energy= 0.408408
Sum of electronic and zero-point Energies= -1342.288940
Sum of electronic and thermal Energies= -1342.261460
Sum of electronic and thermal Enthalpies= -1342.260516

Sum of electronic and thermal Free Energies=   -1342.348080

7.6.4 Computed emission energies of compound 7c (CAM-B3LYP/6-311+G* PCM Toluene, TD=(Nstates=6,root=1))

```
 Excited State   1:      Singlet-A      3.1439 eV  394.36 nm  f=0.1529  <S**2>=0.000
    115 ->116       -0.59499
    115 ->117       -0.30037
    115 ->118        0.18582
 This state for optimization and/or second-order correction.
 Total Energy, E(TD-HF/TD-DFT) =  -1342.75969898
 Copying the excited state density for this state as the 1-particle RhoCI density.

 Excited State   2:      Singlet-A      4.0728 eV  304.42 nm  f=0.0523  <S**2>=0.000
    114 ->116        0.33541
    114 ->117        0.11254
    115 ->119       -0.51589
    115 ->120       -0.16419
    115 ->121        0.10739
    115 ->123        0.13205

 Excited State   3:      Singlet-A      4.1841 eV  296.32 nm  f=0.4217  <S**2>=0.000
    114 ->116        0.41560
    115 ->116        0.21876
    115 ->117       -0.36524
    115 ->118        0.21120
    115 ->119        0.22685

 Excited State   4:      Singlet-A      4.3378 eV  285.82 nm  f=0.1636  <S**2>=0.000
    114 ->116        0.33089
    114 ->117        0.10319
    115 ->116       -0.17838
    115 ->117        0.21980
    115 ->119        0.12782
    115 ->120        0.12349
    115 ->121       -0.46652

 Excited State   5:      Singlet-A      4.4039 eV  281.53 nm  f=0.2690  <S**2>=0.000
    114 ->116       -0.20032
    114 ->117       -0.10491
    115 ->116        0.14703
    115 ->117       -0.21171
    115 ->119       -0.16924
    115 ->121       -0.36644
    115 ->122       -0.15324
    115 ->123        0.38030

 Excited State   6:      Singlet-A      4.5800 eV  270.71 nm  f=0.0283  <S**2>=0.000
    110 ->116       -0.15186
    111 ->116       -0.12521
    112 ->116        0.15588
    113 ->116       -0.18400
    114 ->119       -0.19483
    115 ->116        0.11803
    115 ->117       -0.23201
    115 ->119       -0.13995
    115 ->121       -0.20529
    115 ->122        0.18592
    115 ->123       -0.28194
    115 ->125       -0.11068
```

7.7 Quantum chemical calculation data of 6-(2′-(diphenylamino)-[1,1′-biphenyl]-4-yl)picolinonitrile (6b) in the gas phase

7.7.1 Computed xyz-coordinates (S_0 geometry) of compound 6b (CAM-B3LYP/6-311+G*) in the gas phase

N	-2.567528	-0.067322	0.247035
C	-3.402585	-0.962409	-0.455834
C	-1.900920	-0.456943	1.436017
C	-2.592775	1.316294	-0.096307
C	-1.407873	2.010652	-0.381169
C	-1.489605	3.379657	-0.644031
C	-2.704759	4.044766	-0.660651
C	-3.874577	3.342703	-0.402870
C	-3.812472	1.990120	-0.114041
C	-4.042785	-2.024375	0.187997
C	-4.855958	-2.891307	-0.525890
C	-5.062904	-2.714406	-1.887319
C	-4.440597	-1.651867	-2.528543
C	-3.615080	-0.787608	-1.826451
C	-1.233209	-1.680694	1.505679
C	-0.575426	-2.054876	2.665388
C	-0.553624	-1.211484	3.770006
C	-1.204950	0.010785	3.699503
C	-1.880298	0.385521	2.546060
C	-0.079649	1.347129	-0.428021
C	0.995917	1.867731	0.290249
C	2.251285	1.288428	0.219283
C	2.469708	0.161783	-0.570932
C	1.397286	-0.355165	-1.298263
C	0.143444	0.226577	-1.226605
C	3.818256	-0.452186	-0.625563
C	4.008448	-1.795443	-0.972545
C	5.288114	-2.314082	-1.013717
C	6.361190	-1.494633	-0.698022
C	6.076398	-0.181940	-0.345041
N	4.849663	0.330468	-0.308532
C	7.157392	0.707007	0.009398
N	8.040720	1.384979	0.287010
H	-0.577934	3.920287	-0.872977
H	-2.738612	5.105101	-0.883695
H	-4.832982	3.849565	-0.412295
H	-4.716314	1.435238	0.109989
H	-3.905319	-2.168351	1.252073
H	-5.344068	-3.707440	-0.004116
H	-5.703751	-3.391861	-2.439546
H	-4.591199	-1.494148	-3.591046
H	-3.140640	0.039617	-2.340812
H	-1.231094	-2.335924	0.643136
H	-0.061646	-3.009463	2.701197
H	-0.030669	-1.503941	4.673244
H	-1.199045	0.680343	4.552667
H	-2.393467	1.338761	2.504864
H	0.843189	2.731250	0.928100
H	3.077371	1.703722	0.782016
H	1.538482	-1.205120	-1.956093
H	-0.669147	-0.188281	-1.810730
H	3.158688	-2.429758	-1.187256
H	5.451492	-3.352043	-1.279780
H	7.382319	-1.852828	-0.714110

SCF Done: E(RCAM-B3LYP) = -1319.63364821 A.U. after 1 cycles

Zero-point correction= 0.430480 (Hartree/Particle)
Thermal correction to Energy= 0.456146
Thermal correction to Enthalpy= 0.457090
Thermal correction to Gibbs Free Energy= 0.371151
Sum of electronic and zero-point Energies= -1319.203168
Sum of electronic and thermal Energies= -1319.177502
Sum of electronic and thermal Enthalpies= -1319.176558
Sum of electronic and thermal Free Energies= -1319.262497

7.7.2 Computed molecule volume of compound 6b (CAM-B3LYP/6-311+G*, volume)

gas phase
SCF Done: E(RCAM-B3LYP) = -1319.63368152 A.U. after 7 cycles
Recommended a0 for SCRF calculation = 6.05 angstrom (11.43 bohr)

cyclohexane
SCF Done: E(RCAM-B3LYP) = -1319.63435655 A.U. after 13 cycles
Recommended a0 for SCRF calculation = 5.71 angstrom (10.78 bohr)

toluene
SCF Done: E(RCAM-B3LYP) = -1319.63450695 A.U. after 13 cycles
Recommended a0 for SCRF calculation = 6.02 angstrom (11.39 bohr)

ethyl acetate
SCF Done: E(RCAM-B3LYP) = -1319.63517452 A.U. after 14 cycles
Recommended a0 for SCRF calculation = 5.68 angstrom (10.74 bohr)

THF
SCF Done: E(RCAM-B3LYP) = -1319.63528283 A.U. after 14 cycles
Recommended a0 for SCRF calculation = 6.11 angstrom (11.54 bohr)

dichloromethane
SCF Done: E(RCAM-B3LYP) = -1319.63536282 A.U. after 14 cycles
Recommended a0 for SCRF calculation = 6.19 angstrom (11.70 bohr)

acetonitrile
SCF Done: E(RCAM-B3LYP) = -1319.63569259 A.U. after 14 cycles
Recommended a0 for SCRF calculation = 6.11 angstrom (11.54 bohr)

7.8 Quantum chemical calculation data of rotamer analysis of compound 7b

7.8.1 Rotation acceptor unit (BMK/6-31+G*)

D(22,23,26,31)

19 -1303.19754987 20.3191

25 -1303.1994982 30.3191

35 -1303.20049694 40.319

44 -1303.20053085 50.3189

55 -1303.19968987 60.3189

68 -1303.19844987 70.3188

115 -1303.19823286 80.3189

128 -1303.19812375 90.319

140 -1303.1989795 100.3191

152 -1303.20032625 110.3191

160 -1303.20139572 120.3191

171 -1303.2018144 130.319

181 -1303.20122848 140.3188

196 -1303.19986603 150.3188

203 -1303.19787068 160.3188

526 -1303.19324907 -179.6811

536 -1303.19517029 -169.6809

565 -1303.19782408 -159.6809

669 -1303.19979799 -149.6809

676 -1303.20083115 -139.681

700 -1303.20093861 -129.681

713 -1303.20030243 -119.6811

918 -1303.19933302 -109.6811

930 -1303.19851849 -99.6811

944 -1303.1982326 -89.681

956 -1303.19859693 -79.6809

963 -1303.19951218 -69.6809

971 -1303.20049202 -59.6809

978 -1303.20112675 -49.681

984 -1303.2009106 -39.6811

991 -1303.19974795 -29.6812

1000 -1303.19765468 -19.6811

1038 -1303.1951305 -9.6811

1095 -1303.19352389 0.319

1102 -1303.19511321 10.3191

1109 -1303.19754955 20.319

7.8.2 Rotation bridged acceptor unit (BMK/6-31+G*)

D(6,5,20,21)

18 -1303.19999145 33.9681

29 -1303.20063377 43.968 rotamer 1

37 -1303.20032366 53.9679

75 -1303.20043021 63.9679

83 -1303.19993074 73.9679

93 -1303.19896176 83.9678

112 -1303.19802919 93.9679

122 -1303.19781022 103.9679

135 -1303.19739604 113.9679

148 -1303.19660994 123.9678

166 -1303.19695078 133.9679

174 -1303.19627004 143.9679

185 -1303.19477622 153.9679

194 -1303.19259713 163.9679

218 -1303.1908241 173.9678 transitional state (TS)

234 -1303.191436 -176.0319

242 -1303.19446544 -166.032

253 -1303.19756968 -156.0319

258 -1303.19976305 -146.032

268 -1303.2008351 -136.032 rotamer 2

599 -1303.20049022 -116.032

621 -1303.19964633 -106.0322

634 -1303.198659 -96.0321

645 -1303.19781283 -86.0321

668 -1303.19746696 -76.032

678 -1303.1975595 -66.032

708 -1303.19776099 -56.032

723 -1303.19780823 -46.0321

731 -1303.19715319 -36.0322

738 -1303.1955383 -26.0321

745 -1303.19316149 -16.0322

764 -1303.19105775 -6.0321

775 -1303.19229467 3.9681

797 -1303.19546173 13.968

810 -1303.19820939 23.9681

820 -1303.19999144 33.9681

7.8.3 Computed xyz-coordinates (S_0 geometry) of bridged acceptor unit rotamer 1 (BMK/6-31+G* PCM Toluene)

N	-2.436097	-0.088213	0.301216
C	-3.179322	-1.186414	-0.182587
C	-1.720351	-0.146263	1.523073
C	-2.539097	1.164732	-0.373691
C	-1.389561	1.834065	-0.860972
C	-1.563029	3.100172	-1.458507
C	-2.827672	3.678842	-1.603132
C	-3.960420	2.993622	-1.140866
C	-3.809444	1.749387	-0.523150
C	-3.756029	-2.130163	0.694012
C	-4.486782	-3.210751	0.188654
C	-4.674889	-3.369731	-1.192312
C	-4.116996	-2.425548	-2.065232
C	-3.372652	-1.347902	-1.571640
C	-0.987570	-1.298689	1.872081
C	-0.276023	-1.345139	3.074361
C	-0.261415	-0.241100	3.940345
C	-0.973090	0.911772	3.586030
C	-1.704176	0.961007	2.391886
C	-0.014242	1.251965	-0.790776
C	1.074855	2.052863	-0.399447
C	2.379908	1.549968	-0.396851
C	2.637472	0.224212	-0.789785
C	1.547372	-0.581074	-1.178005
C	0.244723	-0.079036	-1.173568
C	4.023607	-0.325528	-0.844716
C	4.429489	-1.076859	-1.965011
C	5.719305	-1.608274	-2.064855
C	6.651951	-1.401508	-1.038274
C	6.279632	-0.665518	0.087432
C	4.977210	-0.134031	0.188109
C	4.655588	0.577762	1.404538
N	4.465844	1.134431	2.403780
H	-0.687269	3.617431	-1.847526
H	-2.928057	4.650856	-2.082709
H	-4.952276	3.429709	-1.246766
H	-4.675658	1.213573	-0.138165
H	-3.633366	-2.012561	1.767902
H	-4.923270	-3.925268	0.885180
H	-5.248540	-4.209678	-1.579132
H	-4.252663	-2.526953	-3.141084
H	-2.949361	-0.620143	-2.261331
H	-0.976150	-2.151364	1.196431
H	0.286784	-2.243244	3.324600
H	0.306063	-0.276077	4.868173
H	-0.968948	1.780469	4.242653
H	-2.259342	1.858903	2.128521
H	0.900462	3.080632	-0.084798
H	3.199639	2.201957	-0.102138
H	1.719770	-1.615547	-1.471680
H	-0.577051	-0.725180	-1.476150
H	3.722380	-1.223375	-2.779272
H	5.998879	-2.176841	-2.950054
H	7.657537	-1.809827	-1.112423
H	6.982098	-0.502053	0.901929

SCF Done: E(RBMK) = -1303.20772464 A.U. after 1 cycles
Zero-point correction= 0.441556 (Hartree/Particle)
Thermal correction to Energy= 0.467241
Thermal correction to Enthalpy= 0.468186
Thermal correction to Gibbs Free Energy= 0.383647

Sum of electronic and zero-point Energies= -1302.766168
Sum of electronic and thermal Energies= -1302.740483
Sum of electronic and thermal Enthalpies= -1302.739539
Sum of electronic and thermal Free Energies= -1302.824078

7.8.4 Computed xyz-coordinates (S_0 geometry) of bridged acceptor unit rotamer 2 (BMK/6-31+G* PCM Toluene)

N	2.421455	-0.078441	-0.306549
C	3.056917	-1.259590	0.137070
C	1.814532	0.010156	-1.582696
C	2.534435	1.104330	0.483447
C	1.383154	1.805964	0.916938
C	1.565453	3.006062	1.634192
C	2.840703	3.486587	1.948435
C	3.972748	2.767136	1.539389
C	3.814934	1.589378	0.803721
C	3.666773	-2.151544	-0.769978
C	4.289798	-3.315272	-0.306193
C	4.335063	-3.609146	1.064614
C	3.743989	-2.716773	1.969640
C	3.106494	-1.556448	1.515730
C	1.050861	-1.060188	-2.091632
C	0.449019	-0.962445	-3.349562
C	0.576355	0.206911	-4.115524
C	1.318642	1.278738	-3.603187
C	1.939178	1.183587	-2.350784
C	-0.006025	1.317299	0.660659
C	-0.383247	-0.008332	0.949985
C	-1.700725	-0.434481	0.769550
C	-2.682431	0.448312	0.279211
C	-2.304786	1.769285	-0.024752
C	-0.987985	2.197719	0.170111
C	-4.086268	0.000931	0.045035
C	-4.724377	0.306193	-1.172198
C	-6.037882	-0.099479	-1.431611
C	-6.756476	-0.829346	-0.473682
C	-6.153612	-1.141645	0.745979
C	-4.831142	-0.728358	1.006661
C	-4.286256	-1.037979	2.309372
N	-3.905661	-1.305986	3.371383
H	0.684768	3.545070	1.980533
H	2.948658	4.407412	2.518738
H	4.972091	3.126721	1.778529
H	4.683910	1.030939	0.459281
H	3.653659	-1.928828	-1.834248
H	4.754643	-3.988655	-1.024984
H	4.824763	-4.513618	1.420083
H	3.768309	-2.924042	3.038468
H	2.654788	-0.870950	2.230206
H	0.930997	-1.962899	-1.496305
H	-0.136360	-1.800734	-3.724671
H	0.101229	0.280805	-5.091972
H	1.428852	2.194655	-4.182059
H	2.520588	2.018377	-1.964941
H	0.356829	-0.712088	1.326152
H	-1.962879	-1.465649	0.999164
H	-3.048849	2.470865	-0.399551
H	-0.716644	3.224262	-0.072395

H	-4.166228	0.851501	-1.930976
H	-6.497363	0.147025	-2.387190
H	-7.776798	-1.149869	-0.672801
H	-6.696545	-1.694669	1.509464

SCF Done: E(RBMK) = -1303.20827567 A.U. after 2 cycles
Zero-point correction= 0.440950 (Hartree/Particle)
Thermal correction to Energy= 0.466767
Thermal correction to Enthalpy= 0.467712
Thermal correction to Gibbs Free Energy= 0.382750
Sum of electronic and zero-point Energies= -1302.767326
Sum of electronic and thermal Energies= -1302.741508
Sum of electronic and thermal Enthalpies= -1302.740564
Sum of electronic and thermal Free Energies= -1302.825526

7.8.4 Computed xyz-coordinates (S_0 geometry) of bridged acceptor unit transitional state (TS) (BMK/6-31+G* PCM Toluene)

N	2.387973	0.090536	0.165418
C	2.785810	1.277060	-0.508511
C	2.356253	0.001024	1.571894
C	2.389371	-1.119102	-0.606965
C	1.211916	-1.845598	-0.945885
C	1.426272	-3.070407	-1.629476
C	2.689714	-3.529556	-2.002944
C	3.827031	-2.769182	-1.705969
C	3.659612	-1.577543	-1.003396
C	3.802974	2.110888	-0.004733
C	4.183009	3.259809	-0.708469
C	3.570756	3.586207	-1.927403
C	2.567971	2.748841	-2.436347
C	2.172417	1.605826	-1.732199
C	2.012917	1.130148	2.351504
C	1.944213	1.037806	3.744148
C	2.193313	-0.178686	4.398090
C	2.510504	-1.304151	3.628374
C	2.594535	-1.222939	2.231876
C	-0.206227	-1.398978	-0.676414
C	-0.543902	-0.229966	0.044211
C	-1.872026	0.159805	0.246472
C	-2.940689	-0.597947	-0.255722
C	-2.623822	-1.763333	-0.977695
C	-1.298549	-2.144516	-1.187157
C	-4.363778	-0.223782	-0.016685
C	-5.280872	-1.204722	0.406574
C	-6.623795	-0.896084	0.647513
C	-7.092133	0.413776	0.470651
C	-6.209155	1.407148	0.044059
C	-4.856866	1.093121	-0.201730
C	-4.011881	2.157523	-0.694476
N	-3.387665	3.047956	-1.097009
H	0.583126	-3.700622	-1.887105
H	2.778693	-4.478502	-2.528873
H	4.820622	-3.104979	-1.996749
H	4.521283	-0.970853	-0.728390
H	4.293728	1.857370	0.932551
H	4.971274	3.894048	-0.305479
H	3.871563	4.478916	-2.472490
H	2.079501	2.989306	-3.379226
H	1.385916	0.963177	-2.124426
H	1.799296	2.078163	1.862799
H	1.679847	1.924267	4.319101
H	2.133779	-0.246678	5.482506
H	2.701950	-2.261134	4.111884
H	2.844302	-2.110253	1.656404
H	0.225444	0.399162	0.464971
H	-2.064848	1.060581	0.825613
H	-3.421700	-2.367970	-1.406816
H	-1.141899	-3.037131	-1.781899
H	-4.921792	-2.219309	0.569067
H	-7.303146	-1.677985	0.982281
H	-8.134920	0.659056	0.660008
H	-6.553522	2.426985	-0.113733

SCF Done: E(RBMK) = -1303.19809657 A.U. after 2 cycles

Zero-point correction= 0.441549 (Hartree/Particle)
Thermal correction to Energy= 0.466510
Thermal correction to Enthalpy= 0.467455
Thermal correction to Gibbs Free Energy= 0.385225
Sum of electronic and zero-point Energies= -1302.756547
Sum of electronic and thermal Energies= -1302.731586
Sum of electronic and thermal Enthalpies= -1302.730642
Sum of electronic and thermal Free Energies= -1302.812872

7.9 Calculated absorption spectra and transitions in comparison to the experimental data

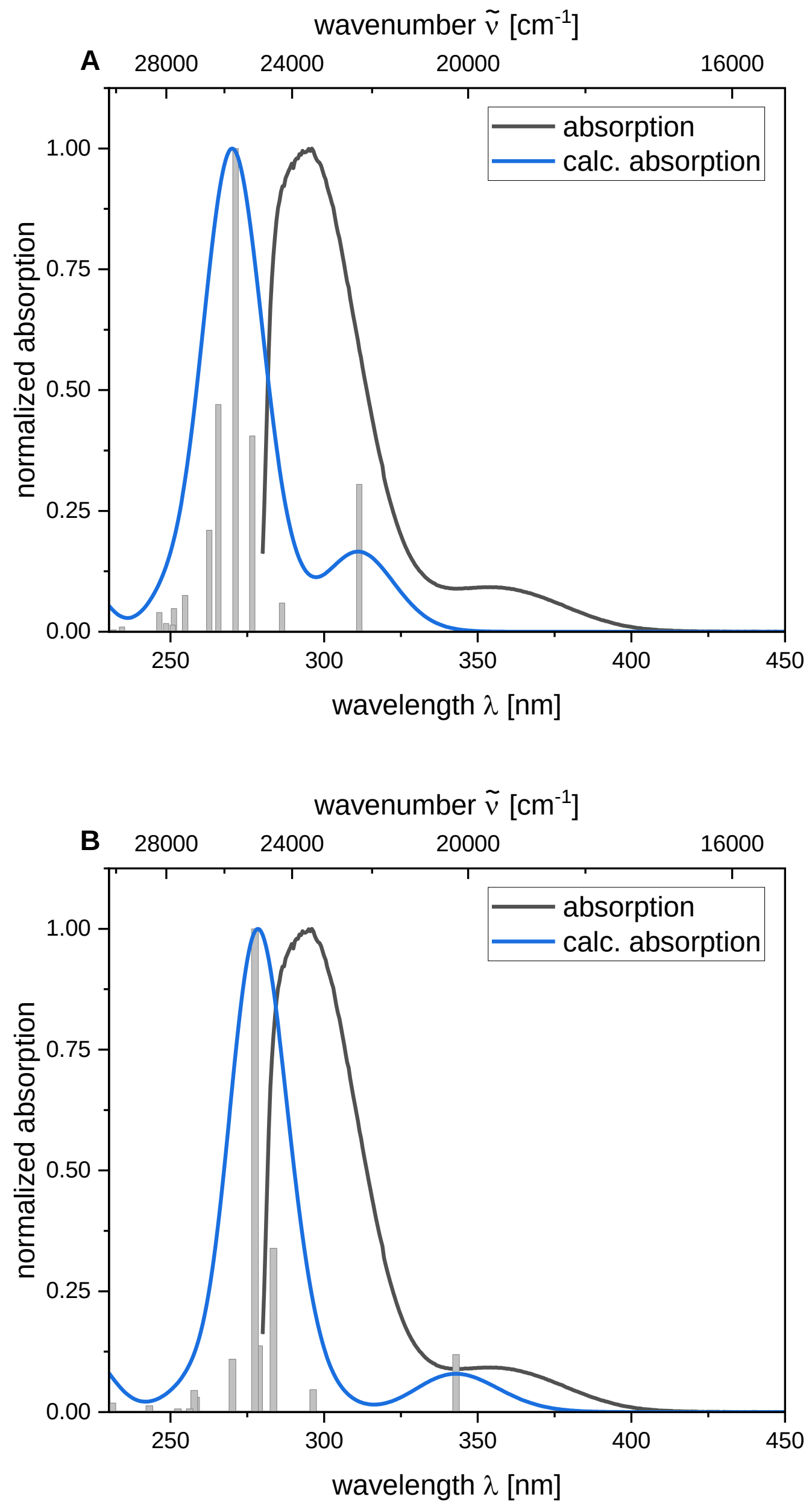

Figure S84. Normalized to intensity maximum: experimental absorption spectra recorded in toluene (black line, T = 298 K, c_{abs}(**6b**) = 10^{-5} M, c_{em}(**6b**) = 10^{-6} M, $\lambda_{exc} = \lambda_{max}$) and TD-DFT-calculated absorption spectrum of compound **6b** (blue line) Gaussian 16, cam-B3LYP/6-311+G*, PCM toluene (**A**) and Gaussian 16, BMK/6-311+G*, PCM toluene (**B**). Calculated transitions are shown in grey.

Table S11. TD-DFT calculations on the absorption maxima (Gaussian 16, different functionals/6-311+G*, PCM toluene) of compound **6b** in comparison with the experimental data (T = 293 K, c_{abs} = 10^{-5} M, c_{em} = 10^{-6} M, λ_{exc} = $\lambda_{max,abs}$).

entry	comp-ound	*functional*	$\lambda_{abs,exp}$ [nm] (ε [L mol^{-1} cm^{-1}])	$\lambda_{abs,calcd}$ [nm] (oscillator strength), most dominant contributions	$\lambda_{em,exp}$ [nm]	$\lambda_{em,calcd}$ [nm] (oscillator strength), most dominant contributions
**1**	**6b**	ωB97X-D	295 (39200)	265 (0.6529) HOMO-1→LUMO+1 (64%)<br>275 (0.2938) HOMO→LUMO+4 (81%)	448	nd[a]
			353 (3900)	298 (0.1665) HOMO→LUMO+1 (50%)		
**2**	**6b**	cam-B3LYP	295 (39200)	266 (0.3199) HOMO-1→LUMO (29%) HOMO→LUMO (28%)<br>271 (0.6804) HOMO-1→LUMO+1 (55%)<br>276 (0.2758) HOMO→LUMO+4 (78%)	448	421 (0.2214) HOMO→LUMO (85%)
			353 (3900)	311 (0.2074) HOMO→LUMO+1 (55%)		
**3**	**6b**	LC-ωHPBE	295 (39200)	250 (0.7410) HOMO-1→LUMO+1 (52%)<br>258 (0.2933) HOMO→LUMO+5 (66%)	448	nd[a]
			353 (3900)	274 (0.3331) HOMO→LUMO (19%) HOMO→LUMO+1 (19%) HOMO→LUMO+2 (21%)		
**4**	**6b**	BMK	295 (39200)	278 (0.9688) HOMO-1→LUMO+1 (70%)<br>284 (0.3284) HOMO→LUMO+4 (83%)	448	483 (2.57) (0.0951) HOMO→LUMO (93%)
			353 (3900)	343 (0.1152) HOMO→LUMO (43%) HOMO→LUMO+1 (44%)		
**5**	**6b**	M062X	295 (39200)	269 (0.2730) HOMO-1→LUMO+1 (16%) HOMO→LUMO+4 (38%)<br>268 (0.6396) HOMO-1→LUMO+1 (58%)	448	nd[a]
			353 (3900)	318 (0.1186) HOMO→LUMO (28%) HOMO→LUMO+1 (47%)		

[a] not determined.

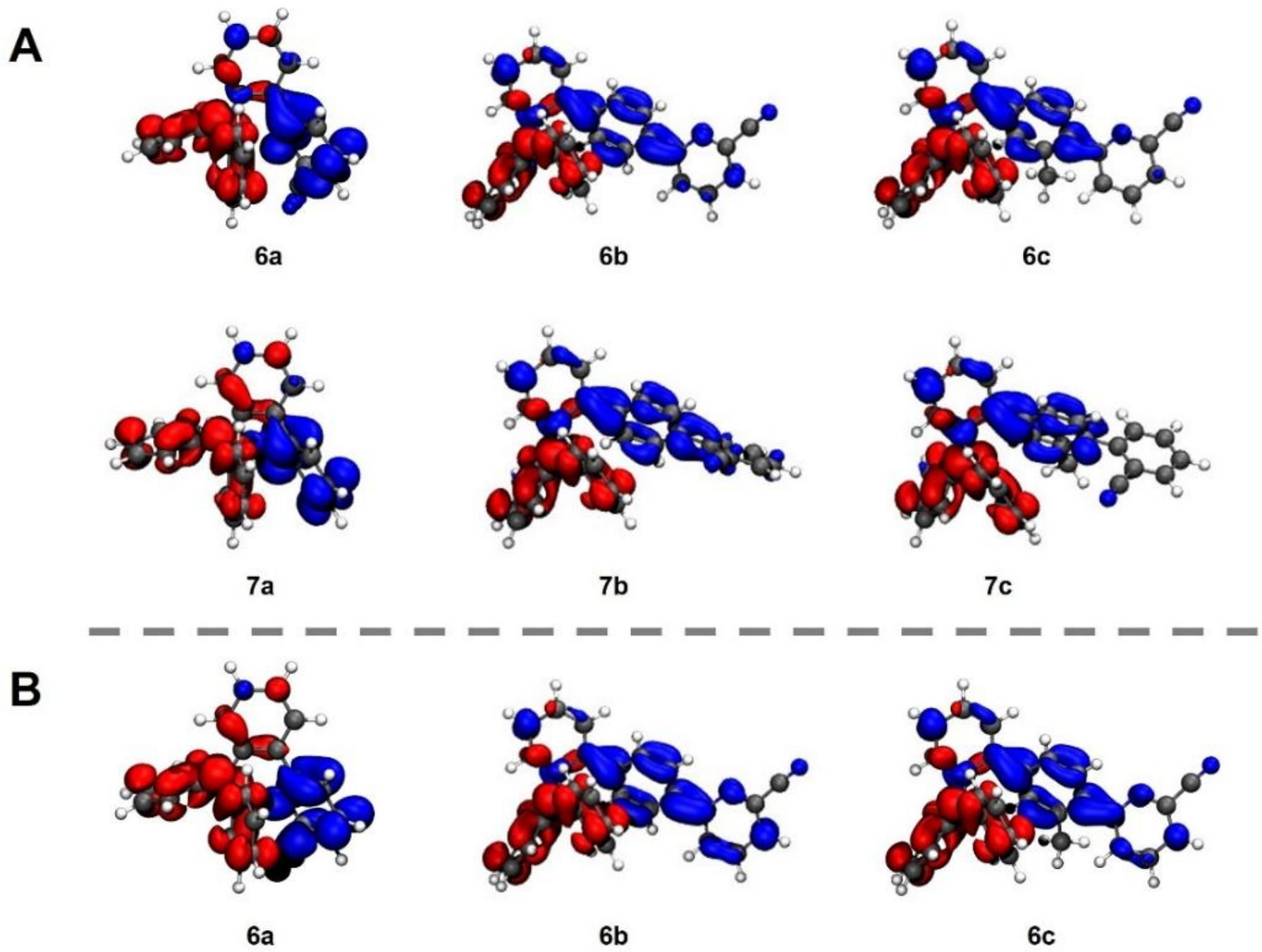

Figure S85. Charge density difference plots upon photoexcitation (longest wavelength absorption) of cyanopyridine-based **6** and benzonitrile-based donor–acceptor conjugates **7** (Gaussian 16, cam-B3LYP/6-311+G*, PCM toluene, isosurface value at 0.0015) (**A**) and exemplified for cyanopyridine-based donor–acceptor conjugates **6** (Gaussian 16, BMK/6-311+G*, PCM toluene, isosurface value at 0.0015) (**B**), (blue indicates electron accumulation and red electron depletion) determined by Multiwfn[13].

Table S12. TD-DFT calculations on the absorption and emission maxima (Gaussian 16, cam-B3LYP/6-311+G*, PCM toluene) of compounds **6** and **7** in comparison with the experimental data (T = 293 K, $c_{abs} = 10^{-5}$ M, $c_{em} = 10^{-6}$ M, $\lambda_{exc} = \lambda_{max,abs}$).

entry	comp-ound	$\lambda_{abs,exp}$ [nm] ([eV]) (ε [L mol^{-1} cm^{-1}])	$\lambda_{abs,calcd}$ [nm] ([eV]) (oscillator strength), most dominant contributions	$\lambda_{em,exp}$ [nm] ([ev])	$\lambda_{em,calcd}$ [nm] ([eV]) (oscillator strength), most dominant contributions S_r index Δr index [Å]
**1**	**6a**	291 (22500)	274 (0.2907) HOMO→LUMO+4 (67%) 287 (0.1701) HOMO→LUMO+2 (88%)	457 (2.71) 469sh	462 (2.68) (0.0438) HOMO→LUMO (94%) S_r = 0.38 Δr = 2.67
		351sh (3.53) (3800)	315 (0.0840) HOMO→LUMO (72%) 329 (3.77) (0.0290) HOMO→LUMO+1 (69%)		
**2**	**6b**	295 (39200)	266 (0.3199) HOMO-1→LUMO (29%) HOMO→LUMO (28%)	448 (2.77)	421 (2.94) (0.2214) HOMO→LUMO (85%) S_r = 0.54 Δr = 2.50
			271 (0.6804) HOMO-1→LUMO+1 (55%)		
			276 (0.2758) HOMO→LUMO+4 (78%)		
		353 (3.51) (3900)	311 (3.99) (0.2074) HOMO→LUMO+1 (55%)		

**3**	**6c**	293 (43100)	277 (0.3353)<br>HOMO→LUMO+4 (76%)	442<br>(2.81)	408 (3.04) (0.2179)<br>HOMO→LUMO (83%)<br>S_r = 0.57<br>Δr = 2.17
			265 (0.3356)<br>HOMO-1→LUMO+1 (27%)<br>HOMO→LUMO (23%)		
			258 (0.4206)<br>HOMO-1→LUMO+1 (47%)<br>HOMO→LUMO (21%)		
		347sh (3.57)<br>(4800)	305 (4.07) (0.1868)<br>HOMO→LUMO+1 (56%)		
**4**	**7a**	294 (17100)	271 (0.2221)<br>HOMO→LUMO+1 (28%)<br>HOMO→LUMO+2 (20%)<br>HOMO→LUMO+3 (14%)	434<br>(2.86)	429 (2.89) (0.0330)<br>HOMO→LUMO (95%)<br>S_r = 0.36<br>Δr = 2.75
			277 (0.1733)<br>HOMO→LUMO+3 (65%)		
			282 (0.1949)<br>HOMO→LUMO+1 (37%)<br>HOMO→LUMO+2 (40%)		
		349sh (3.55)<br>(3200)	304 (4.08) (0.0295)<br>HOMO→LUMO (84%)		
**5**	**7b**	284sh<br>(25400)	260 (0.1594)<br>HOMO-1→LUMO (24%)<br>HOMO→LUMO (11%)<br>HOMO→LUMO+8 (18%)	441<br>(2.81)	407 (3.05) (0.1881)<br>HOMO→LUMO (78%)<br>S_r = 0.31<br>Δr = 5.76
		296 (29200)	265 (0.4401)<br>HOMO-1→LUMO (47%)<br>HOMO→LUMO+3 (11%)		
			275 (0.3071)<br>HOMO→LUMO+4 (54%)		
		345sh (3.59)<br>(3700)	305 (4.07) (0.1853)<br>HOMO→LUMO (44%)<br>HOMO→LUMO+1 (29%)		
**6**	**7c**	297 (29900)	275 (0.3257)<br>HOMO→LUMO+4 (61%)	424<br>(2.92)	394 (3.15) (0.1529)<br>HOMO→LUMO (71%)<br>S_r = 0.57<br>Δr = 1.94
		348sh (3.56)<br>(4100)	301 (4.12) (0.1715)<br>HOMO→LUMO+1 (54%)		

[a] density-difference-based CT distance [b] density-difference-based overlap integral

Table S13. TD-DFT calculations on the absorption maxima (Gaussian 16, BMK/6-311+G*, PCM toluene) of compounds **6** and **7** in comparison with the experimental data (T = 293 K, c_{abs} = 10^{-5} M, λ_{exc} = $\lambda_{max,abs}$).

entry	compound	$\lambda_{abs,exp}$ [nm]<br>(ε [L mol^{-1} cm^{-1}])	$\lambda_{abs,calcd}$ [nm]<br>(oscillator strength), most dominant contributions
**1**	**6a**	291 (22500)	281 (0.3000)<br>HOMO→LUMO+3 (89%)<br>297 (0.1674)<br>HOMO→LUMO+2 (94%)
		351sh (3800)	346 (0.0783)<br>HOMO→LUMO+1 (67%)<br>357 (0.0041)<br>HOMO→LUMO (69%)
**2**	**6b**		278 (0.9688)

		295 (39200)	HOMO-1→LUMO+1 (70%)
			284 (0.3284)<br>HOMO→LUMO+4 (83%)
		353 (3900)	343 (0.1152)<br>HOMO→LUMO (43%)<br>HOMO→LUMO+1 (44%)
**3**	**6c**	293 (43100)	266 (0.1924)<br>HOMO-1→LUMO (78%)
			271 (0.7292)<br>HOMO-1→LUMO+1 (82%)
			284 (0.3504)<br>HOMO→LUMO+4 (90%)
		347sh (4800)	333 (0.1191)<br>HOMO→LUMO+1 (74%)
**4**	**7a**	294 (17100)	285 (0.1029)<br>HOMO→LUMO+3 (74%)
			288 (0.2342)<br>HOMO→LUMO+1 (50%)
			294 (0.1855)<br>HOMO→LUMO+2 (71%)
		349sh (3200)	335 (0.0148)<br>HOMO→LUMO (94%)
**5**	**7b**	284sh (25400)	275 (0.6952)<br>HOMO-1→LUMO (86%)
		296 (29200)	283 (0.3250)<br>HOMO→LUMO+4 (66%)
		345sh (3700)	337 (0.1143)<br>HOMO→LUMO (72%)
**6**	**7c**	297 (29900)	271 (0.5087)<br>HOMO-1→LUMO (81%)
			282 (0.3381)<br>HOMO→LUMO+4 (79%)
		348sh (4100)	331 (0.1185)<br>HOMO→LUMO (55%)<br>HOMO→LUMO+1 (32%)

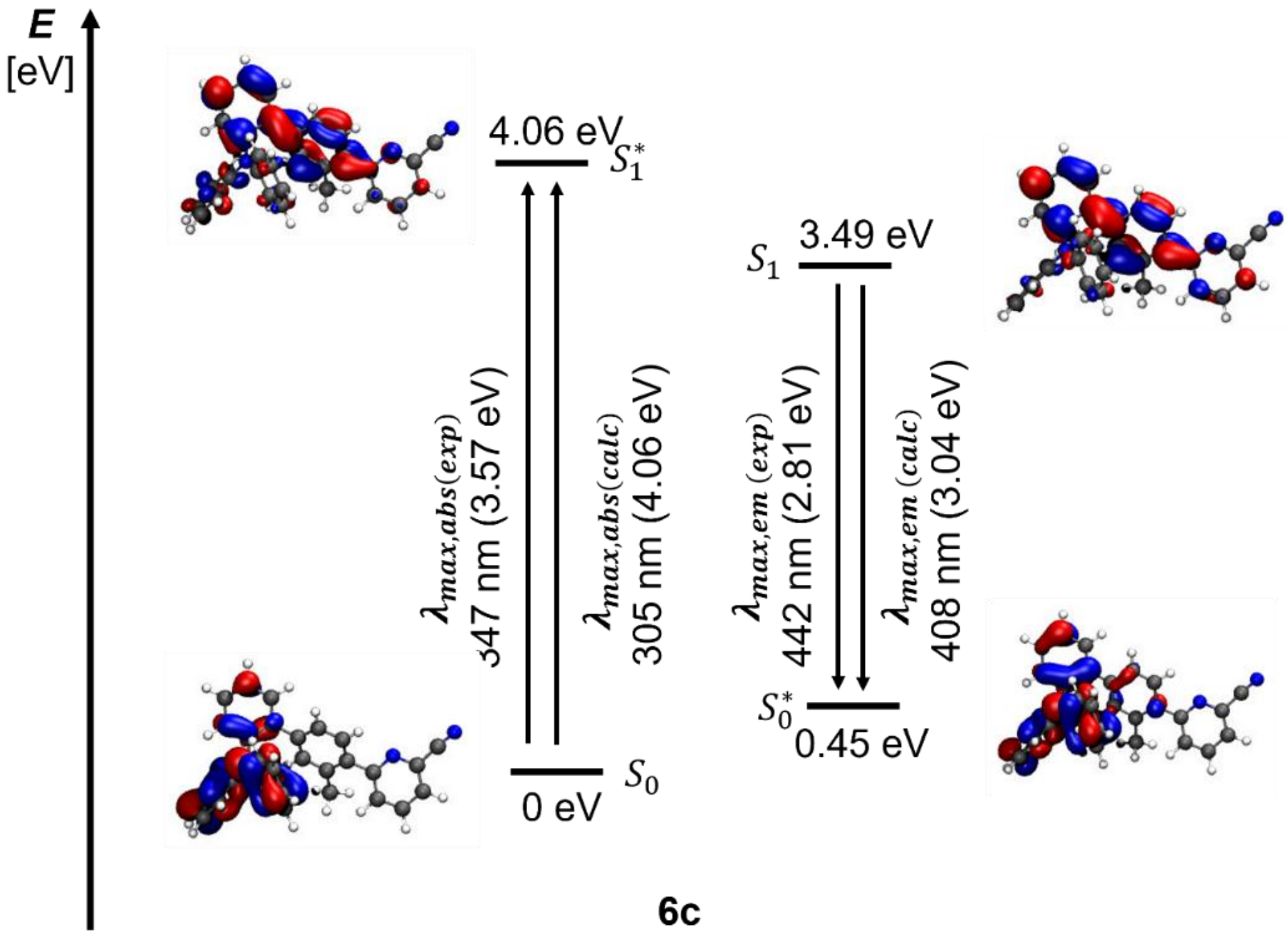

Figure S86. TD-DFT-calculated Jablonski diagram of compound **6c** including natural transition orbitals (NTOs) of the longest wavelength absorption and emission transition. (Gaussian 16, cam-B3LYP/6-311+G*, PCM toluene, isosurface value at 0.03).

Table S14. TD-DFT calculated bond length (a, b, c) and dihedra angles (α, β, γ) (Gaussian 16, cam-B3LYP/6-311+G* and BMK/6-311+G*, PCM toluene) of compounds **6** and **7**.

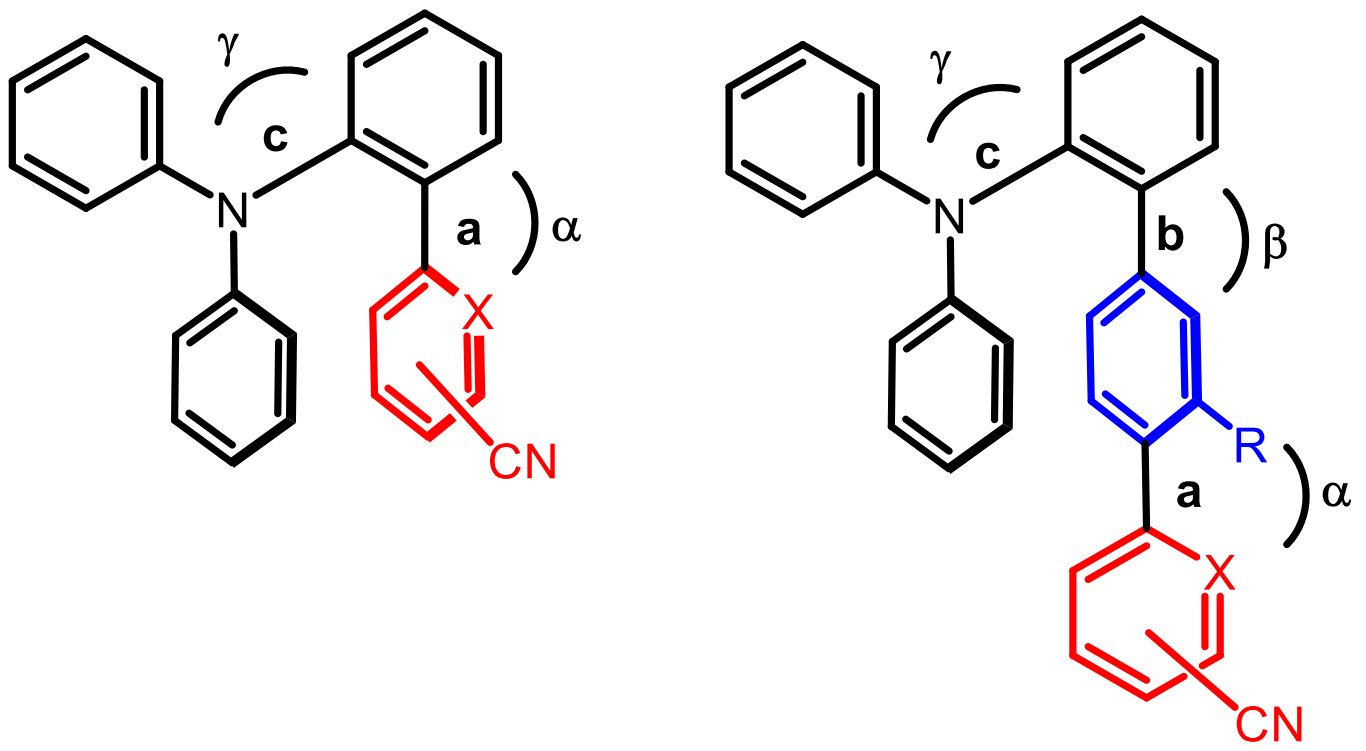

**entry**	**compound**	**functional**	**transition $\lambda_{max,exp}$ [nm] (Φ_{PL}) (calculated *f*)**	**calculated bond length a, b, c [Å]**	**calculated dihedra angles α, β, γ [°]**
**1**	**6a**	cam-B3LYP	$S_0 \rightarrow S_1$ (absorption)	a = 1.487<br>c = 1.420	α = -123.9<br>γ = 54.0
			$S_1 \rightarrow S_0$ (emission) 457 469sh (0.18) (0.0438)	a = 1.433<br>c = 1.417	α = 20.0<br>γ = 56.8
		BMK	$S_0 \rightarrow S_1$ (absorption)	a = 1.494<br>c = 1.419	α = -124.1<br>γ = 55.9
**2**	**6b**	cam-B3LYP	$S_0 \rightarrow S_1$ (absorption)	a = 1.483<br>b = 1.486<br>c = 1.427	α = -24.7<br>β = 51.3<br>γ = 54.0
			$S_1 \rightarrow S_0$ (emission) 448 (0.29) (0.2214)	a = 1.449<br>b = 1.429<br>c = 1.404	α = -0.44<br>β = 30.2<br>γ = 45.9
		BMK	$S_0 \rightarrow S_1$ (absorption)	a = 1.491<br>b = 1.493<br>c = 1.425	α = -24.2<br>β = 49.0<br>γ = 56.4
**3**	**6c**	cam-B3LYP	$S_0 \rightarrow S_1$ (absorption)	a = 1.487<br>b = 1.486<br>c = 1.427	α = -44.5<br>β = 49.8<br>γ = 53.3
			$S_1 \rightarrow S_0$ (emission) 442 (0.24) (0.2179)	a = 1.459<br>b = 1.428<br>c = 1.403	α = -25.1<br>β = 29.0<br>γ = 45.5
		BMK	$S_0 \rightarrow S_1$ (absorption)	a = 1.495<br>b = 1.493<br>c = 1.425	α = -42.3<br>β = 47.5<br>γ = 55.7

**4**	**7a**	cam-B3LYP	$S_0 \rightarrow S_1$ (absorption)	a = 1.491<br>c = 1.422	α = -104.7<br>γ = 38.2
			$S_1 \rightarrow S_0$ (emission) 434 (0.16) (0.0330)	a = 1.446<br>c = 1.405	α = -137.3<br>γ = 38.0
		BMK	$S_0 \rightarrow S_1$ (absorption)	a = 1.497<br>c = 1.419	α = -111.2<br>γ = 38.0
**5**	**7b**	cam-B3LYP	$S_0 \rightarrow S_1$ (absorption)	a = 1.484<br>b = 1.486<br>c = 1.426	α = 50.8<br>β = 51.9<br>γ = 54.3
			$S_1 \rightarrow S_0$ (emission) 441 (0.21) (0.1881)	a = 1.459<br>b = 1.430<br>c = 1.403	α = 36.7<br>β = 30.4<br>γ = 46.0
		BMK	$S_0 \rightarrow S_1$ (absorption)	a = 1.491<br>b = 1.493<br>c = 1.425	α = 47.3<br>β = 46.4<br>γ = 56.5
**6**	**7c**	cam-B3LYP	$S_0 \rightarrow S_1$ (absorption)	a = 1.490<br>b = 1.487<br>c = 1.426	α = 67.7<br>β = 51.0<br>γ = 54.4
			$S_1 \rightarrow S_0$ (emission) 424 (0.19) (0.1529)	a = 1.471<br>b = 1.432<br>c = 1.403	α = 50.4<br>β = 31.6<br>γ = 46.0
		BMK	$S_0 \rightarrow S_1$ (absorption)	a = 1.495<br>b = 1.494<br>c = 1.425	α = 57.6<br>β = 44.4<br>γ = 56.6

7.10 NTO analysis and excited-state character as well as charge-transfer analysis exemplified for compound 6c and 7c

Table S15. TD-DFT calculated natural transition orbitals (NTOs) (Gaussian 16, cam-B3LYP/6-311+G*, PCM toluene) of compounds **6** and **7** using Multiwfn[13].

entry	compound	transition	NTO contribution
**1**	**6a**	$S_0 \rightarrow S_1$ (absorption)	0.927504
		$S_1 \rightarrow S_0$ (emission)	0.936615
**2**	**6b**	$S_0 \rightarrow S_1$ (absorption)	0.949214<br>0.008415
		$S_1 \rightarrow S_0$ (emission)	0.953105
**3**	**6c**	$S_0 \rightarrow S_1$ (absorption)	0.928704
		$S_1 \rightarrow S_0$ (emission)	0.957167
**4**	**7a**	$S_0 \rightarrow S_1$ (absorption)	0.949166
		$S_1 \rightarrow S_0$ (emission)	0.954465
**5**	**7b**	$S_0 \rightarrow S_1$ (absorption)	0.918807
		$S_1 \rightarrow S_0$ (emission)	0.957939
**6**	**7c**	$S_0 \rightarrow S_1$ (absorption)	0.920491
		$S_1 \rightarrow S_0$ (emission)	0.957529

To further elucidate the electronic nature of the lowest excited states of the sterically hindered compounds **6c** and **7c**, complementary natural transition orbital (NTO), hole–electron, and interfragment charge-transfer (IFCT) analyses were performed for the corresponding absorption transitions using the BMK functional and Multiwfn[13] as analyzer. The results are summarized in Table S16. In both compounds, the excitation is dominated by a single NTO pair, accounting for 97.7% and 97.6% of the transition for **6c** and **7c**, respectively. This indicates that the electronic structure of the excited states can be described to a very good approximation by one predominant hole–particle transition.

The hole–electron analysis provides further indication for a pronounced redistribution of electron density upon excitation. For **6c**, the hole–electron centroid distance Δr is 3.214 Å, indicating a substantial spatial displacement between the centroids of the hole and electron distributions. The corresponding CT-related spatial extent H_{CT} is 2.279 Å, reflecting the spatial extent of the charge redistribution associated with the excitation. The t index amounts to 0.934 Å and provides a measure of the degree of spatial separation between the hole and electron distributions relative to their respective spatial extents. The positive t value therefore indicates a distinct spatial separation of the two distributions. For **7c**, slightly smaller values of Δr = 3.014 Å, H_{CT} = 2.127 Å, and t = 0.887 Å are obtained, suggesting a less pronounced spatial separation and charge-transfer character than in **6c**. Despite this spatial separation, the hole–electron overlap index S_r remains appreciable, with values of 0.421 and 0.424 for **6c** and **7c**, respectively. As S_r quantifies the spatial overlap between the hole and electron distributions, these values indicate that the two distributions retain a considerable degree of spatial coincidence. Thus, the excitations cannot be regarded as purely charge-separated states. Rather, the combination of substantial centroid separation and a non-negligible hole–electron overlap points toward an excitation

with a pronounced charge-transfer contribution while retaining a significant locally excited component.[13-15]

The fragment-resolved analysis provides a more detailed picture of this mixed electronic character (Figure 12). In both compounds, the hole is strongly localized on fragment F1 (triphenylamine donor), accounting for 93.5% of the total hole distribution in both **6c** and **7c**. In contrast, the excited electron is distributed over all three fragments. For **6c**, the electron distribution amounts to 39.6% on F1, 33.4% on F2 (phenylene bridge), and 19.5% on F3 (acceptor), whereas the corresponding values for **7c** are 43.6% (F1), 32.8% (F2), and 16.2% (F3), respectively. Hence, excitation predominantly removes electron density from F1, while the resulting electron density is redistributed over F1–F3. In particular, the substantial electron populations on F2 and F3 are consistent with the spatial charge redistribution indicated by the hole–electron analysis and demonstrate that the excitation is not confined to a single molecular fragment.

The IFCT analysis quantitatively corroborates this interpretation. The intrinsic CT contributions amount to 50.5% for **6c** and 46.8% for **7c**, whereas the corresponding intrinsic local-excitation (LE) contributions are 37.6% (**6c**) and 41.3% (**7c**), respectively. Both excited states therefore contain substantial CT and LE contributions, with the CT component being somewhat more pronounced in **6c**. The simultaneous presence of significant CT and LE contributions is characteristic of a hybridized local-excitation/charge-transfer (HLCT) character rather than a purely locally excited or purely charge-transfer character.

The calculated interfragment charge-transfer pathways provide further quantitative support for this assignment. For **6c**, the dominant pathways are F1→F2 (0.306 *e*) and F1→F3 (0.181 *e*), while for **7c** they amount to 0.300 *e* and 0.151 *e*, respectively. The consistently dominant F1→F2 pathway identifies F2 as the dominant electron accepting region, whereas the additional F1→F3 transfer demonstrates that the excited electron is distributed over more than one fragment. Notably, substantial interfragment charge transfer is maintained in both sterically hindered compounds despite their different structural environments.

Overall, the NTO, hole–electron, fragment-resolved, and IFCT analyses provide a consistent picture of the excited-state electronic structure of **6c** and **7c**. The nearly exclusive contribution of a single NTO pair indicates a well-defined dominant excitation, while the relatively large Δr and H_{CT} values, together with positive t values, reveal pronounced spatial charge redistribution. At the same time, the appreciable S_r values demonstrate that the hole and electron distributions retain significant spatial overlap, excluding a description as purely charge-separated states. This interpretation is further supported by the localization of the hole on F1, the delocalization of the excited electron over F1–F3, and the substantial interfragment CT pathways. Most importantly, the IFCT analysis quantitatively establishes the coexistence of significant CT and LE contributions, supporting the assignment of HLCT character. The overall electronic characteristics are highly similar for the two compounds, although **6c** exhibits a comparatively stronger CT contribution, larger Δr, H_{CT}, and t values, and more pronounced F1→F3 charge transfer, whereas **7c** shows a slightly larger LE contribution and marginally greater hole–electron overlap.

Table S16. TD-DFT calculated natural transition orbitals (NTOs) and hole–electron distribution parameters (Gaussian 16, BMK/6-311+G*, PCM toluene) of compounds **6** and **7** using Multiwfn[13].

entry	parameters	compound 6c	compound 7c
**1**	single dominant NTO pair contribution [%]	97.73	97.60
**2**	hole–electron centroid distance Δr [Å]	3.214	3.014
**3**	CT-related spatial extent H_{CT} [Å]	2.279	2.127
**4**	t index [Å]	0.934	0.887
**5**	hole–electron overlap index S_r	0.421	0.424
**6**	intrinsic CT contribution [%]	50.45	46.82
**7**	intrinsic LE contribution [%]	37.57	41.31
**8**[a]	hole distribution on fragments [%]	93.54 (F1)<br>1.54 (F2)<br>0.14 (F3)	93.54 (F1)<br>1.52 (F2)<br>0.13 (F3)
**9**[a]	electron distribution on fragments [%]	39.58 (F1)<br>33.40 (F2)<br>19.45 (F3)	43.61 (F1)<br>32.75 (F2)<br>16.23 (F3)
**10**[a]	fragment-resolved hole–electron overlap [%]	60.85 (F1)<br>7.17 (F2)<br>1.67 (F3)	63.87 (F1)<br>7.05 (F2)<br>1.43 (F3)
**11**	CT amount [e] and pathways	0.306 (F1→F2)<br>0.181 (F1→F3)	0.300 (F1→F2)<br>0.151 (F1→F3)

[a] Values do not sum up to 100% due to fragment definitions without including H atoms.

7.11 Frontier molecular orbital contributions to transitions to excited states and orbital energy differences in toluene

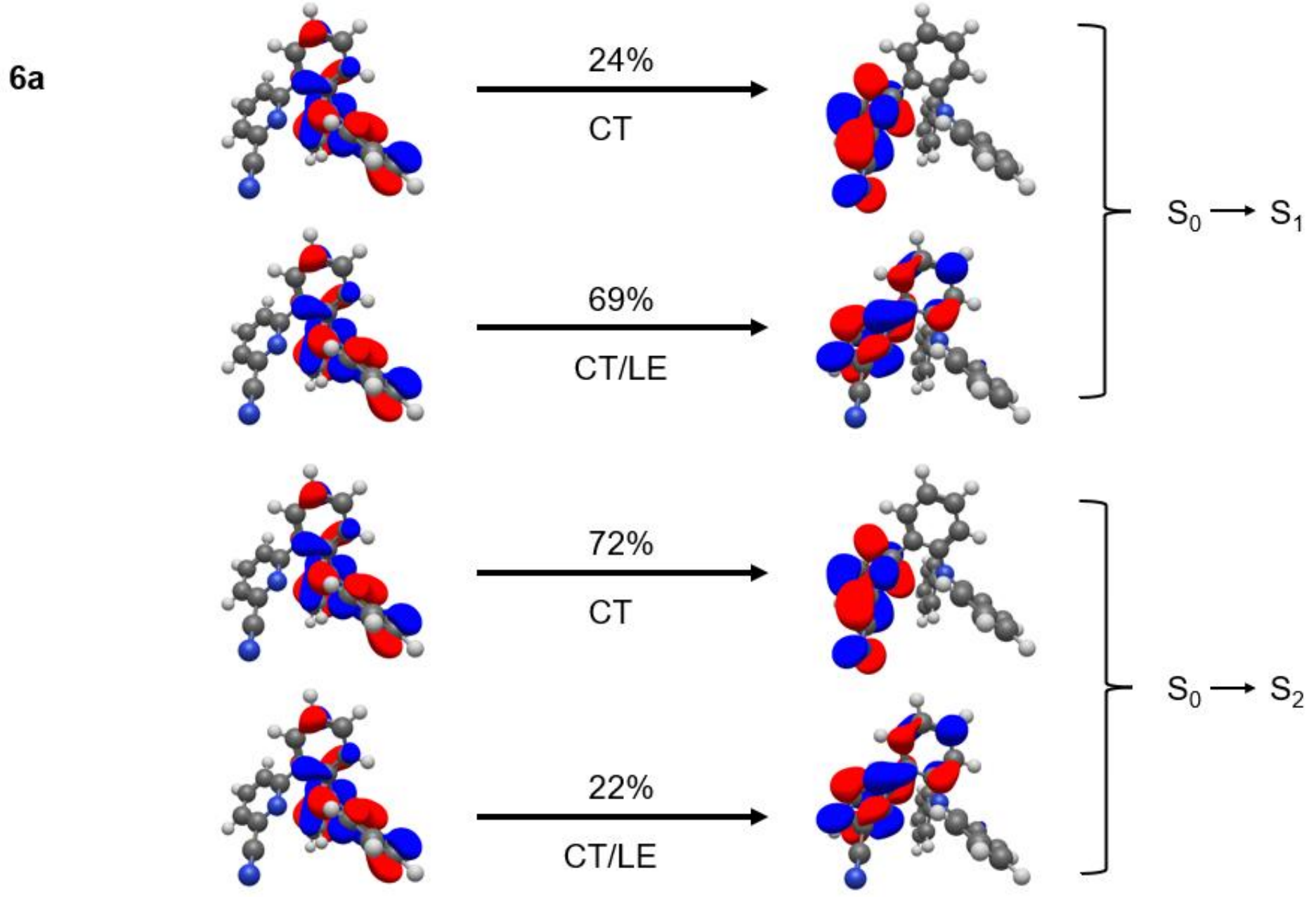

Figure S87. Frontier molecular orbital contributions to the TDDFT-computed longest absorption transition to the first and second excited state (S_0 to S_1^* and S_0 to S_2^*) of compound **6a** (Gaussian 16, cam-B3LYP/6-311+G*, PCM toluene, isosurface value at 0.03).

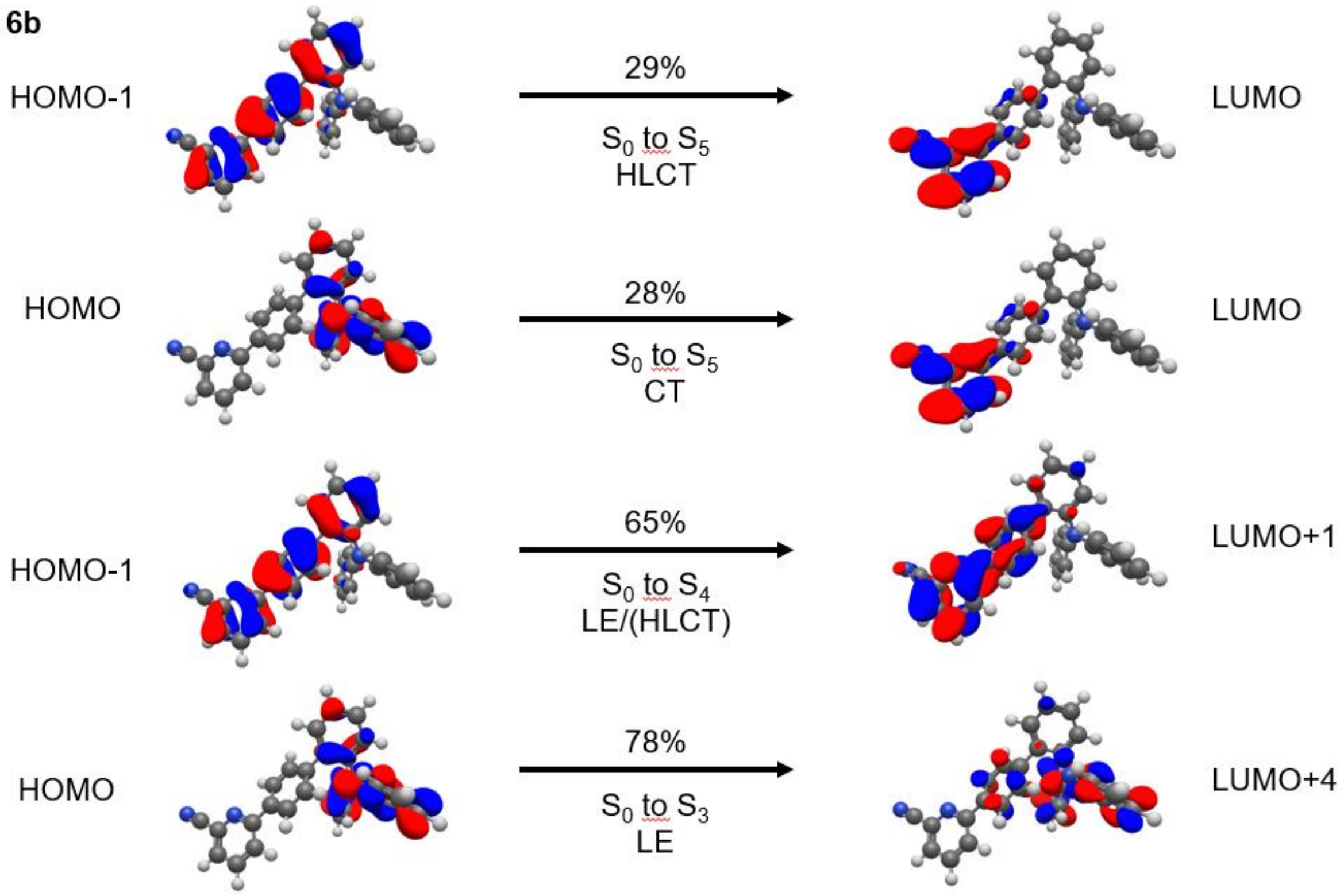

Figure S88. Frontier molecular orbital contributions to the TD-DFT-computed absorption transitions combining into the absorption band at higher energy of compound **6b** (Gaussian 16, cam-B3LYP/6-311+G*, PCM toluene, isosurface value at 0.03).

6b

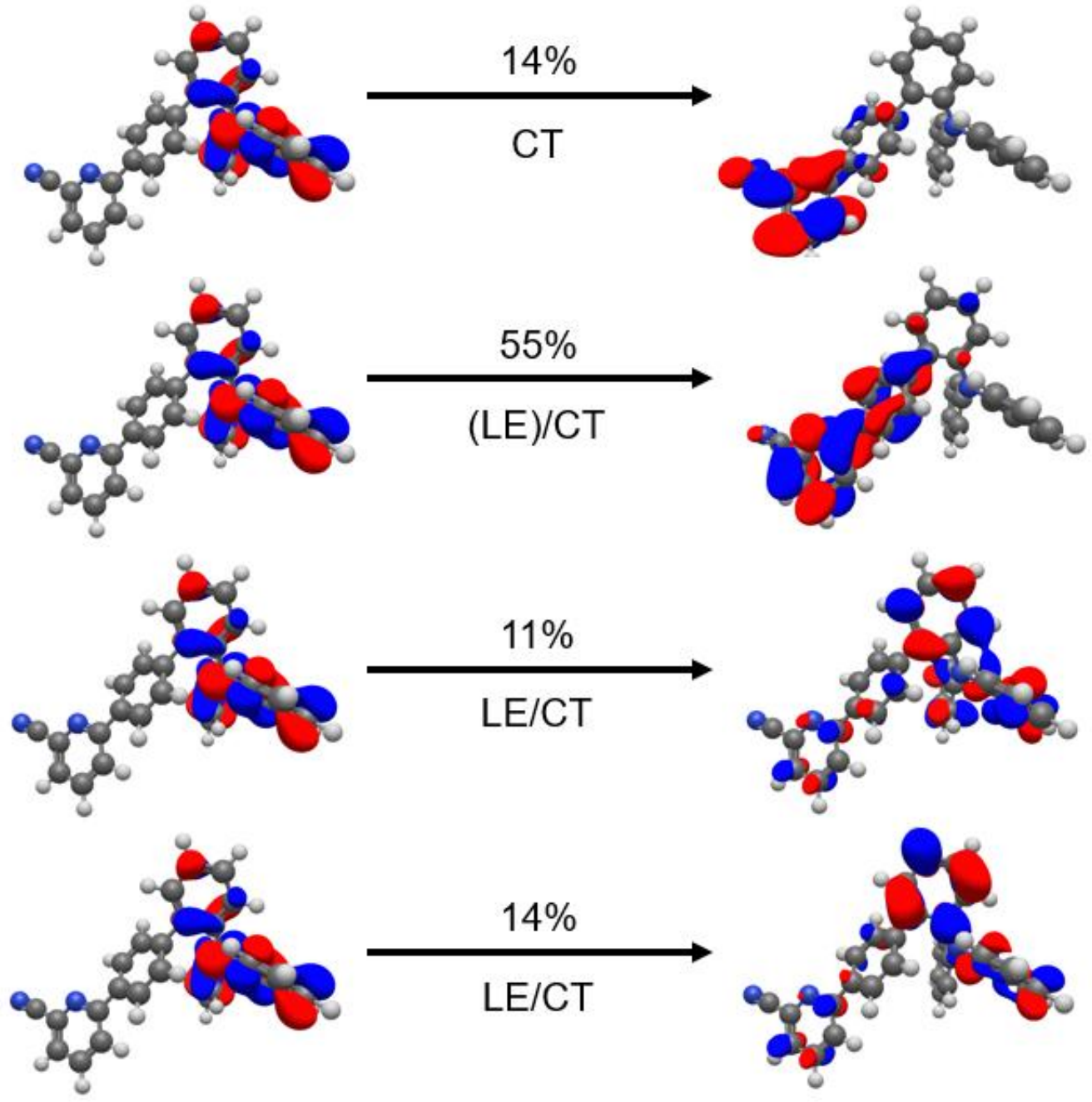

Figure S89. Frontier molecular orbital contributions to the TDDFT-computed longest absorption transition to the first excited state (S_0 to S_1^*) of compound **6b** (Gaussian 16, cam-B3LYP/6-311+G*, PCM toluene, isosurface value at 0.03).

6c

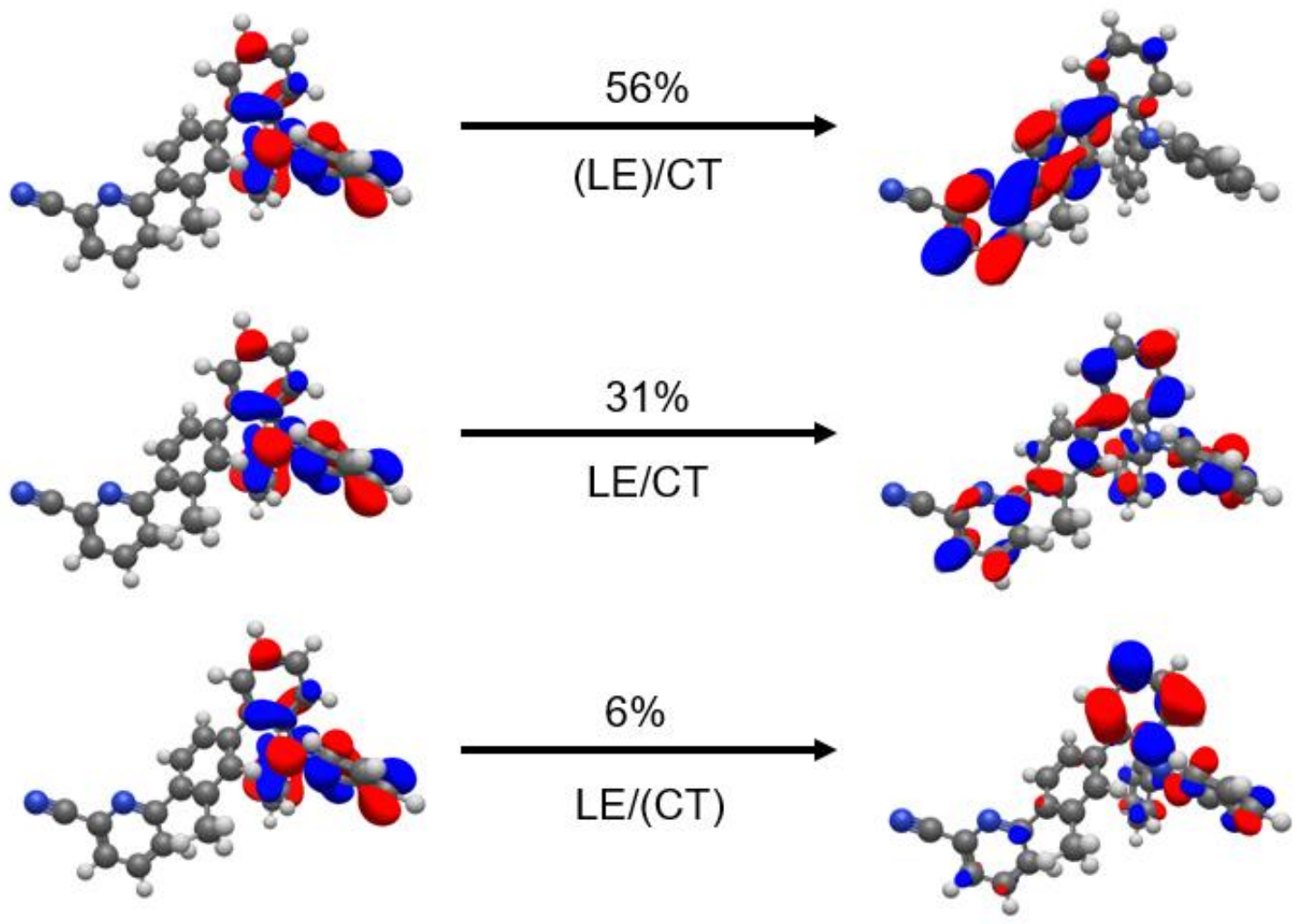

Figure S90. Frontier molecular orbital contributions to the TDDFT-computed longest absorption transition to the first excited state (S_0 to S_1^*) of compound **6c** (Gaussian 16, cam-B3LYP/6-311+G*, PCM toluene, isosurface value at 0.03).

7a

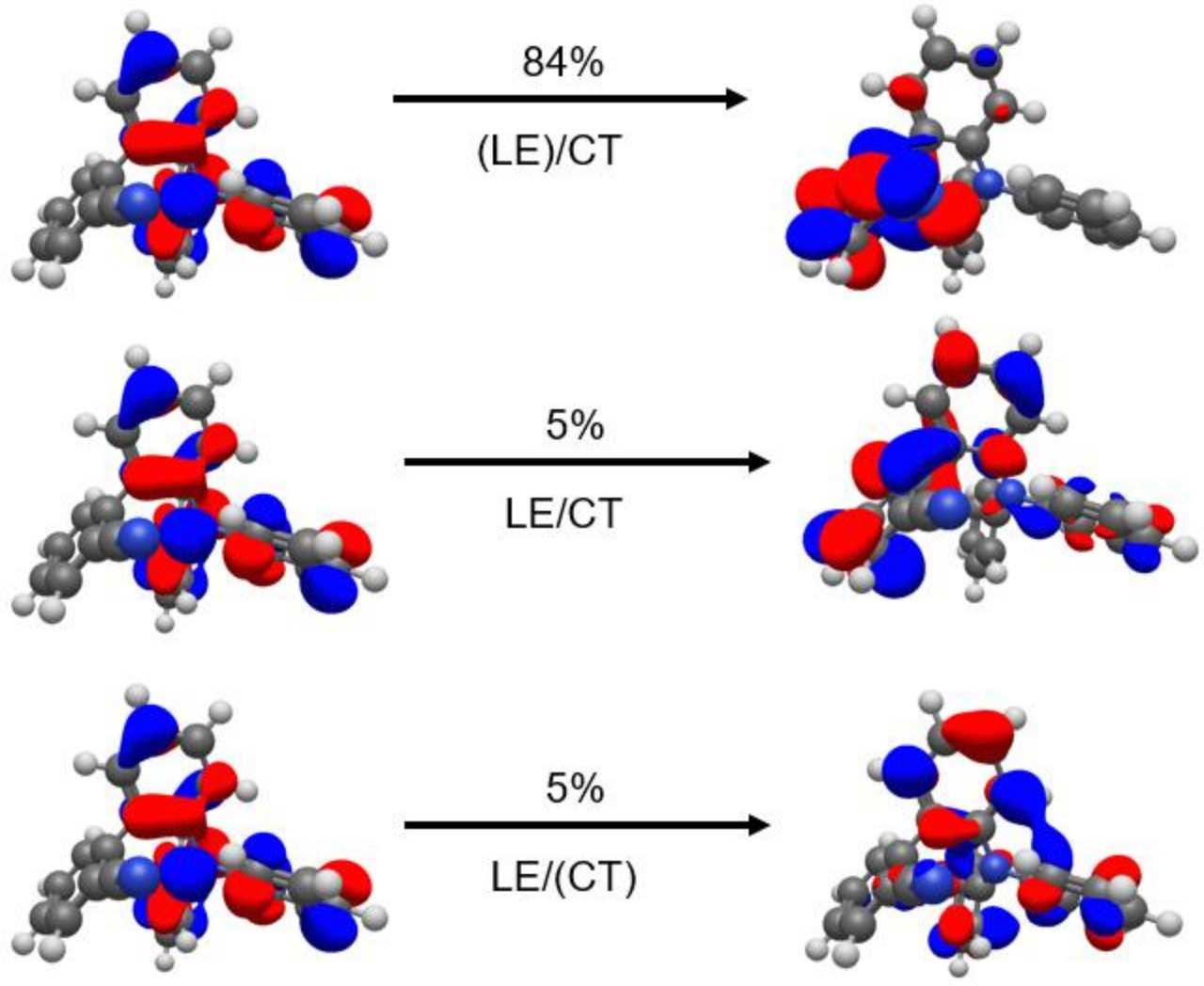

Figure S91. Frontier molecular orbital contributions to the TDDFT-computed longest absorption transition to the first excited state (S_0 to S_1^*) of compound **7a** (Gaussian 16, cam-B3LYP/6-311+G*, PCM toluene, isosurface value at 0.03).

7b

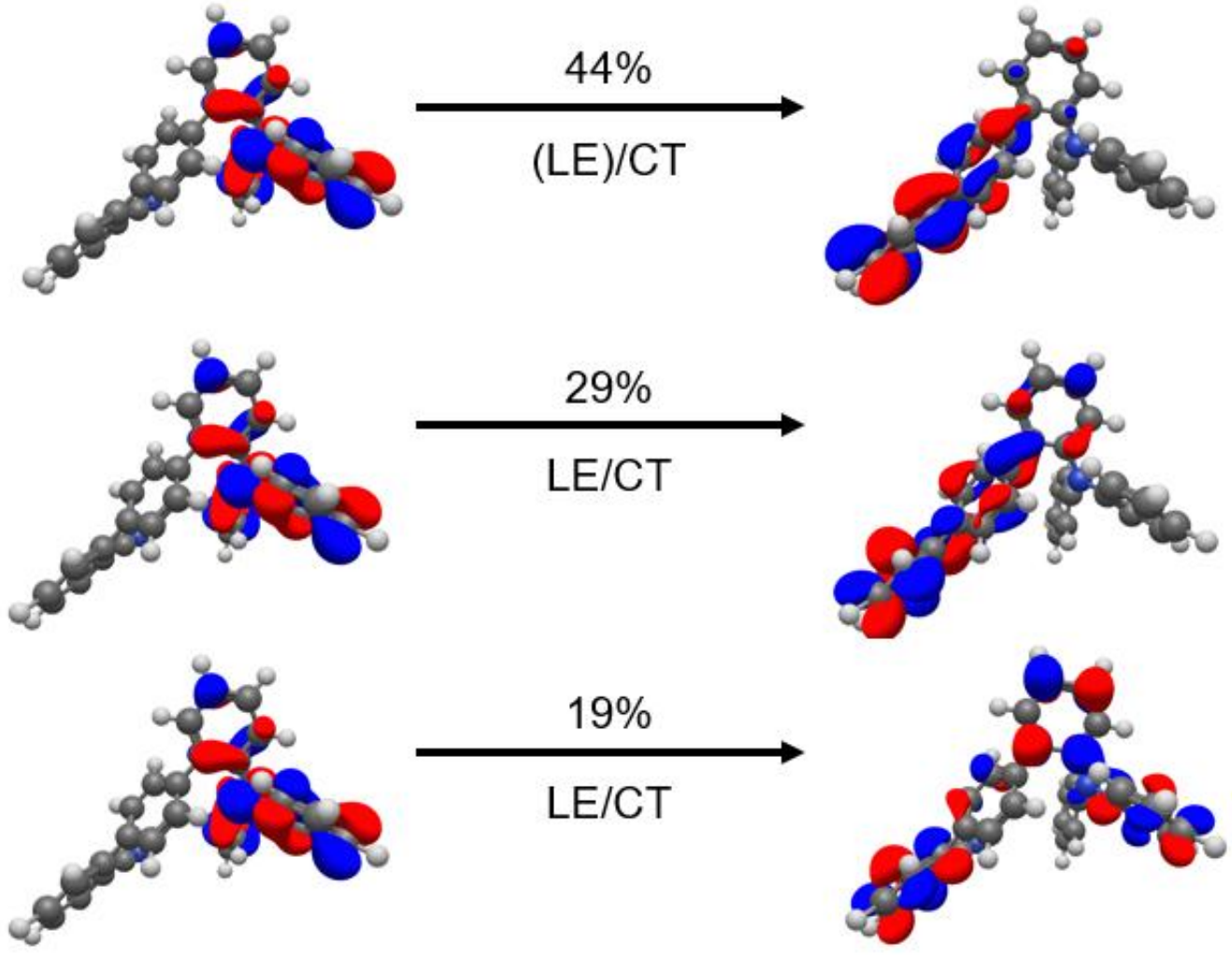

Figure S92. Frontier molecular orbital contributions to the TDDFT-computed longest absorption transition to the first excited state (S_0 to S_1^*) of compound **7b** (Gaussian 16, cam-B3LYP/6-311+G*, PCM toluene, isosurface value at 0.03).

7c

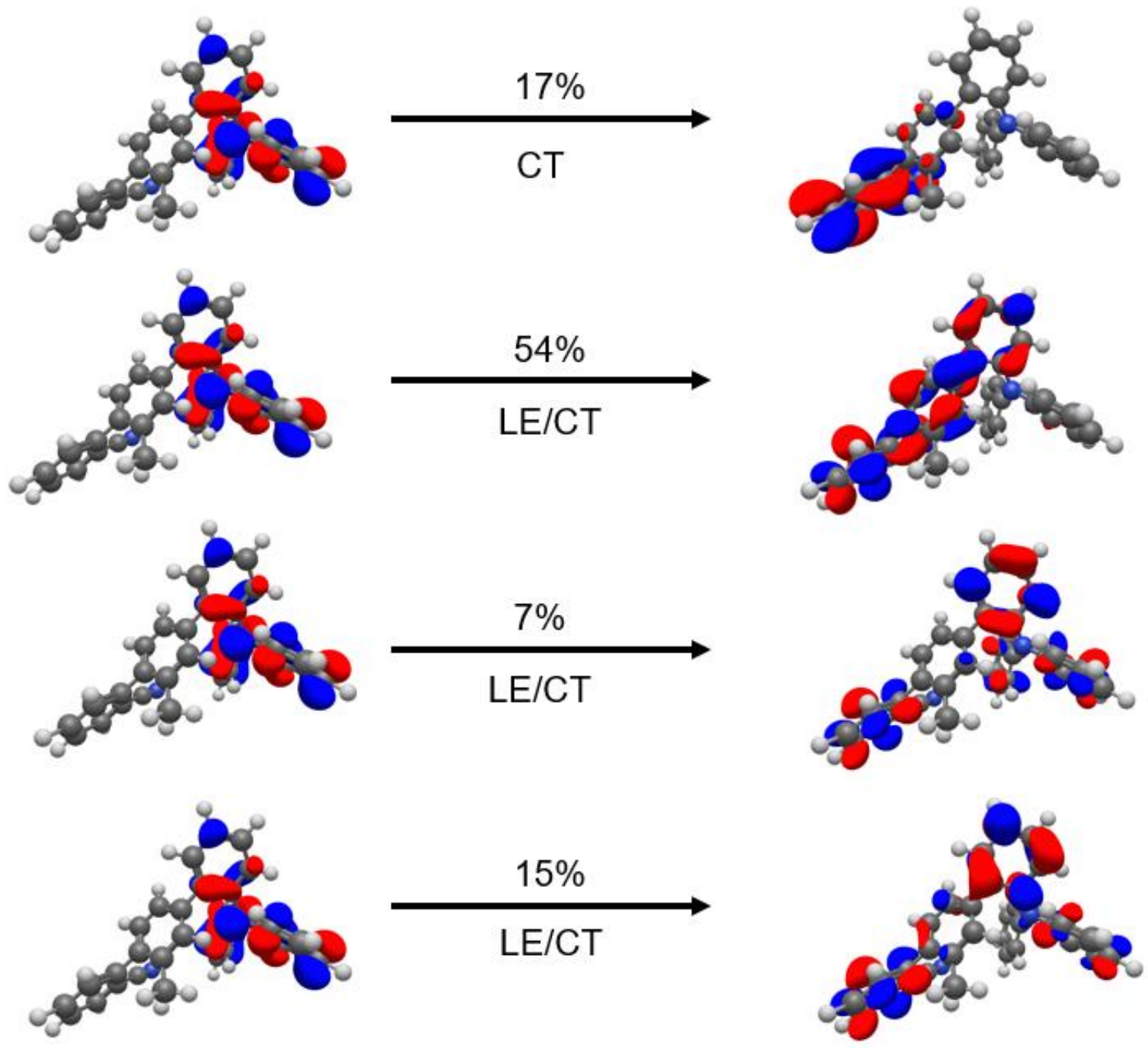

Figure S93. Frontier molecular orbital contributions to the TDDFT-computed longest absorption transition to the first excited state (S_0 to S_1^*) of compound **7c** (Gaussian 16, cam-B3LYP/6-311+G*, PCM toluene, isosurface value at 0.03).

6a

6b

6c

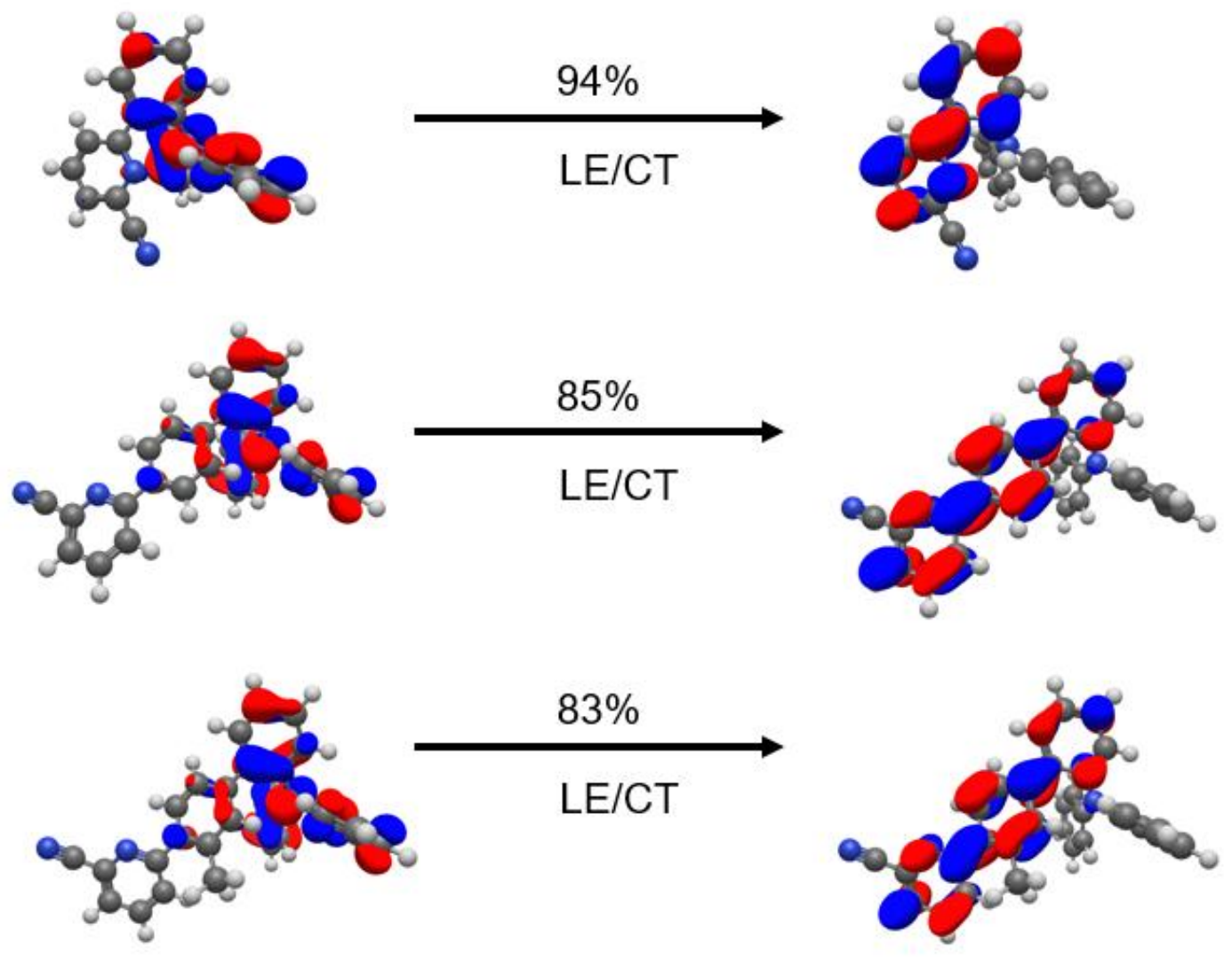

Figure S94. Frontier molecular orbital contributions to the TD-DFT-computed fluorescence from the first excited state to the ground state (S_1 to S_0^*) of compounds **6** (Gaussian 16, cam-B3LYP/6-311+G*, PCM toluene, isosurface value at 0.03).

7a

7b

7c

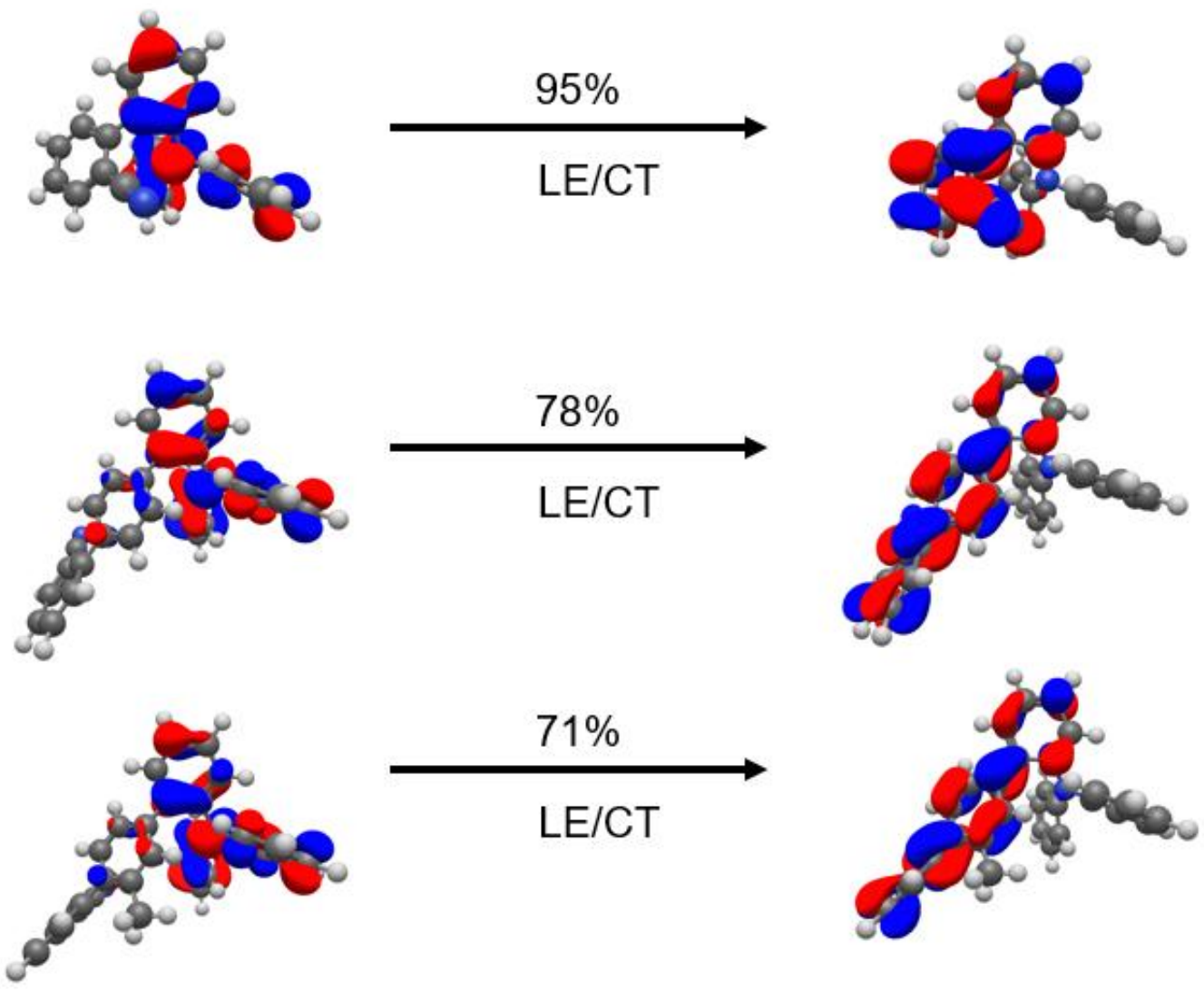

Figure S95. Frontier molecular orbital contributions to the TD-DFT-computed fluorescence from the first excited state to the ground state (S_1 to S_0^*) of compounds **7** (Gaussian 16, cam-B3LYP/6-311+G*, PCM toluene, isosurface value at 0.03).

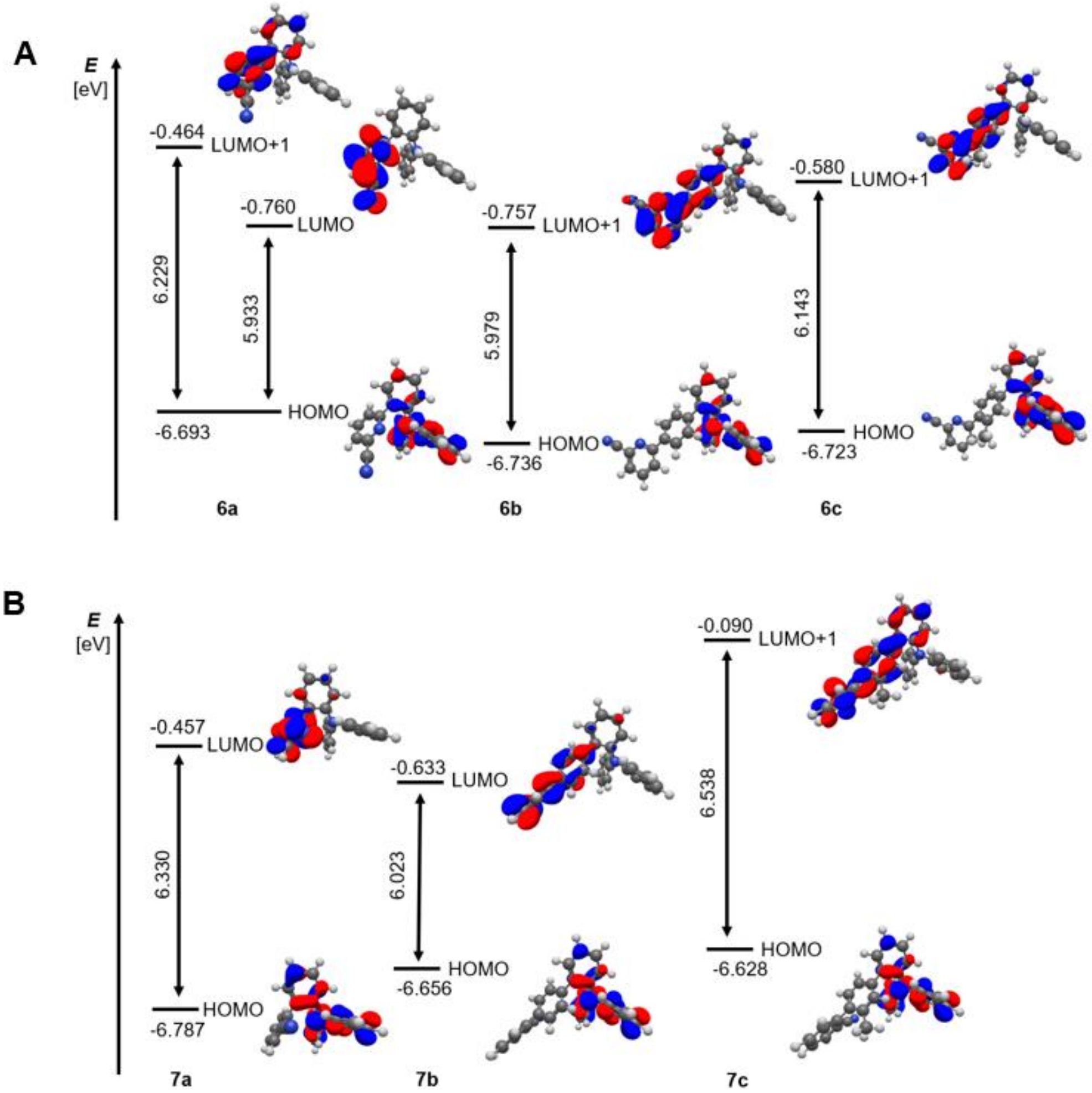

Figure S96. Kohn-Sham frontier molecular orbitals and calculated HOMO and LUMO/LUMO+1 energies including energy differences of the longest wavelength absorption of cyanopyridine-based **6** (**A**) and benzonitrile-based donor-acceptor conjugates **7** (**B**) (Gaussian 16, cam-B3LYP/6-311+G*, PCM toluene, isosurface value at 0.03).

8 References

[1] Y. Kanazawa, T. Yokota, H. Ogasa, H. Watanabe, T. Hanakawa, S. Soga, M. Kawatsura, Tetrahedron **2015**, 71 (9), 1395–1402.

[2] W. E. G. N. G. Connelly, *Chem. Rev.* **1996**, *96*, 877–910.

[3] M. J. Frisch, G. W. Trucks, H. B. Schlegel, G. E. Scuseria, M. A. Robb, J. R. Cheeseman, G. Scalmani, V. Barone, G. A. Petersson, H. Nakatsuji, X. Li, M. Caricato, A. V. Marenich, J. Bloino, B. G. Janesko, R. Gomperts, B. Mennucci, H. P. Hratchian, J. V. Ortiz, A. F. Izmaylov, J. L. Sonnenberg, D. Williams-Young, F. Ding, F. Lipparini, F. Egidi, J. Goings, B. Peng, A. Petrone, T. Henderson, D. Ranasinghe, V. G. Zakrzewski, J. Gao, N. Rega, G. Zheng, W. Liang, M. Hada, M. Ehara, K. Toyota, R. Fukuda, J. Hasegawa, M. Ishida, T. Nakajima, Y. Honda, O. Kitao, H. Nakai, T. Vreven, K. Throssell, J. J. A. Montgomery, J. E. Peralta, F. Ogliaro, M. J. Bearpark, J. J. Heyd, E. N. Brothers, K. N. Kudin, V. N. Staroverov, T. A. Keith, R. Kobayashi, J. Normand, K. Raghavachari, A. P. Rendell, J. C. Burant, S. S. Iyengar, J. Tomasi, M. Cossi, J. M. Millam, M. Klene, C. Adamo, R. Cammi, J. W. Ochterski, R. L. Martin, K. Morokuma, O. Farkas, J. B. Foresman, D. J. Fox, *Gaussian 16, Revision A.03*, Gaussian, Inc., Wallingford CT, **2016**.

[4] E. Grimley, D. H. Collum, E. G. Alley, B. the late Layton, *Org. Magn. Reson.* **1981**, *15*, 296–302.

[5] O. Yurchenko, D. Freytag, L. zur Borg, R. Zentel, J. Heinze, S. Ludwigs, *J. Phys. Chem.* **2012**, 116, 30–39.

[6] R. Kohlbecher, M. Flörke, D. Kempe, T. J. J. Müller, *RSC Adv.* **2026**, 16, 37734–37745.

[7] R. Kohlbecher, H. Schröder, D. N. Jordan, C. Janiak, T. J. J. Müller, *Eur. J. Org. Chem.* **2026**, *29*, 1–27.

[8] F. F. Nielson, S. M. Colby, D. G. Thomas, R. S. Renslow, T. O. Metz, *Anal Chem.* **2021**, 8, 3830–3838.

[9] T. J. Dwyer, P. G. Jasien, *Theochem* **1996**, 363, 139–150.

[10] C. Reichardt, T. Welton, Solvents and Solvent Effects in Organic Chemistry; Wiley VCH Verlag GmbH & Co. KGaA, Weinheim, Germany, **2010**, 455–460.

[11] C. Reichardt, T. Welton, Solvents and Solvent Effects in Organic Chemistry; Wiley VCH Verlag GmbH & Co. KGaA, Weinheim, Germany, **2010**, 549–589.

[12] a) J. G. Kirkwood, *J. Chem. Phys.* **1934**, 2, 351–361. b) L. Onsager, *J. Am. Chem. Soc.* **1936**, 58, 1486–1493. c) M. W. Wong, M. J. Frisch, K. B. Wiberg, *J. Am. Chem. Soc.* **1991**, 113, 4776–4782. d) M. W. Wong, K. B. Wiberg, M. Frisch, *J. Chem. Phys.* **1991**, 95, 8991–8998. e) M. W. Wong, K. B. Wiberg, M. J. Frisch, *J. Am. Chem. Soc.* **1992**, 114, 523–529. f) M. W. Wong, K. B. Wiberg, M. J. Frisch, *J. Am. Chem. Soc.* **1992**, 114, 1645–1652.

[13] T. Lu, F. Chen, *J. Comput. Chem.* **2012**, 33, 580–592.

[14] C. A. Guido, P. Cortona, B. Mennucci, C. Adamo, *J. Chem. Theory Comput*. **2013**, 9, 3118–3126.

[15] Z. Liu, T. Lu, Q. Chen, *Carbon* **2020**, 165, 461–467.
